# Resource use and direct medical costs of acute respiratory illness in the UK based on linked primary and secondary care records from 2001 to 2009

**DOI:** 10.1371/journal.pone.0236472

**Published:** 2020-08-06

**Authors:** Geneviève C. Meier, John Watkins, Phil McEwan, Rhys D. Pockett

**Affiliations:** 1 Health Economics, GSK, Wavre, Belgium; 2 Public Health Medicine, College of Biomedical and Life Sciences, Cardiff University, Cardiff, Wales, United Kingdom; 3 Swansea Centre for Health Economics, Swansea University, Swansea, Wales, United Kingdom; National Center for Global Health and Medicine, JAPAN

## Abstract

**Background:**

Previous studies have shown that influenza is associated with a substantial healthcare burden in the United Kingdom (UK), but more studies are needed to evaluate the resource use and direct medical costs of influenza in primary care and secondary care.

**Methods:**

A retrospective observational database study in the UK to describe the primary care and directly-associated secondary care resource use, and direct medical costs of acute respiratory illness (ARI), according to age, and risk status (NCT Number: 01521416). Patients with influenza, ARI or influenza-related respiratory infections during 9 consecutive pre-pandemic influenza peak seasons were identified by READ codes in the linked Clinical Practice Research Datalink (CPRD) and Hospital Episodes Statistics (HES) dataset. The study period was from 21st January 2001 to 31st March 2009.

**Results:**

A total of 156,193 patients had ≥1 general practitioner (GP) episode of ARI, and a total of 82,204 patients received ≥1 GP prescription, at a mean of 2.5 (standard deviation [SD]: 3.0) prescriptions per patient. The total cost of GP consultations and prescriptions equated to £462,827 per year per 100,000 patients. The yearly cost of prescribed medication for ARI was £319,732, at an estimated cost of £11,596,350 per year extrapolated to the UK, with 40% attributable to antibiotics. The mean cost of hospital admissions equated to a yearly cost of £981,808 per 100,000 patients. The total mean direct medical cost of ARI over 9 influenza seasons was £21,343,445 (SD: £10,441,364), at £136.65 (SD: £66.85) per case.

**Conclusions:**

Extrapolating to the UK population, for pre-pandemic influenza seasons from 2001 to 2009, the direct medical cost of ARI equated to £86 million each year. More studies are needed to assess the costs of influenza disease to help guide public health decision-making for seasonal influenza in the UK.

## Introduction

The resource use and costs of seasonal influenza epidemics remains substantial despite many countries recommending annual vaccination for either specific population groups that are vulnerable to infection (such as children, elderly adults, and anyone with chronic conditions), or universal vaccination such as in the United States (US), Canada, and Australia.[1–4] However, measuring the value of influenza vaccination is challenging because the clinical and economic effect depends on the epidemic intensity of the influenza season, and the degree of vaccine match with circulating viruses.

National influenza vaccination policies are generally guided by studies that have evaluated influenza costs or the cost-effectiveness of influenza vaccination in different settings and patient groups; in a review of 140 studies published up to 2012, the per capita cost of a case of influenza illness ranged from $30 to $64, and 22 studies reported that influenza vaccination was cost-saving.[5] In the United Kingdom (UK) publically-funded influenza vaccination is recommended for people aged ≥65 years, pregnant women, carers and health and social care workers, and anyone at increased risk of influenza infection or complications such as people with asthma, diabetes, cardiovascular disease, or kidney disease.[4] The paediatric recommendation for publically-funded vaccination in the UK is currently all children aged from 2 to 7 years/school year 3, and children aged ≥6 months if they have chronic health conditions.[4]

The publically-funded vaccination policy in the UK is supported by various analyses that have assessed the burden of influenza illness and costs in the UK.[6–12] This includes a study representing about 52.6 million people over 14 influenza seasons (1995–2009) in England and Wales, which showed that vaccinating people aged ≥65 years and people at high-risk of influenza disease was likely to be cost-effective (incremental cost-effectiveness ratio [ICER]: £7,475/QALY).[8] The analysis also showed that vaccinating low-risk groups would also likely to be cost-effective, with the highest net benefit achieved by including all children in the vaccination programme.[8]

To help guide public health decision-making, more studies are needed that assess the burden and cost of influenza disease in the UK including episodes of influenza in primary care and secondary care, and an evaluation of risk factors associated with influenza episodes. To address this, we conducted a retrospective, cross-sectional, observational study to evaluate the primary care and directly-associated secondary care resource use, and direct medical costs of acute respiratory illness (ARI) in patients with READ codes for acute respiratory illnesses in the linked Clinical Practice Research Datalink (CPRD) and Hospital Episodes Statistics (HES) dataset during influenza seasons between 2001 and 2009. Data from the CPRD/HES dataset, the Health Protection Agency (HPA), and the Office of National Statistics (ONS), were used to identify patients with ≥1 general practitioner episode of ARI (i.e. primary care) and patients with linked secondary care data. The first analysis from the study provided data on vaccination uptake and direct medical costs by vaccination status and degree of vaccine mismatch.[13] Here we report the resource use and direct medical costs of GP episodes of ARI and hospital admissions in the total population, and according to age and risk status.

## Methods

### Design

This retrospective, cross-sectional, observational study was conducted to evaluate the resource use and direct medical costs of GP episodes of ARI and linked hospital admissions during influenza seasons in the study period of 21st January 2001 and 31st March 2009 in the UK (NCT Number: 01521416). Approval for this study was obtained from the CPRD Independent Scientific Advisory Committee (11_065) negating the need for individual patient consent. The full methods are provided in S1 Data and are published elsewhere.[13]

### Case definitions and data extraction

Fully anonymised data on patients who had ≥1 GP episode with a READ code identifying influenza like illnesses (ARI) were extracted from the linked Clinical Practice Research Datalink (CPRD)/Health Episode Statistics (HES) dataset for the study. Cases of ARI were identified using a broad range of READ codes describing influenza, influenza-like illness, or acute upper or lower respiratory tract infection with influenza. For each influenza peak season, ARI episodes recorded in the CPRD were identified.

For patients with linked data, the HES record was assessed for hospital admissions for influenza or a complication following influenza, including data for 14 days before and after the index ARI episode. Hospitalised patients were assessed for 28 days post-discharge.

Patients were categorised as low- or high-risk for influenza infection and related complications using criteria from the National Institute for Health and Care Excellence (NICE) and the UK Department of Health.[4, 14]

READ codes for ARI in the CPRD and READ codes for high-risk conditions are shown in S1 Data; Appendices 1 and 3 in S1 Data.

### Influenza peak seasons analysed

The study was conducted from 2001 to 2009 covering 9 influenza seasons, up to the outbreak of pandemic A(H1N1)pdm09 in the UK on the 27th of April 2009. GP episodes with any relevant READ code recorded during periods of influenza peak season each year were included in the study. The start and end of influenza peak season in each year was based on laboratory-confirmed reports of respiratory pathogens circulating in the UK from the Health Protection Agency (HPA) for the period November 2002 to December 2008.[15] Peak influenza season was based on the weeks during which there was high viral circulation and ARI clinical events based on HPA laboratory reports for influenza A and B and other respiratory infections.

### Outcome measures

#### Burden of ARI

The outcome measures were the incidence of: ARI GP episodes, linked hospital admissions, death, complications, and high-risk conditions: respiratory, central nervous system, diabetes, chronic heart disease, pregnant, liver disease, renal disease, and immunocompromised. The outcomes were assessed in the total population including all patients with ≥1 ARI GP episode and the hospitalized population including patients with an index ARI episode and a subsequent hospital admission. Subgroup analyses were performed by age (<5 years; 5–18 years; 19–49 years; 50–65 years, ≥65 years), complication status (complicated cases and uncomplicated cases), and clinical risk group (low-risk and high-risk). Mortality was classified as being without hospital admission, in hospital, or within 28 days of discharge from hospital.

#### Resource use and costs

GP consultations for ARI were classified as resource use in ‘primary care’. Prescriptions for medications from GPs were also classified as resource use in ‘primary care’. Medications were assessed by drug category: antipyretics/analgesics, antibiotics, amantadine, aminoglycosides, nasal decongestants, antisecretory drugs and mucosal protectants, and antihistamines. Antibiotics were assessed by class. Resource use was classified as hospitalization if it was associated with patients who were admitted to hospital or who attended a hospital out-patient appointment. Hospital resource use outcomes were: the incidence of hospital admission, mean number of unique admissions, mean length of stay per hospitalization, mean number of out-patient appointments, and route of hospital admission (A&E, GP referral, or other). Medications prescribed in hospital were not captured. The incidence of GP referrals to ambulatory out-patient appointments was also assessed.

The study was based on National Health Service (NHS) records from primary and secondary care providers in the UK. The cost perspective was that of the NHS and included all primary care and hospital admissions for ARI. GP consultation costs and out-patient visit costs were from the Personal Social Services Research Unit (PSSRU) database:[16] GP consultation £36, out-patient care £147, and hospital admission £686. Unit costs for hospitalizations were derived from the NHS secondary care tariff for healthcare-related groups (HRG). All HRG codes for a complication relevant to the study were grouped together to calculate a mean unit cost for that complication. The analysis was based on 2011 costs expressed in £ (pounds sterling).

### Data analysis

No comparisons were made between study groups so there was no target sample size. All eligible patients within the database were used in the analysis. Raw data were cleaned before analysis. It was assumed that a patient could experience only one ARI GP episode and one hospitalization on one day. Data were extracted using MySQL, and analyses were conducted in *Microsoft Access*, *Microsoft Excel* and *Statistical Package for the Social Sciences* (SPSS).

The burden of ARI, resource use, and costs were analysed descriptively in the total population and by age, complication status, and risk status. The burden and resource use data were reported as the number of patients and as a percentage of the total population. The data were also provided as the incidence of the outcome per 100,000 population in the CPRD. The data was extrapolated to the UK population using ONS; population estimates by year, and by age group are publicly available and provided end of year estimates of the resident population from 2001 to 2009:[17] Calculated from the CPRD/HES data extract, the yearly average number of patients in the CPRD/HES database in 2000–2009 was 1,662,953, and the average UK population in 2001–2009 was 60,313,633, giving a multiplying factor of 36.3 which was used to extrapolate the study data since the CPRD database was shown to be broadly representative of the UK population [18]. The burden, resource use, and costs were assessed over the study period (9 seasons) and for each season. Summary statistics were provided as mean values and standard deviation (SD) for continuous variables and frequency distributions for categorical variables.

## Results

### Primary care

**Fig 1:**
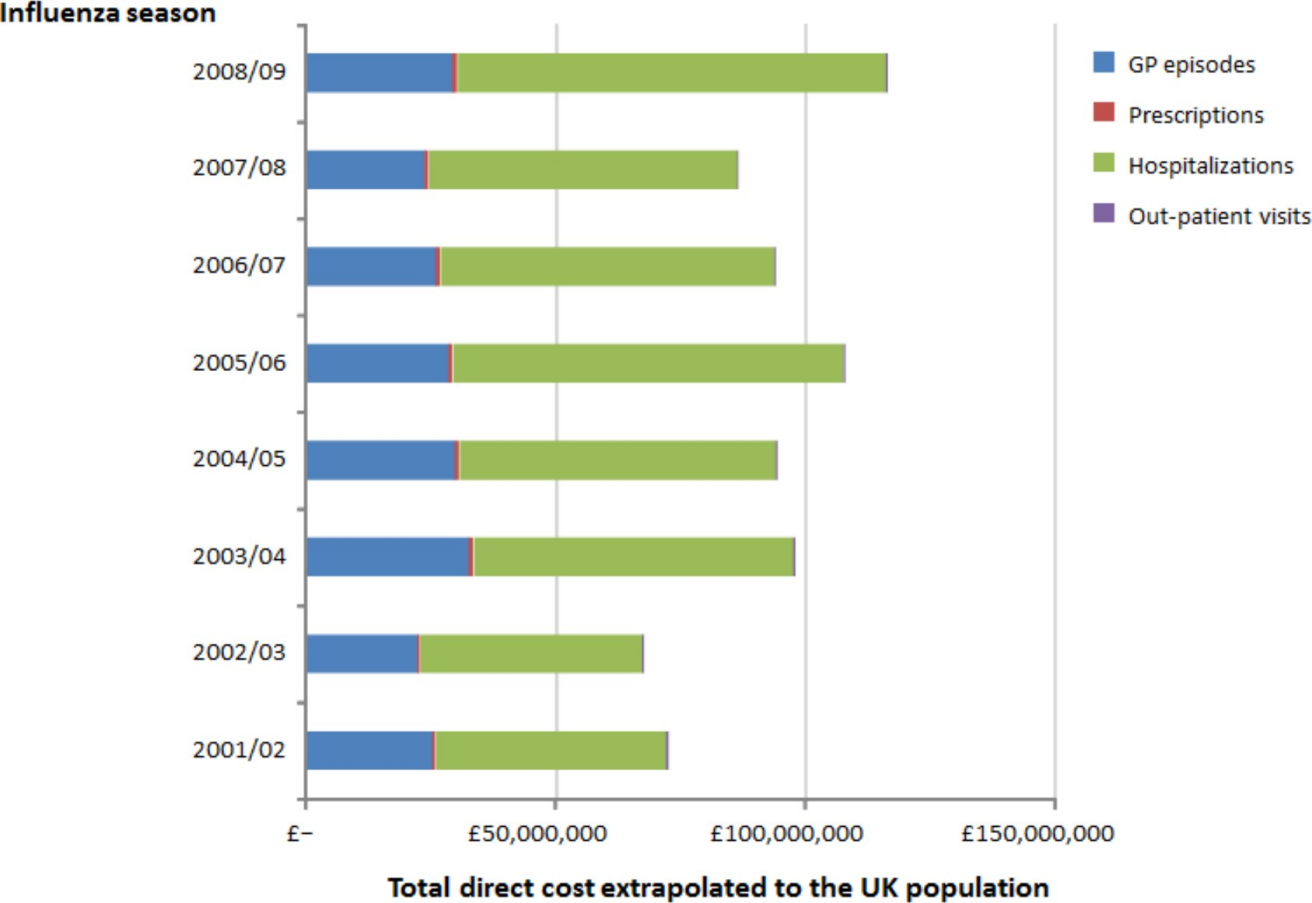
Resource use for acute respiratory illness in the CPRD/HES by influenza season extrapolated to the UK population for corresponding years.

A total of 156,193 patients had ≥1 GP episode of ARI during the study period (total population), with 82,204 patients receiving ≥1 GP prescription, at a mean of 2.5 (SD: 3.0) prescriptions per patient. The total cost of GP consultations and prescriptions during the study period was £6,506,137, equating to a yearly cost of £462,827 per 100,000 patients. The yearly cost of GP episodes of ARI ranged from £660,420 in 2007/08 to £904,644 in 2003/04. A summary of primary care resource use and costs by age group is shown in Table 1. The total yearly costs extrapolated to the UK population are shown in Fig 1. A summary of ambulatory care resource use and costs is shown in S2 Data.

**Table 1:** Primary care resource use and direct medical costs of acute respiratory illness by age and complication status in the CPRD population during influenza seasons from 2001 to 2009.

	Patients with ≥1 GP episode, n	Total number of GP episodes, n	Received ≥1 prescription, n	Mean (SD) number of prescriptions per patient	Total primary care cost
**Total**	156,193	178,304	82,204	2.5 (3.0)	£6,506,137
**<5 years**	2,845	3,294	1,251	1.6 (1.0)	£122,974
**5–18 years**	17,241	19,091	7,014	1.5 (1.0)	£702,780
**19–49 years**	78,906	88,404	34,994	1.7 (1.7)	£2,906,477
**50–64 years**	33,547	38,704	20,683	2.8 (3.0)	£1,416,237
**≥65 years**	23,654	28,811	18,262	4.3 (4.3)	£1,067,038

CPRD, Clinical Practice Research Datalink; SD, standard deviation; GP, general practitioner.

**Fig 2:**
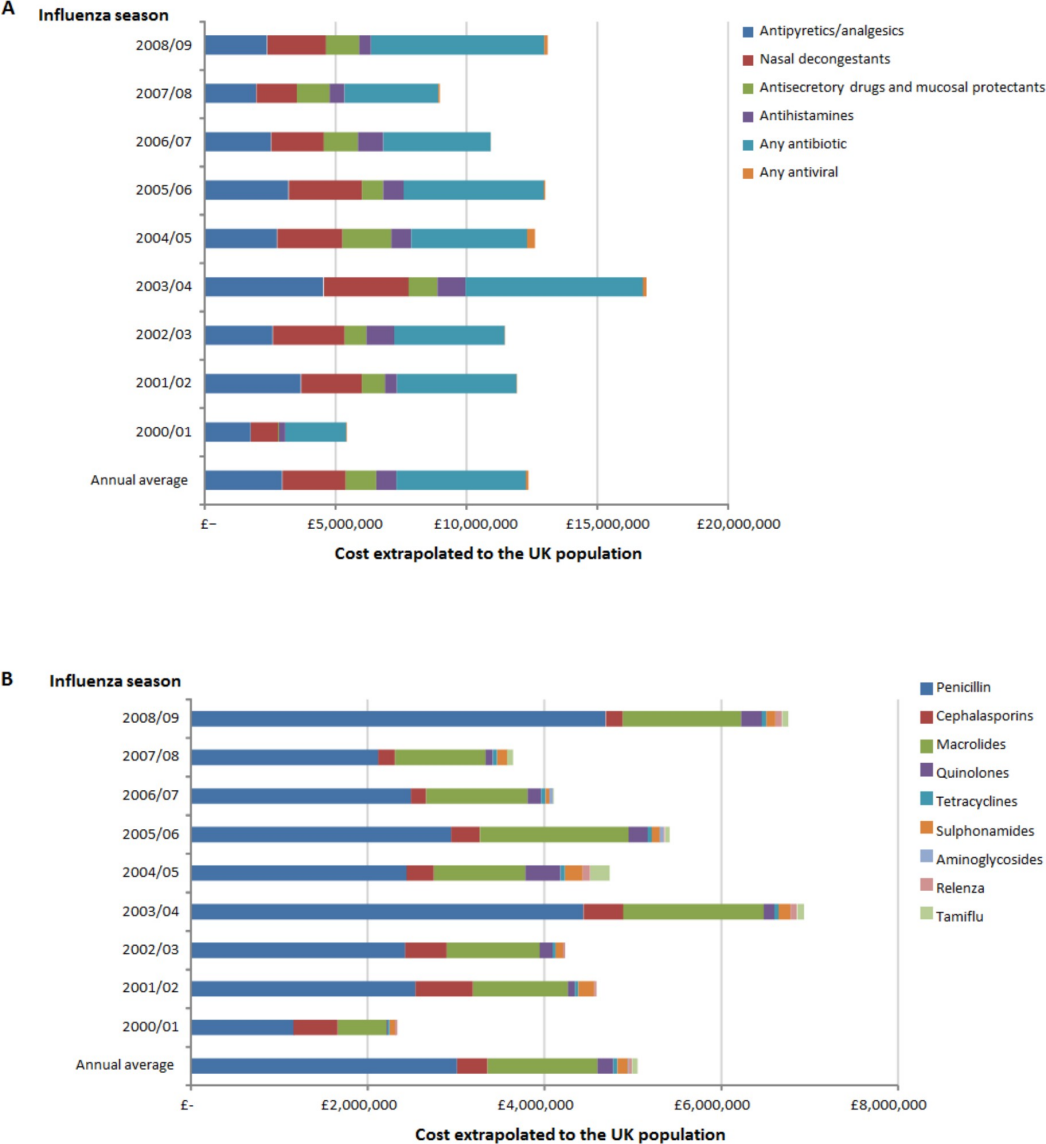
Prescriptions by medication class (A) and antibiotic and antiviral use (B) for acute respiratory illness in the CPRD by influenza season extrapolated to the UK population for corresponding years.CPRD, Clinical Practice Research Datalink.

**Fig 3:**
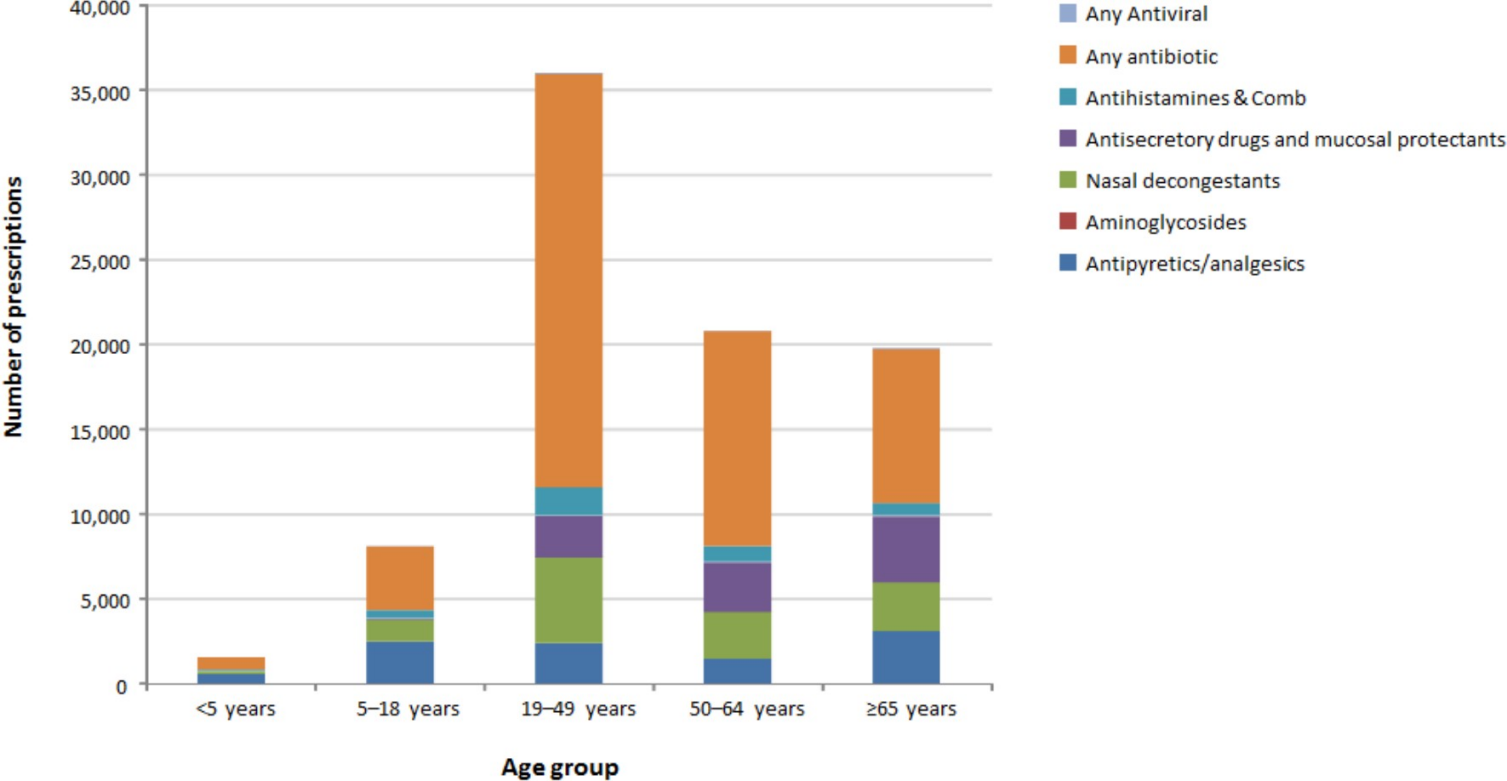
Number of prescriptions for acute respiratory illness in the CPRD by age group in influenza seasons from 2001 to 2009. CPRD, Clinical Practice Research Datalink.

The total yearly cost of prescribed medications in the study was £319,732, and extrapolated to the UK was estimated to be £104,367,150 (£11,596,350 per year), with 40% of this cost attributable to antibiotics (Fig 2). In the total population, the most commonly prescribed drug categories were antibiotics (41.0%), analgesics (31.1%), and anti-secretory drugs and mucosal protectants (11.6%) (Fig 2). Penicillin was the most commonly prescribed antibiotic (n = 30,979; 21.2%). Among the 82,204 patients who received a GP prescription, 25.5% were aged <5 years, 21.8% aged 5–18 years, 30.9% aged 19–49 years, 37.8% aged 50–64 years, and 38.5% aged ≥65 years (Fig 3).

### Secondary care

A total of 1,601 (1.0%) patients were admitted to hospital, with a total of 1,856 hospital admissions. In the total population, the total cost of hospital admissions was £13,801,634, equating to a yearly cost of £981,808 per 100,000 patients. A summary of secondary care resource use and direct medical costs is shown in Table 2.

**Table 2:** Secondary care resource use and direct medical costs of acute respiratory illness during influenza seasons from 2001 to 2009 in the CPRD/HES population and extrapolated to the UK population.

	All hospital admissions		Hospital admission via A&E		Hospital admission via GP referral		Hospital admission via other route^†^	
	CPRD/HES	Extrapolated	CPRD/HES	Extrapolated	CPRD/HES	Extrapolated	CPRD/HES	Extrapolated
**Had ≥ 1 hospital admission, n, (% of total population)**	1,601 (1.0%)	58,067	908 (0.6%)	32,932	532 (0.3%)	19,295	161 (0.1%)	5,839
**Absolute number of admissions**	1,856	67,315	1,012	36,704	619	22,451	225	8,161
**Mean number of unique admissions (SD)**	1.2 (0.46)	–	1.1 (0.38)		1.2 (0.47)	–	1.4 (0.65)	–
**Absolute length of stay, days**	20,119	729,696	12,173	441,502	6,052	219,500	1,894	68,693
**Mean length of stay (SD)**	11.3 (15.3)	–	12.5 (17.3)	–	9.9 (12.9)	–	9.0 (8.2)	–
**Absolute total cost**	£13,801,634	£500,571,386	£8,350,678	£302,870,693	£4,151,672	£150,576,968	£1,299,284	£47,123,724
**Mean (SD) total cost**	£14,892,758 (£7,729,737)	£540,145,356 (£280,349,788)	£8,564,710 (£4,094,866)	£310,633,419 (£148,516,672)	£4,335,630 (£2,212,704)	£157,248,940 (£80,252,549)	£1,391,620 (£588,677)	£50,472,658 (£21,350,723)
**Mean cost per hospitalisation (SD)**	£7,752 (£3,566)	–	£8,575 (£3,259)	–	£6,791 (£3,192)	–	£6,174 (£4,013)	–
**Mean cost per patient hospitalised (SD)**	£9,302 (£4,276)	–	£9,433 (£3,584)	–	£8,150 (£3,830)	–	£8,644 (£5,6180)	–

Standard deviation; CPRD/HES, Clinical Practice Research Datalink/ Hospital Event Statistics database; A&E, accident and emergency, GP, general practitioner. †Other routes of admission: elective, via bed bureau, via consultant outpatient clinic, via maternity unit, transfer from another hospital, unknown.

A total of 908 (56.7%) patients were admitted via A&E, 532 (33.2%) patients via a GP referral, and 161 (10.1%) patients via another route. The mean length of hospital stay in the total population was 11.3 (SD: 15.3) days, and the median length of hospital stay was 12.6 days. The cost of secondary care in those admitted via A&E was £9,433 (SD: £3,584) per patient, and in those admitted by GP referral was £8,150 (SD: £3,830) per patient. The average length of stay by year was lowest in 2002/03 (10.0 days) and highest in 2008/09 (12.9 days). The yearly cost of hospital admissions for ARI extrapolated to the UK population was £44,508,313 in 2002/03 and £85,803,090 in 2008/09 (Table 3).

**Table 3:** Resource use and direct medical costs of acute respiratory illness in hospitalised patients by influenza season in the CPRD/HES population and extrapolated to the UK population.

	Number of hospitalised patients	Average length of stay, days	Rate per 100,000 population	Total hospital cost	Extrapolated cost
2001/02	152	11.1	9.1	£1,273,892	£46,202,812
2002/03	162	9.9	9.7	£1,227,172	£44,508,313
2003/04	208	11.0	12.5	£1,763,805	£63,971,466
2004/05	189	11.6	11.4	£1,747,165	£63,367,945
2005/06	230	11.4	13.8	£2,154,609	£78,145,507
2006/07	214	10.6	12.9	£1,845,067	£66,918,743
2007/08	185	11.6	11.1	£1,703,719	£61,792,208
2008/09	214	12.8	12.9	£2,365,742	£85,803,090

CPRD/HES, Clinical Practice Research Datalink/ Hospital Event Statistics database.

### Total direct medical costs

The total mean direct medical cost in the total population was £21,343,445 (SD: £10,441,364), at a mean cost of £136.65 (SD: £66.85) per case (Table 4). By age subgroup, the cost per case was £92.78 (<5 years), £57.19 (5–18 years), £61.21 (19–49 years), £116.32 (50–64 years), £477.72 (≥65 years). Extrapolated to the UK population, the total estimated cost of ARI in the study period was £774.1 million (SD: £378.7 million) or £86,011,699 per year.

**Table 4:** Total direct medical costs of acute respiratory illness during influenza seasons from 2001 to 2009 in the CPRD/HES population and extrapolated to the UK.

	Total cost (SD)	Total cost per case (SD)	Extrapolated (SD)
**Total population**	£21,343,445 (£10,441,364)	£136.65 (£66.85)	£774,105,286 (£378,697,772)
**<5 years**	£263,949 (£63,813)	£92.78 (£22.43)	£9,573,164 (£2,314,433)
**5–18 years**	£985,948 (£447,395)	£57.19 (£25.95)	£35,759,342 (£16,226,566)
**19–49 years**	£4,829,902 (£1,964,789)	£61.21 (£24.90)	£175,175,688 (£71,260,921)
**50–64 years**	£3,902,205 (£1,947,666)	£116.32 (£58.06)	£141,529,051 (£70,639,887)
**≥65 years**	£11,299,991 (£5,542,153)	£477.72 (£234.30)	£409,839,310 (£201,008,315)

CPRD/HES, Clinical Practice Research Datalink/ Hospital Event Statistics database; SD, standard deviation.

The highest total costs were incurred in high risk patients with CHD at £4,591,080 (SD: £2,603,295), and the highest cost per case was in patients with renal disease at £13,773 (SD: £6,656.23) per case (Table 5). Extrapolated to the UK population, the total cost in the study period of patients with ARI and CHD was £166,513,855 (SD: £94,418,892), at a cost per case of £18,750 (SD: £10,632) (Table 6).

**Table 5:** Secondary care resource use for acute respiratory illness in high-risk patients extrapolated to the UK population for influenza seasons from 2001 to 2009.

	Patients with ≥1 admission to hospital	Mean (SD) number of admissions	Total number of admissions	Mean (SD) length of stay, days	Total hospital bed-days	Bed-days per 100,000 high-risk population
Total	26,295	1.2 (0.53)	32,098	12.8 (17.1)	383,835	49,182
Respiratory	9,684	1.3 (0.64)	12,803	10.8 (12.0)	132,454	16,972
CNS	1,414	1.3 (0.58)	1,886	19.2 (37.2)	31,336	4,015
Diabetes	8,197	1.2 (0.50)	9,684	13.2 (17.6)	119,869	15,359
CHD	11,824	1.2 (0.52)	14,326	15.4 (20.4)	202,671	25,969
Liver disease	290	1.4 (0.52)	399	12.1 (10.2)	5,186	665
Renal disease	3,192	1.4 (0.70)	4,389	15.1 (14.6)	58,792	7,533
Immunocompromised	544	1.3 (0.62)	725	10.1 (7.5)	6,420	823

CPRD/HES, Clinical Practice Research Datalink/ Hospital Event Statistics database; SD, standard deviation; CHD, chronic heart disease, CNS, Central nervous system.

**Table 6:** Total direct medical cost of acute respiratory illness in high-risk patients during influenza seasons from 2001 to 2009 in the CPRD/HES population and extrapolated to the UK population.

	CPRD/HES		Extrapolated	
	Total cost (SD)	Cost per case (SD)	Total cost (SD)	Cost per case (SD)
**Respiratory**	£2,962,011 (£1,647,509)	£441.89 (£245.79)	£107,429,160 (£59,753,495)	£16,027 (£8,915)
**Central nervous system**	£722,220 (£604,167)	£676.87 (£566.23)	£26,194,193 (£21,912,530)	£24,549 (£20,537)
**Diabetes**	£2,821,988 (£1,549,777)	£407.57 (£223.83)	£102,350,667 (£56,208,853)	£14,782 (£8,118)
**Chronic heart disease**	£4,591,080 (£2,603,295)	£516.96 (£293.13)	£166,513,855 (£94,418,892)	£18,750 (£10,632)
**Liver disease**	£101,284 (£32,887)	£565.83 (£183.72)	£3,673,469 (£1,192,778)	£20,522 (£6,663)
**Renal disease**	£1,280,888 (£619,029)	£13,772.99 (£6,656.23)	£46,456,520 (£22,451,559)	£499,532 (£241,415)
**Immunocompromised**	£144,767 (£51,827)	£689.37 (£246.80)	£5,250,554 (£1,879,713)	£25,003 (£8,951)

CPRD/HES, Clinical Practice Research Datalink/ Hospital Event Statistics database; SD, standard deviation.

## Discussion

This retrospective, cross-sectional study over 9 influenza seasons between 2001 and 2009, identified 156,193 patients with ≥1 GP episode of ARI in the CPRD, of which 1,601 (1.0%) patients were subsequently admitted to hospital. The cost of GP consultations and prescriptions was £462,827 per 100,000 patients per year and the cost of hospital admissions was £981,808 per 100,000 patients per year. The total cost of prescribed medications extrapolated to the UK was estimated at £11,596,350 per year, with £4.6m (40%) attributable to antibiotics, and the direct medical cost of ARI in the UK was estimated to be about £86 million per year and £137 per case. The study shows the substantial costs of ARI in otherwise healthy people presenting to primary care, with the greatest direct medical cost component due to hospital admissions, largely among patients at high-risk of severe influenza outcomes.

Of the 156,193 patients with an ARI GP episode, 82,204 (52.6%) patients received a mean of 2.5 prescriptions. The cost in primary care in the total population study ranged from £666,420 in 2007/08 to £904,644 in 2003/04. Antibiotics accounted for about 40% of prescribed medications, whereas antiviral use was relatively low. The most commonly prescribed antibiotic was penicillin at an average cost of £3,015,314 per year, followed by macrolides at an average cost of £1,244,624 per year, whereas the average cost of *Tamiflu* was £61,539 per year, and of *Relenza* was £36,003 per year.

The pattern of antibiotic prescribing in our study was broadly consistent with previous reports in the UK including a CPRD study from 1991 to 1996, which showed that 59.4% of patients with influenza-related primary care episodes received medication on prescription, of which 45% were for antibiotics.[19] In a qualitative study of antibiotic prescribing in primary care for cough and suspected lower respiratory tract infection (LRTI) in 13 European countries including the UK (Cardiff and Southampton networks), 53% of patients received an antibiotic, although there were wide regional differences; amoxicillin was the most commonly prescribed antibiotic, accounting for 29% of prescriptions, ranging from 3% in Tromso to 83% in Southampton.[20] In a further study of primary care physicians in nine European countries, the clinical factors most often cited as guiding antibiotic prescribing were auscultation findings, fever, discoloured sputum, and breathlessness.[21]

Despite the wide use of antibiotics, it is likely that the majority of patients who are prescribed them for respiratory illness in primary care have viral illness. For example, in a study of children aged <12 years over four winters in the UK, among about 400 cases of cough and fever for which GPs considered prescribing an antibiotic, 77% had a laboratory-confirmed viral infection.[22] Furthermore, in a secondary care study conducted over three winters at a hospital in the UK, of 780 patients admitted for an acute respiratory illness, 76% received an antibiotic, and although bacterial infection was frequently confirmed in patients with COPD and pneumonia, 21% of antibiotic use was in patients with no detectable bacterial infection.[23] Antibiotics prescribed in primary care may improve serious outcomes in high-risk patients with LRTI, although antibiotics are more often reported to have no benefit on symptom severity or time to recovery in adults presenting to primary care with acute respiratory illnesses caused by viral infection.[24, 25]

Resource use among patients with a GP episode of ARI and linked HES data was substantial even though hospitalised patients represented only 1.0% of the population. A total of 1,601 patients were hospitalised during the study period, the majority of which were admitted once (87%); 10.6% were admitted twice, and two patients were admitted 5 times. The mean number of unique admissions per patient was 1.2 to 1.4 depending upon risk status. The highest rate of hospitalisation was in patients aged ≥65 years (3.7%) or <5 years (2.1%), whereas the rate of hospital admissions in the other age groups was <1%. In the previous analysis of the population stratified by influenza vaccination status, although the majority of GP episodes of ARI were in unvaccinated patients, a higher proportion of the vaccinated group (2.3%) were hospitalised than the unvaccinated group (0.5%). Hospitalised patients tended to be older with clinical risk factors associated with serious influenza outcomes, and as such, were likely to have been eligible for publically-funded vaccination.[4] In the total population, among high-risk patients, we observed the highest hospital admission rates in those aged ≥65 years at a rate of 3,474 per 100,000 population.

The rates of hospitalisation observed in our study were substantially lower than those previously reported in England and Wales based on HES data,[26] which is expected given that our study includes hospitalised patients who first presented to primary care, rather than all hospital admissions in the HES. In a further study using data from in the UK from the Royal College of General Practitioners’ database and the HES between 2000 and 2008, the highest rate of influenza-related hospital admissions in patients without clinical risk factors was in infants aged <6 months at 84.5 per 1000, followed by children aged 6 months to <4 years at 33.6 per 1000, and adults aged ≥65 years at 12.1 per 1000.[12] The incidence of hospitalisation was relatively low in healthy adults, and in all groups, the presence of clinical risk factors for influenza infection and related complications increased the risk of hospitalisation.[12]

The vast majority of patients received only primary care at a cost of £6,506,137 over the study period, whereas the cost of secondary care among the 1.0% of patients who were hospitalised was £13,801,634 over the study period. The mean cost of hospital admissions in the total population was £9,302 per patient, in those admitted via A&E was £9,433 per patient, and in those admitted by GP referral was £8,150 per patient. The mean cost per hospitalisation was highest in patients with heart disease (£10,564), CNS disease (£13,171) and renal disease (£10,359). In our previous analysis, in patients aged ≥65 years, influenza vaccination coverage was high, peaking at 84.5% in 2005/06; despite this, the analysis of the total population showed that the total direct medical cost of ARI was £477.72 per patient aged ≥65 years compared with £61.21 per patient aged 19–49 years.

The cost-effectiveness of seasonal influenza vaccination campaigns is difficult to measure because the clinical benefits of vaccination vary according to epidemic intensity, vaccine match with circulating strains, and risk-factors of the population; moreover, measuring absolute vaccine efficacy in elderly populations is not possible as the use of a placebo control is not ethical. To guide public health decision-making, and to assess the real world burden of influenza and the effectiveness of vaccination, many countries have established sentinel surveillance networks. Recent sentinel swabbing surveillance schemes in seasons between 2010 and 2014 in Europe and North America that assessed vaccine effectiveness for the prevention of laboratory-confirmed influenza, report adjusted effectiveness estimates from 23% to 75% in adults, with most studies showing moderate effectiveness of about 45% to 55% in the general population, and lower effectiveness in years of vaccine mismatch.[27–36] In a sentinel influenza surveillance study in primary care in the UK, over three winters from 2004 to 2007, the yearly adjusted vaccine effectiveness estimate was 55% to 67% in the general population, and 46% in people aged ≥65 years.[37]

In most countries the aim of vaccinating elderly populations is to reduce hospitalisation and death, but the wide-variation in vaccine effectiveness reported in this high-risk population means that the benefit of vaccination against serious outcomes in elderly people remains a matter for debate. Estimates of vaccine effectiveness in elderly populations hospitalised with laboratory-confirmed influenza range from 33% to 86% in populations in Europe, North America, and Australia.[27, 34, 37–49] Despite variations in the reported vaccine effectiveness, cost-effectiveness models in various regions and settings generally show that vaccinating older people is cost-effective, but given the clinical challenge of assessing serious outcomes in high-risk patients, the benefits of vaccination are likely to have been underestimated. For example, in Canada, a hospital-based sentential surveillance network was established in 2009 and has since been prospectively monitoring seasonal influenza vaccine effectiveness in the prevention of laboratory-confirmed influenza-hospitalisation over consecutive seasons. Based on influenza-related hospitalisations in the 2011/2012 season, in people aged ≥65 years, the adjusted vaccine effectiveness estimate was 58%, but after removing baseline frailty from the model, the vaccine effectiveness was 43%.[50] This is the first sentinel study where vulnerability to illness (frailty) was assessed in detail, and the effect of this risk-factor on serious influenza-outcomes was substantial.[50]

Using READ codes to identify patients with clinical influenza syndromes including a range of proxy diagnoses was the main limitation of our study. Although clinical diagnoses probably vary in their sensitivity and specificity for ARI depending upon the patient age and co-morbidities, logistic regression analysis of CPRD/HES data and weekly influenza activity from sentinel surveillance showed a strong correlation between circulating influenza viruses and clinical episodes in the study (GP episodes of ARI, hospitalisations, and deaths).[13] However, the resource use and costs are based on all ARI episodes and not the influenza-attributable rate. Medications prescribed in hospital were not captured, as these are not collected as part of the HES data collection. The incidence of GP referrals to ambulatory out-patient appointments was also not assessed, due to the scantiness of the data collection around this data point. A strength of the study was the ability to follow patient journeys from the index episode in primary care, through to hospital admission and discharge, yet a limitation of this approach was that the study did not identify all influenza-related hospital admissions, such as those admitted via A&E without a previous GP visit. A further strength was that the study period of 2001 to 2009 was selected to provide estimates over multiple consecutive seasons, but not including contemporary seasons during which episodes of ARI were unusually high due to, for example, the circulation of A(H1N1)pdm09 (2009/10 season) and the emergence of an A(H3N2) strain that was drifted from the vaccine strain (2014/15 season).[49]

In summary, this retrospective, cross-sectional study using the CPRD and linked HES databases in the UK showed that the cost of ARI in primary care was £462,827 per 100,000 population per year and the cost of linked hospital admissions was £981,808 per 100,000 population per year. Extrapolating to the UK population, for 9 influenza seasons from 2001 to 2009, the direct medical cost of ARI was estimated to be £137 per case, equating to £86 million per year.

## Supporting information

S1 Data (DOCX)

S2 Data (DOCX)
