# Supplementary material for: Resource use and direct medical costs of acute respiratory illness in the UK based on linked primary and secondary care records from 2001 to 2009
Source: PLoS One. 2020 Aug 6;15(8):e0236472. doi: 10.1371/journal.pone.0236472 (PMC7410242; doi:10.1371/journal.pone.0236472)
Supplement: S1 Data — (DOCX) [file pone.0236472.s001.docx]

**Methods**

*Case definitions and data extraction*

Data on patients who had at least one GP episode with a READ code identifying acute respiratory illnesses were extracted from the GPRD dataset for the period between 21st January 2001 and 31st March 2009 inclusive (NCT Number: 01521416). Cases of acute respiratory illness (ARI) were identified using a broad range of READ codes describing influenza, influenza-like illness, or acute upper or lower respiratory tract infection with influenza. READ codes are shown **Appendix I**. If two ARI GP episodes were recorded for a patient on the same day, only one was included.

The HES database was assessed for patients with an ARIGP episode (primary care) and a subsequent linked hospital admission(s). Data from patients linked to the HES was extracted for 14 days before and after the index ARIepisode. Hospitalized patients were followed for 28 days after discharge. For patients who were admitted to hospital and then transferred to another consultant generating a new episode, only codes for the first admission were included. Subsequent hospitalisations within a 30-day window were considered as separate admissions. Mortality data were identified from the CPRD/HES in patients with death recorded by the GP or at hospital discharge. If the cause of death was not identifiable within the database, it was assumed that death was influenza-related if it occurred within 28 days of the index ARIepisode, while hospitalized, or within 28 days of discharge from hospital.

Complications were identified using International Classification of Diseases (ICD)-10 codes and were defined as the incidence of a new event or an exacerbation of a chronic condition (**Appendix II**). ARIGP episodes defined as ‘complicated’ had to have a diagnosis for a complication additional to the index ARI episode (**Appendix III**). Patients could have ≥1 complication, and complications were attributed to ARIif they occurred within 30 days before or after the onset of the index ARIepisode. (ICD)-10 codes included: acute and chronic otitis media; cardiovascular; central nervous system; cerebrovascular; gastrointestinal bleeding; renal; chronic obstructive pulmonary disease (COPD); upper respiratory tract infection; lower respiratory tract infection; other respiratory complications; and other complications. ‘Other respiratory complications’ included asthma, chronic respiratory disease (CRD), emphysema, laryngotracheobronchitis, pharyngitis, pneumonia, pneumothorax, sinusitis and tonsillitis. ‘Other complications’ included febrile convulsion, septicaemia, sepsis, meningitis, encephalitis, myelitis and encephalomyelitis, myositis and myalgia.

Patients were categorised as low- or high-risk for influenza infection and related complications using criteria from the National Institute for Health and Care Excellence (NICE) and the UK Department of Health (**Appendix IV**) [1, 2]. Patients were defined as high-risk if they had ≥1 co-morbid factor: chronic respiratory disease, including asthma and COPD, chronic heart disease, chronic renal disease, chronic liver disease, chronic neurological conditions, diabetes mellitus, aged ≥65 years, and immunosuppressed, including transplanted patients. The Department of Health criteria for high-risk patients are the same as NICE criteria with the addition of patients who are pregnant. READ codes for the high-risk co-morbid factors must have been entered at least 1 year before the index ARIepisode. Patients not fulfilling any high-risk definition were classified as low-risk.

The data extracted included: age, sex, prior co-morbidities, risk status, events (clinical, medications, hospital referrals, and immunization), lifestyle indicators, body mass index (BMI) complications, clinical information about diagnoses and operations, date of hospital admission, discharge, and admission route. Patients were stratified by age (<5 years, 5–≤18 years, 19–≤49 years, 50–≤64 years, ≥65 years), the presence of complications (complicated or uncomplicated), and risk status (high- or low-risk).

*Influenza peak season*

The study was conducted from 2001 to 2009 covering 9 influenza seasons, up to the outbreak of pandemic A(H1N1)pdm09 in the UK on the 27th of April 2009. GP episodes with any relevant READ code recorded during periods of influenza peak season each year were included in the study. The start and end of peak influenza season in each year was based on laboratory-confirmed reports of influenza and other respiratory infections circulating in the UK from the Health Protection Agency (HPA) for the period November 2002 to December 2008 [3]. HPA laboratory reports for influenza A and B and other respiratory infections (adenovirus, coronavirus, mycoplasma pneumoniae, parainfluenza, rhinovirus, and respiratory syncytial virus) included the number of laboratory-confirmed specimens by week, with demographics such as age and sex. Temporal trends in laboratory-confirmed reports of agents that could potentially cause influenza/ARI/influenza like illness in the weekly national HPA laboratory reports were compared with the number of ARIevents reported over the same time period in the study. For clinical cases of ARI identified in the database, each was confirmed as occurring within the peak season according to the HPA surveillance data.

*Outcome measures*

Burden of ARI

The outcome measures were the incidence of: ARI GP episodes, linked hospital admissions, death, complications, and high-risk conditions: respiratory, central nervous system, diabetes, chronic heart disease, pregnant, liver disease, renal disease, and immunocompromised. The outcomes were assessed in the total population including all patients with ≥1 ARI GP episode and the hospitalized population including patients with an index ARI episode and a subsequent hospital admission. Subgroup analyses were performed by age (<5 years; 5–≤18 years; 19–≤49 years; 50–≤65 years, ≥65 years), complication status (complicated cases and uncomplicated cases), and clinical risk group (low-risk and high-risk). Mortality was classified as being without hospital admission, in hospital, or within 28 days of discharge from hospital.

Resource use and costs

Resource use was classified as ‘primary care’ if it was associated with a GP consultation including prescriptions for medications. Medications were assessed by drug category: antipyretics/analgesics, antibiotics, amantadine, aminoglycosides, nasal decongestants, antisecretory drugs and mucosal protectants, and antihistamines. Antibiotics were assessed by class. Resource use was classified as hospitalization if it was associated with patients who were admitted to hospital or who attended a hospital out-patient appointment. Hospital resource use outcomes were: the incidence of hospital admission, mean number of unique admissions, mean length of stay per hospitalization, mean number of out-patient appointments, and route of hospital admission (A&E, GP referral, or other). Medications prescribed in hospital were not captured. The incidence of GP referrals to ambulatory out-patient appointments was also assessed.

The cost perspective was that of the UK National Health Service (NHS). GP consultation costs and out-patient visit costs are from the Personal Social Services Research Unit (PSSRU) database [4]. Unit costs for hospitalizations were derived from the NHS secondary care tariff for healthcare-related groups (HRG). All HRG codes for a complication relevant to the study were grouped together to calculate a mean unit cost for that complication. The analysis was based on 2011 costs expressed in £ (pounds sterling).

| **Summary of source costs used to calculate the direct costs of acute respiratory illness in the UK during influenza seasons from 2001 to 2009** | | | |
| --- | --- | --- | --- |
| **Resource** | **Cost** | **Source** | |
| GP consultation | £36.00 | Unit costs of health and social care 2011. Available online at http://www.pssru.ac.uk/archive/pdf/uc/uc2011/uc2011.pdf. Accessed on 18th May 2012. | Table 10.8 |
| Out-patient care | £147.00 | Unit costs of health and social care 2011.  Available online at http://www.pssru.ac.uk/archive/pdf/uc/uc2011/uc2011.pdf. Accessed on 18th May 2012. | Table 7.1 |
| Hospital admission | £686.00 | Unit costs of health and social care 2011.  Available online at: http://www.pssru.ac.uk/archive/pdf/uc/uc2011/uc2011.pdf. Accessed on 18th May 2012. | Table 7.1 |

*Data analysis*

No comparisons were made between study groups so there was no target sample size. All eligible patients within the database were used in the analysis. Raw data were cleaned before analysis. It was assumed that a patient could experience only one ARI GP episode and one hospitalization on one day. Data were extracted using MySQL, and analyses were conducted in *Microsoft Access*, *Microsoft Excel* and *Statistical Package for the Social Sciences* (SPSS).

The burden of ARI, resource use, and costs were analysed descriptively in the total population and by age, complication status, and risk status. The burden and resource use data were reported as the number of patients and as a percentage of the total population. The data were also provided as the incidence of the outcome per 100,000 population in the GPRD. The data was extrapolated to the UK population using Office National Statistics mid-year estimates for 2001–2009 [5]: calculated from the GPRD/HES data extract, the yearly average number of patients in the GPRD/HES database in 2000–2009 was 1,662,953, and the average UK population in 2001–2009 was 60,313,633, giving a multiplying factor of 36.3 which was used to extrapolate the study data. The burden, resource use, and costs were assessed over the study period (9 seasons) and for each season. Summary statistics were provided as mean values and standard deviation (SD) for continuous variables and frequency distributions for categorical variables.

**References**

1. National Institute for Health and Clinical Excellence. Amantadine, oseltamivir and zanamivir for the treatment of influenza. Technology Appraisal 168. February 2009.

2. United Kingdom National Health Service. Who should have the flu jab? <http://www.nhs.uk/Conditions/vaccinations/Pages/flu-influenza-vaccine.aspx>.

3. Health Protection Agency. Weekly influenza reports. 2000/2001 to 2008/2009.

4. Personal Social Services Research Unit. 2011. <http://wwwpssruacuk/archive/pdf/uc/uc2011/uc2011pdf>

5. Office for National Statistics (ONS). Population Estimates for UK, England and Wales, Scotland and Northern Ireland. Mid-2001 to Mid-2010.

**Appendix I**

# READ codes for patient selection in GPRD

Below are the proposed READ codes required to identify eligible patients within the GPRD database.

## Influenza

| **Read Code** | **Description** |
| --- | --- |
| **Those we believe should be included** | |
| A08..11 | Gastric flu |
| F030800 | Encephalitis due to influenza-specific virus not identified |
| F030A00 | Encephalitis due to influenza-virus identified |
| H2...00 | Pneumonia and influenza |
| H27..00 | Influenza |
| H270.00 | Influenza with pneumonia |
| H270.11 | Chest infection - influenza with pneumonia |
| H270000 | Influenza with bronchopneumonia |
| H270100 | Influenza with pneumonia, influenza virus identified |
| H270z00 | Influenza with pneumonia NOS |
| H271.00 | Influenza with other respiratory manifestation |
| H271000 | Influenza with laryngitis |
| H271100 | Influenza with pharyngitis |
| H271z00 | Influenza with respiratory manifestations NOS |
| H27y.00 | Influenza with other manifestations |
| H27y000 | Influenza with encephalopathy |
| H27y100 | Influenza with gastrointestinal tract involvement |
| H27yz00 | Influenza with other manifestations NOS |
| H27z.00 | Influenza NOS |
| Hyu0400 | [X]Flu+oth respiratory manifestations,'flu virus identified |
| Hyu0500 | [X]Influenza+other manifestations,influenza virus identified |
| Hyu0600 | [X]Influenza+oth respiratory manifestatns,virus not identifd |
| Hyu0700 | [X]Influenza+other manifestations, virus not identified |
| 16L..00 | Influenza-like symptoms |
| H27z.11 | Flu like illness |
| H27z.12 | Influenza like illness |
| H2y..00 | Other specified pneumonia or influenza |
| H2z..00 | Pneumonia or influenza NOS |
| 65E..00 | Influenza vaccination |
| 68NE.00 | No consent - influenza imm. |
| 68NN.00 | Influenza imm.advised in surg. |
| 68NN.11 | Influenza immunization advised |
| 68NO.00 | Influenza imm.advised at home |
| 68NV.00 | Influenza vacc consent given |
| 8I2F.00 | Influenza vaccination contraindicated |
| 8I6D.00 | Influenza vaccination not indicated |
| 9k7..00 | Influenza immunisation - enhanced services administration |
| 9N4q.00 | Did not attend flu vaccination appointment |
| 9OX..00 | Influenza vacc. administration. |
| 9OX..11 | Flu vaccination administration |
| 9OX1.00 | Has 'flu vaccination at home |
| 9OX2.00 | Has'flu vaccination at surgery |
| 9OX3.00 | Has 'flu vaccination at hosp. |
| 9OX4.00 | Needs influenza immunisation |
| 9OX5.00 | Influenza vaccination declined |
| 9OX6.00 | Influenza vaccination invitation letter sent |
| 9OX7.00 | Influenza vaccination telephone invite |
| 9OX8.00 | Has influenza vaccination at work |
| 9OX9.00 | Influenza vaccination invitation first letter sent |
| 9OXA.00 | Influenza vaccination invitation second letter sent |
| 9OXB.00 | Influenza vaccination invitation third letter sent |
| 9OXZ.00 | Influenza vacc.administrat.NOS |
| ZV04800 | [V]Influenza vaccination |
| ZV04811 | [V]Flu - influenza vaccination |
| ZV14F00 | [V]Personal history of influenza vaccine allergy |

## Acute otitis media

| **Read Code** | **Description** |
| --- | --- |
| **Those we believe should be included** | |
| F510.00 | Acute non suppurative otitis media |
| F510000 | Acute otitis media with effusion |
| F510011 | Acute secretory otitis media |
| F510100 | Acute serous otitis media |
| F510200 | Acute mucoid otitis media |
| F510300 | Acute sanguinous otitis media |
| F510z00 | Acute nonsuppurative otitis media NOS |
| F520.00 | Acute suppurative otitis media |
| F520000 | Acute suppurative otitis media tympanic membrane intact |
| F520100 | Acute suppurative otitis media tympanic membrane ruptured |
| F520300 | Acute suppurative otitis media due to disease EC |
| F520z00 | Acute suppurative otitis media NOS |
| F525.00 | Recurrent acute otitis media |
| F526.00 | Acute left otitis media |
| F527.00 | Acute right otitis media |
| F528.00 | Acute bilateral otitis media |
| FyuP000 | [X]Other acute nonsuppurative otitis media |
| F51..00 | Nonsuppurative otitis media + eustachian tube disorders |
| F514.00 | Unspecified nonsuppurative otitis media |
| F514100 | Serous otitis media NOS |
| F514200 | Catarrhal otitis media NOS |
| F514300 | Mucoid otitis media NOS |
| F514z00 | Nonsuppurative otitis media NOS |
| F52..00 | Suppurative and unspecified otitis media |
| F524.00 | Purulent otitis media NOS |
| F524000 | Bilateral suppurative otitis media |
| F52z.00 | Otitis media NOS |
| FyuP400 | [X]Otitis media in viral diseases classified elsewhere |

## Bronchitis

| **Read Code** | **Description** |
| --- | --- |
| **Those we believe should be included** | |
| H06..00 | Acute bronchitis and bronchiolitis |
| H060.00 | Acute bronchitis |
| H060.11 | Acute wheezy bronchitis |
| H060000 | Acute fibrinous bronchitis |
| H060100 | Acute membranous bronchitis |
| H060200 | Acute pseudomembranous bronchitis |
| H060300 | Acute purulent bronchitis |
| H060400 | Acute croupous bronchitis |
| H060500 | Acute tracheobronchitis |
| H060A00 | Acute bronchitis due to mycoplasma pneumoniae |
| H060v00 | Subacute bronchitis unspecified |
| H060w00 | Acute viral bronchitis unspecified |
| H060x00 | Acute bacterial bronchitis unspecified |
| H060z00 | Acute bronchitis NOS |
| H06z.00 | Acute bronchitis or bronchiolitis NOS |
| H30..00 | Bronchitis unspecified |
| H30..11 | Chest infection - unspecified bronchitis |
| H30..12 | Recurrent wheezy bronchitis |
| H300.00 | Tracheobronchitis NOS |
| H301.00 | Laryngotracheobronchitis |
| H302.00 | Wheezy bronchitis |
| H30z.00 | Bronchitis NOS |
| Hyu1000 | [X]Acute bronchitis due to other specified organisms |
| H060600 | Acute pneumococcal bronchitis |
| H060700 | Acute streptococcal bronchitis |
| H060800 | Acute haemophilus influenzae bronchitis |
| H060900 | Acute neisseria catarrhalis bronchitis |
| H312100 | Emphysematous bronchitis |
| H060B00 | Acute bronchitis due to coxsackievirus |
| H060C00 | Acute bronchitis due to parainfluenza virus |
| H060D00 | Acute bronchitis due to respiratory syncytial virus |
| H060E00 | Acute bronchitis due to rhinovirus |
| H060F00 | Acute bronchitis due to echovirus |

## Laryngitis

| **Read Code** | **Description** |
| --- | --- |
| **Those we believe should be included** | |
| H04..00 | Acute laryngitis and tracheitis |
| H040.00 | Acute laryngitis |
| H040w00 | Acute viral laryngitis unspecified |
| H040z00 | Acute laryngitis NOS |
| H271000 | Influenza with laryngitis |
| H040000 | Acute oedematous laryngitis |
| H040100 | Acute ulcerative laryngitis |
| H040200 | Acute catarrhal laryngitis |
| H040300 | Acute phlegmonous laryngitis |
| H040400 | Acute haemophilus influenzae laryngitis |
| H040500 | Acute pneumococcal laryngitis |
| H040600 | Acute suppurative laryngitis |
| H043200 | Acute obstructive laryngitis |
| H04z.00 | Acute laryngitis and tracheitis NOS |
| H055.00 | Pharyngolaryngitis |
| H160400 | Laryngitis sicca |

## Laryngotracheobronchitis

| **Read Code** | **Description** |
| --- | --- |
| **Those we believe should be included** | |
| H301.00 | Laryngotracheobronchitis |
| H043211 | Croup |
| H044.00 | Croup |
| H060400 | Acute croupous bronchitis |

## Nasopharyingitis

| **Read Code** | **Description** |
| --- | --- |
| **Those we believe should be included** | |
| H00..00 | Acute nasopharyngitis |

## Pharyngitis

| **Read Code** | **Description** |
| --- | --- |
| **Those we believe should be included** | |
| H00..00 | Acute nasopharyngitis |
| H02..00 | Acute pharyngitis |
| H02..13 | Throat infection - pharyngitis |
| H024.00 | Acute viral pharyngitis |
| H02z.00 | Acute pharyngitis NOS |
| H050.00 | Acute laryngopharyngitis |
| H053.00 | Tracheopharyngitis |
| H271100 | Influenza with pharyngitis |
| Hyu0100 | [X]Acute pharyngitis due to other specified organisms |
| 2DC2.00 | O/E - granular pharyngitis |
| A340200 | Streptococcal pharyngitis |
| AA12.00 | Vincent's pharyngitis |
| AA25.11 | Rhinopharyngitis mutilans |
| H020.00 | Acute gangrenous pharyngitis |
| H021.00 | Acute phlegmonous pharyngitis |
| H022.00 | Acute ulcerative pharyngitis |
| H023.00 | Acute bacterial pharyngitis |
| H023000 | Acute pneumococcal pharyngitis |
| H023100 | Acute staphylococcal pharyngitis |
| H023z00 | Acute bacterial pharyngitis NOS |
| H121100 | Atrophic pharyngitis |
| H121200 | Granular pharyngitis |
| H121300 | Hypertrophic pharyngitis |
| H121400 | Pharyngitis keratosa |
| H121500 | Pharyngitis sicca |

## Pneumonia and secondary bacterial pneumonia

| **Read Code** | **Description** |
| --- | --- |
| **Those we believe should be included** | |
| A116.00 | Tuberculous pneumonia |
| A380300 | Septicaemia due to streptococcus pneumoniae |
| A3BXA00 | Mycoplasma pneumoniae [PPLO] cause/dis classifd/oth chaptr |
| A3BXB00 | Klebsiella pneumoniae/cause/disease classifd/oth chapters |
| A3By400 | Pleuropneumonia-like organism (PPLO) infection |
| H060A00 | Acute bronchitis due to mycoplasma pneumoniae |
| H2...00 | Pneumonia and influenza |
| H20..00 | Viral pneumonia |
| H20..11 | Chest infection - viral pneumonia |
| H20y.00 | Viral pneumonia NEC |
| H20z.00 | Viral pneumonia NOS |
| H21..00 | Lobar (pneumococcal) pneumonia |
| H21..11 | Chest infection - pneumococcal pneumonia |
| H22..00 | Other bacterial pneumonia |
| H22..11 | Chest infection - other bacterial pneumonia |
| H220.00 | Pneumonia due to klebsiella pneumoniae |
| H221.00 | Pneumonia due to pseudomonas |
| H222.00 | Pneumonia due to haemophilus influenzae |
| H222.11 | Pneumonia due to haemophilus influenzae |
| H223.00 | Pneumonia due to streptococcus |
| H223000 | Pneumonia due to streptococcus, group B |
| H224.00 | Pneumonia due to staphylococcus |
| H22y.00 | Pneumonia due to other specified bacteria |
| H22y000 | Pneumonia due to escherichia coli |
| H22y011 | E.coli pneumonia |
| H22y100 | Pneumonia due to proteus |
| H22y200 | Pneumonia - Legionella |
| H22yX00 | Pneumonia due to other aerobic gram-negative bacteria |
| H22yz00 | Pneumonia due to bacteria NOS |
| H22z.00 | Bacterial pneumonia NOS |
| H23..00 | Pneumonia due to other specified organisms |
| H23..11 | Chest infection - pneumonia organism OS |
| H230.00 | Pneumonia due to Eaton's agent |
| H231.00 | Pneumonia due to mycoplasma pneumoniae |
| H232.00 | Pneumonia due to pleuropneumonia like organisms |
| H23z.00 | Pneumonia due to specified organism NOS |
| H24..00 | Pneumonia with infectious diseases EC |
| H246.00 | Pneumonia with aspergillosis |
| H24y.00 | Pneumonia with other infectious diseases EC |
| H24yz00 | Pneumonia with other infectious diseases EC NOS |
| H24z.00 | Pneumonia with infectious diseases EC NOS |
| H25..00 | Bronchopneumonia due to unspecified organism |
| H25..11 | Chest infection - unspecified bronchopneumonia |
| H26..00 | Pneumonia due to unspecified organism |
| H260.00 | Lobar pneumonia due to unspecified organism |
| H261.00 | Basal pneumonia due to unspecified organism |
| H270.00 | Influenza with pneumonia |
| H270.11 | Chest infection - influenza with pneumonia |
| H270000 | Influenza with bronchopneumonia |
| H270100 | Influenza with pneumonia, influenza virus identified |
| H270z00 | Influenza with pneumonia NOS |
| H28..00 | Atypical pneumonia |
| H2y..00 | Other specified pneumonia or influenza |
| H2z..00 | Pneumonia or influenza NOS |
| H530300 | Abscess of lung with pneumonia |
| H56y100 | Interstitial pneumonia |
| H571.00 | Rheumatic pneumonia |
| Hyu0800 | [X]Other viral pneumonia |
| Hyu0900 | [X]Pneumonia due to other aerobic gram-negative bacteria |
| Hyu0A00 | [X]Other bacterial pneumonia |
| Hyu0B00 | [X]Pneumonia due to other specified infectious organisms |
| Hyu0C00 | [X]Pneumonia in bacterial diseases classified elsewhere |
| Hyu0D00 | [X]Pneumonia in viral diseases classified elsewhere |
| Hyu0E00 | [X]Pneumonia in mycoses classified elsewhere |
| Hyu0G00 | [X]Pneumonia in other diseases classified elsewhere |
| Hyu0H00 | [X]Other pneumonia, organism unspecified |
| H200.00 | Pneumonia due to adenovirus |
| H201.00 | Pneumonia due to respiratory syncytial virus |
| H202.00 | Pneumonia due to parainfluenza virus |
| H243.00 | Pneumonia with whooping cough |
| H243.11 | Pneumonia with pertussis |
| H24y700 | Pneumonia with varicella |
| H540000 | Hypostatic pneumonia |
| H540100 | Hypostatic bronchopneumonia |
| H564.00 | Bronchiolitis obliterans organising pneumonia |
| Hyu0F00 | [X]Pneumonia in parasitic diseases classified elsewhere |
| Q310.00 | Congenital pneumonia |
| Q310000 | Congenital pneumonia due to staphylococcus |
| Q310100 | Congenital pneumonia due to group A haemolytic streptococcus |
| Q310200 | Congenital pneumonia due to group B haemolytic streptococcus |
| Q310300 | Congenital pneumonia due to Escherichia coli |
| Q310400 | Congenital pneumonia due to pseudomonas |
| Q310500 | Congenital pneumonia due to viral agent |
| Q310600 | Congenital pneumonia due to Chlamydia |
| Q310y00 | Other specified congenital pneumonia |
| Q310z00 | Congenital pneumonia NOS |
| Qyu3100 | [X]Congenital pneumonia due to other bacterial agents |
| Qyu3200 | [X]Congenital pneumonia due to other organisms |
| SP13100 | Other aspiration pneumonia as a complication of care |

## Sinusitis

| **Read Code** | **Description** |
| --- | --- |
| **Those we believe should be included** | |
| H01..00 | Acute sinusitis |
| H01..11 | Sinusitis |
| H010.00 | Acute maxillary sinusitis |
| H011.00 | Acute frontal sinusitis |
| H012.00 | Acute ethmoidal sinusitis |
| H013.00 | Acute sphenoidal sinusitis |
| H01y.00 | Other acute sinusitis |
| H01y000 | Acute pansinusitis |
| H01yz00 | Other acute sinusitis NOS |
| H01z.00 | Acute sinusitis NOS |
| H130.12 | Maxillary sinusitis |
| H131.11 | Frontal sinusitis |
| H133.00 | Chronic sphenoidal sinusitis |
| H135.00 | Recurrent sinusitis |
| H13y100 | Pansinusitis |
| Hyu0000 | [X]Other acute sinusitis |
| SN31.11 | Aerosinusitis |

## Unspecific upper respiratory tract infection

| **Read Code** | **Description** |
| --- | --- |
| **Those we believe should be included** | |
| H051.00 | Acute upper respiratory tract infection |
| H054.00 | Recurrent upper respiratory tract infection |
| H05z.11 | Upper respiratory tract infection NOS |
| H05z.12 | Viral upper respiratory tract infection NOS |
| H1...00 | Other upper respiratory tract diseases |
| H1y..00 | Other specified diseases of upper respiratory tract |
| H1y8.00 | Upper respiratory tract hypersensitivity reaction NOS |
| H1yz.00 | Other upper respiratory tract diseases NOS |
| H1yzz00 | Other upper respiratory tract disease NOS |
| H1z..00 | Upper respiratory tract disease NOS |
| Hyu2.00 | [X]Other diseases of the upper respiratory tract |
| Hyu2A00 | [X]Other specified diseases of upper respiratory tract |

**Appendix II**

# ICD-10 codes for complications in HES

## Acute and Chronic Otitis Media

Acute otitis media

| **ICD-10 Code** | **Description** |
| --- | --- |
| **Those we believe should be included** | |
| H65.0 | **Acute serous otitis media** |
|  | Acute and subacute secretory otitis media |
| H65.1 | **Other acute nonsuppurative otitis media** |
|  | Otitis media, acute and subacute: |
|  | · allergic (mucoid)(sanguinous)(serous) |
|  | · mucoid |
|  | · nonsuppurative NOS |
|  | · sanguinous |
|  | · seromucinous |
|  | Excludes: otitic barotrauma ( T70.0 ) |
|  | otitis media (acute) NOS ( H66.9 ) |
| H66.0 | **Acute suppurative otitis media** |
| H67.1* | **Otitis media in viral diseases classified elsewhere** |
|  | Otitis media in: |
|  | · influenza ( J09-J11+ ) |

## Cardiovascular Disease

Acute myocardial infarction

| **ICD-10 Code** | **Description** |
| --- | --- |
| **Those we believe should be included** | |
| I21 | **Acute myocardial infarction** |
|  | Includes: myocardial infarction specified as acute or with a stated duration of 4 weeks (28 days) or less from onset |
|  | Excludes: certain current complications following acute myocardial infarction ( I23.- ) |
|  | myocardial infarction: |
|  | · old ( I25.2 ) |
|  | · specified as chronic or with a stated duration of more than 4 weeks (more than 28 days) from onset ( I25.8 ) |
|  | · subsequent ( I22.- ) |
|  | postmyocardial infarction syndrome ( I24.1 ) |
| I21.0 | **Acute transmural myocardial infarction of anterior wall** |
|  | Transmural infarction (acute)(of): |
|  | · anterior (wall) NOS |
|  | · anteroapical |
|  | · anterolateral |
|  | · anteroseptal |
| I21.1 | **Acute transmural myocardial infarction of inferior wall** |
|  | Transmural infarction (acute)(of): |
|  | · diaphragmatic wall |
|  | · inferior (wall) NOS |
|  | · inferolateral |
|  | · inferoposterior |
| I21.2 | **Acute transmural myocardial infarction of other sites** |
|  | Transmural infarction (acute)(of): |
|  | · apical-lateral |
|  | · basal-lateral |
|  | · high lateral |
|  | · lateral (wall) NOS |
|  | · posterior (true) |
|  | · posterobasal |
|  | · posterolateral |
|  | · posteroseptal |
|  | · septal NOS |
| I21.3 | **Acute transmural myocardial infarction of unspecified site** |
|  | Transmural myocardial infarction NOS |
| I21.4 | **Acute subendocardial myocardial infarction** |
|  | Nontransmural myocardial infarction NOS |
| I21.9 | **Acute myocardial infarction, unspecified** |
|  | Myocardial infarction (acute) NOS |
| I22 | **Subsequent myocardial infarction** |
|  | Includes: recurrent myocardial infarction |
|  | Excludes: specified as chronic or with a stated duration of more than 4 weeks (more than 28 days) from onset ( I25.8 ) |
| I22.0 | **Subsequent myocardial infarction of anterior wall** |
|  | Subsequent infarction (acute)(of): |
|  | · anterior (wall) NOS |
|  | · anteroapical |
|  | · anterolateral |
|  | · anteroseptal |
| I22.1 | **Subsequent myocardial infarction of inferior wall** |
|  | Subsequent infarction (acute)(of): |
|  | · diaphragmatic wall |
|  | · inferior (wall) NOS |
|  | · inferolateral |
|  | · inferoposterior |
| I22.8 | **Subsequent myocardial infarction of other sites** |
|  | Subsequent myocardial infarction (acute)(of): |
|  | · apical-lateral |
|  | · basal-lateral |
|  | · high lateral |
|  | · lateral (wall) NOS |
|  | · posterior (true) |
|  | · posterobasal |
|  | · posterolateral |
|  | · posteroseptal |
|  | · septal NOS |
| I22.9 | **Subsequent myocardial infarction of unspecified site** |

Heart failure

| **ICD-10 Code** | **Description** |
| --- | --- |
| **Those we believe should be included** | |
| I50.0 | **Congestive heart failure** |
|  | Congestive heart disease |
|  | Right ventricular failure (secondary to left heart failure) |

Hypertension

| **ICD-10 Code** | **Description** |
| --- | --- |
| **Those we believe should be included** | |
| I10 | **Essential (primary) hypertension** |
|  | High blood pressure |
|  | Hypertension (arterial)(benign)(essential)(malignant)(primary)(systemic) |
|  | Excludes: involving vessels of: |
|  | · brain ( I60-I69 ) |
|  | · eye ( H35.0 ) |
| I11 | **Hypertensive heart disease** |
|  | Includes any condition in I50.-, I51.4-I51.9 due to hypertension |
| I11.0 | **Hypertensive heart disease with (congestive) heart failure** |
|  | Hypertensive heart failure |
| I11.9 | **Hypertensive heart disease without (congestive) heart failure** |
|  | Hypertensive heart disease NOS |
| I12 | **Hypertensive renal disease** |
|  | Includes any condition in N00-N07, N18.-, N19 or N26 with any condition in I10 |
|  | arteriosclerosis of kidney |
|  | arteriosclerotic nephritis (chronic)(interstitial) |
|  | hypertensive nephropathy |
|  | nephrosclerosis |
|  | Excludes secondary hypertension ( I15.- ) |
| I12.0 | **Hypertensive renal disease with renal failure** |
|  | Hypertensive renal failure |
| I12.9 | **Hypertensive renal disease without renal failure** |
|  | Hypertensive renal disease NOS |
| I13 | **Hypertensive heart and renal disease** |
|  | Includes any condition in I11.- with any condition in I12.- |
|  | disease: |
|  | · cardiorenal |
|  | · cardiovascular renal |
| I13.0 | **Hypertensive heart and renal disease with (congestive) heart failure** |
| I13.1 | **Hypertensive heart and renal disease with renal failure** |
| I13.2 | **Hypertensive heart and renal disease with both (congestive) heart failure and renal failure** |
| I13.9 | **Hypertensive heart and renal disease, unspecified** |
| I15 | **Secondary hypertension** |
|  | Excluding involving vessels of: |
|  | · brain ( I60-I69 ) |
|  | · eye ( H35.0 ) |
| I15.0 | **Renovascular hypertension** |
| I15.1 | **Hypertension secondary to other renal disorders** |
| I15.2 | **Hypertension secondary to endocrine disorders** |
| I15.8 | **Other secondary hypertension** |
| I15.9 | **Secondary hypertension, unspecified** |

Myocarditis

| **ICD-10 Code** | **Description** |
| --- | --- |
| **Those we believe should be included** | |
| I09.0 | **Rheumatic myocarditis** |
|  | Excludes: myocarditis not specified as rheumatic ( I51.4 ) |
| I51.4 | **Myocarditis, unspecified** |
|  | Myocardial fibrosis |
|  | Myocarditis: |
|  | · NOS |
|  | · chronic (interstitial) |

## Central Nervous System Disease

Epilepsy

| **ICD-10 Code** | **Description** |
| --- | --- |
| **Those we believe should be included** | |
| G40 | **Epilepsy** |
|  | Excludes: Landau-Kleffner syndrome ( F80.3 ) |
|  | seizure (convulsive) NOS ( R56.8 ) |
|  | status epilepticus ( G41.- ) |
|  | Todd's paralysis ( G83.8 ) |
| G40.0 | **Localization-related (focal)(partial) idiopathic epilepsy and epileptic syndromes with seizures of localized onset** |
|  | Benign childhood epilepsy with centrotemporal EEG spikes |
|  | Childhood epilepsy with occipital EEG paroxysms |
| G40.1 | **Localization-related (focal)(partial) symptomatic epilepsy and epileptic syndromes with simple partial seizures** |
|  | Attacks without alteration of consciousness |
|  | Simple partial seizures developing into secondarily generalized seizures |
| G40.2 | **Localization-related (focal)(partial) symptomatic epilepsy and epileptic syndromes with complex partial seizures** |
|  | Attacks with alteration of consciousness, often with automatisms |
|  | Complex partial seizures developing into secondarily generalized seizures |
| G40.3 | **Generalized idiopathic epilepsy and epileptic syndromes** |
|  | Benign: |
|  | · myoclonic epilepsy in infancy |
|  | · neonatal convulsions (familial) |
|  | Childhood absence epilepsy [pyknolepsy] |
|  | Epilepsy with grand mal seizures on awakening |
|  | Juvenile: |
|  | · absence epilepsy |
|  | · myoclonic epilepsy [impulsive petit mal] |
|  | Nonspecific epileptic seizures: |
|  | · atonic |
|  | · clonic |
|  | · myoclonic |
|  | · tonic |
|  | · tonic-clonic |
| G40.4 | **Other generalized epilepsy and epileptic syndromes** |
|  | Epilepsy with: |
|  | · myoclonic absences |
|  | · myoclonic-astatic seizures |
|  | Infantile spasms |
|  | Lennox-Gastaut syndrome |
|  | Salaam attacks |
|  | Symptomatic early myoclonic encephalopathy |
|  | West's syndrome |
| G40.6 | **Grand mal seizures, unspecified (with or without petit mal)** |
| G40.7 | **Petit mal, unspecified, without grand mal seizures** |
| G40.8 | **Other epilepsy** |
|  | Epilepsies and epileptic syndromes undetermined as to whether they are focal or generalized |
| G40.9 | **Epilepsy, unspecified** |
|  | Epileptic: |
|  | · convulsions NOS |
|  | · fits NOS |
|  | · seizures NOS |
| G41 | **Status epilepticus** |
| G41.0 | **Grand mal status epilepticus** |
|  | Tonic-clonic status epilepticus |
|  | Excludes: epilepsia partialis continua [Kozhevnikof] ( G40.5 ) |
| G41.1 | **Petit mal status epilepticus** |
|  | Epileptic absence status |
| G41.2 | **Complex partial status epilepticus** |
| G41.8 | **Other status epilepticus** |
| G41.9 | **Status epilepticus, unspecified** |

Parkinson’s disease

| **ICD-10 Code** | **Description** |
| --- | --- |
| **Those we believe should be included** | |
| G20 | **Parkinson's disease** |
|  | Hemiparkinsonism |
|  | Paralysis agitans |
|  | Parkinsonism or Parkinson's disease: |
|  | · NOS |
|  | · idiopathic |
|  | · primary |

Psychosis

| **ICD-10 Code** | **Description** |
| --- | --- |
| **Those we believe should be included** | |
| F20 | **Schizophrenia** |
|  | Excludes schizophrenia: |
|  | · acute (undifferentiated) ( F23.2 ) |
|  | · cyclic ( F25.2 ) |
|  | schizophrenic reaction ( F23.2 ) |
|  | schizotypal disorder ( F21 ) |
| F20.0 | **Paranoid schizophrenia** |
|  | Excludes: involutional paranoid state ( F22.8 ) |
|  | paranoia ( F22.0 ) |
| F20.1 | **Hebephrenic schizophrenia** |
| F20.2 | **Catatonic schizophrenia** |
| F20.3 | **Undifferentiated schizophrenia** |
|  | acute schizophrenia-like psychotic disorder ( F23.2 ) |
|  | chronic undifferentiated schizophrenia ( F20.5 ) |
|  | post-schizophrenic depression ( F20.4 ) |
| F20.4 | **Post-schizophrenic depression** |
|  | A depressive episode, which may be prolonged, arising in the aftermath of a schizophrenic illness. Some schizophrenic symptoms, either "positive" or "negative", must still be present but they no longer dominate the clinical picture. These depressive states are associated with an increased risk of suicide. If the patient no longer has any schizophrenic symptoms, a depressive episode should be diagnosed (F32.-). If schizophrenic symptoms are still florid and prominent, the diagnosis should remain that of the appropriate schizophrenic subtype (F20.0-F20.3). |
| F20.5 | **Residual schizophrenia** |
| F20.6 | **Simple schizophrenia** |
| F20.8 | **Other schizophrenia** |
|  | Excludes brief schizophreniform disorders ( F23.2 ) |
| F20.9 | **Schizophrenia, unspecified** |
| F21 | **Schizotypal disorder** |
|  | Excludes: Asperger's syndrome ( F84.5 ) |
|  | schizoid personality disorder ( F60.1 ) |
| F22 | **Persistent delusional disorders** |
| F22.0 | **Delusional disorder** |
|  | Excludes paranoid: |
|  | · personality disorder ( F60.0 ) |
|  | · psychosis, psychogenic ( F23.3 ) |
|  | · reaction ( F23.3 ) |
|  | · schizophrenia ( F20.0 ) |
| F22.8 | **Other persistent delusional disorders** |
| F22.9 | **Persistent delusional disorder, unspecified** |
| F23 | **Acute and transient psychotic disorders** |
| F23.0 | **Acute polymorphic psychotic disorder without symptoms of schizophrenia** |
| F23.1 | **Acute polymorphic psychotic disorder with symptoms of schizophrenia** |
| F23.2 | **Acute schizophrenia-like psychotic disorder** |
|  | Excludes: organic delusional [schizophrenia-like] disorder ( F06.2 ) |
|  | schizophreniform disorders NOS ( F20.8 ) |
| F23.3 | **Other acute predominantly delusional psychotic disorders** |
| F23.8 | **Other acute and transient psychotic disorders** |
| F23.9 | **Acute and transient psychotic disorder, unspecified** |
| F24 | **Induced delusional disorder** |
| F25 | **Schizoaffective disorders** |
| F25.0 | **Schizoaffective disorder, manic type** |
| F25.1 | **Schizoaffective disorder, depressive type** |
| F25.2 | **Schizoaffective disorder, mixed type** |
| F25.8 | **Other schizoaffective disorders** |
| F25.9 | **Schizoaffective disorder, unspecified** |
| F28 | **Other nonorganic psychotic disorders** |
| F29 | **Unspecified nonorganic psychosis** |
|  | Excludes: mental disorder NOS ( F99 ) |
|  | organic or symptomatic psychosis NOS ( F09 ) |

## Cerebrovascular Disease

Cerebrovascular disease

| **ICD-10 Code** | **Description** |
| --- | --- |
| **Those we believe should be included** | |
| I60 | **Subarachnoid haemorrhage** |
|  | Includes: ruptured cerebral aneurysm |
|  | Excludes: sequelae of subarachnoid haemorrhage ( I69.0 ) |
| I60.0 | **Subarachnoid haemorrhage from carotid siphon and bifurcation** |
| I60.1 | **Subarachnoid haemorrhage from middle cerebral artery** |
| I60.2 | **Subarachnoid haemorrhage from anterior communicating artery** |
| I60.3 | **Subarachnoid haemorrhage from posterior communicating artery** |
| I60.4 | **Subarachnoid haemorrhage from basilar artery** |
| I60.5 | **Subarachnoid haemorrhage from vertebral artery** |
| I60.6 | **Subarachnoid haemorrhage from other intracranial arteries** |
|  | Multiple involvement of intracranial arteries |
| I60.7 | **Subarachnoid haemorrhage from intracranial artery, unspecified** |
|  | Ruptured (congenital) berry aneurysm NOS |
|  | Subarachnoid haemorrhage from: |
|  | · cerebral |
|  | · communicating |
| I60.8 | **Other subarachnoid haemorrhage** |
|  | Meningeal haemorrhage |
|  | Rupture of cerebral arteriovenous malformation |
| I60.9 | **Subarachnoid haemorrhage, unspecified** |
|  | Ruptured (congenital) cerebral aneurysm NOS |
| I61 | **Intracerebral haemorrhage** |
|  | Excludes: sequelae of intracerebral haemorrhage ( I69.1 ) |
| I61.0 | **Intracerebral haemorrhage in hemisphere, subcortical** |
|  | Deep intracerebral haemorrhage |
| I61.1 | **Intracerebral haemorrhage in hemisphere, cortical** |
|  | Cerebral lobe haemorrhage |
|  | Superficial intracerebral haemorrhage |
| I61.2 | **Intracerebral haemorrhage in hemisphere, unspecified** |
| I61.3 | **Intracerebral haemorrhage in brain stem** |
| I61.4 | **Intracerebral haemorrhage in cerebellum** |
| I61.5 | **Intracerebral haemorrhage, intraventricular** |
| I61.6 | **Intracerebral haemorrhage, multiple localized** |
| I61.8 | **Other intracerebral haemorrhage** |
| I61.9 | **Intracerebral haemorrhage, unspecified** |
| I62 | **Other nontraumatic intracranial haemorrhage** |
|  | Excludes: sequelae of intracranial haemorrhage ( I69.2 ) |
| I62.0 | **Subdural haemorrhage (acute)(nontraumatic)** |
| I62.1 | **Nontraumatic extradural haemorrhage** |
|  | Nontraumatic epidural haemorrhage |
| I62.9 | **Intracranial haemorrhage (nontraumatic), unspecified** |
| I63 | **Cerebral infarction** |
|  | Includes: occlusion and stenosis of cerebral and precerebral arteries, resulting in cerebral infarction |
|  | Excludes: sequelae of cerebral infarction ( I69.3 ) |
| I63.0 | **Cerebral infarction due to thrombosis of precerebral arteries** |
| I63.1 | **Cerebral infarction due to embolism of precerebral arteries** |
| I63.2 | **Cerebral infarction due to unspecified occlusion or stenosis of precerebral arteries** |
| I63.3 | **Cerebral infarction due to thrombosis of cerebral arteries** |
| I63.4 | **Cerebral infarction due to embolism of cerebral arteries** |
| I63.5 | **Cerebral infarction due to unspecified occlusion or stenosis of cerebral arteries** |
| I63.6 | **Cerebral infarction due to cerebral venous thrombosis, nonpyogenic** |
| I63.8 | **Other cerebral infarction** |
| I63.9 | **Cerebral infarction, unspecified** |
| I64 | **Stroke, not specified as haemorrhage or infarction** |
|  | Cerebrovascular accident NOS |
|  | Excludes: sequelae of stroke ( I69.4 ) |
| I65 | **Occlusion and stenosis of precerebral arteries, not resulting in cerebral infarction** |
|  | Includes: embolism |
|  | narrowing |
|  | obstruction (complete)(partial) |
|  | thrombosis |
|  | Excludes: when causing cerebral infarction ( I63.- ) |
| I65.0 | **Occlusion and stenosis of vertebral artery** |
| I65.1 | **Occlusion and stenosis of basilar artery** |
| I65.2 | **Occlusion and stenosis of carotid artery** |
| I65.3 | **Occlusion and stenosis of multiple and bilateral precerebral arteries** |
| I65.8 | **Occlusion and stenosis of other precerebral artery** |
| I65.9 | **Occlusion and stenosis of unspecified precerebral artery** |
|  | Precerebral artery NOS |
| I66 | **Occlusion and stenosis of cerebral arteries, not resulting in cerebral infarction** |
|  | Includes: embolism |
|  | narrowing |
|  | obstruction (complete)(partial) |
|  | thrombosis |
|  | Excludes: when causing cerebral infarction ( I63.- ) |
| I66.0 | **Occlusion and stenosis of middle cerebral artery** |
| I66.1 | **Occlusion and stenosis of anterior cerebral artery** |
| I66.2 | **Occlusion and stenosis of posterior cerebral artery** |
| I66.3 | **Occlusion and stenosis of cerebellar arteries** |
| I66.4 | **Occlusion and stenosis of multiple and bilateral cerebral arteries** |
| I66.8 | **Occlusion and stenosis of other cerebral artery** |
|  | Occlusion and stenosis of perforating arteries |
| I66.9 | **Occlusion and stenosis of unspecified cerebral artery** |
| I67 | **Other cerebrovascular diseases** |
|  | Excludes: sequelae of the listed conditions ( I69.8 ) |
| I67.0 | **Dissection of cerebral arteries, nonruptured** |
|  | Excludes: ruptured cerebral arteries ( I60.7 ) |
| I67.1 | **Cerebral aneurysm, nonruptured** |
|  | Cerebral: |
|  | · aneurysm NOS |
|  | · arteriovenous fistula, acquired |
|  | Excludes: congenital cerebral aneurysm, nonruptured ( Q28.- ) |
|  | ruptured cerebral aneurysm ( I60.9 ) |
| I67.2 | **Cerebral atherosclerosis** |
|  | Atheroma of cerebral arteries |
| I67.3 | **Progressive vascular leukoencephalopathy** |
|  | Binswanger's disease |
|  | Excludes: subcortical vascular dementia ( F01.2 ) |
| I67.4 | **Hypertensive encephalopathy** |
| I67.5 | **Moyamoya disease** |
| I67.6 | **Nonpyogenic thrombosis of intracranial venous system** |
|  | Nonpyogenic thrombosis of: |
|  | · cerebral vein |
|  | · intracranial venous sinus |
|  | Excludes: when causing infarction ( I63.6 ) |
| I67.7 | **Cerebral arteritis, not elsewhere classified** |
| I67.8 | **Other specified cerebrovascular diseases** |
|  | Acute cerebrovascular insufficiency NOS |
|  | Cerebral ischaemia (chronic) |
| I67.9 | **Cerebrovascular disease, unspecified** |
| I68* | **Cerebrovascular disorders in diseases classified elsewhere** |
| I68.0* | Cerebral amyloid angiopathy ( E85.-+ ) |
| I68.1* | **Cerebral arteritis in infectious and parasitic diseases classified elsewhere** |
|  | Cerebral arteritis: |
|  | · listerial ( A32.8+ ) |
|  | · syphilitic ( A52.0+ ) |
|  | · tuberculous ( A18.8+ ) |
| I68.2* | **Cerebral arteritis in other diseases classified elsewhere** |
|  | Cerebral arteritis in systemic lupus erythematosus ( M32.1+ ) |
| I68.8* | **Other cerebrovascular disorders in diseases classified elsewhere** |
| I69 | **Sequelae of cerebrovascular disease** |
| I69.0 | **Sequelae of subarachnoid haemorrhage** |
| I69.1 | **Sequelae of intracerebral haemorrhage** |
| I69.2 | **Sequelae of other nontraumatic intracranial haemorrhage** |
| I69.3 | **Sequelae of cerebral infarction** |
| I69.4 | **Sequelae of stroke, not specified as haemorrhage or infarction** |
| I69.8 | **Sequelae of other and unspecified cerebrovascular diseases** |

## Diabetes

Diabetes

| **ICD-10 Code** | **Description** |
| --- | --- |
| **Those we believe should be included** | |
| E10 | **Insulin-dependent diabetes mellitus** |
|  | Includes: diabetes (mellitus): |
|  | · brittle |
|  | · juvenile-onset |
|  | · ketosis-prone |
|  | · type I |
|  | excludes: diabetes mellitus (in): |
|  | · malnutrition-related ( E12.- ) |
|  | · neonatal ( P70.2 ) |
|  | · pregnancy, childbirth and the puerperium ( O24.- ) |
|  | glycosuria: |
|  | · NOS ( R81 ) |
|  | · renal ( E74.8 ) |
|  | impaired glucose tolerance ( R73.0 ) |
|  | postsurgical hypoinsulinaemia ( E89.1 ) |
| E11 | **Non-insulin-dependent diabetes mellitus** |
|  | Includes: diabetes (mellitus)(nonobese)(obese): |
|  | · adult-onset |
|  | · maturity-onset |
|  | · nonketotic |
|  | · stable |
|  | · type II |
|  | non-insulin-dependent diabetes of the young |
|  | Excludes: diabetes mellitus (in): |
|  | · malnutrition-related ( E12.- ) |
|  | · neonatal ( P70.2 ) |
|  | · pregnancy, childbirth and the puerperium ( O24.- ) |
|  | glycosuria: |
|  | · NOS ( R81 ) |
|  | · renal ( E74.8 ) |
|  | impaired glucose tolerance ( R73.0 ) |
|  | postsurgical hypoinsulinaemia ( E89.1 ) |
| E13 | **Other specified diabetes mellitus** |
|  | Excludes: diabetes mellitus (in): |
|  | · insulin-dependent ( E10.- ) |
|  | · malnutrition-related ( E12.- ) |
|  | · neonatal ( P70.2 ) |
|  | · non-insulin-dependent ( E11.- ) |
|  | · pregnancy, childbirth and the puerperium ( O24.- ) |
|  | glycosuria: |
|  | · NOS ( R81 ) |
|  | · renal ( E74.8 ) |
|  | impaired glucose tolerance ( R73.0 ) |
|  | postsurgical hypoinsulinaemia ( E89.1 ) |
| E14 | **Unspecified diabetes mellitus** |
|  | Includes: diabetes NOS |
|  | Excludes: diabetes mellitus (in): |
|  | · insulin-dependent ( E10.- ) |
|  | · malnutrition-related ( E12.- ) |
|  | · neonatal ( P70.2 ) |
|  | · non-insulin-dependent ( E11.- ) |
|  | · pregnancy, childbirth and the puerperium ( O24.- ) |
|  | glycosuria: |
|  | · NOS ( R81 ) |
|  | · renal ( E74.8 ) |
|  | impaired glucose tolerance ( R73.0 ) |
|  | postsurgical hypoinsulinaemia ( E89.1 ) |

## Gastrointestinal Disease

Gastrointestinal Bleeding

| **ICD-10 Code** | **Description** |
| --- | --- |
| **Those we believe should be included** | |
| K29.0 | **Acute haemorrhagic gastritis** |
|  | Acute (erosive) gastritis with haemorrhage |
|  | Excludes: erosion (acute) of stomach ( K25.- ) |
| K92.2 | **Gastrointestinal haemorrhage, unspecified** |
|  | Haemorrhage: |
|  | · gastric NOS |
|  | · intestinal NOS |
|  | Excludes: acute haemorrhagic gastritis ( K29.0 ) |
|  | haemorrhage of anus and rectum ( K62.5 ) |
|  | with peptic ulcer ( K25-K28 ) |

## Renal Disease

Myoglobrinuria

| **ICD-10 Code** | **Description** |
| --- | --- |
| **Those we believe should be included** | |
| R82.1 | **Myoglobinuria** |

Nephrotic syndrome

| **ICD-10 Code** | **Description** |
| --- | --- |
| **Those we believe should be included** | |
| N04 | **Nephrotic syndrome** |
|  | Includes: congenital nephrotic syndrome |
|  | lipoid nephrosis |

Renal disease

| **ICD-10 Code** | **Description** |
| --- | --- |
| **Those we believe should be included** | |
| N00 | **Acute nephritic syndrome** |
|  | Includes acute: |
|  | · glomerular disease |
|  | · glomerulonephritis |
|  | · nephritis |
|  | · renal disease NOS |
|  | Excludes: acute infectious tubulo-interstitial nephritis ( N10 ) |
|  | nephritic syndrome NOS ( N05.- ) |
| N01 | **Rapidly progressive nephritic syndrome** |
|  | Includes rapidly progressive: |
|  | · glomerular disease |
|  | · glomerulonephritis |
|  | · nephritis |
|  | Excludes nephritic syndrome NOS ( N05.- ) |
| N02 | **Recurrent and persistent haematuria** |
|  | Includes haematuria: |
|  | · benign (familial)(of childhood) |
|  | · with morphological lesion specified in .0-.8 before N00.- |
|  | Excludes haematuria NOS ( R31 ) |
| N03 | **Chronic nephritic syndrome** |
|  | Includes chronic: |
|  | · glomerular disease |
|  | · glomerulonephritis |
|  | · nephritis |
|  | · renal disease NOS |
|  | Excludes: chronic tubulo-interstitial nephritis ( N11.- ) |
|  | diffuse sclerosing glomerulonephritis ( N18.- ) |
|  | nephritic syndrome NOS ( N05.- ) |
| N04 | **Nephrotic syndrome** |
|  | Inludes: congenital nephrotic syndrome |
|  | lipoid nephrosis |
| N05 | **Unspecified nephritic syndrome** |
|  | Includes: glomerular disease |
|  | glomerulonephritis |
|  | nephritis |
|  | nephropathy NOS and renal disease NOS with morphological lesion specified in .0-.8 before N00.- |
|  | Excludes: nephropathy NOS with no stated morphological lesion ( N28.9 ) |
|  | renal disease NOS with no stated morphological lesion ( N28.9 ) |
|  | tubulo-interstitial nephritis NOS ( N12 ) |
| N06 | **Isolated proteinuria with specified morphological lesion** |
|  | Includes proteinuria (isolated)(orthostatic)(persistent) with morphological lesion specified in .0-.8 before N00.- |
|  | Excludes proteinuria: |
|  | · NOS ( R80 ) |
|  | · Bence Jones ( R80 ) |
|  | · gestational ( O12.1 ) |
|  | · isolated NOS ( R80 ) |
|  | · orthostatic NOS ( N39.2 ) |
|  | · persistent NOS ( N39.1 ) |
| N07 | **Hereditary nephropathy, not elsewhere classified** |
|  | Excludes: Alport's syndrome ( Q87.8 ) |
|  | hereditary amyloid nephropathy ( E85.0 ) |
|  | nail patella syndrome ( Q87.2 ) |
|  | non-neuropathic heredofamilial amyloidosis ( E85.0 ) |
| N08* | **Glomerular disorders in diseases classified elsewhere** |
|  | Includes nephropathy in diseases classified elsewhere |
|  | Excludes renal tubulo-interstitial disorders in diseases classified elsewhere ( N16.-* ) |
| N08.0* | **Glomerular disorders in infectious and parasitic diseases classified elsewhere** |
|  | Glomerular disorders in: |
|  | · Plasmodium malariae malaria ( B52.0+ ) |
|  | · mumps ( B26.8+ ) |
|  | · schistosomiasis [bilharziasis] ( B65.-+ ) |
|  | · septicaemia ( A40-A41+ ) |
|  | · strongyloidiasis ( B78.-+ ) |
|  | · syphilis ( A52.7+ ) |
| N08.1* | **Glomerular disorders in neoplastic diseases** |
|  | Glomerular disorders in: |
|  | · multiple myeloma ( C90.0+ ) |
|  | · Waldenström's macroglobulinaemia ( C88.0+ ) |
| N08.2* | **Glomerular disorders in blood diseases and disorders involving the immune mechanism** |
|  | Glomerular disorders in: |
|  | · cryoglobulinaemia ( D89.1+ ) |
|  | · disseminated intravascular coagulation [defibrination syndrome] ( D65+ ) |
|  | · haemolytic-uraemic syndrome ( D59.3+ ) |
|  | · Henoch(-Schönlein) purpura ( D69.0+ ) |
|  | · sickle-cell disorders ( D57.-+ ) |
| N08.3* | **Glomerular disorders in diabetes mellitus ( E10-E14+ with common fourth character .2)** |
| N08.4* | **Glomerular disorders in other endocrine, nutritional and metabolic diseases** |
|  | Glomerular disorders in: |
|  | · amyloidosis ( E85.-+ ) |
|  | · Fabry(-Anderson) disease ( E75.2+ ) |
|  | · lecithin cholesterol acyltransferase deficiency ( E78.6+ ) |
| N08.5* | **Glomerular disorders in systemic connective tissue disorders** |
|  | Glomerular disorders in: |
|  | · Goodpasture's syndrome ( M31.0+ ) |
|  | · microscopic polyangiitis ( M31.7+ ) |
|  | · systemic lupus erythematosus ( M32.1+ ) |
|  | · thrombotic thrombocytopenic purpura ( M31.1+ ) |
|  | · Wegener's granulomatosis ( M31.3+ ) |
| N08.8* | **Glomerular disorders in other diseases classified elsewhere** |
|  | Glomerular disorders in subacute bacterial endocarditis ( I33.0+ ) |
| N10 | **Acute tubulo-interstitial nephritis** |
|  | Acute: |
|  | · infectious interstitial nephritis |
|  | · pyelitis |
|  | · pyelonephritis |
| N11 | **Chronic tubulo-interstitial nephritis** |
|  | Includes chronic: |
|  | · infectious interstitial nephritis |
|  | · pyelitis |
|  | · pyelonephritis |
| N11.0 | **Nonobstructive reflux-associated chronic pyelonephritis** |
|  | Pyelonephritis (chronic) associated with (vesicoureteral) reflux |
|  | Excludes vesicoureteral reflux NOS ( N13.7 ) |
| N11.1 | **Chronic obstructive pyelonephritis** |
|  | Pyelonephritis (chronic) associated with: |
|  | · anomaly |
|  | · kinking |
|  | · obstruction |
|  | · stricture |
|  | Excludes: calculous pyelonephritis ( N20.9 ) |
|  | obstructive uropathy ( N13.- ) |
| N11.8 | **Other chronic tubulo-interstitial nephritis** |
|  | Nonobstructive chronic pyelonephritis NOS |
| N11.9 | **Chronic tubulo-interstitial nephritis, unspecified** |
|  | Chronic: |
|  | · interstitial nephritis NOS |
|  | · pyelitis NOS |
|  | · pyelonephritis NOS |
| N12 | **Tubulo-interstitial nephritis, not specified as acute or chronic** |
|  | Interstitial nephritis NOS |
|  | Pyelitis NOS |
|  | Pyelonephritis NOS |
|  | Excludes calculous pyelonephritis ( N20.9 ) |
| N13 | **Obstructive and reflux uropathy** |
|  | Excludes: calculus of kidney and ureter without hydronephrosis ( N20.- ) |
|  | congenital obstructive defects of renal pelvis and ureter ( Q62.0-Q62.3 ) |
|  | obstructive pyelonephritis ( N11.1 ) |
| N13.6 | **Pyonephrosis** |
|  | Conditions in N13.0-N13.5 with infection |
|  | Obstructive uropathy with infection |
| N13.7 | **Vesicoureteral-reflux-associated uropathy** |
|  | Vesicoureteral reflux: |
|  | · NOS |
|  | · with scarring |
|  | Excludes reflux-associated pyelonephritis ( N11.0 ) |
| N13.8 | **Other obstructive and reflux uropathy** |
| N13.9 | **Obstructive and reflux uropathy, unspecified** |
|  | Urinary tract obstruction NOS |
|  | Balkan endemic nephropathy |
| N15.1 | **Renal and perinephric abscess** |
| N15.8 | **Other specified renal tubulo-interstitial diseases** |
| N15.9 | **Renal tubulo-interstitial disease, unspecified** |
|  | Infection of kidney NOS |
|  | Excludes urinary tract infection NOS ( N39.0 ) |
| N16* | **Renal tubulo-interstitial disorders in diseases classified elsewhere** |
| N16.0* | **Renal tubulo-interstitial disorders in infectious and parasitic diseases classified elsewhere** |
|  | Renal tubulo-interstitial disorders (due to)(in): |
|  | · brucellosis ( A23.-+ ) |
|  | · diphtheria ( A36.8+ ) |
|  | · salmonella infection ( A02.2+ ) |
|  | · septicaemia ( A40-A41+ ) |
|  | · toxoplasmosis ( B58.8+ ) |
| N16.1* | **Renal tubulo-interstitial disorders in neoplastic diseases** |
|  | Renal tubulo-interstitial disorders in: |
|  | · leukaemia ( C91-C95+ ) |
|  | · lymphoma (C81-C85+, C96+) |
|  | · multiple myeloma ( C90.0+ ) |
| N16.2* | **Renal tubulo-interstitial disorders in blood diseases and disorders involving the immune mechanism** |
|  | Renal tubulo-interstitial disorders in: |
|  | · mixed cryoglobulinaemia ( D89.1+ ) |
|  | · sarcoidosis ( D86.-+ ) |
| N16.3* | **Renal tubulo-interstitial disorders in metabolic diseases** |
|  | Renal tubulo-interstitial disorders in: |
|  | · cystinosis ( E72.0+ ) |
|  | · glycogen storage disease ( E74.0+ ) |
|  | · Wilson's disease ( E83.0+ ) |
| N16.4* | **Renal tubulo-interstitial disorders in systemic connective tissue disorders** |
|  | Renal tubulo-interstitial disorders in: |
|  | · sicca syndrome [Sjögren] ( M35.0+ ) |
|  | · systemic lupus erythematosus ( M32.1+ ) |
| N16.5* | **Renal tubulo-interstitial disorders in transplant rejection ( T86.-+ )** |
| N16.8* | **Renal tubulo-interstitial disorders in other diseases classified elsewhere** |
| N17 | **Acute renal failure** |
|  | Includes acute renal impairment |
| N17.0 | **Acute renal failure with tubular necrosis** |
|  | Tubular necrosis: |
|  | · NOS |
|  | · acute |
|  | · renal |
| N17.1 | **Acute renal failure with acute cortical necrosis** |
|  | Cortical necrosis: |
|  | · NOS |
|  | · acute |
|  | · renal |
| N17.2 | **Acute renal failure with medullary necrosis** |
|  | Medullary [papillary] necrosis: |
|  | · NOS |
|  | · acute |
|  | · renal |
| N17.8 | **Other acute renal failure** |
| N17.9 | **Acute renal failure, unspecified** |
| N18 | **Chronic renal failure** |
|  | Includes: chronic uraemia |
|  | diffuse sclerosing glomerulonephritis |
|  | Escludes chronic renal failure with hypertension ( I12.0 ) |
| N18.0 | **End-stage renal disease** |
| N18.8 | **Other chronic renal failure** |
|  | Uraemic: |
|  | · neuropathy+ ( G63.8* ) |
|  | · pericarditis+ ( I32.8* ) |
| N18.9 | **Chronic renal failure, unspecified** |
| N19 | **Unspecified renal failure** |
|  | Uraemia NOS |
|  | Excludes: renal failure with hypertension ( I12.0 ) |
|  | uraemia of newborn ( P96.0 ) |

N23 Unspecified renal colic

##

## Respiratory Disease

Asthma

| **ICD-10 Code** | **Description** |
| --- | --- |
| **Those we believe should be included** | |
| J45 | **Asthma** |
|  | Excludes: acute severe asthma ( J46 ) |
|  | chronic asthmatic (obstructive) bronchitis ( J44.- ) |
|  | chronic obstructive asthma ( J44.- ) |
|  | eosinophilic asthma ( J82 ) |
|  | lung diseases due to external agents ( J60-J70 ) |
|  | status asthmaticus ( J46 ) |
| J45.0 | **Predominantly allergic asthma** |
|  | Allergic: |
|  | · bronchitis NOS |
|  | · rhinitis with asthma |
|  | Atopic asthma |
|  | Extrinsic allergic asthma |
|  | Hay fever with asthma |
| J45.1 | **Nonallergic asthma** |
|  | Idiosyncratic asthma |
|  | Intrinsic nonallergic asthma |
| J45.8 | **Mixed asthma** |
|  | Combination of conditions listed in J45.0 and J45.1 |
| J45.9 | **Asthma, unspecified** |
|  | Asthmatic bronchitis NOS |
|  | Late-onset asthma |
| J46 | **Status asthmaticus** |
|  | Acute severe asthma |

Bronchitis

| **ICD-10 Code** | **Description** |
| --- | --- |
| **Those we believe should be included** | |
| J40 | **Bronchitis, not specified as acute or chronic** |
| J20 | **Acute bronchitis** |
|  | Includes bronchitis: |
|  | · NOS, in those under l5 years of age |
|  | · acute and subacute (with): |
|  | · bronchospasm |
|  | · fibrinous |
|  | · membranous |
|  | · purulent |
|  | · septic |
|  | · tracheitis |
|  | tracheobronchitis, acute |
|  | Excludes bronchitis: |
|  | [· NOS, in those 15 years of age and above ( J40 )](http://apps.who.int/classifications/apps/icd/icd10online/gj40.htm#j40) |
|  | [· allergic NOS ( J45.0 )](http://apps.who.int/classifications/apps/icd/icd10online/gj40.htm#j450) |
|  | · chronic: |
|  | [· NOS ( J42 )](http://apps.who.int/classifications/apps/icd/icd10online/gj40.htm#j42) |
|  | [· mucopurulent ( J41.1 )](http://apps.who.int/classifications/apps/icd/icd10online/gj40.htm#j411) |
|  | [· obstructive ( J44.- )](http://apps.who.int/classifications/apps/icd/icd10online/gj40.htm#j44) |
|  | [· simple ( J41.0 )](http://apps.who.int/classifications/apps/icd/icd10online/gj40.htm#j410) |
|  | tracheobronchitis: |
|  | [· NOS ( J40 )](http://apps.who.int/classifications/apps/icd/icd10online/gj40.htm#j40) |
|  | [· chronic ( J42 )](http://apps.who.int/classifications/apps/icd/icd10online/gj40.htm#j42) |
|  | [· chronic obstructive ( J44.- )](http://apps.who.int/classifications/apps/icd/icd10online/gj40.htm#j44) |
| J20.0 | **Acute bronchitis due to Mycoplasma pneumoniae** |
| J20.1 | **Acute bronchitis due to Haemophilus influenzae** |
| J20.2 | **Acute bronchitis due to streptococcus** |
| J20.3 | **Acute bronchitis due to coxsackievirus** |
| J20.4 | **Acute bronchitis due to parainfluenza virus** |
| J20.5 | **Acute bronchitis due to respiratory syncytial virus** |
| J20.6 | **Acute bronchitis due to rhinovirus** |
| J20.7 | **Acute bronchitis due to echovirus** |
| J20.8 | **Acute bronchitis due to other specified organisms** |
| J20.9 | **Acute bronchitis, unspecified** |

Chronic obstructive pulmonary disease

| **ICD-10 Code** | **Description** |
| --- | --- |
| **Those we believe should be included** | |
| J40 | **Bronchitis, not specified as acute or chronic** |
|  | Bronchitis: |
|  | · NOS |
|  | · catarrhal |
|  | · with tracheitis NOS |
|  | Tracheobronchitis NOS |
|  | Excludes: bronchitis: |
|  | · allergic NOS ( J45.0 ) |
|  | · asthmatic NOS ( J45.9 ) |
|  | · chemical (acute) ( J68.0 ) |
| J41 | **Simple and mucopurulent chronic bronchitis** |
|  | Excludes: chronic bronchitis: |
|  | · NOS ( J42 ) |
|  | · obstructive ( J44.- ) |
| J41.0 | **Simple chronic bronchitis** |
| J41.1 | **Mucopurulent chronic bronchitis** |
| J41.8 | **Mixed simple and mucopurulent chronic bronchitis** |
| J42 | **Unspecified chronic bronchitis** |
|  | Chronic: |
|  | · bronchitis NOS |
|  | · tracheitis |
|  | · tracheobronchitis |
|  | Excludes: chronic: |
|  | · asthmatic bronchitis ( J44.- ) |
|  | · bronchitis: |
|  | · simple and mucopurulent ( J41.- ) |
|  | · with airways obstruction ( J44.- ) |
|  | · emphysematous bronchitis ( J44.- ) |
|  | · obstructive pulmonary disease NOS ( J44.9 ) |
| J43 | **Emphysema** |
|  | Excludes: emphysema: |
|  | · compensatory ( J98.3 ) |
|  | · due to inhalation of chemicals, gases, fumes or vapours ( J68.4 ) |
|  | · interstitial ( J98.2 ) |
|  | · neonatal ( P25.0 ) |
|  | · mediastinal ( J98.2 ) |
|  | · surgical (subcutaneous) ( T81.8 ) |
|  | · traumatic subcutaneous ( T79.7 ) |
|  | · with chronic (obstructive) bronchitis ( J44.- ) |
|  | emphysematous (obstructive) bronchitis ( J44.- ) |
| J43.0 | **MacLeod's syndrome** |
|  | Unilateral: |
|  | · emphysema |
|  | · transparency of lung |
| J43.1 | **Panlobular emphysema** |
|  | Panacinar emphysema |
| J43.2 | **Centrilobular emphysema** |
| J43.8 | **Other emphysema** |
| J43.9 | **Emphysema, unspecified** |
|  | Emphysema (lung)(pulmonary): |
|  | · NOS |
|  | · bullous |
|  | · vesicular |
|  | Emphysematous bleb |
| J44 | **Other chronic obstructive pulmonary disease** |
|  | Includes: chronic: |
|  | · bronchitis: |
|  | · asthmatic (obstructive) |
|  | · emphysematous |
|  | · with: |
|  | · airways obstruction |
|  | · emphysema |
|  | · obstructive: |
|  | · asthma |
|  | · bronchitis |
|  | · tracheobronchitis |
|  | Excludes: asthma ( J45.- ) |
|  | asthmatic bronchitis NOS ( J45.9 ) |
|  | bronchiectasis ( J47 ) |
|  | chronic: |
|  | · bronchitis: |
|  | · NOS ( J42 ) |
|  | · simple and mucopurulent ( J41.- ) |
|  | · tracheitis ( J42 ) |
|  | · tracheobronchitis ( J42 ) |
|  | emphysema ( J43.- ) |
|  | lung diseases due to external agents ( J60-J70 ) |
| J44.0 | **Chronic obstructive pulmonary disease with acute lower respiratory infection** |
|  | Excludes: with influenza ( J98-J11 ) |
| J44.1 | **Chronic obstructive pulmonary disease with acute exacerbation, unspecified** |
| J44.8 | **Other specified chronic obstructive pulmonary disease** |
|  | Chronic bronchitis: |
|  | · asthmatic (obstructive) NOS |
|  | · emphysematous NOS |
|  | · obstructive NOS |
|  | Excludes: with acute exacerbation ( J44.1 ) |
|  | with acute lower respiratory infection ( J44.0 ) |
| J44.9 | **Chronic obstructive pulmonary disease, unspecified** |
|  | Chronic obstructive: |
|  | · airway disease NOS |
|  | · lung disease NOS |
| J47 | **Bronchiectasis** |
|  | Bronchiolectasis |
|  | Excludes: congenital bronchiectasis ( Q33.4 ) |
|  | tuberculous bronchiectasis (current disease) ( A15-A16 ) |

Chronic Respiratory Disease

| **ICD-10 Code** | | **Description** |
| --- | --- | --- |
| E84 | **Cystic fibrosis** | |
|  | Includes: mucoviscidosis | |
| E84.0 | **Cystic fibrosis with pulmonary manifestations** | |
| E84.1 | **Cystic fibrosis with intestinal manifestations** | |
|  | Meconium ileus+ ( P75* ) | |
|  | Excludes: meconium obstruction in cases where cystic fibrosis is known not to be present ( P76.0 ) | |
| E84.8 | **Cystic fibrosis with other manifestations** | |
|  | Cystic fibrosis with combined manifestations | |
| E84.9 | **Cystic fibrosis, unspecified** | |
|  |  | |
| J80 | **Adult respiratory distress syndrome** | |
|  | Adult hyaline membrane disease | |
|  |  | |
| J81 | **Pulmonary oedema** | |
|  | Acute oedema of lung | |
|  | Pulmonary congestion (passive) | |
|  | Excludes: hypostatic pneumonia ( J18.2 ) | |
|  | pulmonary oedema: | |
|  | · chemical (acute) ( J68.1 ) | |
|  | · due to external agents ( J60-J70 ) | |
|  | · with mention of heart disease NOS or heart failure ( I50.1 ) | |
|  |  | |
| J82 | **Pulmonary eosinophilia, not elsewhere classified** | |
|  | Eosinophilic asthma | |
|  | Löffler's pneumonia | |
|  | Tropical (pulmonary) eosinophilia NOS | |
|  | Excludes due to: | |
|  | · aspergillosis ( B44.- ) | |
|  | · drugs ( J70.2-J70.4 ) | |
|  | · specified parasitic infection ( B50-B83 ) | |
|  | · systemic connective tissue disorders ( M30-M36 ) | |
| J84.0 | **Alveolar and parietoalveolar conditions** | |
|  | Alveolar proteinosis | |
|  | Pulmonary alveolar microlithiasis | |
| J84.1 | **Other interstitial pulmonary diseases with fibrosis** | |
|  | Diffuse pulmonary fibrosis | |
|  | Fibrosing alveolitis (cryptogenic) | |
|  | Hamman-Rich syndrome | |
|  | Idiopathic pulmonary fibrosis | |
|  | Excludes: pulmonary fibrosis (chronic): | |
|  | · due to inhalation of chemicals, gases, fumes or vapours ( J68.4 ) | |
|  | · following radiation ( J70.1 ) | |
| J84.8 | **Other specified interstitial pulmonary diseases** | |
| J84.9 | **Interstitial pulmonary disease, unspecified** | |
|  | Interstitial pneumonia NOS | |
| P27.0 | **Wilson-Mikity syndrome** | |
|  | Pulmonary dysmaturity | |
| P28.0 | **Primary atelectasis of newborn** | |
|  | Primary failure to expand terminal respiratory units | |
|  | Pulmonary: | |
|  | · hypoplasia associated with short gestation | |
|  | · immaturity NOS | |
| P28.1 | **Other and unspecified atelectasis of newborn** | |
|  | Atelectasis: | |
|  | · NOS | |
|  | · partial | |
|  | · secondary | |
|  | Resorption atelectasis without respiratory distress syndrome | |
| P28.2 | **Cyanotic attacks of newborn** | |
|  | Excludes: apnoea of newborn ( P28.3-P28.4 ) | |
| P28.3 | **Primary sleep apnoea of newborn** | |
|  | Sleep apnoea of newborn NOS | |
| P28.4 | **Other apnoea of newborn** | |
| P28.5 | **Respiratory failure of newborn** | |
| P28.8 | **Other specified respiratory conditions of newborn** | |
|  | Congenital (laryngeal) stridor NOS | |
|  | Snuffles in newborn | |
|  | Excludes: early congenital syphilitic rhinitis ( A50.0 ) | |
| P28.9 | **Respiratory condition of newborn, unspecified** | |

Emphysema

| **ICD-10 Code** | **Description** |
| --- | --- |
| **Those we believe should be included** | |
| J43 | **Emphysema** |
|  | Excludes: emphysema: |
|  | · compensatory ( J98.3 ) |
|  | · due to inhalation of chemicals, gases, fumes or vapours ( J68.4 ) |
|  | · interstitial ( J98.2 ) |
|  | · neonatal ( P25.0 ) |
|  | · mediastinal ( J98.2 ) |
|  | · surgical (subcutaneous) ( T81.8 ) |
|  | · traumatic subcutaneous ( T79.7 ) |
|  | · with chronic (obstructive) bronchitis ( J44.- ) |
|  | emphysematous (obstructive) bronchitis ( J44.- ) |
| J43.0 | **MacLeod's syndrome** |
|  | Unilateral: |
|  | · emphysema |
|  | · transparency of lung |
| J43.1 | **Panlobular emphysema** |
|  | Panacinar emphysema |
| J43.2 | **Centrilobular emphysema** |
| J43.8 | **Other emphysema** |
| J43.9 | **Emphysema, unspecified** |
|  | Emphysema (lung)(pulmonary): |
|  | · NOS |
|  | · bullous |
|  | · vesicular |
|  | Emphysematous bleb |

Influenza

| **ICD-10 Code** | **Description** |
| --- | --- |
| **Those we believe should be included** | |
| J10 | **Influenza due to other identified influenza virus**  Excludes: Haemophilus influenzae [H. influenzae], infection NOS (A49.2), meningitis (G00.0), and pneumonia (J14) |
| J10.0 | **Influenza with pneumonia, other influenza virus identified**  Influenzal (broncho) pneumonia, other influenza virus identified |
| J10.1 | **Influenza with other respiratory manifestations, other influenza virus identified**  Influenza  Influenzal:   - acute upper respiratory infection - laryngitis - pharyngitis - pleural effusion |
| J10.8 | **Influenza with other manifestations, other influenza virus identified**  Encephalopathy due to influenza  Influenzal:   - gastroenteritis - myocarditis (acute) |
| J11 | Influenza, virus not identified  Includes: influenza and viral influenza, where the specific virus is not stated to have been identified  Excludes: Haemophilus influenzae [H. influenzae],·infection NOS (A49.2), meningitis (G00.0), pneumonia (J14) |
| J11.0 | **Influenza with pneumonia, virus not identified**  Influenzal (broncho)pneumonia, unspecified or specific virus not identified |
| J11.1 | **Influenza with other respiratory manifestations, virus not unspecified or identified**  Influenza NOS  Influenzal:   - acute upper respiratory infection - laryngitis - pharyngitis - pleural effusion |
| J11.8 | **Influenza with other manifestations, virus not unspecified or identified**  Encephalopathy due to influenza  Influenzal:   - gastroenteritis - myocarditis (acute) |

Laryngitis & Tracheitis

| **ICD-10 Code** | **Description** |
| --- | --- |
| **Those we believe should be included** | |
| J04.0 | **Acute laryngitis** |
|  | Laryngitis (acute): |
|  | · NOS |
|  | · oedematous |
|  | · subglottic |
|  | · suppurative |
|  | · ulcerative |
|  | Excludes: chronic laryngitis ( J37.0 ) |
|  | influenzal laryngitis, influenza virus: |
|  | · identified (J09, J10.1) |
|  | · not identified ( J11.1 ) |
| J04.1 | **Acute tracheitis** |
|  | Tracheitis (acute): |
|  | · NOS |
|  | · catarrhal |
|  | Excludes chronic tracheitis ( J42 ) |
| J04.2 | **Acute laryngotracheitis** |
|  | Laryngotracheitis NOS |
|  | Tracheitis (acute) with laryngitis (acute) |
|  | Excludes chronic laryngotracheitis ( J37.1 ) |
| J37.0 | **Chronic laryngitis** |
|  | Laryngitis: |
|  | · catarrhal |
|  | · hypertrophic |
|  | · sicca |
|  | Excludes: laryngitis: |
|  | · NOS ( J04.0 ) |
|  | · acute ( J04.0 ) |
|  | · obstructive (acute) ( J05.0 ) |
| J42 | **Unspecified chronic bronchitis** |
|  | Chronic: |
|  | · bronchitis NOS |
|  | · tracheitis |
|  | · tracheobronchitis |
|  | Excludes chronic: |
|  | · asthmatic bronchitis ( J44.- ) |
|  | · bronchitis: |
|  | · simple and mucopurulent ( J41.- ) |
|  | · with airways obstruction ( J44.- ) |
|  | · emphysematous bronchitis ( J44.- ) |
|  | · obstructive pulmonary disease NOS ( J44.9 ) |

Laryngotracheobronchitis (croup)

| **ICD-10 Code** | **Description** |
| --- | --- |
| **Those we believe should be included** | |
| J05.0 | **Acute obstructive laryngitis [croup]** |
|  | Obstructive laryngitis NOS |

Lung abscess

| **ICD-10 Code** | **Description** |
| --- | --- |
| **Those we believe should be included** | |
| J85 | **Abscess of lung and mediastinum** |
| J85.0 | **Gangrene and necrosis of lung** |
| J85.1 | **Abscess of lung with pneumonia** |
|  | Excludes: with pneumonia due to specified organism ( J09-J16 ) |
| J85.2 | **Abscess of lung without pneumonia** |
|  | Abscess of lung NOS |
| J85.3 | **Abscess of mediastinum** |

Nasopharyngitis

| **ICD-10 Code** | **Description** |
| --- | --- |
| **Those we believe should be included** | |
| J00 | **Acute nasopharyngitis [common cold]** |
|  | Coryza (acute) |
|  | Nasal catarrh, acute |
|  | Nasopharyngitis: |
|  | · NOS |
|  | · infective NOS |
|  | Rhinitis: |
|  | · acute |
|  | · infective |
|  | Excludes: nasopharyngitis, chronic ( J31.1 ) |
|  | pharyngitis: |
|  | · NOS ( J02.9 ) |
|  | · acute ( J02.- ) |
|  | · chronic ( J31.2 ) |
|  | rhinitis: |
|  | · NOS ( J31.0 ) |
|  | · allergic ( J30.1-J30.4 ) |
|  | · chronic ( J31.0 ) |
|  | · vasomotor ( J30.0 ) |
|  | sore throat: |
|  | · NOS ( J02.9 ) |
|  | · acute ( J02.- ) |
|  | · chronic ( J31.2 ) |
| J31.1 | **Chronic nasopharyngitis** |
|  | Excludes nasopharyngitis, acute or NOS ( J00 ) |

Pharyngitis acute & chronic

| **ICD-10 Code** | **Description** |
| --- | --- |
| **Those we believe should be included** | |
| J02 | **Acute pharyngitis** |
|  | Includes acute sore throat |
|  | Excludes abscess: |
|  | · peritonsillar ( J36 ) |
|  | · pharyngeal ( J39.1 ) |
|  | · retropharyngeal ( J39.0 ) |
|  | acute laryngopharyngitis ( J06.0 ) |
|  | chronic pharyngitis ( J31.2 ) |
| J02.0 | **Streptococcal pharyngitis** |
|  | Streptococcal sore throat |
|  | Excludes scarlet fever ( A38 ) |
| J02.9 | **Acute pharyngitis, unspecified** |
|  | Pharyngitis (acute): |
|  | · NOS |
|  | · gangrenous |
|  | · infective NOS |
|  | · suppurative |
|  | · ulcerative |
|  | Sore throat (acute) NOS |
| J31.2 | **Chronic pharyngitis** |
|  | Chronic sore throat |
|  | Pharyngitis (chronic): |
|  | · atrophic |
|  | · granular |
|  | · hypertrophic |
|  | Excludes pharyngitis, acute or NOS ( J02.9 ) |

Pneumonia and secondary bacterial pneumonia

| **ICD-10 Code** | **Description** |
| --- | --- |
| **Those we believe should be included** | |
| J12 | **Viral pneumonia, not elsewhere classified** |
|  | Includes bronchopneumonia due to viruses other than influenza viruses |
|  | Excludes: congenital rubella pneumonitis ( P35.0 ) |
|  | pneumonia: |
|  | · aspiration (due to): |
|  | · NOS ( J69.0 ) |
|  | · anaesthesia during: |
|  | · labour and delivery ( O74.0 ) |
|  | · pregnancy ( O29.0 ) |
|  | · puerperium ( O89.0 ) |
|  | · neonatal ( P24.9 ) |
|  | · solids and liquids ( J69.- ) |
|  | · congenital ( P23.0 ) |
|  | · in influenza (J09, J10.0, J11.0) |
|  | · interstitial NOS ( J84.9 ) |
|  | · lipid ( J69.1 ) |
|  | severe acute respiratory syndrome [SARS] ( U04.9 ) |
| J12.0 | **Adenoviral pneumonia** |
| J12.1 | **Respiratory syncytial virus pneumonia** |
| J12.2 | **Parainfluenza virus pneumonia** |
| J12.8 | **Other viral pneumonia** |
| J12.9 | **Viral pneumonia, unspecified** |
| J13 | **Pneumonia due to Streptococcus pneumoniae** |
|  | Bronchopneumonia due to S. pneumoniae |
|  | Excludes: congenital pneumonia due to S. pneumoniae ( P23.6 ) |
|  | pneumonia due to other streptococci ( J15.3-J15.4 ) |
| J14 | **Pneumonia due to Haemophilus influenzae** |
|  | Bronchopneumonia due to H. influenzae |
|  | Excludes congenital pneumonia due to H. influenzae ( P23.6 ) |
| J15 | **Bacterial pneumonia, not elsewhere classified** |
|  | Includes bronchopneumonia due to bacteria other than S. pneumoniae and H. influenzae |
|  | Excludes: chlamydial pneumonia ( J16.0 ) |
|  | congenital pneumonia ( P23.- ) |
|  | Legionnaires' disease ( A48.1 ) |
| J15.0 | **Pneumonia due to Klebsiella pneumoniae** |
| J15.1 | **Pneumonia due to Pseudomonas** |
| J15.2 | **Pneumonia due to staphylococcus** |
| J15.3 | **Pneumonia due to streptococcus, group B** |
| J15.4 | **Pneumonia due to other streptococci** |
|  | pneumonia due to: |
|  | · streptococcus, group B ( J15.3 ) |
|  | · Streptococcus pneumoniae ( J13 ) |
| J15.5 | **Pneumonia due to Escherichia coli** |
| J15.6 | **Pneumonia due to other aerobic Gram-negative bacteria** |
|  | Pneumonia due to Serratia marcescens |
| J15.7 | **Pneumonia due to Mycoplasma pneumoniae** |
| J15.8 | **Other bacterial pneumonia** |
| J15.9 | **Bacterial pneumonia, unspecified** |
| J16 | **Pneumonia due to other infectious organisms, not elsewhere classified** |
|  | Excludes: ornithosis ( A70 ) |
|  | pneumocystosis ( B59 ) |
|  | pneumonia: |
|  | · NOS ( J18.9 ) |
|  | · congenital ( P23.- ) |
| J16.0 | **Chlamydial pneumonia** |
| J16.8 | **Pneumonia due to other specified infectious organisms** |
| J17* | **Pneumonia in diseases classified elsewhere** |
| J17.0* | **Pneumonia in bacterial diseases classified elsewhere** |
|  | Pneumonia (due to)(in): |
|  | · actinomycosis ( A42.0+ ) |
|  | · anthrax ( A22.1+ ) |
|  | · gonorrhoea ( A54.8+ ) |
|  | · nocardiosis ( A43.0+ ) |
|  | · salmonella infection ( A02.2+ ) |
|  | · tularaemia ( A21.2+ ) |
|  | · typhoid fever ( A01.0+ ) |
|  | · whooping cough ( A37.-+ ) |
| J17.1* | **Pneumonia in viral diseases classified elsewhere** |
|  | Pneumonia in: |
|  | · cytomegalovirus disease ( B25.0+ ) |
|  | · measles ( B05.2+ ) |
|  | · rubella ( B06.8+ ) |
|  | · varicella ( B01.2+ ) |
| J17.2* | **Pneumonia in mycoses** |
|  | Pneumonia in: |
|  | · aspergillosis ( B44.0-B44.1+ ) |
|  | · candidiasis ( B37.1+ ) |
|  | · coccidioidomycosis ( B38.0-B38.2+ ) |
|  | · histoplasmosis ( B39.-+ ) |
| J17.3* | **Pneumonia in parasitic diseases** |
|  | Pneumonia in: |
|  | · ascariasis ( B77.8+ ) |
|  | · schistosomiasis ( B65.-+ ) |
|  | · toxoplasmosis ( B58.3+ ) |
| J17.8* | **Pneumonia in other diseases classified elsewhere** |
|  | Pneumonia (in): |
|  | · ornithosis ( A70+ ) |
|  | · Q fever ( A78+ ) |
|  | · rheumatic fever ( I00+ ) |
|  | · spirochaetal, not elsewhere classified ( A69.8+ ) |
| J18 | **Pneumonia, organism unspecified** |
|  | Excludes: abscess of lung with pneumonia ( J85.1 ) |
|  | drug-induced interstitial lung disorders ( J70.2-J70.4 ) |
|  | pneumonia: |
|  | · aspiration (due to): |
|  | · NOS ( J69.0 ) |
|  | · anaesthesia during: |
|  | · labour and delivery ( O74.0 ) |
|  | · pregnancy ( O29.0 ) |
|  | · puerperium ( O89.0 ) |
|  | · neonatal ( P24.9 ) |
|  | · solids and liquids ( J69.- ) |
|  | · congenital ( P23.9 ) |
|  | · interstitial NOS ( J84.9 ) |
|  | · lipid ( J69.1 ) |
|  | pneumonitis, due to external agents ( J67-J70 ) |
| J18.0 | **Bronchopneumonia, unspecified** |
|  | Excludes bronchiolitis ( J21.- ) |
| J18.1 | **Lobar pneumonia, unspecified** |
| J18.2 | **Hypostatic pneumonia, unspecified** |
| J18.8 | **Other pneumonia, organism unspecified** |
| J18.9 | **Pneumonia, unspecified** |

Other Diseases of Upper Respiratory Tract

| **ICD-10 Code** | **Description** |
| --- | --- |
| **Those we believe should be included** | |

| J30 | **Vasomotor and allergic rhinitis** |
| --- | --- |
|  | Includes spasmodic rhinorrhoea |
|  | Excludes: allergic rhinitis with asthma ( J45.0 ) |
|  | rhinitis NOS ( J31.0 ) |
| J30.0 | **Vasomotor rhinitis** |
| J30.1 | **Allergic rhinitis due to pollen** |
|  | Allergy NOS due to pollen |
|  | Hay fever |
|  | Pollinosis |
| J30.2 | **Other seasonal allergic rhinitis** |
| J30.3 | **Other allergic rhinitis** |
|  | Perennial allergic rhinitis |
| J30.4 | **Allergic rhinitis, unspecified** |
| J31.0 | **Chronic rhinitis** |
|  | Ozena |
|  | Rhinitis (chronic): |
|  | · NOS |
|  | · atrophic |
|  | · granulomatous |
|  | · hypertrophic |
|  | · obstructive |
|  | · purulent |
|  | · ulcerative |
|  | Excludes rhinitis: |
|  | · allergic ( J30.1-J30.4 ) |
|  | · vasomotor ( J30.0 ) |
| J33 | **Nasal polyp** |
|  | Excludes adenomatous polyps ( D14.0 ) |
| J33.0 | **Polyp of nasal cavity** |
|  | Polyp: |
|  | · choanal |
|  | · nasopharyngeal |
| J33.1 | **Polypoid sinus degeneration** |
|  | Woakes' syndrome or ethmoiditis |
| J33.8 | **Other polyp of sinus** |
|  | Polyp of sinus: |
|  | · accessory |
|  | · ethmoidal |
|  | · maxillary |
|  | · sphenoidal |
| J33.9 | **Nasal polyp, unspecified** |
| J34 | **Other disorders of nose and nasal sinuses** |
|  | Excludes varicose ulcer of nasal septum ( I86.8 ) |
| J34.0 | **Abscess, furuncle and carbuncle of nose** |
|  | Cellulitis |
|  | Necrosis |
|  | Ulceration |
| J34.1 | **Cyst and mucocele of nose and nasal sinus** |
| J34.2 | **Deviated nasal septum** |
|  | Deflection or deviation of septum (nasal)(acquired) |
| J34.3 | **Hypertrophy of nasal turbinates** |
| J34.8 | **Other specified disorders of nose and nasal sinuses** |
|  | Perforation of nasal septum NOS |
|  | Rhinolith |
| J35.1 | **Hypertrophy of tonsils** |
|  | Enlargement of tonsils |
| J35.2 | **Hypertrophy of adenoids** |
|  | Enlargement of adenoids |
| J35.3 | **Hypertrophy of tonsils with hypertrophy of adenoids** |
| J35.8 | **Other chronic diseases of tonsils and adenoids** |
|  | Adenoid vegetations |
|  | Amygdalolith |
|  | Cicatrix of tonsil (and adenoid) |
|  | Tonsillar tag |
|  | Ulcer of tonsil |
| J35.9 | **Chronic disease of tonsils and adenoids, unspecified** |
|  | Disease (chronic) of tonsils and adenoids NOS |
| J36 | **Peritonsillar abscess** |
|  | Abscess of tonsil |
|  | Peritonsillar cellulitis |
|  | Quinsy |
|  | Excludes: retropharyngeal abscess ( J39.0 ) |
|  | tonsillitis: |
|  | · NOS ( J03.9 ) |
|  | · acute ( J03.- ) |
|  | · chronic ( J35.0 ) |
| J38 | **Diseases of vocal cords and larynx, not elsewhere classified** |
|  | Excludes laryngitis: |
|  | · obstructive (acute) ( J05.0 ) |
|  | · ulcerative ( J04.0 ) |
|  | postprocedural subglottic stenosis ( J95.5 ) |
|  | stridor: |
|  | · congenital laryngeal NOS ( P28.8 ) |
|  | · NOS ( R06.1 ) |
| J38.0 | **Paralysis of vocal cords and larynx** |
|  | Laryngoplegia |
|  | Paralysis of glottis |
| J38.1 | **Polyp of vocal cord and larynx** |
|  | Excludes adenomatous polyps ( D14.1 ) |
| J38.2 | **Nodules of vocal cords** |
|  | Chorditis (fibrinous)(nodosa)(tuberosa) |
|  | Singer's nodes |
|  | Teacher's nodes |
| J38.3 | **Other diseases of vocal cords** |
|  | Abscess |
|  | Cellulitis |
|  | Granuloma |
|  | Leukokeratosis |
|  | Leukoplakia |
| J38.4 | **Oedema of larynx** |
|  | Oedema (of): |
|  | · glottis |
|  | · subglottic |
|  | · supraglottic |
|  | Excludes laryngitis: |
|  | · acute obstructive [croup] ( J05.0 ) |
|  | · oedematous ( J04.0 ) |
| J38.5 | **Laryngeal spasm** |
|  | Laryngismus (stridulus) |
| J38.6 | **Stenosis of larynx** |
| J38.7 | **Other diseases of larynx** |
|  | Abscess |
|  | Cellulitis |
|  | Disease NOS |
|  | Necrosis |
|  | Pachyderma |
|  | Perichondritis |
|  | Ulcer |
| J39 | **Other diseases of upper respiratory tract** |
|  | Excludes acute respiratory infection NOS ( J22 ) |
|  | · upper ( J06.9 ) |
|  | upper respiratory inflammation due to chemicals, gases, fumes or vapours ( J68.2 ) |
| J39.0 | **Retropharyngeal and parapharyngeal abscess** |
|  | Peripharyngeal abscess |
|  | Excludes peritonsillar abscess ( J36 ) |
| J39.1 | **Other abscess of pharynx** |
|  | Cellulitis of pharynx |
|  | Nasopharyngeal abscess |
| J39.2 | **Other diseases of pharynx** |
|  | Cyst |
|  | Oedema |
|  | Excludes pharyngitis: |
|  | · chronic ( J31.2 ) |
|  | · ulcerative ( J02.9 ) |
| J39.3 | **Upper respiratory tract hypersensitivity reaction, site unspecified** |
| J39.8 | **Other specified diseases of upper respiratory tract** |
| J39.9 | **Disease of upper respiratory tract, unspecified** |

Pneumothorax

| **ICD-10 Code** | **Description** |
| --- | --- |
| **Those we believe should be included** | |
| J93 | **Pneumothorax** |
|  | Excludes pneumothorax: |
|  | · congenital or perinatal ( P25.1 ) |
|  | · traumatic ( S27.0 ) |
|  | · tuberculous (current disease) ( A15-A16 ) |
|  | pyopneumothorax ( J86.- ) |
| J93.0 | **Spontaneous tension pneumothorax** |
| J93.1 | **Other spontaneous pneumothorax** |
| J93.8 | **Other pneumothorax** |
| J93.9 | **Pneumothorax, unspecified** |

Pulmonary aspergillosis

| **ICD-10 Code** | **Description** |
| --- | --- |
| **Those we believe should be included** | |
| B44.0 | **Invasive pulmonary aspergillosis** |
| B44.1 | **Other pulmonary aspergillosis** |

Sinusitis

| **ICD-10 Code** | **Description** |
| --- | --- |
| **Those we believe should be included** | |
| J01 | **Acute sinusitis** |
|  | Includes: abscess |
|  | empyema |
|  | infection |
|  | inflammation |
|  | suppuration |
|  | Excludes sinusitis, chronic or NOS ( J32.- ) |
| J01.0 | **Acute maxillary sinusitis** |
|  | Acute antritis |
| J01.1 | **Acute frontal sinusitis** |
| J01.2 | **Acute ethmoidal sinusitis** |
| J01.3 | **Acute sphenoidal sinusitis** |
| J01.4 | **Acute pansinusitis** |
| J01.8 | **Other acute sinusitis** |
|  | Acute sinusitis involving more than one sinus but not pansinusitis |
| J01.9 | **Acute sinusitis, unspecified** |
| J32 | **Chronic sinusitis** |
|  | Includes: abscess |
|  | empyema |
|  | infection |
|  | suppuration |
|  | Excludes acute sinusitis ( J01.- ) |
| J32.0 | **Chronic maxillary sinusitis** |
|  | Antritis (chronic) |
|  | Maxillary sinusitis NOS |
| J32.1 | **Chronic frontal sinusitis** |
|  | Frontal sinusitis NOS |
| J32.2 | **Chronic ethmoidal sinusitis** |
|  | Ethmoidal sinusitis NOS |
| J32.3 | **Chronic sphenoidal sinusitis** |
|  | Sphenoidal sinusitis NOS |
| J32.4 | **Chronic pansinusitis** |
|  | Pansinusitis NOS |
| J32.8 | **Other chronic sinusitis** |
|  | Sinusitis (chronic) involving more than one sinus but not pansinusitis |
| J32.9 | **Chronic sinusitis, unspecified** |
|  | Sinusitis (chronic) NOS |

Tonsillitis

| **ICD-10 Code** | **Description** |
| --- | --- |
| **Those we believe should be included** | |
| J03 | **Acute tonsillitis** |
|  | Excludes: peritonsillar abscess ( J36 ) |
|  | sore throat: |
|  | · NOS ( J02.9 ) |
|  | · acute ( J02.- ) |
|  | · streptococcal ( J02.0 ) |
| J03.0 | **Streptococcal tonsillitis** |
| J03.8 | **Acute tonsillitis due to other specified organisms** |
|  | Excludes herpesviral [herpes simplex] pharyngotonsillitis ( B00.2 ) |
| J03.9 | **Acute tonsillitis, unspecified** |
|  | Tonsillitis (acute): |
|  | · NOS |
|  | · follicular |
|  | · gangrenous |
|  | · infective |
|  | · ulcerative |
| J35.0 | **Chronic tonsillitis** |
|  | Excludes tonsillitis: |
|  | · NOS ( J03.9 ) |
|  | · acute ( J03.- ) |

Unspecific upper respiratory tract infection

| J06 | **Acute upper respiratory infections of multiple and unspecified sites** |
| --- | --- |
|  | Excludes: acute respiratory infection NOS ( J22 ) |
|  | influenza virus: |
|  | · identified (J09, J10.1) |
|  | · not identified ( J11.1 ) |
| J06.0 | **Acute laryngopharyngitis** |
| J06.8 | **Other acute upper respiratory infections of multiple sites** |
| J06.9 | **Acute upper respiratory infection, unspecified** |
|  | Upper respiratory: |
|  | · disease, acute |
|  | · infection NOS |
| J30 | **Vasomotor and allergic rhinitis** |
|  | Includes spasmodic rhinorrhoea |
|  | Excludes: allergic rhinitis with asthma ( J45.0 ) |
|  | rhinitis NOS ( J31.0 ) |
| J30.0 | **Vasomotor rhinitis** |
| J30.1 | **Allergic rhinitis due to pollen** |
|  | Allergy NOS due to pollen |
|  | Hay fever |
|  | Pollinosis |
| J30.2 | **Other seasonal allergic rhinitis** |
| J30.3 | **Other allergic rhinitis** |
|  | Perennial allergic rhinitis |
| J30.4 | **Allergic rhinitis, unspecified** |
| J31.0 | **Chronic rhinitis** |
|  | Ozena |
|  | Rhinitis (chronic): |
|  | · NOS |
|  | · atrophic |
|  | · granulomatous |
|  | · hypertrophic |
|  | · obstructive |
|  | · purulent |
|  | · ulcerative |
|  | Excludes rhinitis: |
|  | · allergic ( J30.1-J30.4 ) |
|  | · vasomotor ( J30.0 ) |
| J33 | **Nasal polyp** |
|  | Excludes adenomatous polyps ( D14.0 ) |
| J33.0 | **Polyp of nasal cavity** |
|  | Polyp: |
|  | · choanal |
|  | · nasopharyngeal |
| J33.1 | **Polypoid sinus degeneration** |
|  | Woakes' syndrome or ethmoiditis |
| J33.8 | **Other polyp of sinus** |
|  | Polyp of sinus: |
|  | · accessory |
|  | · ethmoidal |
|  | · maxillary |
|  | · sphenoidal |
| J33.9 | **Nasal polyp, unspecified** |
| J34 | **Other disorders of nose and nasal sinuses** |
|  | Excludes varicose ulcer of nasal septum ( I86.8 ) |
| J34.0 | **Abscess, furuncle and carbuncle of nose** |
|  | Cellulitis |
|  | Necrosis |
|  | Ulceration |
| J34.1 | **Cyst and mucocele of nose and nasal sinus** |
| J34.2 | **Deviated nasal septum** |
|  | Deflection or deviation of septum (nasal)(acquired) |
| J34.3 | **Hypertrophy of nasal turbinates** |
| J34.8 | **Other specified disorders of nose and nasal sinuses** |
|  | Perforation of nasal septum NOS |
|  | Rhinolith |
| J35.1 | **Hypertrophy of tonsils** |
|  | Enlargement of tonsils |
| J35.2 | **Hypertrophy of adenoids** |
|  | Enlargement of adenoids |
| J35.3 | **Hypertrophy of tonsils with hypertrophy of adenoids** |
| J35.8 | **Other chronic diseases of tonsils and adenoids** |
|  | Adenoid vegetations |
|  | Amygdalolith |
|  | Cicatrix of tonsil (and adenoid) |
|  | Tonsillar tag |
|  | Ulcer of tonsil |
| J35.9 | **Chronic disease of tonsils and adenoids, unspecified** |
|  | Disease (chronic) of tonsils and adenoids NOS |
| J36 | **Peritonsillar abscess** |
|  | Abscess of tonsil |
|  | Peritonsillar cellulitis |
|  | Quinsy |
|  | Excludes: retropharyngeal abscess ( J39.0 ) |
|  | tonsillitis: |
|  | · NOS ( J03.9 ) |
|  | · acute ( J03.- ) |
|  | · chronic ( J35.0 ) |
| J38 | **Diseases of vocal cords and larynx, not elsewhere classified** |
|  | Excludes laryngitis: |
|  | · obstructive (acute) ( J05.0 ) |
|  | · ulcerative ( J04.0 ) |
|  | postprocedural subglottic stenosis ( J95.5 ) |
|  | stridor: |
|  | · congenital laryngeal NOS ( P28.8 ) |
|  | · NOS ( R06.1 ) |
| J38.0 | **Paralysis of vocal cords and larynx** |
|  | Laryngoplegia |
|  | Paralysis of glottis |
| J38.1 | **Polyp of vocal cord and larynx** |
|  | Excludes adenomatous polyps ( D14.1 ) |
| J38.2 | **Nodules of vocal cords** |
|  | Chorditis (fibrinous)(nodosa)(tuberosa) |
|  | Singer's nodes |
|  | Teacher's nodes |
| J38.3 | **Other diseases of vocal cords** |
|  | Abscess |
|  | Cellulitis |
|  | Granuloma |
|  | Leukokeratosis |
|  | Leukoplakia |
| J38.4 | **Oedema of larynx** |
|  | Oedema (of): |
|  | · glottis |
|  | · subglottic |
|  | · supraglottic |
|  | Excludes laryngitis: |
|  | · acute obstructive [croup] ( J05.0 ) |
|  | · oedematous ( J04.0 ) |
| J38.5 | **Laryngeal spasm** |
|  | Laryngismus (stridulus) |
| J38.6 | **Stenosis of larynx** |
| J38.7 | **Other diseases of larynx** |
|  | Abscess |
|  | Cellulitis |
|  | Disease NOS |
|  | Necrosis |
|  | Pachyderma |
|  | Perichondritis |
|  | Ulcer |
| J39 | **Other diseases of upper respiratory tract** |
|  | Excludes acute respiratory infection NOS ( J22 ) |
|  | · upper ( J06.9 ) |
|  | upper respiratory inflammation due to chemicals, gases, fumes or vapours ( J68.2 ) |
| J39.0 | **Retropharyngeal and parapharyngeal abscess** |
|  | Peripharyngeal abscess |
|  | Excludes peritonsillar abscess ( J36 ) |
| J39.1 | **Other abscess of pharynx** |
|  | Cellulitis of pharynx |
|  | Nasopharyngeal abscess |
| J39.2 | **Other diseases of pharynx** |
|  | Cyst |
|  | Oedema |
|  | Excludes pharyngitis: |
|  | · chronic ( J31.2 ) |
|  | · ulcerative ( J02.9 ) |
| J39.3 | **Upper respiratory tract hypersensitivity reaction, site unspecified** |
| J39.8 | **Other specified diseases of upper respiratory tract** |
| J39.9 | **Disease of upper respiratory tract, unspecified** |

## Other Disease

Encephalitis

| **ICD-10 Code** | **Description** |
| --- | --- |
| **Those we believe should be included** | |
| A86 | **Unspecified viral encephalitis** |
|  | Viral: |
|  | · encephalomyelitis NOS |
|  | · meningoencephalitis NOS |
| B94.1 | **Sequelae of viral encephalitis** |
| G05.1* | **Encephalitis, myelitis and encephalomyelitis in viral diseases classified elsewhere** |
|  | · influenza ( J09+ , J10.8+ , J11.8+ ) |

Encephalopathy

| **ICD-10 Code** | **Description** |  |  |
| --- | --- | --- | --- |
| **Those we believe should be included** | |  |  |
| J10.8 | **Influenza with other manifestations, other influenza virus identified** |  |  |
|  | Encephalopathy due to influenza | } | other influenza virus identified |
|  | Influenzal: | } |  |
|  | · gastroenteritis | } |  |
|  | · myocarditis (acute) | } |  |
| J11.8 | **Influenza with other manifestations, virus not identified** |  |  |
|  | Encephalopathy due to influenza | } | unspecified or specific virus not identified |
|  | Influenzal: | } |  |
|  | · gastroenteritis | } |  |
|  | · myocarditis (acute) | } |  |

Febrile convulsions

| **ICD-10 Code** | **Description** |
| --- | --- |
| **Those we believe should be included** | |
| R56.0 | **Febrile convulsions** |

Guillain-Barre Syndrome

| **ICD-10 Code** | **Description** |
| --- | --- |
| **Those we believe should be included** | |
| G61.0 | **Guillain-Barré syndrome** |
|  | Acute (post-)infective polyneuritis |

Meningitis

| **ICD-10 Code** | **Description** |
| --- | --- |
| **Those we believe should be included** | |
| G00 | **Bacterial meningitis, not elsewhere classified** |
|  | Includes: arachnoiditis |
|  | leptomeningitis |
|  | meningitis |
|  | pachymeningitis |
|  | Excludes bacterial: |
|  | · meningoencephalitis ( G04.2 ) |
|  | · meningomyelitis ( G04.2 ) |
| G00.0 | **Haemophilus meningitis** |
|  | Meningitis due to Haemophilus influenzae |
| G00.1 | **Pneumococcal meningitis** |
| G00.2 | **Streptococcal meningitis** |
| G00.3 | **Staphylococcal meningitis** |
| G00.8 | **Other bacterial meningitis** |
|  | Meningitis due to: |
|  | · Escherichia coli |
|  | · Friedländer bacillus |
|  | · Klebsiella |
| G00.9 | **Bacterial meningitis, unspecified** |
|  | Meningitis: |
|  | · purulent NOS |
|  | · pyogenic NOS |
|  | · suppurative NOS |
| G03 | **Meningitis due to other and unspecified causes** |
|  | Includes: arachnoiditis |
|  | leptomeningitis |
|  | meningitis |
|  | pachymeningitis |
|  | Excludes: meningoencephalitis ( G04.- ) |
|  | meningomyelitis ( G04.- ) |
| G03.0 | **Nonpyogenic meningitis** |
|  | Nonbacterial meningitis |
| G03.1 | **Chronic meningitis** |
| G03.2 | **Benign recurrent meningitis [Mollaret]** |
| G03.8 | **Meningitis due to other specified causes** |
| G03.9 | **Meningitis, unspecified** |
|  | Arachnoiditis (spinal) NOS |

Myositis

| **ICD-10 Code** | **Description** |
| --- | --- |
| **Those we believe should be included** | |
| M60 | **Myositis** |
| M60.0 | **Infective myositis** |
|  | Tropical pyomyositis |
| M60.1 | **Interstitial myositis** |
| M60.2 | **Foreign body granuloma of soft tissue, not elsewhere classified** |
|  | Excludes foreign body granuloma of skin and subcutaneous tissue ( L92.3 ) |
| M60.8 | **Other myositis** |
| M60.9 | **Myositis, unspecified** |

Reye’s syndrome

| **ICD-10 Code** | **Description** |
| --- | --- |
| **Those we believe should be included** | |
| G93.7 | **Reye's syndrome** |

Sepsis and Septicaemia

| **ICD-10 Code** | **Description** |
| --- | --- |
| **Those we believe should be included** | |
| A40 | **Streptococcal septicaemia** |
|  | Excludes: during labour ( O75.3 ) |
|  | following: |
|  | · abortion or ectopic or molar pregnancy (O03-O07, O08.0) |
|  | · immunization ( T88.0 ) |
|  | · infusion, transfusion or therapeutic injection ( T80.2 ) |
|  | neonatal ( P36.0-P36.1 ) |
|  | postprocedural ( T81.4 ) |
|  | puerperal ( O85 ) |
| A40.0 | **Septicaemia due to streptococcus, group A** |
| A40.1 | **Septicaemia due to streptococcus, group B** |
| A40.2 | **Septicaemia due to streptococcus, group D** |
| A40.3 | **Septicaemia due to Streptococcus pneumoniae** |
|  | Pneumococcal septicaemia |
| A40.8 | **Other streptococcal septicaemia** |
| A40.9 | **Streptococcal septicaemia, unspecified** |
| A41 | **Other septicaemia** |
|  | Excludes: bacteraemia NOS ( A49.9 ) |
|  | during labour ( O75.3 ) |
|  | following: |
|  | · abortion or ectopic or molar pregnancy (O03-O07, O08.0) |
|  | · immunization ( T88.0 ) |
|  | · infusion, transfusion or therapeutic injection ( T80.2 ) |
|  | septicaemia (due to)(in): |
|  | · actinomycotic ( A42.7 ) |
|  | · anthrax ( A22.7 ) |
|  | · candidal ( B37.7 ) |
|  | · Erysipelothrix ( A26.7 ) |
|  | · extraintestinal yersiniosis ( A28.2 ) |
|  | · gonococcal ( A54.8 ) |
|  | · herpesviral ( B00.7 ) |
|  | · listerial ( A32.7 ) |
|  | · meningococcal ( A39.2-A39.4 ) |
|  | · neonatal ( P36.- ) |
|  | · postprocedural ( T81.4 ) |
|  | · puerperal ( O85 ) |
|  | · streptococcal ( A40.- ) |
|  | · tularaemia ( A21.7 ) |
|  | septicaemic: |
|  | · melioidosis ( A24.1 ) |
|  | · plague ( A20.7 ) |
|  | toxic shock syndrome ( A48.3 ) |
| A41.0 | **Septicaemia due to Staphylococcus aureus** |
| A41.1 | **Septicaemia due to other specified staphylococcus** |
|  | Septicaemia due to coagulase-negative staphylococcus |
| A41.2 | **Septicaemia due to unspecified staphylococcus** |
| A41.3 | **Septicaemia due to Haemophilus influenzae** |
| A41.4 | **Septicaemia due to anaerobes** |
|  | Excludes: gas gangrene ( A48.0 ) |
| A41.5 | **Septicaemia due to other Gram-negative organisms** |
|  | Gram-negative septicaemia NOS |
| A41.8 | **Other specified septicaemia** |
| A41.9 | **Septicaemia, unspecified** |
|  | Septic shock |

**Appendix III**

# READ codes for complications in GPRD

Below are the proposed READ codes required to identify complications following influenza in patients in GPRD.

## Acute and Chronic Otitis Media

Acute and Chronic otitis media

| **Read Code** | **Description** |
| --- | --- |
| **Those we believe should be included** | |
| F510.00 | Acute non suppurative otitis media |
| F510000 | Acute otitis media with effusion |
| F510011 | Acute secretory otitis media |
| F510100 | Acute serous otitis media |
| F510200 | Acute mucoid otitis media |
| F510300 | Acute sanguinous otitis media |
| F510z00 | Acute nonsuppurative otitis media NOS |
| F520.00 | Acute suppurative otitis media |
| F520000 | Acute suppurative otitis media tympanic membrane intact |
| F520100 | Acute suppurative otitis media tympanic membrane ruptured |
| F520300 | Acute suppurative otitis media due to disease EC |
| F520z00 | Acute suppurative otitis media NOS |
| F525.00 | Recurrent acute otitis media |
| F526.00 | Acute left otitis media |
| F527.00 | Acute right otitis media |
| F528.00 | Acute bilateral otitis media |
| FyuP000 | [X]Other acute nonsuppurative otitis media |
| F51..00 | Nonsuppurative otitis media + eustachian tube disorders |
| F514.00 | Unspecified nonsuppurative otitis media |
| F514100 | Serous otitis media NOS |
| F514200 | Catarrhal otitis media NOS |
| F514300 | Mucoid otitis media NOS |
| F514z00 | Nonsuppurative otitis media NOS |
| F52..00 | Suppurative and unspecified otitis media |
| F524.00 | Purulent otitis media NOS |
| F524000 | Bilateral suppurative otitis media |
| F52z.00 | Otitis media NOS |
| FyuP400 | [X]Otitis media in viral diseases classified elsewhere |
| F511.00 | Chronic otitis media with effusion, serous |
| F511.11 | Chronic secretory otitis media, serous |
| F511100 | Serosanguinous chronic otitis media |
| F511z00 | Chronic serous otitis media NOS |
| F512.00 | Chronic otitis media with effusion, mucoid |
| F512.12 | Chronic secretory otitis media, mucoid |
| F512100 | Mucosanguinous chronic otitis media |
| F512z00 | Chronic mucoid otitis media NOS |
| F513.00 | Chronic otitis media with effusion, other |
| F513000 | Chronic allergic otitis media |
| F513100 | Chronic otitis media with effusion, purulent |
| F513111 | Chronic secretory otitis media, purulent |
| F513z00 | Other chronic nonsuppurative otitis media NOS |
| F518.00 | Chronic otitis media with effusion, unspecified |
| F521.00 | Chronic suppurative otitis media, tubotympanic |
| F522.00 | Chronic suppurative otitis media, atticoantral |
| F523.00 | Chronic suppurative otitis media NOS |
| F541.00 | Chronic myringitis without mention of otitis media |
| FyuP100 | [X]Other chronic nonsuppurative otitis media |
| FyuP200 | [X]Other chronic suppurative otitis media |

## Cardiovascular Disease

Acute myocardial infarction

| **Read Code** | **Description** |
| --- | --- |
| **Those we believe should be included** | |
| G30..00 | Acute myocardial infarction |
| G30..11 | Attack - heart |
| G30..12 | Coronary thrombosis |
| G30..13 | Cardiac rupture following myocardial infarction (MI) |
| G30..14 | Heart attack |
| G30..15 | MI - acute myocardial infarction |
| G30..16 | Thrombosis - coronary |
| G30..17 | Silent myocardial infarction |
| G300.00 | Acute anterolateral infarction |
| G301.00 | Other specified anterior myocardial infarction |
| G301000 | Acute anteroapical infarction |
| G301100 | Acute anteroseptal infarction |
| G301z00 | Anterior myocardial infarction NOS |
| G302.00 | Acute inferolateral infarction |
| G303.00 | Acute inferoposterior infarction |
| G304.00 | Posterior myocardial infarction NOS |
| G305.00 | Lateral myocardial infarction NOS |
| G306.00 | True posterior myocardial infarction |
| G307.00 | Acute subendocardial infarction |
| G307000 | Acute non-Q wave infarction |
| G307100 | Acute non-ST segment elevation myocardial infarction |
| G308.00 | Inferior myocardial infarction NOS |
| G309.00 | Acute Q-wave infarct |
| G30A.00 | Mural thrombosis |
| G30B.00 | Acute posterolateral myocardial infarction |
| G30X.00 | Acute transmural myocardial infarction of unspecif site |
| G30X000 | Acute ST segment elevation myocardial infarction |
| G30y.00 | Other acute myocardial infarction |
| G30y000 | Acute atrial infarction |
| G30y100 | Acute papillary muscle infarction |
| G30y200 | Acute septal infarction |
| G30yz00 | Other acute myocardial infarction NOS |
| G30z.00 | Acute myocardial infarction NOS |
| G35..00 | Subsequent myocardial infarction |
| G350.00 | Subsequent myocardial infarction of anterior wall |
| G351.00 | Subsequent myocardial infarction of inferior wall |
| G353.00 | Subsequent myocardial infarction of other sites |
| G35X.00 | Subsequent myocardial infarction of unspecified site |

Heart failure

| **Read Code** | **Description** |
| --- | --- |
| **Those we believe should be included** | |
| 1J60.00 | Suspected heart failure |
| 1O1..00 | Heart failure confirmed |
| 8H2S.00 | Admit heart failure emergency |
| G232.00 | Hypertensive heart&renal dis wth (congestive) heart failure |
| G58..00 | Heart failure |
| G58..11 | Cardiac failure |
| G580.00 | Congestive heart failure |
| G580.11 | Congestive cardiac failure |
| G580.12 | Right heart failure |
| G580000 | Acute congestive heart failure |
| G580100 | Chronic congestive heart failure |
| G580200 | Decompensated cardiac failure |
| G580300 | Compensated cardiac failure |
| G582.00 | Acute heart failure |
| G58z.00 | Heart failure NOS |
| G58z.12 | Cardiac failure NOS |
| Q490.00 | Neonatal cardiac failure |
| SP11111 | Heart failure as a complication of care |

Hypertension

| **Read Code** | **Description** |
| --- | --- |
| **Those we believe should be included** | |
| 1JD..00 | Suspected hypertension |
| G20..00 | Essential hypertension |
| G200.00 | Malignant essential hypertension |
| G201.00 | Benign essential hypertension |
| G202.00 | Systolic hypertension |
| G20z.00 | Essential hypertension NOS |
| G20z.11 | Hypertension NOS |
| G22z.11 | Renal hypertension |
| G24..00 | Secondary hypertension |
| G240.00 | Secondary malignant hypertension |
| G240000 | Secondary malignant renovascular hypertension |
| G240z00 | Secondary malignant hypertension NOS |
| G241.00 | Secondary benign hypertension |
| G241000 | Secondary benign renovascular hypertension |
| G241z00 | Secondary benign hypertension NOS |
| G24z.00 | Secondary hypertension NOS |
| G24z000 | Secondary renovascular hypertension NOS |
| G24zz00 | Secondary hypertension NOS |
| G410.00 | Primary pulmonary hypertension |
| G41y000 | Secondary pulmonary hypertension |
| G8y3.00 | Chronic peripheral venous hypertension |
| Gyu2000 | [X]Other secondary hypertension |
| Gyu2100 | [X]Hypertension secondary to other renal disorders |
| J623.00 | Portal hypertension |
| Q492.00 | Neonatal hypertension |

Myocarditis

| **Read Code** | **Description** |
| --- | --- |
| **Those we believe should be included** | |
| G012.00 | Acute rheumatic myocarditis |
| G1y0.00 | Rheumatic myocarditis |
| G52..00 | Acute myocarditis |
| G520.00 | Acute myocarditis in diseases EC |
| G520000 | Acute aseptic myocarditis of the newborn |
| G520300 | Acute myocarditis - influenzal |
| G520z00 | Acute myocarditis in diseases EC, NOS |
| G52y.00 | Other acute myocarditis |
| G52y000 | Acute myocarditis, unspecified |
| G52yz00 | Other acute myocarditis NOS |
| G52z.00 | Acute myocarditis NOS |
| G5y0.00 | Myocarditis NOS |
| G5y7.00 | Sarcoid myocarditis |
| G5y8.00 | Rheumatoid myocarditis |
| Gyu5F00 | [X]Other acute myocarditis |
| Gyu5G00 | [X]Acute myocarditis, unspecified |
| Gyu5J00 | [X]Myocarditis in viral diseases classified elsewhere |
| Gyu5L00 | [X]Myocarditis in other diseases classified elsewhere |
| Gyu5K00 | [X]Myocarditis in other infectious+parasitic diseases CE |

## Central Nervous System Disease

Epilepsy

| **Read Code** | **Description** |
| --- | --- |
| **Those we believe should be included** | |
| F1...00 | Hereditary and degenerative diseases of the CNS |
| F250.00 | Generalised nonconvulsive epilepsy |
| F250000 | Petit mal (minor) epilepsy |
| F250011 | Epileptic absences |
| F250100 | Pykno-epilepsy |
| F250200 | Epileptic seizures - atonic |
| F250300 | Epileptic seizures - akinetic |
| F250400 | Juvenile absence epilepsy |
| F250500 | Lennox-Gastaut syndrome |
| F250y00 | Other specified generalised nonconvulsive epilepsy |
| F250z00 | Generalised nonconvulsive epilepsy NOS |
| F251.00 | Generalised convulsive epilepsy |
| F251000 | Grand mal (major) epilepsy |
| F251011 | Tonic-clonic epilepsy |
| F251100 | Neonatal myoclonic epilepsy |
| F251111 | Otohara syndrome |
| F251200 | Epileptic seizures - clonic |
| F251300 | Epileptic seizures - myoclonic |
| F251400 | Epileptic seizures - tonic |
| F251500 | Tonic-clonic epilepsy |
| F251600 | Grand mal seizure |
| F251y00 | Other specified generalised convulsive epilepsy |
| F251z00 | Generalised convulsive epilepsy NOS |
| F252.00 | Petit mal status |
| F253.00 | Grand mal status |
| F253.11 | Status epilepticus |
| F254.00 | Partial epilepsy with impairment of consciousness |
| F254000 | Temporal lobe epilepsy |
| F254100 | Psychomotor epilepsy |
| F254200 | Psychosensory epilepsy |
| F254300 | Limbic system epilepsy |
| F254400 | Epileptic automatism |
| F254500 | Complex partial epileptic seizure |
| F254z00 | Partial epilepsy with impairment of consciousness NOS |
| F255.00 | Partial epilepsy without impairment of consciousness |
| F255000 | Jacksonian, focal or motor epilepsy |
| F255011 | Focal epilepsy |
| F255012 | Motor epilepsy |
| F255100 | Sensory induced epilepsy |
| F255200 | Somatosensory epilepsy |
| F255300 | Visceral reflex epilepsy |
| F255311 | Partial epilepsy with autonomic symptoms |
| F255400 | Visual reflex epilepsy |
| F255500 | Unilateral epilepsy |
| F255600 | Simple partial epileptic seizure |
| F255y00 | Partial epilepsy without impairment of consciousness OS |
| F255z00 | Partial epilepsy without impairment of consciousness NOS |
| F256.00 | Infantile spasms |
| F256.11 | Lightning spasms |
| F256.12 | West syndrome |
| F256000 | Hypsarrhythmia |
| F256100 | Salaam attacks |
| F256z00 | Infantile spasms NOS |
| F257.00 | Kojevnikov's epilepsy |
| F258.00 | Post-ictal state |
| F259.00 | Early infant epileptic encephalopathy wth suppression bursts |
| F259.11 | Ohtahara syndrome |
| F25A.00 | Juvenile myoclonic epilepsy |
| F25B.00 | Alcohol-induced epilepsy |
| F25C.00 | Drug-induced epilepsy |
| F25D.00 | Menstrual epilepsy |
| F25E.00 | Stress-induced epilepsy |
| F25F.00 | Photosensitive epilepsy |
| F25X.00 | Status epilepticus, unspecified |
| F25y.00 | Other forms of epilepsy |
| F25y000 | Cursive (running) epilepsy |
| F25y100 | Gelastic epilepsy |
| F25y200 | Locl-rlt(foc)(part)idiop epilep&epilptic syn seiz locl onset |
| F25y300 | Complex partial status epilepticus |
| F25y400 | Benign Rolandic epilepsy |
| F25yz00 | Other forms of epilepsy NOS |
| F25z.00 | Epilepsy NOS |
| F25z.11 | Fit (in known epileptic) NOS |

Parkinson’s Disease

| **Read Code** | **Description** |
| --- | --- |
| **Those we believe should be included** | |
| F12..00 | Parkinson's disease |
| F12z.00 | Parkinson's disease NOS |

Psychosis

| **Read Code** | **Description** |
| --- | --- |
| **Those we believe should be included** | |
| E00y.00 | Other senile and presenile organic psychoses |
| E00y.11 | Presbyophrenic psychosis |
| E00z.00 | Senile or presenile psychoses NOS |
| E03..00 | Transient organic psychoses |
| E03y.00 | Other transient organic psychoses |
| E03yz00 | Other transient organic psychoses NOS |
| E03z.00 | Transient organic psychoses NOS |
| E04..00 | Other chronic organic psychoses |
| E04y.00 | Other specified chronic organic psychoses |
| E04z.00 | Chronic organic psychosis NOS |
| E0y..00 | Other specified organic psychoses |
| E0z..00 | Organic psychoses NOS |
| Eu02z12 | [X] Presenile psychosis NOS |
| Eu02z15 | [X] Senile psychosis NOS |
| Eu04.13 | [X]Acute / subacute infective psychosis |
| Eu0z.11 | [X]Organic psychosis NOS |
| Eu0z.12 | [X]Symptomatic psychosis NOS |
| E11..00 | Affective psychoses |
| E11..11 | Bipolar psychoses |
| E11..12 | Depressive psychoses |
| E11..13 | Manic psychoses |
| E110.11 | Hypomanic psychoses |
| E110300 | Single manic episode, severe without mention of psychosis |
| E110400 | Single manic episode, severe, with psychosis |
| E111300 | Recurrent manic episodes, severe without mention psychosis |
| E111400 | Recurrent manic episodes, severe, with psychosis |
| E112300 | Single major depressive episode, severe, without psychosis |
| E112400 | Single major depressive episode, severe, with psychosis |
| E113300 | Recurrent major depressive episodes, severe, no psychosis |
| E113400 | Recurrent major depressive episodes, severe, with psychosis |
| E114300 | Bipolar affect disord, currently manic, severe, no psychosis |
| E114400 | Bipolar affect disord, currently manic,severe with psychosis |
| E115300 | Bipolar affect disord, now depressed, severe, no psychosis |
| E115400 | Bipolar affect disord, now depressed, severe with psychosis |
| E116300 | Mixed bipolar affective disorder, severe, without psychosis |
| E116400 | Mixed bipolar affective disorder, severe, with psychosis |
| E117300 | Unspecified bipolar affective disorder, severe, no psychosis |
| E117400 | Unspecified bipolar affective disorder,severe with psychosis |
| E11y.00 | Other and unspecified manic-depressive psychoses |
| E11y000 | Unspecified manic-depressive psychoses |
| E11y300 | Other mixed manic-depressive psychoses |
| E11yz00 | Other and unspecified manic-depressive psychoses NOS |
| E11z.00 | Other and unspecified affective psychoses |
| E11z000 | Unspecified affective psychoses NOS |
| E11zz00 | Other affective psychosis NOS |
| E121.00 | Chronic paranoid psychosis |
| E12z.00 | Paranoid psychosis NOS |
| E13..00 | Other nonorganic psychoses |
| E13..11 | Reactive psychoses |
| E130.00 | Reactive depressive psychosis |
| E131.00 | Acute hysterical psychosis |
| E134.00 | Psychogenic paranoid psychosis |
| E13y.00 | Other reactive psychoses |
| E13y100 | Brief reactive psychosis |
| E13yz00 | Other reactive psychoses NOS |
| E13z.00 | Nonorganic psychosis NOS |
| E14..00 | Psychoses with origin in childhood |
| E141.00 | Disintegrative psychosis |
| E141000 | Active disintegrative psychoses |
| E141100 | Residual disintegrative psychoses |
| E141z00 | Disintegrative psychosis NOS |
| E14y.00 | Other childhood psychoses |
| E14y000 | Atypical childhood psychoses |
| E14y100 | Borderline psychosis of childhood |
| E14yz00 | Other childhood psychoses NOS |
| E14z.00 | Child psychosis NOS |
| E1y..00 | Other specified non-organic psychoses |
| E1z..00 | Non-organic psychosis NOS |
| Eu22011 | [X]Paranoid psychosis |
| Eu23012 | [X]Cycloid psychosis |
| Eu23112 | [X]Cycloid psychosis with symptoms of schizophrenia |
| Eu23312 | [X]Psychogenic paranoid psychosis |
| Eu23z11 | [X]Brief reactive psychosis NOS |
| Eu23z12 | [X]Reactive psychosis |
| Eu25011 | [X]Schizoaffective psychosis, manic type |
| Eu25012 | [X]Schizophreniform psychosis, manic type |
| Eu25111 | [X]Schizoaffective psychosis, depressive type |
| Eu25112 | [X]Schizophreniform psychosis, depressive type |
| Eu25212 | [X]Mixed schizophrenic and affective psychosis |
| Eu25z11 | [X]Schizoaffective psychosis NOS |
| Eu2y.11 | [X]Chronic hallucinatory psychosis |
| Eu2z.00 | [X]Unspecified nonorganic psychosis |
| Eu2z.11 | [X]Psychosis NOS |
| Eu31.12 | [X]Manic-depressive psychosis |
| Eu32312 | [X]Single episode of psychogenic depressive psychosis |
| Eu32314 | [X]Single episode of reactive depressive psychosis |
| Eu33213 | [X]Manic-depress psychosis,depressd,no psychotic symptoms |
| Eu33312 | [X]Manic-depress psychosis,depressed type+psychotic symptoms |
| Eu33314 | [X]Recurr severe episodes/psychogenic depressive psychosis |
| Eu33316 | [X]Recurrent severe episodes/reactive depressive psychosis |
| Eu3z.11 | [X]Affective psychosis NOS |
| Eu44.14 | [X]Hysterical psychosis |
| Eu53111 | [X]Puerperal psychosis NOS |
| Eu84013 | [X]Infantile psychosis |
| Eu84111 | [X]Atypical childhood psychosis |
| Eu84312 | [X]Disintegrative psychosis |
| Eu84314 | [X]Symbiotic psychosis |

## Cerebrovascular Disease

Cerebrovascular disease

| **Read Code** | **Description** |
| --- | --- |
| **Those we believe should be included** | |
| 1JA1.00 | Suspected cerebrovascular disease |
| G6...00 | Cerebrovascular disease |
| G61..11 | CVA - cerebrovascular accid due to intracerebral haemorrhage |
| G61..12 | Stroke due to intracerebral haemorrhage |
| G64..13 | Stroke due to cerebral arterial occlusion |
| G65..12 | Transient ischaemic attack |
| G66..00 | Stroke and cerebrovascular accident unspecified |
| G66..12 | Stroke unspecified |
| G66..13 | CVA - Cerebrovascular accident unspecified |
| G663.00 | Brain stem stroke syndrome |
| G664.00 | Cerebellar stroke syndrome |
| G67..00 | Other cerebrovascular disease |
| G671.00 | Generalised ischaemic cerebrovascular disease NOS |
| G671000 | Acute cerebrovascular insufficiency NOS |
| G671z00 | Generalised ischaemic cerebrovascular disease NOS |
| G67y.00 | Other cerebrovascular disease OS |
| G67z.00 | Other cerebrovascular disease NOS |
| G68..00 | Late effects of cerebrovascular disease |
| G68W.00 | Sequelae/other + unspecified cerebrovascular diseases |
| G68X.00 | Sequelae of stroke,not specfd as h'morrhage or infarction |
| G6y..00 | Other specified cerebrovascular disease |
| G6z..00 | Cerebrovascular disease NOS |
| Gyu6.00 | [X]Cerebrovascular diseases |
| Gyu6700 | [X]Other specified cerebrovascular diseases |
| Gyu6A00 | [X]Other cerebrovascular disorders in diseases CE |
| Gyu6C00 | [X]Sequelae of stroke,not specfd as h'morrhage or infarction |
| Gyu6D00 | [X]Sequelae/other + unspecified cerebrovascular diseases |
| 8HBJ.00 | Stroke / transient ischaemic attack referral |
| 8HHM.00 | Ref to multidisciplinary stroke function improvement service |
| 8HTQ.00 | Referral to stroke clinic |
| L440.00 | Cerebrovascular disorders in the puerperium |
| L440.11 | CVA - cerebrovascular accident in the puerperium |
| L440.12 | Stroke in the puerperium |
| L440000 | Puerperal cerebrovascular disorder unspecified |
| L440100 | Puerperal cerebrovascular disorder - delivered |
| L440z00 | Puerperal cerebrovascular disorder NOS |
| P7y0.00 | Cerebrovascular system anomalies |
| P7y0y00 | Other specified cerebrovascular anomaly |
| P7y0z00 | Cerebrovascular system anomaly NOS |

## Diabetes

Diabetes

| **Read Code** | **Description** |
| --- | --- |
| **Those we believe should be included** | |
| 66AJ.11 | Unstable diabetes |
| 66AJ100 | Brittle diabetes |
| 8H2J.00 | Admit diabetic emergency |
| 8H3O.00 | Non-urgent diabetic admission |
| C10..00 | Diabetes mellitus |
| C100.00 | Diabetes mellitus with no mention of complication |
| C100000 | Diabetes mellitus, juvenile type, no mention of complication |
| C100011 | Insulin dependent diabetes mellitus |
| C100100 | Diabetes mellitus, adult onset, no mention of complication |
| C100111 | Maturity onset diabetes |
| C100112 | Non-insulin dependent diabetes mellitus |
| C100z00 | Diabetes mellitus NOS with no mention of complication |
| C101.00 | Diabetes mellitus with ketoacidosis |
| C101000 | Diabetes mellitus, juvenile type, with ketoacidosis |
| C101100 | Diabetes mellitus, adult onset, with ketoacidosis |
| C101y00 | Other specified diabetes mellitus with ketoacidosis |
| C101z00 | Diabetes mellitus NOS with ketoacidosis |
| C102.00 | Diabetes mellitus with hyperosmolar coma |
| C102000 | Diabetes mellitus, juvenile type, with hyperosmolar coma |
| C102100 | Diabetes mellitus, adult onset, with hyperosmolar coma |
| C102z00 | Diabetes mellitus NOS with hyperosmolar coma |
| C103.00 | Diabetes mellitus with ketoacidotic coma |
| C103000 | Diabetes mellitus, juvenile type, with ketoacidotic coma |
| C103100 | Diabetes mellitus, adult onset, with ketoacidotic coma |
| C103y00 | Other specified diabetes mellitus with coma |
| C103z00 | Diabetes mellitus NOS with ketoacidotic coma |
| C104.00 | Diabetes mellitus with renal manifestation |
| C104.11 | Diabetic nephropathy |
| C104000 | Diabetes mellitus, juvenile type, with renal manifestation |
| C104100 | Diabetes mellitus, adult onset, with renal manifestation |
| C104y00 | Other specified diabetes mellitus with renal complications |
| C104z00 | Diabetes mellitis with nephropathy NOS |
| C105.00 | Diabetes mellitus with ophthalmic manifestation |
| C105000 | Diabetes mellitus, juvenile type, + ophthalmic manifestation |
| C105100 | Diabetes mellitus, adult onset, + ophthalmic manifestation |
| C105y00 | Other specified diabetes mellitus with ophthalmic complicatn |
| C105z00 | Diabetes mellitus NOS with ophthalmic manifestation |
| C106.00 | Diabetes mellitus with neurological manifestation |
| C106.11 | Diabetic amyotrophy |
| C106.12 | Diabetes mellitus with neuropathy |
| C106.13 | Diabetes mellitus with polyneuropathy |
| C106000 | Diabetes mellitus, juvenile, + neurological manifestation |
| C106100 | Diabetes mellitus, adult onset, + neurological manifestation |
| C106y00 | Other specified diabetes mellitus with neurological comps |
| C106z00 | Diabetes mellitus NOS with neurological manifestation |
| C107.00 | Diabetes mellitus with peripheral circulatory disorder |
| C107.11 | Diabetes mellitus with gangrene |
| C107.12 | Diabetes with gangrene |
| C107000 | Diabetes mellitus, juvenile +peripheral circulatory disorder |
| C107100 | Diabetes mellitus, adult, + peripheral circulatory disorder |
| C107200 | Diabetes mellitus, adult with gangrene |
| C107y00 | Other specified diabetes mellitus with periph circ comps |
| C107z00 | Diabetes mellitus NOS with peripheral circulatory disorder |
| C108.00 | Insulin dependent diabetes mellitus |
| C108.11 | IDDM-Insulin dependent diabetes mellitus |
| C108.12 | Type 1 diabetes mellitus |
| C108.13 | Type I diabetes mellitus |
| C108000 | Insulin-dependent diabetes mellitus with renal complications |
| C108011 | Type I diabetes mellitus with renal complications |
| C108012 | Type 1 diabetes mellitus with renal complications |
| C108100 | Insulin-dependent diabetes mellitus with ophthalmic comps |
| C108111 | Type I diabetes mellitus with ophthalmic complications |
| C108112 | Type 1 diabetes mellitus with ophthalmic complications |
| C108200 | Insulin-dependent diabetes mellitus with neurological comps |
| C108211 | Type I diabetes mellitus with neurological complications |
| C108212 | Type 1 diabetes mellitus with neurological complications |
| C108300 | Insulin dependent diabetes mellitus with multiple complicatn |
| C108311 | Type I diabetes mellitus with multiple complications |
| C108312 | Type 1 diabetes mellitus with multiple complications |
| C108400 | Unstable insulin dependent diabetes mellitus |
| C108411 | Unstable type I diabetes mellitus |
| C108412 | Unstable type 1 diabetes mellitus |
| C108500 | Insulin dependent diabetes mellitus with ulcer |
| C108511 | Type I diabetes mellitus with ulcer |
| C108512 | Type 1 diabetes mellitus with ulcer |
| C108600 | Insulin dependent diabetes mellitus with gangrene |
| C108611 | Type I diabetes mellitus with gangrene |
| C108612 | Type 1 diabetes mellitus with gangrene |
| C108700 | Insulin dependent diabetes mellitus with retinopathy |
| C108711 | Type I diabetes mellitus with retinopathy |
| C108712 | Type 1 diabetes mellitus with retinopathy |
| C108800 | Insulin dependent diabetes mellitus - poor control |
| C108811 | Type I diabetes mellitus - poor control |
| C108812 | Type 1 diabetes mellitus - poor control |
| C108900 | Insulin dependent diabetes maturity onset |
| C108911 | Type I diabetes mellitus maturity onset |
| C108912 | Type 1 diabetes mellitus maturity onset |
| C108A00 | Insulin-dependent diabetes without complication |
| C108A11 | Type I diabetes mellitus without complication |
| C108A12 | Type 1 diabetes mellitus without complication |
| C108B00 | Insulin dependent diabetes mellitus with mononeuropathy |
| C108B11 | Type I diabetes mellitus with mononeuropathy |
| C108B12 | Type 1 diabetes mellitus with mononeuropathy |
| C108C00 | Insulin dependent diabetes mellitus with polyneuropathy |
| C108C11 | Type I diabetes mellitus with polyneuropathy |
| C108C12 | Type 1 diabetes mellitus with polyneuropathy |
| C108D00 | Insulin dependent diabetes mellitus with nephropathy |
| C108D11 | Type I diabetes mellitus with nephropathy |
| C108D12 | Type 1 diabetes mellitus with nephropathy |
| C108E00 | Insulin dependent diabetes mellitus with hypoglycaemic coma |
| C108E11 | Type I diabetes mellitus with hypoglycaemic coma |
| C108E12 | Type 1 diabetes mellitus with hypoglycaemic coma |
| C108F00 | Insulin dependent diabetes mellitus with diabetic cataract |
| C108F11 | Type I diabetes mellitus with diabetic cataract |
| C108F12 | Type 1 diabetes mellitus with diabetic cataract |
| C108G11 | Type I diabetes mellitus with peripheral angiopathy |
| C108G12 | Type 1 diabetes mellitus with peripheral angiopathy |
| C108H00 | Insulin dependent diabetes mellitus with arthropathy |
| C108H11 | Type I diabetes mellitus with arthropathy |
| C108H12 | Type 1 diabetes mellitus with arthropathy |
| C108J11 | Type I diabetes mellitus with neuropathic arthropathy |
| C108J12 | Type 1 diabetes mellitus with neuropathic arthropathy |
| C108y00 | Other specified diabetes mellitus with multiple comps |
| C108z00 | Unspecified diabetes mellitus with multiple complications |
| C109.00 | Non-insulin dependent diabetes mellitus |
| C109.11 | NIDDM - Non-insulin dependent diabetes mellitus |
| C109.12 | Type 2 diabetes mellitus |
| C109.13 | Type II diabetes mellitus |
| C109000 | Non-insulin-dependent diabetes mellitus with renal comps |
| C109011 | Type II diabetes mellitus with renal complications |
| C109012 | Type 2 diabetes mellitus with renal complications |
| C109100 | Non-insulin-dependent diabetes mellitus with ophthalm comps |
| C109111 | Type II diabetes mellitus with ophthalmic complications |
| C109112 | Type 2 diabetes mellitus with ophthalmic complications |
| C109200 | Non-insulin-dependent diabetes mellitus with neuro comps |
| C109211 | Type II diabetes mellitus with neurological complications |
| C109212 | Type 2 diabetes mellitus with neurological complications |
| C109300 | Non-insulin-dependent diabetes mellitus with multiple comps |
| C109311 | Type II diabetes mellitus with multiple complications |
| C109312 | Type 2 diabetes mellitus with multiple complications |
| C109400 | Non-insulin dependent diabetes mellitus with ulcer |
| C109411 | Type II diabetes mellitus with ulcer |
| C109412 | Type 2 diabetes mellitus with ulcer |
| C109500 | Non-insulin dependent diabetes mellitus with gangrene |
| C109511 | Type II diabetes mellitus with gangrene |
| C109512 | Type 2 diabetes mellitus with gangrene |
| C109600 | Non-insulin-dependent diabetes mellitus with retinopathy |
| C109611 | Type II diabetes mellitus with retinopathy |
| C109612 | Type 2 diabetes mellitus with retinopathy |
| C109700 | Non-insulin dependent diabetes mellitus - poor control |
| C109711 | Type II diabetes mellitus - poor control |
| C109712 | Type 2 diabetes mellitus - poor control |
| C109900 | Non-insulin-dependent diabetes mellitus without complication |
| C109911 | Type II diabetes mellitus without complication |
| C109912 | Type 2 diabetes mellitus without complication |
| C109A00 | Non-insulin dependent diabetes mellitus with mononeuropathy |
| C109A11 | Type II diabetes mellitus with mononeuropathy |
| C109A12 | Type 2 diabetes mellitus with mononeuropathy |
| C109B00 | Non-insulin dependent diabetes mellitus with polyneuropathy |
| C109B11 | Type II diabetes mellitus with polyneuropathy |
| C109B12 | Type 2 diabetes mellitus with polyneuropathy |
| C109C00 | Non-insulin dependent diabetes mellitus with nephropathy |
| C109C11 | Type II diabetes mellitus with nephropathy |
| C109C12 | Type 2 diabetes mellitus with nephropathy |
| C109D00 | Non-insulin dependent diabetes mellitus with hypoglyca coma |
| C109D11 | Type II diabetes mellitus with hypoglycaemic coma |
| C109D12 | Type 2 diabetes mellitus with hypoglycaemic coma |
| C109E00 | Non-insulin depend diabetes mellitus with diabetic cataract |
| C109E11 | Type II diabetes mellitus with diabetic cataract |
| C109E12 | Type 2 diabetes mellitus with diabetic cataract |
| C109F11 | Type II diabetes mellitus with peripheral angiopathy |
| C109F12 | Type 2 diabetes mellitus with peripheral angiopathy |
| C109G00 | Non-insulin dependent diabetes mellitus with arthropathy |
| C109G11 | Type II diabetes mellitus with arthropathy |
| C109G12 | Type 2 diabetes mellitus with arthropathy |
| C109H11 | Type II diabetes mellitus with neuropathic arthropathy |
| C109H12 | Type 2 diabetes mellitus with neuropathic arthropathy |
| C109J00 | Insulin treated Type 2 diabetes mellitus |
| C109J11 | Insulin treated non-insulin dependent diabetes mellitus |
| C109J12 | Insulin treated Type II diabetes mellitus |
| C109K00 | Hyperosmolar non-ketotic state in type 2 diabetes mellitus |
| C10C.00 | Diabetes mellitus autosomal dominant |
| C10C.11 | Maturity onset diabetes in youth |
| C10C.12 | Maturity onset diabetes in youth type 1 |
| C10D.00 | Diabetes mellitus autosomal dominant type 2 |
| C10D.11 | Maturity onset diabetes in youth type 2 |
| C10E.00 | Type 1 diabetes mellitus |
| C10E.11 | Type I diabetes mellitus |
| C10E.12 | Insulin dependent diabetes mellitus |
| C10E000 | Type 1 diabetes mellitus with renal complications |
| C10E011 | Type I diabetes mellitus with renal complications |
| C10E012 | Insulin-dependent diabetes mellitus with renal complications |
| C10E100 | Type 1 diabetes mellitus with ophthalmic complications |
| C10E111 | Type I diabetes mellitus with ophthalmic complications |
| C10E112 | Insulin-dependent diabetes mellitus with ophthalmic comps |
| C10E200 | Type 1 diabetes mellitus with neurological complications |
| C10E211 | Type I diabetes mellitus with neurological complications |
| C10E212 | Insulin-dependent diabetes mellitus with neurological comps |
| C10E300 | Type 1 diabetes mellitus with multiple complications |
| C10E311 | Type I diabetes mellitus with multiple complications |
| C10E312 | Insulin dependent diabetes mellitus with multiple complicat |
| C10E400 | Unstable type 1 diabetes mellitus |
| C10E411 | Unstable type I diabetes mellitus |
| C10E412 | Unstable insulin dependent diabetes mellitus |
| C10E500 | Type 1 diabetes mellitus with ulcer |
| C10E511 | Type I diabetes mellitus with ulcer |
| C10E512 | Insulin dependent diabetes mellitus with ulcer |
| C10E600 | Type 1 diabetes mellitus with gangrene |
| C10E611 | Type I diabetes mellitus with gangrene |
| C10E612 | Insulin dependent diabetes mellitus with gangrene |
| C10E700 | Type 1 diabetes mellitus with retinopathy |
| C10E711 | Type I diabetes mellitus with retinopathy |
| C10E712 | Insulin dependent diabetes mellitus with retinopathy |
| C10E800 | Type 1 diabetes mellitus - poor control |
| C10E811 | Type I diabetes mellitus - poor control |
| C10E812 | Insulin dependent diabetes mellitus - poor control |
| C10E900 | Type 1 diabetes mellitus maturity onset |
| C10E911 | Type I diabetes mellitus maturity onset |
| C10E912 | Insulin dependent diabetes maturity onset |
| C10EA00 | Type 1 diabetes mellitus without complication |
| C10EA11 | Type I diabetes mellitus without complication |
| C10EA12 | Insulin-dependent diabetes without complication |
| C10EB00 | Type 1 diabetes mellitus with mononeuropathy |
| C10EB11 | Type I diabetes mellitus with mononeuropathy |
| C10EB12 | Insulin dependent diabetes mellitus with mononeuropathy |
| C10EC00 | Type 1 diabetes mellitus with polyneuropathy |
| C10EC11 | Type I diabetes mellitus with polyneuropathy |
| C10EC12 | Insulin dependent diabetes mellitus with polyneuropathy |
| C10ED00 | Type 1 diabetes mellitus with nephropathy |
| C10ED11 | Type I diabetes mellitus with nephropathy |
| C10ED12 | Insulin dependent diabetes mellitus with nephropathy |
| C10EE00 | Type 1 diabetes mellitus with hypoglycaemic coma |
| C10EE11 | Type I diabetes mellitus with hypoglycaemic coma |
| C10EE12 | Insulin dependent diabetes mellitus with hypoglycaemic coma |
| C10EF00 | Type 1 diabetes mellitus with diabetic cataract |
| C10EF11 | Type I diabetes mellitus with diabetic cataract |
| C10EF12 | Insulin dependent diabetes mellitus with diabetic cataract |
| C10EG00 | Type 1 diabetes mellitus with peripheral angiopathy |
| C10EG11 | Type I diabetes mellitus with peripheral angiopathy |
| C10EH00 | Type 1 diabetes mellitus with arthropathy |
| C10EH11 | Type I diabetes mellitus with arthropathy |
| C10EH12 | Insulin dependent diabetes mellitus with arthropathy |
| C10EJ00 | Type 1 diabetes mellitus with neuropathic arthropathy |
| C10EJ11 | Type I diabetes mellitus with neuropathic arthropathy |
| C10EK00 | Type 1 diabetes mellitus with persistent proteinuria |
| C10EK11 | Type I diabetes mellitus with persistent proteinuria |
| C10EL00 | Type 1 diabetes mellitus with persistent microalbuminuria |
| C10EL11 | Type I diabetes mellitus with persistent microalbuminuria |
| C10EM00 | Type 1 diabetes mellitus with ketoacidosis |
| C10EM11 | Type I diabetes mellitus with ketoacidosis |
| C10EN00 | Type 1 diabetes mellitus with ketoacidotic coma |
| C10EN11 | Type I diabetes mellitus with ketoacidotic coma |
| C10EP00 | Type 1 diabetes mellitus with exudative maculopathy |
| C10EP11 | Type I diabetes mellitus with exudative maculopathy |
| C10EQ00 | Type 1 diabetes mellitus with gastroparesis |
| C10F.00 | Type 2 diabetes mellitus |
| C10F.11 | Type II diabetes mellitus |
| C10F000 | Type 2 diabetes mellitus with renal complications |
| C10F011 | Type II diabetes mellitus with renal complications |
| C10F100 | Type 2 diabetes mellitus with ophthalmic complications |
| C10F111 | Type II diabetes mellitus with ophthalmic complications |
| C10F200 | Type 2 diabetes mellitus with neurological complications |
| C10F211 | Type II diabetes mellitus with neurological complications |
| C10F300 | Type 2 diabetes mellitus with multiple complications |
| C10F311 | Type II diabetes mellitus with multiple complications |
| C10F400 | Type 2 diabetes mellitus with ulcer |
| C10F411 | Type II diabetes mellitus with ulcer |
| C10F500 | Type 2 diabetes mellitus with gangrene |
| C10F511 | Type II diabetes mellitus with gangrene |
| C10F600 | Type 2 diabetes mellitus with retinopathy |
| C10F611 | Type II diabetes mellitus with retinopathy |
| C10F700 | Type 2 diabetes mellitus - poor control |
| C10F711 | Type II diabetes mellitus - poor control |
| C10F900 | Type 2 diabetes mellitus without complication |
| C10F911 | Type II diabetes mellitus without complication |
| C10FA00 | Type 2 diabetes mellitus with mononeuropathy |
| C10FA11 | Type II diabetes mellitus with mononeuropathy |
| C10FB00 | Type 2 diabetes mellitus with polyneuropathy |
| C10FB11 | Type II diabetes mellitus with polyneuropathy |
| C10FC00 | Type 2 diabetes mellitus with nephropathy |
| C10FC11 | Type II diabetes mellitus with nephropathy |
| C10FD00 | Type 2 diabetes mellitus with hypoglycaemic coma |
| C10FD11 | Type II diabetes mellitus with hypoglycaemic coma |
| C10FE00 | Type 2 diabetes mellitus with diabetic cataract |
| C10FE11 | Type II diabetes mellitus with diabetic cataract |
| C10FF00 | Type 2 diabetes mellitus with peripheral angiopathy |
| C10FF11 | Type II diabetes mellitus with peripheral angiopathy |
| C10FG00 | Type 2 diabetes mellitus with arthropathy |
| C10FG11 | Type II diabetes mellitus with arthropathy |
| C10FH00 | Type 2 diabetes mellitus with neuropathic arthropathy |
| C10FH11 | Type II diabetes mellitus with neuropathic arthropathy |
| C10FJ00 | Insulin treated Type 2 diabetes mellitus |
| C10FJ11 | Insulin treated Type II diabetes mellitus |
| C10FK00 | Hyperosmolar non-ketotic state in type 2 diabetes mellitus |
| C10FL00 | Type 2 diabetes mellitus with persistent proteinuria |
| C10FL11 | Type II diabetes mellitus with persistent proteinuria |
| C10FM00 | Type 2 diabetes mellitus with persistent microalbuminuria |
| C10FM11 | Type II diabetes mellitus with persistent microalbuminuria |
| C10FN00 | Type 2 diabetes mellitus with ketoacidosis |
| C10FN11 | Type II diabetes mellitus with ketoacidosis |
| C10FP00 | Type 2 diabetes mellitus with ketoacidotic coma |
| C10FP11 | Type II diabetes mellitus with ketoacidotic coma |
| C10FQ00 | Type 2 diabetes mellitus with exudative maculopathy |
| C10FQ11 | Type II diabetes mellitus with exudative maculopathy |
| C10FR00 | Type 2 diabetes mellitus with gastroparesis |
| C10G.00 | Secondary pancreatic diabetes mellitus |
| C10G000 | Secondary pancreatic diabetes mellitus without complication |
| C10M.00 | Lipoatrophic diabetes mellitus |
| C10M000 | Lipoatrophic diabetes mellitus without complication |
| C10N.00 | Secondary diabetes mellitus |
| C10N000 | Secondary diabetes mellitus without complication |
| C10y.00 | Diabetes mellitus with other specified manifestation |
| C10y000 | Diabetes mellitus, juvenile, + other specified manifestation |
| C10y100 | Diabetes mellitus, adult, + other specified manifestation |
| C10yy00 | Other specified diabetes mellitus with other spec comps |
| C10yz00 | Diabetes mellitus NOS with other specified manifestation |
| C10z.00 | Diabetes mellitus with unspecified complication |
| C10z000 | Diabetes mellitus, juvenile type, + unspecified complication |
| C10z100 | Diabetes mellitus, adult onset, + unspecified complication |
| C10zy00 | Other specified diabetes mellitus with unspecified comps |
| C10zz00 | Diabetes mellitus NOS with unspecified complication |
| C135.00 | Diabetes insipidus |
| C135.12 | Diabetes insipidus - pituitary |
| C314.11 | Renal diabetes |
| C350011 | Bronzed diabetes |
| Cyu2.00 | [X]Diabetes mellitus |
| Cyu2000 | [X]Other specified diabetes mellitus |
| Cyu2300 | [X]Unspecified diabetes mellitus with renal complications |
| F345000 | Diabetic mononeuritis multiplex |
| F35z000 | Diabetic mononeuritis NOS |
| F372.00 | Polyneuropathy in diabetes |
| F372.11 | Diabetic polyneuropathy |
| F372.12 | Diabetic neuropathy |
| F372000 | Acute painful diabetic neuropathy |
| F372100 | Chronic painful diabetic neuropathy |
| F372200 | Asymptomatic diabetic neuropathy |
| F381300 | Myasthenic syndrome due to diabetic amyotrophy |
| F381311 | Diabetic amyotrophy |
| F3y0.00 | Diabetic mononeuropathy |
| F420.00 | Diabetic retinopathy |
| F420000 | Background diabetic retinopathy |
| F420100 | Proliferative diabetic retinopathy |
| F420200 | Preproliferative diabetic retinopathy |
| F420300 | Advanced diabetic maculopathy |
| F420400 | Diabetic maculopathy |
| F420500 | Advanced diabetic retinal disease |
| F420600 | Non proliferative diabetic retinopathy |
| F420700 | High risk proliferative diabetic retinopathy |
| F420800 | High risk non proliferative diabetic retinopathy |
| F420z00 | Diabetic retinopathy NOS |
| F440700 | Diabetic iritis |
| F464000 | Diabetic cataract |
| G73y000 | Diabetic peripheral angiopathy |
| K01x100 | Nephrotic syndrome in diabetes mellitus |
| K081.00 | Nephrogenic diabetes insipidus |
| Kyu0300 | [X]Glomerular disorders in diabetes mellitus |
| L180500 | Pre-existing diabetes mellitus, insulin-dependent |
| L180600 | Pre-existing diabetes mellitus, non-insulin-dependent |
| L180X00 | Pre-existing diabetes mellitus, unspecified |
| Lyu2900 | [X]Pre-existing diabetes mellitus, unspecified |

## Gastrointestinal Disease

Gastrointestinal bleeding

| **Read Code** | **Description** |
| --- | --- |
| **Those we believe should be included** | |
| J68z.11 | GIB - Gastrointestinal bleeding |

## Renal Disease

Myoglobinuria

| **Read Code** | **Description** |
| --- | --- |
| **Those we believe should be included** | |
| R113.00 | [D]Myoglobinuria |

Nephrotic syndrome

| **Read Code** | **Description** |
| --- | --- |
| **Those we believe should be included** | |
| K0...00 | Nephritis, nephrosis and nephrotic syndrome |
| K01..00 | Nephrotic syndrome |
| K010.00 | Nephrotic syndrome with proliferative glomerulonephritis |
| K011.00 | Nephrotic syndrome with membranous glomerulonephritis |
| K012.00 | Nephrotic syndrome+membranoproliferative glomerulonephritis |
| K013.00 | Nephrotic syndrome with minimal change glomerulonephritis |
| K014.00 | Nephrotic syndrome, minor glomerular abnormality |
| K015.00 | Nephrotic syndrome, focal and segmental glomerular lesions |
| K016.00 | Nephrotic syndrome, diffuse membranous glomerulonephritis |
| K01A.00 | Nephrotic syndrome, dense deposit disease |
| K01B.00 | Nephrotic syndrome, diffuse crescentic glomerulonephritis |
| K01w.00 | Congenital nephrotic syndrome |
| K01w011 | Microcystic type congenital nephrotic syndrome |
| K01wz00 | Congenital nephrotic syndrome NOS |
| K01x.00 | Nephrotic syndrome in diseases EC |
| K01xz00 | Nephrotic syndrome in diseases EC NOS |
| K01y.00 | Nephrotic syndrome with other pathological kidney lesions |
| K01z.00 | Nephrotic syndrome NOS |
| K0y..00 | Other specified nephritis, nephrosis or nephrotic syndrome |
| K0z..00 | Nephritis, nephrosis and nephrotic syndrome NOS |
| K01x100 | Nephrotic syndrome in diabetes mellitus |

Renal disease

| **Read Code** | **Description** |
| --- | --- |
| **Those we believe should be included** | |
| G22..00 | Hypertensive renal disease |
| G220.00 | Malignant hypertensive renal disease |
| G221.00 | Benign hypertensive renal disease |
| G222.00 | Hypertensive renal disease with renal failure |
| G22z.00 | Hypertensive renal disease NOS |
| G23..00 | Hypertensive heart and renal disease |
| G230.00 | Malignant hypertensive heart and renal disease |
| G231.00 | Benign hypertensive heart and renal disease |
| G233.00 | Hypertensive heart and renal disease with renal failure |
| G23z.00 | Hypertensive heart and renal disease NOS |
| K0D..00 | End-stage renal disease |

## Respiratory Disease

Asthma

| **Read Code** | **Description** |
| --- | --- |
| **Those we believe should be included** | |
| 663d.00 | Emergency asthma admission since last appointment |
| 663m.00 | Asthma accident and emergency attendance since last visit |
| 8H2P.00 | Emergency admission, asthma |
| 9N1d.00 | Seen in asthma clinic |
| G581.11 | Asthma - cardiac |
| H312000 | Chronic asthmatic bronchitis |
| H33..00 | Asthma |
| H33..11 | Bronchial asthma |
| H333.00 | Acute exacerbation of asthma |
| H334.00 | Brittle asthma |
| H33z.00 | Asthma unspecified |
| H33z011 | Severe asthma attack |
| H33z100 | Asthma attack |
| H33z111 | Asthma attack NOS |
| 663V100 | Mild asthma |
| 663V200 | Moderate asthma |
| 663V300 | Severe asthma |
| 66YC.00 | Absent from work or school due to asthma |
| H33z000 | Status asthmaticus NOS |
| 1J70.00 | Suspected asthma |
| 1O2..00 | Asthma confirmed |
| 663j.00 | Asthma - currently active |
| 663N.00 | Asthma disturbing sleep |
| 8HTT.00 | Referral to asthma clinic |
| H33zz00 | Asthma NOS |

Bronchitis

| **Read Code** | **Description** |
| --- | --- |
| **Those we believe should be included** | |
| H06..00 | Acute bronchitis and bronchiolitis |
| H060.00 | Acute bronchitis |
| H060.11 | Acute wheezy bronchitis |
| H060000 | Acute fibrinous bronchitis |
| H060100 | Acute membranous bronchitis |
| H060200 | Acute pseudomembranous bronchitis |
| H060300 | Acute purulent bronchitis |
| H060400 | Acute croupous bronchitis |
| H060500 | Acute tracheobronchitis |
| H060A00 | Acute bronchitis due to mycoplasma pneumoniae |
| H060v00 | Subacute bronchitis unspecified |
| H060w00 | Acute viral bronchitis unspecified |
| H060x00 | Acute bacterial bronchitis unspecified |
| H060z00 | Acute bronchitis NOS |
| H06z.00 | Acute bronchitis or bronchiolitis NOS |
| H30..00 | Bronchitis unspecified |
| H30..11 | Chest infection - unspecified bronchitis |
| H30..12 | Recurrent wheezy bronchitis |
| H300.00 | Tracheobronchitis NOS |
| H301.00 | Laryngotracheobronchitis |
| H302.00 | Wheezy bronchitis |
| H30z.00 | Bronchitis NOS |
| Hyu1000 | [X]Acute bronchitis due to other specified organisms |
| H060600 | Acute pneumococcal bronchitis |
| H060700 | Acute streptococcal bronchitis |
| H060800 | Acute haemophilus influenzae bronchitis |
| H060900 | Acute neisseria catarrhalis bronchitis |
| H31..00 | Chronic bronchitis |
| H310.00 | Simple chronic bronchitis |
| H310000 | Chronic catarrhal bronchitis |
| H310z00 | Simple chronic bronchitis NOS |
| H311.00 | Mucopurulent chronic bronchitis |
| H311000 | Purulent chronic bronchitis |
| H311100 | Fetid chronic bronchitis |
| H311z00 | Mucopurulent chronic bronchitis NOS |
| H312.00 | Obstructive chronic bronchitis |
| H312000 | Chronic asthmatic bronchitis |
| H312011 | Chronic wheezy bronchitis |
| H312100 | Emphysematous bronchitis |
| H312z00 | Obstructive chronic bronchitis NOS |
| H313.00 | Mixed simple and mucopurulent chronic bronchitis |
| H31y.00 | Other chronic bronchitis |
| H31y100 | Chronic tracheobronchitis |
| H31yz00 | Other chronic bronchitis NOS |
| H31z.00 | Chronic bronchitis NOS |
| H060B00 | Acute bronchitis due to coxsackievirus |
| H060C00 | Acute bronchitis due to parainfluenza virus |
| H060D00 | Acute bronchitis due to respiratory syncytial virus |
| H060E00 | Acute bronchitis due to rhinovirus |
| H060F00 | Acute bronchitis due to echovirus |

Chronic obstructive pulmonary disease

| **Read Code** | **Description** |
| --- | --- |
| **Those we believe should be included** | |
| 66Yd.00 | COPD accident and emergency attendance since last visit |
| 66Ye.00 | Emergency COPD admission since last appointment |
| 8H2R.00 | Admit COPD emergency |
| H3...00 | Chronic obstructive pulmonary disease |
| H3...11 | Chronic obstructive airways disease |
| H312200 | Acute exacerbation of chronic obstructive airways disease |
| H36..00 | Mild chronic obstructive pulmonary disease |
| H37..00 | Moderate chronic obstructive pulmonary disease |
| H38..00 | Severe chronic obstructive pulmonary disease |
| H3y..00 | Other specified chronic obstructive airways disease |
| H3y..11 | Other specified chronic obstructive pulmonary disease |
| H3z..00 | Chronic obstructive airways disease NOS |
| H3z..11 | Chronic obstructive pulmonary disease NOS |
| Hyu3100 | [X]Other specified chronic obstructive pulmonary disease |
| 66Yi.00 | Multiple COPD emergency hospital admissions |
| 66Yg.00 | Chronic obstructive pulmonary disease disturbs sleep |

Chronic respiratory disease

| **Read Code** | **Description** |
| --- | --- |
| **Those we believe should be included** | |
| C370.00 | Cystic fibrosis |
| C370.11 | Fibrocystic disease |
| C370.12 | Mucoviscidosis |
| C370000 | Cystic fibrosis with no meconium ileus |
| C370100 | Cystic fibrosis with meconium ileus |
| C370111 | Meconium ileus in cystic fibrosis |
| C370200 | Cystic fibrosis with pulmonary manifestations |
| C370300 | Cystic fibrosis with intestinal manifestations |
| C370y00 | Cystic fibrosis with other manifestations |
| C370y11 | Cystic fibrosis with combined manifestations |
| C370z00 | Cystic fibrosis NOS |
| H541.00 | Pulmonary congestion |
| H541000 | Chronic pulmonary oedema |
| H541z00 | Pulmonary oedema NOS |
| H56..00 | Other alveolar and parietoalveolar disease |
| H560.00 | Pulmonary alveolar proteinosis |
| H561.00 | Idiopathic pulmonary haemosiderosis |
| H562.00 | Pulmonary alveolar microlithiasis |
| H563.00 | Idiopathic fibrosing alveolitis |
| H563.11 | Hamman - Rich syndrome |
| H563.12 | Cryptogenic fibrosing alveolitis |
| H563000 | Alveolar capillary block |
| H563100 | Diffuse pulmonary fibrosis |
| H563z00 | Idiopathic fibrosing alveolitis NOS |
| H564.00 | Bronchiolitis obliterans organising pneumonia |
| H56y.00 | Other alveolar and parietoalveolar disease |
| H56y000 | Endogenous lipoid pneumonia |
| H56y100 | Interstitial pneumonia |
| H56yz00 | Other alveolar and parietoalveolar disease NOS |
| H56z.00 | Alveolar and parietoalveolar disease NOS |
| H58..00 | Other diseases of lung |
| H580.00 | Pulmonary collapse with atelectasis |
| H580.11 | Atelectasis |
| H580.12 | Collapse of lung |
| H580000 | Post operative atelectasis |
| H581.00 | Interstitial emphysema |
| H581.11 | Pneumomediastinum |
| H582.00 | Compensatory emphysema |
| H583.00 | Pulmonary eosinophilia |
| H583000 | Loeffler's syndrome |
| H583100 | Tropical eosinophilia |
| H583z00 | Pulmonary eosinophilia NOS |
| H584.00 | Acute pulmonary oedema unspecified |
| H584.11 | Acute oedema of lung, unspecified |
| H584000 | Postoperative pulmonary oedema |
| H584z00 | Acute pulmonary oedema NOS |
| H585.11 | Adult respiratory distress syndrome |
| H585300 | Adult respiratory distress syndrome |
| H58y.00 | Other lung disease NEC |
| H58y000 | Broncholithiasis |
| H58y100 | Calcification of lung |
| H58y200 | Pulmolithiasis |
| H58y300 | Interstitial lung disease NEC |
| H58y400 | Squamous metaplasia of lung |
| H58yz00 | Other lung disease NEC NOS |
| H58z.00 | Lung disease NOS |
| Q314.00 | Primary atelectasis |
| Q317200 | Wilson-Mikity syndrome |

Emphysema

| **Read Code** | **Description** |
| --- | --- |
| **Those we believe should be included** | |
| H312100 | Emphysematous bronchitis |
| H32..00 | Emphysema |
| H320.00 | Chronic bullous emphysema |
| H320000 | Segmental bullous emphysema |
| H320100 | Zonal bullous emphysema |
| H320200 | Giant bullous emphysema |
| H320300 | Bullous emphysema with collapse |
| H320z00 | Chronic bullous emphysema NOS |
| H321.00 | Panlobular emphysema |
| H322.00 | Centrilobular emphysema |
| H32y.00 | Other emphysema |
| H32y000 | Acute vesicular emphysema |
| H32y100 | Atrophic (senile) emphysema |
| H32y111 | Acute interstitial emphysema |
| H32y200 | MacLeod's unilateral emphysema |
| H32yz00 | Other emphysema NOS |
| H32z.00 | Emphysema NOS |
| H581.00 | Interstitial emphysema |
| H582.00 | Compensatory emphysema |
| Hyu3000 | [X]Other emphysema |
| Q312.00 | Perinatal interstitial emphysema and related conditions |
| Q312111 | Perinatal mediastinal emphysema |
| Q312300 | Perinatal interstitial emphysema |
| Q312y00 | Perinatal interstitial emphysema or related condition OS |
| Q312z00 | Perinatal interstitial emphysema or related condition NOS |
| Qyu3400 | [X]Oth conds relat/interstial emphysema orig perinatl period |
| SK07.00 | Subcutaneous emphysema |
| J650200 | Acute emphysematous cholecystitis |

Influenza

| **Read Code** | **Description** |
| --- | --- |
| **Those we believe should be included** | |
| A08..11 | Gastric flu |
| F030800 | Encephalitis due to influenza-specific virus not identified |
| F030A00 | Encephalitis due to influenza-virus identified |
| H2...00 | Pneumonia and influenza |
| H27..00 | Influenza |
| H270.00 | Influenza with pneumonia |
| H270.11 | Chest infection - influenza with pneumonia |
| H270000 | Influenza with bronchopneumonia |
| H270100 | Influenza with pneumonia, influenza virus identified |
| H270z00 | Influenza with pneumonia NOS |
| H271.00 | Influenza with other respiratory manifestation |
| H271000 | Influenza with laryngitis |
| H271100 | Influenza with pharyngitis |
| H271z00 | Influenza with respiratory manifestations NOS |
| H27y.00 | Influenza with other manifestations |
| H27y000 | Influenza with encephalopathy |
| H27y100 | Influenza with gastrointestinal tract involvement |
| H27yz00 | Influenza with other manifestations NOS |
| H27z.00 | Influenza NOS |
| Hyu0400 | [X]Flu+oth respiratory manifestations,'flu virus identified |
| Hyu0500 | [X]Influenza+other manifestations,influenza virus identified |
| Hyu0600 | [X]Influenza+oth respiratory manifestatns,virus not identifd |
| Hyu0700 | [X]Influenza+other manifestations, virus not identified |
| 16L..00 | Influenza-like symptoms |
| H27z.11 | Flu like illness |
| H27z.12 | Influenza like illness |
| H2y..00 | Other specified pneumonia or influenza |
| H2z..00 | Pneumonia or influenza NOS |
| 65E..00 | Influenza vaccination |
| 68NE.00 | No consent - influenza imm. |
| 68NN.00 | Influenza imm.advised in surg. |
| 68NN.11 | Influenza immunization advised |
| 68NO.00 | Influenza imm.advised at home |
| 68NV.00 | Influenza vacc consent given |
| 8I2F.00 | Influenza vaccination contraindicated |
| 8I6D.00 | Influenza vaccination not indicated |
| 9k7..00 | Influenza immunisation - enhanced services administration |
| 9N4q.00 | Did not attend flu vaccination appointment |
| 9OX..00 | Influenza vacc. administration. |
| 9OX..11 | Flu vaccination administration |
| 9OX1.00 | Has 'flu vaccination at home |
| 9OX2.00 | Has'flu vaccination at surgery |
| 9OX3.00 | Has 'flu vaccination at hosp. |
| 9OX4.00 | Needs influenza immunisation |
| 9OX5.00 | Influenza vaccination declined |
| 9OX6.00 | Influenza vaccination invitation letter sent |
| 9OX7.00 | Influenza vaccination telephone invite |
| 9OX8.00 | Has influenza vaccination at work |
| 9OX9.00 | Influenza vaccination invitation first letter sent |
| 9OXA.00 | Influenza vaccination invitation second letter sent |
| 9OXB.00 | Influenza vaccination invitation third letter sent |
| 9OXZ.00 | Influenza vacc.administrat.NOS |
| ZV04800 | [V]Influenza vaccination |
| ZV04811 | [V]Flu - influenza vaccination |
| ZV14F00 | [V]Personal history of influenza vaccine allergy |

Laryngitis & Tracheitis

| **Read Code** | **Description** |
| --- | --- |
| **Those we believe should be included** | |
| H04..00 | Acute laryngitis and tracheitis |
| H040.00 | Acute laryngitis |
| H040w00 | Acute viral laryngitis unspecified |
| H040z00 | Acute laryngitis NOS |
| H16..00 | Chronic laryngitis and laryngotracheitis |
| H160.00 | Chronic laryngitis |
| H160000 | Chronic simple laryngitis |
| H160z00 | Chronic laryngitis NOS |
| H16z.00 | Chronic laryngitis NOS |
| H271000 | Influenza with laryngitis |
| H040000 | Acute oedematous laryngitis |
| H040100 | Acute ulcerative laryngitis |
| H040200 | Acute catarrhal laryngitis |
| H040300 | Acute phlegmonous laryngitis |
| H040400 | Acute haemophilus influenzae laryngitis |
| H040500 | Acute pneumococcal laryngitis |
| H040600 | Acute suppurative laryngitis |
| H043200 | Acute obstructive laryngitis |
| H04z.00 | Acute laryngitis and tracheitis NOS |
| H055.00 | Pharyngolaryngitis |
| H160100 | Chronic catarrhal laryngitis |
| H160200 | Chronic hypertrophic laryngitis |
| H160300 | Chronic atrophic laryngitis |
| H160400 | Laryngitis sicca |
| H040x00 | Acute bacterial laryngitis unspecified |

Laryngotracheobronchitis

| **Read Code** | **Description** |
| --- | --- |
| **Those we believe should be included** | |
| H301.00 | Laryngotracheobronchitis |
| H043211 | Croup |
| H044.00 | Croup |
| H060400 | Acute croupous bronchitis |

Lung abscess

| **Read Code** | **Description** |
| --- | --- |
| **Those we believe should be included** | |
| A054.00 | Amoebic lung abscess |
| H530000 | Single lung abscess |
| H530100 | Multiple lung abscess |

Nasopharyngitis

| **Read Code** | **Description** |
| --- | --- |
| **Those we believe should be included** | |
| H00..00 | Acute nasopharyngitis |
| H12..00 | Chronic pharyngitis and nasopharyngitis |
| H122.00 | Chronic nasopharyngitis |
| H12z.00 | Chronic pharyngitis and nasopharyngitis NOS |

Other respiratory codes

| **Read Code** | **Description** |
| --- | --- |
| **Those we believe should be included** | |
| H270.00 | Influenza with pneumonia |
| H270000 | Influenza with bronchopneumonia |
| H270100 | Influenza with pneumonia, influenza virus identified |
| H270z00 | Influenza with pneumonia NOS |
| H271000 | Influenza with laryngitis |
| H271100 | Influenza with pharyngitis |
| H271z00 | Influenza with respiratory manifestations NOS |
| H27y.00 | Influenza with other manifestations |
| Hyu0400 | [X]Flu+oth respiratory manifestations,'flu virus identified |
| Hyu0600 | [X]Influenza+oth respiratory manifestatns,virus not identifd |

Pharyngitis acute & chronic

| **Read Code** | **Description** |
| --- | --- |
| **Those we believe should be included** | |
| H00..00 | Acute nasopharyngitis |
| H02..00 | Acute pharyngitis |
| H02..13 | Throat infection - pharyngitis |
| H024.00 | Acute viral pharyngitis |
| H02z.00 | Acute pharyngitis NOS |
| H050.00 | Acute laryngopharyngitis |
| H053.00 | Tracheopharyngitis |
| H12..00 | Chronic pharyngitis and nasopharyngitis |
| H121.00 | Chronic pharyngitis |
| H121000 | Simple chronic pharyngitis |
| H121z00 | Chronic pharyngitis NOS |
| H122.00 | Chronic nasopharyngitis |
| H12z.00 | Chronic pharyngitis and nasopharyngitis NOS |
| H271100 | Influenza with pharyngitis |
| Hyu0100 | [X]Acute pharyngitis due to other specified organisms |
| 2DC2.00 | O/E - granular pharyngitis |
| A340200 | Streptococcal pharyngitis |
| AA12.00 | Vincent's pharyngitis |
| AA25.11 | Rhinopharyngitis mutilans |
| H020.00 | Acute gangrenous pharyngitis |
| H021.00 | Acute phlegmonous pharyngitis |
| H022.00 | Acute ulcerative pharyngitis |
| H023.00 | Acute bacterial pharyngitis |
| H023000 | Acute pneumococcal pharyngitis |
| H023100 | Acute staphylococcal pharyngitis |
| H023z00 | Acute bacterial pharyngitis NOS |
| H121100 | Atrophic pharyngitis |
| H121200 | Granular pharyngitis |
| H121300 | Hypertrophic pharyngitis |
| H121400 | Pharyngitis keratosa |
| H121500 | Pharyngitis sicca |
| H121600 | Chronic follicular pharyngitis |

Pneumonia and secondary bacterial pneumonia

| **Read Code** | **Description** |
| --- | --- |
| **Those we believe should be included** | |
| A116.00 | Tuberculous pneumonia |
| A380300 | Septicaemia due to streptococcus pneumoniae |
| A3BXA00 | Mycoplasma pneumoniae [PPLO] cause/dis classifd/oth chaptr |
| A3BXB00 | Klebsiella pneumoniae/cause/disease classifd/oth chapters |
| A3By400 | Pleuropneumonia-like organism (PPLO) infection |
| H060A00 | Acute bronchitis due to mycoplasma pneumoniae |
| H2...00 | Pneumonia and influenza |
| H20..00 | Viral pneumonia |
| H20..11 | Chest infection - viral pneumonia |
| H20y.00 | Viral pneumonia NEC |
| H20z.00 | Viral pneumonia NOS |
| H21..00 | Lobar (pneumococcal) pneumonia |
| H21..11 | Chest infection - pneumococcal pneumonia |
| H22..00 | Other bacterial pneumonia |
| H22..11 | Chest infection - other bacterial pneumonia |
| H220.00 | Pneumonia due to klebsiella pneumoniae |
| H221.00 | Pneumonia due to pseudomonas |
| H222.00 | Pneumonia due to haemophilus influenzae |
| H222.11 | Pneumonia due to haemophilus influenzae |
| H223.00 | Pneumonia due to streptococcus |
| H223000 | Pneumonia due to streptococcus, group B |
| H224.00 | Pneumonia due to staphylococcus |
| H22y.00 | Pneumonia due to other specified bacteria |
| H22y000 | Pneumonia due to escherichia coli |
| H22y011 | E.coli pneumonia |
| H22y100 | Pneumonia due to proteus |
| H22y200 | Pneumonia - Legionella |
| H22yX00 | Pneumonia due to other aerobic gram-negative bacteria |
| H22yz00 | Pneumonia due to bacteria NOS |
| H22z.00 | Bacterial pneumonia NOS |
| H23..00 | Pneumonia due to other specified organisms |
| H23..11 | Chest infection - pneumonia organism OS |
| H230.00 | Pneumonia due to Eaton's agent |
| H231.00 | Pneumonia due to mycoplasma pneumoniae |
| H232.00 | Pneumonia due to pleuropneumonia like organisms |
| H23z.00 | Pneumonia due to specified organism NOS |
| H24..00 | Pneumonia with infectious diseases EC |
| H246.00 | Pneumonia with aspergillosis |
| H24y.00 | Pneumonia with other infectious diseases EC |
| H24yz00 | Pneumonia with other infectious diseases EC NOS |
| H24z.00 | Pneumonia with infectious diseases EC NOS |
| H25..00 | Bronchopneumonia due to unspecified organism |
| H25..11 | Chest infection - unspecified bronchopneumonia |
| H26..00 | Pneumonia due to unspecified organism |
| H260.00 | Lobar pneumonia due to unspecified organism |
| H261.00 | Basal pneumonia due to unspecified organism |
| H270.00 | Influenza with pneumonia |
| H270.11 | Chest infection - influenza with pneumonia |
| H270000 | Influenza with bronchopneumonia |
| H270100 | Influenza with pneumonia, influenza virus identified |
| H270z00 | Influenza with pneumonia NOS |
| H28..00 | Atypical pneumonia |
| H2y..00 | Other specified pneumonia or influenza |
| H2z..00 | Pneumonia or influenza NOS |
| H530300 | Abscess of lung with pneumonia |
| H56y100 | Interstitial pneumonia |
| H571.00 | Rheumatic pneumonia |
| Hyu0800 | [X]Other viral pneumonia |
| Hyu0900 | [X]Pneumonia due to other aerobic gram-negative bacteria |
| Hyu0A00 | [X]Other bacterial pneumonia |
| Hyu0B00 | [X]Pneumonia due to other specified infectious organisms |
| Hyu0C00 | [X]Pneumonia in bacterial diseases classified elsewhere |
| Hyu0D00 | [X]Pneumonia in viral diseases classified elsewhere |
| Hyu0E00 | [X]Pneumonia in mycoses classified elsewhere |
| Hyu0G00 | [X]Pneumonia in other diseases classified elsewhere |
| Hyu0H00 | [X]Other pneumonia, organism unspecified |
| H200.00 | Pneumonia due to adenovirus |
| H201.00 | Pneumonia due to respiratory syncytial virus |
| H202.00 | Pneumonia due to parainfluenza virus |
| H243.00 | Pneumonia with whooping cough |
| H243.11 | Pneumonia with pertussis |
| H24y700 | Pneumonia with varicella |
| H540000 | Hypostatic pneumonia |
| H540100 | Hypostatic bronchopneumonia |
| H564.00 | Bronchiolitis obliterans organising pneumonia |
| H56y000 | Endogenous lipoid pneumonia |
| Hyu0F00 | [X]Pneumonia in parasitic diseases classified elsewhere |
| Q310.00 | Congenital pneumonia |
| Q310000 | Congenital pneumonia due to staphylococcus |
| Q310100 | Congenital pneumonia due to group A haemolytic streptococcus |
| Q310200 | Congenital pneumonia due to group B haemolytic streptococcus |
| Q310300 | Congenital pneumonia due to Escherichia coli |
| Q310400 | Congenital pneumonia due to pseudomonas |
| Q310500 | Congenital pneumonia due to viral agent |
| Q310600 | Congenital pneumonia due to Chlamydia |
| Q310y00 | Other specified congenital pneumonia |
| Q310z00 | Congenital pneumonia NOS |
| Qyu3100 | [X]Congenital pneumonia due to other bacterial agents |
| Qyu3200 | [X]Congenital pneumonia due to other organisms |
| SP13100 | Other aspiration pneumonia as a complication of care |

Pneumothorax

| **Read Code** | **Description** |
| --- | --- |
| **Those we believe should be included** | |
| H501500 | Pyopneumothorax |
| H51y100 | Haemopneumothorax |
| H51y300 | Hydropneumothorax |
| H52..00 | Pneumothorax |
| H520.00 | Spontaneous tension pneumothorax |
| H52y.00 | Other spontaneous pneumothorax |
| H52y000 | Acute pneumothorax NOS |
| H52y100 | Chronic pneumothorax |
| H52yz00 | Other spontaneous pneumothorax NOS |
| H52yz11 | Spontaneous pneumothorax NOS |
| H52z.00 | Pneumothorax NOS |
| Hyu7100 | [X]Other spontaneous pneumothorax |
| Hyu7200 | [X]Other pneumothorax |
| Q312000 | Perinatal pneumothorax |
| S70..00 | Traumatic pneumothorax and haemothorax |
| S700.00 | Closed traumatic pneumothorax |
| S701.00 | Open traumatic pneumothorax |
| S704.00 | Closed traumatic haemopneumothorax |
| S705.00 | Open traumatic haemopneumothorax |
| S706.00 | Traumatic pneumothorax |
| S708.00 | Traumatic haemopneumothorax |
| S70z.00 | Traumatic pneumothorax and haemothorax NOS |

Pulmonary aspergillosis

| **Read Code** | **Description** |
| --- | --- |
| **Those we believe should be included** | |
| AB63.00 | Aspergillosis |
| AB63000 | Invasive pulmonary aspergillosis |
| AB63X00 | Aspergillosis, unspecified |
| AyuEK00 | [X]Other forms of aspergillosis |
| AyuEL00 | [X]Aspergillosis, unspecified |
| AyuEU00 | [X]Other pulmonary aspergillosis |
| H246.00 | Pneumonia with aspergillosis |
| **Those we believe may be needed** | |
| AB63200 | Disseminated aspergillosis |
| **Those that could be considered relevant that we don’t believe should be used** | |
| AB63100 | Tonsillar aspergillosis |
| F501B00 | Chronic otitis externa due to aspergillosis |

Sinusitis

| **Read Code** | **Description** |
| --- | --- |
| **Those we believe should be included** | |
| H01..00 | Acute sinusitis |
| H01..11 | Sinusitis |
| H010.00 | Acute maxillary sinusitis |
| H011.00 | Acute frontal sinusitis |
| H012.00 | Acute ethmoidal sinusitis |
| H013.00 | Acute sphenoidal sinusitis |
| H01y.00 | Other acute sinusitis |
| H01y000 | Acute pansinusitis |
| H01yz00 | Other acute sinusitis NOS |
| H01z.00 | Acute sinusitis NOS |
| H13..00 | Chronic sinusitis |
| H13..11 | Chronic rhinosinusitis |
| H130.00 | Chronic maxillary sinusitis |
| H130.12 | Maxillary sinusitis |
| H131.00 | Chronic frontal sinusitis |
| H131.11 | Frontal sinusitis |
| H132.00 | Chronic ethmoidal sinusitis |
| H133.00 | Chronic sphenoidal sinusitis |
| H135.00 | Recurrent sinusitis |
| H13y.00 | Other chronic sinusitis |
| H13y000 | Chronic pansinusitis |
| H13y100 | Pansinusitis |
| H13yz00 | Other chronic sinusitis NOS |
| H13z.00 | Chronic sinusitis NOS |
| Hyu0000 | [X]Other acute sinusitis |
| Hyu2200 | [X]Other chronic sinusitis |
| SN31.11 | Aerosinusitis |

Tonsillitis

| **Read Code** | **Description** |
| --- | --- |
| **Those we believe should be included** | |
| H03..00 | Acute tonsillitis |
| H03..11 | Throat infection - tonsillitis |
| H03..12 | Tonsillitis |
| H036.00 | Acute viral tonsillitis |
| H037.00 | Recurrent acute tonsillitis |
| H03z.00 | Acute tonsillitis NOS |
| H140.00 | Chronic tonsillitis |
| Hyu0200 | [X]Acute tonsillitis due to other specified organisms |
| AA1z.12 | Vincent's tonsillitis |
| H030.00 | Acute erythematous tonsillitis |
| H031.00 | Acute follicular tonsillitis |
| H032.00 | Acute ulcerative tonsillitis |
| H033.00 | Acute catarrhal tonsillitis |
| H034.00 | Acute gangrenous tonsillitis |
| H14y500 | Caseous tonsillitis |
| H14y600 | Lingular tonsillitis |
| A340300 | Streptococcal tonsillitis |
| A383000 | Fusobacterial necrotising tonsillitis |
| H035.00 | Acute bacterial tonsillitis |
| H035000 | Acute pneumococcal tonsillitis |
| H035100 | Acute staphylococcal tonsillitis |
| H035z00 | Acute bacterial tonsillitis NOS |
| H143.00 | Chronic adenotonsillitis |

##

## Other Disease

Encephalitis

| **Read Code** | **Description** |
| --- | --- |
| **Those we believe should be included** | |
| A4zz.11 | Viral encephalitis NOS |
| Ayu8A00 | [X]Other specified viral encephalitis |
| Ayu8B00 | [X]Unspecified viral encephalitis |
| AyuJ800 | [X]Sequelae of viral encephalitis |
| F03..00 | Encephalitis, myelitis and encephalomyelitis |
| F030.00 | Encephalitis in viral disease EC |
| F030800 | Encephalitis due to influenza-specific virus not identified |
| F030A00 | Encephalitis due to influenza-virus identified |
| F030z00 | Encephalitis in viral disease NOS |
| F033.00 | Encephalitis due to other infection EC |
| F033z00 | Unspecified encephalitis due to other infection EC |
| F034G00 | Post influenza vaccination encephalitis |
| F035.00 | Postinfectious encephalitis |
| F03y.00 | Other causes of encephalitis |
| F03z.00 | Encephalitis NOS |
| Fyu0600 | [X]Other encephalitis, myelitis and encephalomyelitis |
| Fyu0700 | [X]Encephalitis,myelitis+encephalomyelitis/bactrl disease CE |
| Fyu0800 | [X]Encephalitis,myelitis+encephalomyelitis/viral disease CE |
| Fyu0A00 | [X]Encephalitis,myelitis+encephalomyelitis/other diseases CE |
| ZV05000 | [V]Arthropod-borne viral encephalitis vaccination |
| ZV73512 | [V]Screening for mosquito viral encephalitis |

Encephalopathy

| **Read Code** | **Description** |
| --- | --- |
| H27y000 | Influenza with encephalopathy |

Febrile convulsions

| **Read Code** | **Description** |
| --- | --- |
| **Those we believe should be included** | |
| 1B6B.00 | Febrile convulsion |

Guillain-Barre syndrome

| **Read Code** | **Description** |
| --- | --- |
| **Those we believe should be included** | |
| F370000 | Guillain-Barre syndrome |

Meningitis

| **Read Code** | **Description** |
| --- | --- |
| **Those we believe should be included** | |
| A42y.00 | Other specified viral meningitis |
| A42z.00 | Viral meningitis NOS |
| Ayu8C00 | [X]Other viral meningitis |
| Ayu8D00 | [X]Viral meningitis, unspecified |
| F011.00 | Meningitis due to viral organisms EC |
| F011y00 | Other viral meningitis |
| F011z00 | Meningitis - viral NOS |
| F01y.00 | Other non-bacterial meningitis |
| F01yz00 | Other non-bacterial meningitis NOS |
| F01z.00 | Meningitis due to organism NOS |
| F02..00 | Meningitis of unspecified cause |
| F022.00 | Chronic meningitis |
| F024.00 | Benign recurrent meningitis |
| F02z.00 | Unspecified meningitis |
| Fyu0100 | [X]Meningitis in viral diseases classified elsewhere |
| Fyu0400 | [X]Meningitis due to other specified causes |

Myositis

| **Read Code** | **Description** |
| --- | --- |
| **Those we believe should be included** | |
| F4G1200 | Orbital myositis |
| N003.00 | Dermatomyositis |
| N003000 | Juvenile dermatomyositis |
| N003X00 | Dermatopolymyositis, unspecified |
| N004.00 | Polymyositis |
| N230.00 | Infective myositis |
| N230.11 | Purulent myositis |
| N230.12 | Suppurative myositis |
| N230000 | Infective myositis-neck |
| N230100 | Infective myositis-back |
| N230200 | Infective myositis-shoulder |
| N230300 | Infective myositis-arm |
| N230400 | Infective myositis-forearm |
| N230500 | Infective myositis-hand |
| N230600 | Infective myositis-pelvis |
| N230700 | Infective myositis-thigh |
| N230800 | Infective myositis-leg |
| N230900 | Infective myositis-foot |
| N233200 | Myositis in sarcoidosis |
| N23y000 | Interstitial myositis |
| N241.00 | Myalgia and myositis unspecified |
| N241100 | Myositis unspecified |
| N241200 | Fibromyositis NOS |
| N241z00 | Myalgia or myositis NOS |
| Nyu4400 | [X]Other dermatomyositis |
| Nyu4E00 | [X]Dermatopolymyositis, unspecified |
| Nyu8000 | [X]Other myositis |
| Nyu8800 | [X]Myositis in other infectious diseases CE |
| Nyu8900 | [X]Myositis in sarcoidosis classified elsewhere |

Reye’s syndrome

| **Read Code** | **Description** |
| --- | --- |
| **Those we believe should be included** | |
| F11y000 | Reye's syndrome |

Sepsis

| **Read Code** | **Description** |
| --- | --- |
| **Those we believe should be included** | |
| A38z.11 | Sepsis |

Septicaemia

| **Read Code** | **Description** |
| --- | --- |
| **Those we believe should be included** | |
| A38..00 | Septicaemia |
| A021.00 | Salmonella septicaemia |
| A223.00 | Anthrax septicaemia |
| A270100 | Listeria septicaemia |
| A271100 | Erysipelothrix septicaemia |
| A362.00 | Meningococcal septicaemia |
| A366.00 | Meningococcal meningitis with meningococcal septicaemia |
| A380.00 | Streptococcal septicaemia |
| A380000 | Septicaemia due to streptococcus, group A |
| A380100 | Septicaemia due to streptococcus, group B |
| A380200 | Septicaemia due to streptococcus, group D |
| A380300 | Septicaemia due to streptococcus pneumoniae |
| A380400 | Septicaemia due to enterococcus |
| A380500 | Vancomycin resistant enterococcal septicaemia |
| A381.00 | Staphylococcal septicaemia |
| A381000 | Septicaemia due to Staphylococcus aureus |
| A381100 | Septicaemia due to coagulase-negative staphylococcus |
| A382.00 | Pneumococcal septicaemia |
| A383.00 | Septicaemia due to anaerobes |
| A384.00 | Septicaemia due to other gram negative organisms |
| A384000 | Gram negative septicaemia NOS |
| A384100 | Haemophilus influenzae septicaemia |
| A384200 | Escherichia coli septicaemia |
| A384211 | E.coli septicaemia |
| A384300 | Pseudomonas septicaemia |
| A384400 | Serratia septicaemia |
| A384z00 | Other gram negative septicaemia NOS |
| A38y.00 | Other specified septicaemias |
| A38z.00 | Septicaemia NOS |
| Ayu3J00 | [X]Septicaemia, unspecified |
| L403.00 | Puerperal septicaemia |
| L403000 | Puerperal septicaemia unspecified |
| L403z00 | Puerperal septicaemia NOS |
| Q407500 | Neonatal candida septicaemia |
| Q407511 | Neonatal monilial septicaemia |
| Q40y012 | Congenital septicaemia |
| Q40y200 | Septicaemia of newborn |

**Appendix IV**

# READ Codes for High Risk Groups

## Cardiovascular Disease

CVS (Vaccine High Risk)

| **Read Code** | **Description** |
| --- | --- |
| **Those we believe should be included** | |
| G1...00 | Chronic rheumatic heart disease |
| G10..00 | Chronic rheumatic pericarditis |
| G100.00 | Adherent rheumatic pericardium |
| G101.00 | Chronic rheumatic mediastinopericarditis |
| G102.00 | Chronic rheumatic myopericarditis |
| G10z.00 | Chronic rheumatic pericarditis NOS |
| G11..00 | Mitral valve diseases |
| G11..11 | Rheumatic mitral valve disease |
| G110.00 | Mitral stenosis |
| G110.11 | Rheumatic mitral stenosis |
| G111.00 | Rheumatic mitral insufficiency |
| G111.11 | Mitral incompetence - rheumatic |
| G111.12 | Mitral regurgitation - rheumatic |
| G112.00 | Mitral stenosis with insufficiency |
| G112.12 | Mitral stenosis with incompetence |
| G112.13 | Mitral stenosis with regurgitation |
| G113.00 | Nonrheumatic mitral valve stenosis |
| G114.00 | Ruptured mitral valve cusp |
| G11z.00 | Mitral valve disease NOS |
| G12..00 | Rheumatic aortic valve disease |
| G120.00 | Rheumatic aortic stenosis |
| G121.00 | Rheumatic aortic insufficiency |
| G121.11 | Aortic incompetence - rheumatic |
| G121.12 | Aortic regurgitation - rheumatic |
| G122.00 | Rheumatic aortic stenosis with insufficiency |
| G12z.00 | Rheumatic aortic valve disease NOS |
| G13..00 | Diseases of mitral and aortic valves |
| G130.00 | Mitral and aortic stenosis |
| G131.00 | Mitral stenosis and aortic insufficiency |
| G131.13 | Mitral stenosis and aortic incompetence |
| G131.14 | Mitral stenosis and aortic regurgitation |
| G132.00 | Mitral insufficiency and aortic stenosis |
| G132.12 | Mitral incompetence and aortic stenosis |
| G132.13 | Mitral regurgitation and aortic stenosis |
| G133.00 | Mitral and aortic incompetence |
| G133.11 | Mitral and aortic insufficiency |
| G133.12 | Mitral and aortic regurgitation |
| G13y.00 | Multiple mitral and aortic valve involvement |
| G13z.00 | Mitral and aortic valve disease NOS |
| G14..00 | Other chronic rheumatic endocardial disease |
| G140.00 | Tricuspid valve disease NEC |
| G140000 | Rheumatic tricuspid stenosis |
| G140100 | Rheumatic tricuspid insufficiency |
| G140111 | Tricuspid regurgitation - rheumatic |
| G140112 | Tricuspid incompetence - rheumatic |
| G140200 | Rheumatic tricuspid stenosis and insufficiency |
| G14021X | Rheumatic tricuspid stenosis and regurgitation |
| G14021Y | Rheumatic tricuspid stenosis and incompetence |
| G140300 | Tricuspid stenosis, cause unspecified |
| G140400 | Tricuspid insufficiency, cause unspecified |
| G140412 | Tricuspid incompetence, cause unspecified |
| G140413 | Tricuspid regurgitation, cause unspecified |
| G140500 | Tricuspid stenosis and insufficiency, cause unspecified |
| G140511 | Tricuspid stenosis and incompetence, cause unspecified |
| G140514 | Tricuspid stenosis and regurgitation, cause unspecified |
| G140z00 | Rheumatic tricuspid valve disease NOS |
| G141.00 | Rheumatic pulmonary valve disease |
| G141000 | Rheumatic pulmonary stenosis |
| G141100 | Rheumatic pulmonary insufficiency |
| G141200 | Rheumatic pulmonary stenosis and insufficiency |
| G141z00 | Rheumatic pulmonary valve disease NOS |
| G14z.00 | Rheumatic endocarditis NOS |
| G14z.11 | Rheumatic valvulitis, chronic NOS |
| G1y..00 | Other specified chronic rheumatic heart disease |
| G1y0.00 | Rheumatic myocarditis |
| G1yz.00 | Other and unspecified rheumatic heart disease |
| G1yz000 | Rheumatic heart disease unspecified |
| G1yz100 | Rheumatic left ventricular failure |
| G1yzz00 | Other rheumatic heart disease NOS |
| G1z..00 | Chronic rheumatic heart disease NOS |
| G2...00 | Hypertensive disease |
| G2...11 | BP - hypertensive disease |
| G21..00 | Hypertensive heart disease |
| G210.00 | Malignant hypertensive heart disease |
| G210000 | Malignant hypertensive heart disease without CCF |
| G210100 | Malignant hypertensive heart disease with CCF |
| G210z00 | Malignant hypertensive heart disease NOS |
| G211.00 | Benign hypertensive heart disease |
| G211000 | Benign hypertensive heart disease without CCF |
| G211100 | Benign hypertensive heart disease with CCF |
| G211z00 | Benign hypertensive heart disease NOS |
| G21z.00 | Hypertensive heart disease NOS |
| G21z000 | Hypertensive heart disease NOS without CCF |
| G21z011 | Cardiomegaly - hypertensive |
| G21z100 | Hypertensive heart disease NOS with CCF |
| G21zz00 | Hypertensive heart disease NOS |
| G22..00 | Hypertensive renal disease |
| G22..11 | Nephrosclerosis |
| G220.00 | Malignant hypertensive renal disease |
| G221.00 | Benign hypertensive renal disease |
| G222.00 | Hypertensive renal disease with renal failure |
| G22z.00 | Hypertensive renal disease NOS |
| G22z.11 | Renal hypertension |
| G23..00 | Hypertensive heart and renal disease |
| G230.00 | Malignant hypertensive heart and renal disease |
| G231.00 | Benign hypertensive heart and renal disease |
| G232.00 | Hypertensive heart&renal dis wth (congestive) heart failure |
| G233.00 | Hypertensive heart and renal disease with renal failure |
| G234.00 | Hyperten heart&renal dis+both(congestv)heart and renal fail |
| G23z.00 | Hypertensive heart and renal disease NOS |
| G24..00 | Secondary hypertension |
| G240.00 | Secondary malignant hypertension |
| G240000 | Secondary malignant renovascular hypertension |
| G240z00 | Secondary malignant hypertension NOS |
| G241.00 | Secondary benign hypertension |
| G241000 | Secondary benign renovascular hypertension |
| G241z00 | Secondary benign hypertension NOS |
| G244.00 | Hypertension secondary to endocrine disorders |
| G24z.00 | Secondary hypertension NOS |
| G24z000 | Secondary renovascular hypertension NOS |
| G24z100 | Hypertension secondary to drug |
| G24zz00 | Secondary hypertension NOS |
| G2y..00 | Other specified hypertensive disease |
| G3...00 | Ischaemic heart disease |
| G3...11 | Arteriosclerotic heart disease |
| G3...12 | Atherosclerotic heart disease |
| G3...13 | IHD - Ischaemic heart disease |
| G30..00 | Acute myocardial infarction |
| G30..11 | Attack - heart |
| G30..12 | Coronary thrombosis |
| G30..13 | Cardiac rupture following myocardial infarction (MI) |
| G30..14 | Heart attack |
| G30..15 | MI - acute myocardial infarction |
| G30..16 | Thrombosis - coronary |
| G30..17 | Silent myocardial infarction |
| G300.00 | Acute anterolateral infarction |
| G301.00 | Other specified anterior myocardial infarction |
| G301000 | Acute anteroapical infarction |
| G301100 | Acute anteroseptal infarction |
| G301z00 | Anterior myocardial infarction NOS |
| G302.00 | Acute inferolateral infarction |
| G303.00 | Acute inferoposterior infarction |
| G304.00 | Posterior myocardial infarction NOS |
| G305.00 | Lateral myocardial infarction NOS |
| G306.00 | True posterior myocardial infarction |
| G307.00 | Acute subendocardial infarction |
| G307000 | Acute non-Q wave infarction |
| G307100 | Acute non-ST segment elevation myocardial infarction |
| G308.00 | Inferior myocardial infarction NOS |
| G309.00 | Acute Q-wave infarct |
| G30A.00 | Mural thrombosis |
| G30B.00 | Acute posterolateral myocardial infarction |
| G30X.00 | Acute transmural myocardial infarction of unspecif site |
| G30X000 | Acute ST segment elevation myocardial infarction |
| G30y.00 | Other acute myocardial infarction |
| G30y000 | Acute atrial infarction |
| G30y100 | Acute papillary muscle infarction |
| G30y200 | Acute septal infarction |
| G30yz00 | Other acute myocardial infarction NOS |
| G30z.00 | Acute myocardial infarction NOS |
| G31..00 | Other acute and subacute ischaemic heart disease |
| G310.00 | Postmyocardial infarction syndrome |
| G310.11 | Dressler's syndrome |
| G311.00 | Preinfarction syndrome |
| G311.11 | Crescendo angina |
| G311.12 | Impending infarction |
| G311.13 | Unstable angina |
| G311.14 | Angina at rest |
| G311000 | Myocardial infarction aborted |
| G311011 | MI - myocardial infarction aborted |
| G311100 | Unstable angina |
| G311200 | Angina at rest |
| G311300 | Refractory angina |
| G311400 | Worsening angina |
| G311500 | Acute coronary syndrome |
| G311z00 | Preinfarction syndrome NOS |
| G312.00 | Coronary thrombosis not resulting in myocardial infarction |
| G31y.00 | Other acute and subacute ischaemic heart disease |
| G31y000 | Acute coronary insufficiency |
| G31y100 | Microinfarction of heart |
| G31y200 | Subendocardial ischaemia |
| G31y300 | Transient myocardial ischaemia |
| G31yz00 | Other acute and subacute ischaemic heart disease NOS |
| G32..00 | Old myocardial infarction |
| G32..11 | Healed myocardial infarction |
| G32..12 | Personal history of myocardial infarction |
| G33..00 | Angina pectoris |
| G330.00 | Angina decubitus |
| G330000 | Nocturnal angina |
| G330z00 | Angina decubitus NOS |
| G331.00 | Prinzmetal's angina |
| G331.11 | Variant angina pectoris |
| G332.00 | Coronary artery spasm |
| G33z.00 | Angina pectoris NOS |
| G33z000 | Status anginosus |
| G33z100 | Stenocardia |
| G33z200 | Syncope anginosa |
| G33z300 | Angina on effort |
| G33z400 | Ischaemic chest pain |
| G33z500 | Post infarct angina |
| G33z600 | New onset angina |
| G33z700 | Stable angina |
| G33zz00 | Angina pectoris NOS |
| G34..00 | Other chronic ischaemic heart disease |
| G340.00 | Coronary atherosclerosis |
| G340.11 | Triple vessel disease of the heart |
| G340.12 | Coronary artery disease |
| G340000 | Single coronary vessel disease |
| G340100 | Double coronary vessel disease |
| G341.00 | Aneurysm of heart |
| G341.11 | Cardiac aneurysm |
| G341000 | Ventricular cardiac aneurysm |
| G341100 | Other cardiac wall aneurysm |
| G341111 | Mural cardiac aneurysm |
| G341200 | Aneurysm of coronary vessels |
| G341300 | Acquired atrioventricular fistula of heart |
| G341z00 | Aneurysm of heart NOS |
| G342.00 | Atherosclerotic cardiovascular disease |
| G343.00 | Ischaemic cardiomyopathy |
| G344.00 | Silent myocardial ischaemia |
| G34y.00 | Other specified chronic ischaemic heart disease |
| G34y000 | Chronic coronary insufficiency |
| G34y100 | Chronic myocardial ischaemia |
| G34yz00 | Other specified chronic ischaemic heart disease NOS |
| G34z.00 | Other chronic ischaemic heart disease NOS |
| G34z000 | Asymptomatic coronary heart disease |
| G35..00 | Subsequent myocardial infarction |
| G350.00 | Subsequent myocardial infarction of anterior wall |
| G351.00 | Subsequent myocardial infarction of inferior wall |
| G353.00 | Subsequent myocardial infarction of other sites |
| G35X.00 | Subsequent myocardial infarction of unspecified site |
| G36..00 | Certain current complication follow acute myocardial infarct |
| G360.00 | Haemopericardium/current comp folow acut myocard infarct |
| G361.00 | Atrial septal defect/curr comp folow acut myocardal infarct |
| G362.00 | Ventric septal defect/curr comp fol acut myocardal infarctn |
| G363.00 | Ruptur cardiac wall w'out haemopericard/cur comp fol ac MI |
| G364.00 | Ruptur chordae tendinae/curr comp fol acute myocard infarct |
| G365.00 | Rupture papillary muscle/curr comp fol acute myocard infarct |
| G366.00 | Thrombosis atrium,auric append&vent/curr comp foll acute MI |
| G37..00 | Cardiac syndrome X |
| G38..00 | Postoperative myocardial infarction |
| G380.00 | Postoperative transmural myocardial infarction anterior wall |
| G381.00 | Postoperative transmural myocardial infarction inferior wall |
| G382.00 | Postoperative transmural myocardial infarction other sites |
| G383.00 | Postoperative transmural myocardial infarction unspec site |
| G384.00 | Postoperative subendocardial myocardial infarction |
| G38z.00 | Postoperative myocardial infarction, unspecified |
| G3y..00 | Other specified ischaemic heart disease |
| G3z..00 | Ischaemic heart disease NOS |
| G4...00 | Pulmonary circulation diseases |
| G4...11 | Heart disease - pulmonary |
| G40..00 | Acute pulmonary heart disease |
| G400.00 | Acute cor pulmonale |
| G401.00 | Pulmonary embolism |
| G401.11 | Infarction - pulmonary |
| G401.12 | Pulmonary embolus |
| G401000 | Post operative pulmonary embolus |
| G402.00 | Pulmonary infarct |
| G40z.00 | Acute pulmonary heart disease NOS |
| G41..00 | Chronic pulmonary heart disease |
| G410.00 | Primary pulmonary hypertension |
| G411.00 | Kyphoscoliotic heart disease |
| G41y.00 | Other chronic pulmonary heart disease |
| G41y000 | Secondary pulmonary hypertension |
| G41yz00 | Other chronic pulmonary heart disease NOS |
| G41z.00 | Chronic pulmonary heart disease NOS |
| G41z.11 | Chronic cor pulmonale |
| G42..00 | Other pulmonary circulation disease |
| G42..11 | Pulmonary vessel disease |
| G420.00 | Arteriovenous fistula of pulmonary vessels |
| G421.00 | Aneurysm of pulmonary artery |
| G42y.00 | Other specified pulmonary circulation disease |
| G42y000 | Pulmonary arteritis |
| G42y100 | Pulmonary vessel rupture |
| G42y200 | Pulmonary vessel stricture |
| G42yz00 | Other specified pulmonary circulation disease NOS |
| G42z.00 | Other pulmonary circulation disease NOS |
| G4y..00 | Other specified pulmonary circulation disease |
| G4z..00 | Pulmonary circulation disease NOS |
| G5...00 | Other forms of heart disease |
| G50..00 | Acute pericarditis |
| G50..11 | Pericardial effusion - acute |
| G500.00 | Acute pericarditis in diseases EC |
| G500000 | Acute pericarditis - coxsackie |
| G500100 | Acute pericarditis - meningococcal |
| G500200 | Acute pericarditis - syphilitic |
| G500300 | Acute pericarditis - tuberculous |
| G500311 | TB - acute pericarditis |
| G500400 | Acute pericarditis - uraemic |
| G500500 | Acute pericarditis - gonococcal |
| G500z00 | Acute pericarditis in diseases EC NOS |
| G501.00 | Post infarction pericarditis |
| G50z.00 | Other and unspecified acute pericarditis |
| G50z000 | Acute pericarditis - unspecified |
| G50z100 | Acute idiopathic pericarditis |
| G50z111 | Viral pericarditis NOS |
| G50z200 | Acute pericarditis - pneumococcal |
| G50z300 | Acute pericarditis - staphylococcal |
| G50z400 | Acute pericarditis - streptococcal |
| G50z500 | Acute purulent pericarditis unspecified |
| G50z511 | Pyopericardium |
| G50zz00 | Acute pericarditis NOS |
| G51..00 | Acute and subacute endocarditis |
| G510.00 | Acute and subacute bacterial endocarditis |
| G510.11 | Bacterial endocarditis |
| G510000 | Acute bacterial endocarditis |
| G510100 | Subacute bacterial endocarditis - SBE |
| G510200 | Chronic bacterial endocarditis |
| G510z00 | Acute and subacute bacterial endocarditis NOS |
| G511.00 | Acute and subacute infective endocarditis in diseases EC |
| G511000 | Endocarditis - monilial |
| G511100 | Endocarditis - coxsackie |
| G511200 | Endocarditis - gonococcal |
| G511300 | Endocarditis - meningococcal |
| G511400 | Endocarditis - typhoid |
| G511500 | Endocarditis - blastomycosis |
| G511600 | Endocarditis - Q fever |
| G511z00 | Infective endocarditis in diseases EC, NOS |
| G51z.00 | Acute and subacute endocarditis unspecified |
| G51z000 | Acute endocarditis NOS |
| G51z100 | Acute myoendocarditis NOS |
| G51z200 | Acute periendocarditis NOS |
| G51z300 | Subacute endocarditis NOS |
| G51z400 | Subacute myoendocarditis NOS |
| G51z500 | Subacute periendocarditis NOS |
| G51zz00 | Acute and subacute endocarditis unspecified, NOS |
| G52..00 | Acute myocarditis |
| G520.00 | Acute myocarditis in diseases EC |
| G520000 | Acute aseptic myocarditis of the newborn |
| G520100 | Acute myocarditis - coxsackie |
| G520200 | Acute myocarditis - diphtheritic |
| G520300 | Acute myocarditis - influenzal |
| G520400 | Acute myocarditis - syphilitic |
| G520500 | Acute myocarditis - toxoplasmosis |
| G520600 | Acute myocarditis - tuberculous |
| G520700 | Acute myocarditis - meningococcal |
| G520z00 | Acute myocarditis in diseases EC, NOS |
| G52y.00 | Other acute myocarditis |
| G52y000 | Acute myocarditis, unspecified |
| G52y100 | Isolated (Fiedler's) myocarditis |
| G52y111 | Giant cell myocarditis |
| G52y200 | Idiopathic myocarditis NOS |
| G52y300 | Septic myocarditis - pneumococcal |
| G52y400 | Septic myocarditis - staphylococcal |
| G52y500 | Septic myocarditis - streptococcal |
| G52y600 | Septic myocarditis NOS |
| G52y700 | Toxic myocarditis |
| G52yz00 | Other acute myocarditis NOS |
| G52z.00 | Acute myocarditis NOS |
| G53..00 | Other diseases of pericardium |
| G530.00 | Haemopericardium |
| G531.00 | Adhesive pericarditis |
| G531000 | Pericardial "milk spots" |
| G531100 | Fibrosis of pericardium |
| G531z00 | Adhesive pericarditis NOS |
| G532.00 | Constrictive pericarditis |
| G532000 | Concato's disease |
| G532100 | Pick's disease of heart |
| G532z00 | Constrictive pericarditis NOS |
| G533.00 | Pericardial effusion - noninflammatory |
| G534.00 | Pericardial effusion - acute |
| G53y.00 | Other diseases of pericardium OS |
| G53y000 | Calcification of pericardium |
| G53y100 | Fistula of pericardium |
| G53yz00 | Other specified pericardial disease NOS |
| G53yz11 | Chronic pericarditis |
| G53z.00 | Other pericardial disease NOS |
| G53z.11 | Cardiac tamponade |
| G53z000 | Non-traumatic pneumopericardium |
| G54..00 | Other diseases of endocardium |
| G54..11 | Heart valve disorders - non rheumatic |
| G540.00 | Mitral valve incompetence |
| G540.12 | Mitral valve insufficiency |
| G540.14 | Mitral valve regurgitation |
| G540.15 | Mitral valve prolapse |
| G540.16 | Mitral regurgitation |
| G540000 | Mitral incompetence, non-rheumatic |
| G540100 | Mitral incompetence, cause unspecified |
| G540200 | Mitral valve prolapse |
| G540300 | Mitral valve leaf prolapse |
| G540z00 | Mitral valve disorders NOS |
| G541.00 | Aortic valve disorders |
| G541000 | Aortic incompetence, non-rheumatic |
| G541011 | Aortic insufficiency, non-rheumatic |
| G541012 | Aortic regurgitation, non-rheumatic |
| G541100 | Aortic stenosis, non-rheumatic |
| G541200 | Aortic incompetence alone, cause unspecified |
| G541211 | Aortic insufficiency alone, cause unspecified |
| G541212 | Aortic regurgitation alone, cause unspecified |
| G541300 | Aortic stenosis alone, cause unspecified |
| G541400 | Aortic valve stenosis with insufficiency |
| G541500 | Aortic stenosis |
| G541600 | Aortic valve sclerosis |
| G541700 | Aortic valve calcification |
| G541z00 | Aortic valve disorders NOS |
| G542.00 | Tricuspid valve disorders, non-rheumatic |
| G542000 | Tricuspid incompetence, non-rheumatic |
| G542011 | Tricuspid insufficiency, non-rheumatic |
| G542012 | Tricuspid regurgitation, non-rheumatic |
| G542100 | Tricuspid stenosis, non-rheumatic |
| G542200 | Nonrheumatic tricuspid valve stenosis with insufficiency |
| G542X00 | Nonrheumatic tricuspid valve disorder, unspecified |
| G542z00 | Tricuspid valve disorders NOS |
| G543.00 | Pulmonary valve disorders |
| G543000 | Pulmonary incompetence, non-rheumatic |
| G543011 | Pulmonary insufficiency, non-rheumatic |
| G543012 | Pulmonary regurgitation, non-rheumatic |
| G543100 | Pulmonary stenosis, non-rheumatic |
| G543200 | Pulmonary incompetence, cause unspecified |
| G543213 | Pulmonary insufficiency, cause unspecified |
| G543215 | Pulmonary regurgitation, cause unspecified |
| G543300 | Pulmonary stenosis, cause unspecified |
| G543311 | Pulmonary stenosis, cause unspecified |
| G543400 | Pulmonary valve stenosis with insufficiency |
| G543z00 | Pulmonary valve disorders NOS |
| G544.00 | Multiple valve diseases |
| G544000 | Disorders of both aortic and tricuspid valves |
| G544100 | Disorders of both mitral and tricuspid valves |
| G544200 | Combined disorders of mitral, aortic and tricuspid valves |
| G544X00 | Multiple valve disease, unspecified |
| G54z.00 | Endocarditis, valve unspecified |
| G54z000 | Incompetence of unspecified heart valve |
| G54z013 | Regurgitation of unspecified heart valve |
| G54z014 | Insufficiency of unspecified heart valve |
| G54z100 | Stenosis of unspecified heart valve |
| G54z200 | Chronic cardiac valvulitis NOS |
| G54z300 | Endocarditis, valve unspecified, OS |
| G54z400 | Endocarditis in disease EC |
| G54z500 | Valvular heart disease |
| G54zz00 | Endocarditis, valve unspecified, NOS |
| G55..00 | Cardiomyopathy |
| G550.00 | Endomyocardial fibrosis |
| G551.00 | Hypertrophic obstructive cardiomyopathy |
| G552.00 | Obscure African cardiomyopathy |
| G552.11 | Becker's disease |
| G553.00 | Endocardial fibroelastosis |
| G554.00 | Other primary cardiomyopathies |
| G554000 | Congestive cardiomyopathy |
| G554011 | Congestive obstructive cardiomyopathy |
| G554100 | Constrictive cardiomyopathy |
| G554200 | Familial cardiomyopathy |
| G554300 | Hypertrophic non-obstructive cardiomyopathy |
| G554400 | Primary dilated cardiomyopathy |
| G554z00 | Other primary cardiomyopathy NOS |
| G555.00 | Alcoholic cardiomyopathy |
| G556.00 | Cardiomyopathy in Chagas's disease |
| G557.00 | Nutritional and metabolic cardiomyopathies |
| G557000 | Amyloid heart disease |
| G557100 | Beriberi heart disease |
| G557200 | Cardiac glycogenosis |
| G557300 | Gouty tophi of heart |
| G557400 | Mucopolysaccharidosis cardiomyopathy |
| G557500 | Thyrotoxic heart disease |
| G557z00 | Nutritional and metabolic cardiomyopathy NOS |
| G557z11 | Degeneration fatty heart |
| G557z12 | Fatty degeneration heart |
| G557z13 | Fatty infiltration heart |
| G558.00 | Cardiomyopathy in disease EC |
| G558000 | Cardiomyopathy in Friedreich's ataxia |
| G558100 | Cardiomyopathy in myotonic dystrophy |
| G558200 | Dystrophic cardiomyopathy |
| G558300 | Sarcoid heart disease |
| G558z00 | Cardiomyopathy in diseases EC, NOS |
| G55y.00 | Secondary cardiomyopathy NOS |
| G55y.11 | Secondary dilated cardiomyopathy |
| G55y000 | Cardiomyopathy due to drugs and other external agents |
| G55z.00 | Cardiomyopathy NOS |
| G56..00 | Conduction disorders |
| G56..11 | Conduction disorders of heart |
| G56..12 | Heart block |
| G560.00 | Complete atrioventricular block |
| G560.11 | Third degree atrioventricular block |
| G561.00 | Partial atrioventricular block |
| G561000 | Atrioventricular block unspecified |
| G561100 | First degree atrioventricular block |
| G561111 | Prolonged P-R interval |
| G561200 | Mobitz type II atrioventricular block |
| G561300 | Mobitz type I (Wenckebach) atrioventricular block |
| G561400 | Second degree atrioventricular block |
| G561z00 | Atrioventricular block NOS |
| G562.00 | Left bundle branch hemiblock |
| G562.11 | Left bundle branch block |
| G562000 | Left anterior fascicular block |
| G562100 | Left posterior fascicular block |
| G562z00 | Left bundle branch hemiblock NOS |
| G563.00 | Left main stem bundle branch block |
| G564.00 | Right bundle branch block |
| G565.00 | Other bundle branch block |
| G565000 | Bundle branch block unspecified |
| G565100 | Right BBB with left posterior fascicular block |
| G565200 | Right BBB with left anterior fascicular block |
| G565300 | Other bilateral bundle branch block |
| G565400 | Trifascicular block |
| G565500 | Bifascicular block |
| G565z00 | Other bundle branch block NOS |
| G566.00 | Other heart block |
| G566000 | Sinoatrial block |
| G566100 | Interventricular block NOS |
| G566200 | Right fascicular block |
| G566z00 | Other heart block NOS |
| G567.00 | Anomalous atrioventricular excitation |
| G567000 | Accelerated atrioventricular conduction |
| G567100 | Accessory atrioventricular conduction |
| G567200 | Pre-excitation atrioventricular conduction |
| G567300 | Ventricular pre-excitation |
| G567400 | Wolff-Parkinson-White syndrome |
| G567z00 | Anomalous atrioventricular excitation NOS |
| G56y.00 | Other conduction disorders |
| G56y000 | Lown-Ganong-Levine syndrome |
| G56y100 | Atrioventricular dissociation |
| G56y200 | Romano - Ward syndrome |
| G56y300 | Jervell and Lange-Nielsen syndrome |
| G56y400 | Right fascicular block |
| G56y500 | Long Q-T syndrome |
| G56yz00 | Other conduction disorders NOS |
| G56z.00 | Conduction disorders unspecified |
| G56z000 | Stokes-Adams syndrome |
| G56zz00 | Conduction disorders NOS |
| G57..00 | Cardiac dysrhythmias |
| G57..11 | Cardiac arrhythmias |
| G570.00 | Paroxysmal supraventricular tachycardia |
| G570000 | Paroxysmal atrial tachycardia |
| G570100 | Paroxysmal atrioventricular tachycardia |
| G570200 | Paroxysmal junctional tachycardia |
| G570300 | Paroxysmal nodal tachycardia |
| G570z00 | Paroxysmal supraventricular tachycardia NOS |
| G571.00 | Paroxysmal ventricular tachycardia |
| G571.11 | Ventricular tachycardia |
| G572.00 | Paroxysmal tachycardia unspecified |
| G572000 | Essential paroxysmal tachycardia |
| G572100 | Bouveret-Hoffmann syndrome |
| G572z00 | Paroxysmal tachycardia NOS |
| G573.00 | Atrial fibrillation and flutter |
| G573000 | Atrial fibrillation |
| G573100 | Atrial flutter |
| G573200 | Paroxysmal atrial fibrillation |
| G573300 | Non-rheumatic atrial fibrillation |
| G573z00 | Atrial fibrillation and flutter NOS |
| G574.00 | Ventricular fibrillation and flutter |
| G574000 | Ventricular fibrillation |
| G574011 | Cardiac arrest-ventricular fibrillation |
| G574100 | Ventricular flutter |
| G574z00 | Ventricular fibrillation and flutter NOS |
| G575.00 | Cardiac arrest |
| G575.11 | Cardio-respiratory arrest |
| G575.12 | Asystole |
| G575000 | Cardiac arrest with successful resuscitation |
| G575100 | Sudden cardiac death, so described |
| G575200 | Electromechanical dissociation with successful resuscitation |
| G575300 | Electromechanical dissociation |
| G575z00 | Cardiac arrest, unspecified |
| G576.00 | Ectopic beats |
| G576.11 | Premature beats |
| G576000 | Ectopic beats unspecified |
| G576011 | Extrasystoles |
| G576100 | Supraventricular ectopic beats |
| G576200 | Ventricular ectopic beats |
| G576300 | Atrial premature depolarization |
| G576400 | Junctional premature depolarization |
| G576500 | Ventricular premature depolarization |
| G576z00 | Ectopic beats NOS |
| G577.00 | Sinus arrhythmia |
| G57y.00 | Other cardiac dysrhythmias |
| G57y.11 | Pulsus alternans |
| G57y.12 | Pulse missed beats |
| G57y.13 | Skipped beat |
| G57y.14 | Heart beats irregular |
| G57y000 | Persistent sinus bradycardia |
| G57y100 | Severe sinus bradycardia |
| G57y300 | Sick sinus syndrome |
| G57y400 | Sinoatrial node dysfunction NOS |
| G57y500 | Wandering atrial pacemaker |
| G57y600 | Nodal rhythm disorder |
| G57y700 | Sinus tachycardia |
| G57y800 | Bigeminal pulse |
| G57y900 | Supraventricular tachycardia NOS |
| G57yA00 | Re-entry ventricular arrhythmia |
| G57yz00 | Other cardiac dysrhythmia NOS |
| G57z.00 | Cardiac dysrhythmia NOS |
| G58..00 | Heart failure |
| G58..11 | Cardiac failure |
| G580.00 | Congestive heart failure |
| G580.11 | Congestive cardiac failure |
| G580.12 | Right heart failure |
| G580.13 | Right ventricular failure |
| G580.14 | Biventricular failure |
| G580000 | Acute congestive heart failure |
| G580100 | Chronic congestive heart failure |
| G580200 | Decompensated cardiac failure |
| G580300 | Compensated cardiac failure |
| G581.00 | Left ventricular failure |
| G581.11 | Asthma - cardiac |
| G581.12 | Pulmonary oedema - acute |
| G581.13 | Impaired left ventricular function |
| G581000 | Acute left ventricular failure |
| G582.00 | Acute heart failure |
| G58z.00 | Heart failure NOS |
| G58z.11 | Weak heart |
| G58z.12 | Cardiac failure NOS |
| G5y..00 | Other specified heart disease |
| G5y0.00 | Myocarditis NOS |
| G5y1.00 | Myocardial degeneration |
| G5y2.00 | Cardiovascular arteriosclerosis unspecified |
| G5y3.00 | Cardiomegaly |
| G5y3.11 | Dilatation - cardiac |
| G5y3000 | Atrial dilatation |
| G5y3100 | Ventricular dilatation |
| G5y3200 | Cardiac dilatation NOS |
| G5y3300 | Atrial hypertrophy |
| G5y3400 | Ventricular hypertrophy |
| G5y3411 | Left ventricular hypertrophy |
| G5y3500 | Cardiac hypertrophy NOS |
| G5y3z00 | Cardiomegaly NOS |
| G5y4.00 | Post cardiac operation functional disturbance |
| G5y4000 | Postcardiotomy syndrome |
| G5y4z00 | Post cardiac operation heart failure NOS |
| G5y5.00 | Rupture of chordae tendinae |
| G5y6.00 | Rupture of papillary muscle |
| G5y7.00 | Sarcoid myocarditis |
| G5y8.00 | Rheumatoid myocarditis |
| G5y9.00 | Cardiac septal defect, acquired |
| G5yA.00 | Rheumatoid carditis |
| G5yX.00 | Cardiovascular disease, unspecified |
| G5yy.00 | Other ill-defined heart disease |
| G5yy.11 | Papillary muscle disease |
| G5yy000 | Papillary muscle atrophy |
| G5yy100 | Papillary muscle degeneration |
| G5yy200 | Papillary muscle dysfunction |
| G5yy300 | Papillary muscle scarring |
| G5yy400 | Papillary muscle disorder NOS |
| G5yy500 | Hyperkinetic heart disease |
| G5yy600 | Atrial thrombosis |
| G5yy700 | Left ventricular thrombosis |
| G5yy800 | Right ventricular thrombosis |
| G5yy900 | Left ventricular systolic dysfunction |
| G5yyA00 | Left ventricular diastolic dysfunction |
| G5yyz00 | Other ill-defined heart disease NOS |
| G5yz.00 | Other heart disease NOS |
| G5z..00 | Heart disease NOS |
| P5...00 | Bulbus cordis and cardiac septal closure anomalies |
| P5...11 | Cardiac septal defects |
| P5...12 | Congenital heart disease, septal and bulbar anomalies |
| P5...13 | Heart septal defects |
| P50..00 | Common aorto-pulmonary trunk |
| P50..11 | Aortic septal defect |
| P50..12 | Common truncus |
| P50..13 | Persistent truncus arteriosus |
| P500.00 | Absent septum between aorta and pulmonary artery |
| P500.11 | Persistent truncus arteriosus |
| P500.12 | Truncus arteriosus |
| P501.00 | Aortic septal defect |
| P501.11 | Aortopulmonary window |
| P501.12 | Aorticopulmonary septal defect |
| P502.00 | Persistent truncus arteriosus |
| P502.11 | Truncus arteriosus |
| P50z.00 | Common aorto-pulmonary trunk NOS |
| P51..00 | Transposition of great vessels |
| P510.00 | Total great vessel transposition |
| P511.00 | Double outlet right ventricle |
| P511000 | Double outlet right ventricle, unspecified |
| P511100 | Dextratransposition of aorta |
| P511200 | Incomplete great vessel transposition |
| P511300 | Taussig-Bing syndrome |
| P511z00 | Double outlet right ventricle NOS |
| P512.00 | Corrected great vessel transposition |
| P51y.00 | Other specified transposition of great vessels |
| P51y.11 | Transposition of aorta |
| P51z.00 | Great vessel transposition NOS |
| P51z.11 | Transposition of arterial trunk NEC |
| P52..00 | Tetralogy of Fallot |
| P520.00 | Tetralogy of Fallot, unspecified |
| P520.11 | Ventricular septal defect in Fallot's tetralogy |
| P520.12 | Dextraposition of aorta in Fallot's tetralogy |
| P521.00 | Pentalogy of Fallot |
| P52z.00 | Tetralogy of Fallot NOS |
| P53..00 | Common ventricle |
| P54..00 | Ventricular septal defect |
| P540.00 | Ventricular septal defect, unspecified |
| P541.00 | Interventricular septal defect |
| P542.00 | Left ventricle to right atrial communication |
| P543.00 | Eisenmenger's complex |
| P544.00 | Gerbode's defect |
| P545.00 | Roger's disease |
| P54y.00 | Other specified ventricular septal defect |
| P54z.00 | Ventricular septal defect NOS |
| P55..00 | Ostium secundum atrial septal defect |
| P550.00 | Atrial septal defect NOS |
| P550.11 | Auricular septal defect NOS |
| P550.12 | Interatrial septal defect NEC |
| P550.13 | Interauricular septal defect |
| P551.00 | Patent foramen ovale |
| P552.00 | Persistent ostium secundum |
| P552.11 | Patent ostium secundum |
| P553.00 | Lutembacher's syndrome |
| P55y.00 | Other specified ostium secundum atrial septal defect |
| P55y.11 | Other specified atrial septal defect |
| P55z.00 | Ostium secundum atrial septal defect NOS |
| P56..00 | Endocardial cushion defects |
| P560.00 | Endocardial cushion defects, unspecified |
| P561.00 | Ostium primum defect |
| P561.11 | Persistent ostium primum |
| P561.12 | Ostium primum type interauricular septal defect |
| P56y.00 | Other specified endocardial cushion defects |
| P56z.00 | Endocardial cushion defects NOS |
| P56z000 | Common atrium |
| P56z011 | Cor triloculare biventriculare |
| P56z100 | Common atrioventricular canal |
| P56z200 | Common atrioventricular-type ventricular septal defect |
| P56zz00 | Endocardial cushion defects NOS |
| P57..00 | Cor biloculare |
| P58..00 | Double outlet left ventricle |
| P59..00 | Isomerism of atrial appendages |
| P5X..00 | Congenital malforms of cardiac chambers+connections unsp |
| P5y..00 | Other heart bulb and septal closure defect |
| P5z..00 | Heart bulb or septal closure defects NOS |
| P6...00 | Other congenital heart anomalies |
| P60..00 | Pulmonary valve anomalies |
| P600.00 | Pulmonary valve anomaly, unspecified |
| P601.00 | Congenital atresia of the pulmonary valve |
| P601000 | Hypoplasia of pulmonary valve |
| P601z00 | Congenital atresia of pulmonary valve NOS |
| P602.00 | Congenital pulmonary stenosis |
| P602000 | Congenital fusion of pulmonic cusps |
| P602100 | Congenital fusion of pulmonary valve segment |
| P602z00 | Congenital pulmonary stenosis NOS |
| P603.00 | Right hypoplastic heart syndrome |
| P603.11 | Pseudotruncus arteriosus |
| P60z.00 | Other pulmonary valve anomalies |
| P60z000 | Congenital insufficiency of the pulmonary valve |
| P60z100 | Fallot's trilogy |
| P60z200 | Supernumerary pulmonary valve cusps |
| P60zz00 | Other pulmonary valve anomaly NOS |
| P61..00 | Congenital tricuspid atresia and stenosis |
| P610.00 | Congenital tricuspid atresia |
| P611.00 | Congenital tricuspid stenosis |
| P61z.00 | Congenital tricuspid atresia or stenosis NOS |
| P62..00 | Ebstein's anomaly |
| P63..00 | Congenital aortic valve stenosis |
| P64..00 | Congenital aortic valve insufficiency |
| P640.00 | Congenital aortic valve insufficiency, unspecified |
| P641.00 | Bicuspid aortic valve |
| P64z.00 | Congenital aortic valve insufficiency NOS |
| P65..00 | Congenital mitral stenosis |
| P65..11 | Duroziez's disease |
| P650.00 | Congenital mitral stenosis, unspecified |
| P651.00 | Fused commissure of the mitral valve |
| P652.00 | Parachute deformity of the mitral valve |
| P653.00 | Supernumerary cusps of the mitral valve |
| P65z.00 | Congenital mitral stenosis NOS |
| P66..00 | Congenital mitral insufficiency |
| P67..00 | Hypoplastic left heart syndrome |
| P6W..00 | Congenital malformation of aortic and mitral valves unsp |
| P6X..00 | Congenital malformation of tricuspid valve, unspecified |
| P6y..00 | Other specified heart anomalies |
| P6y0.00 | Subaortic stenosis |
| P6y1.00 | Cor triatriatum |
| P6y2.00 | Pulmonary infundibular stenosis |
| P6y3.00 | Obstructive heart anomaly NEC |
| P6y3000 | Uhl's disease |
| P6y3z00 | Obstructive heart anomaly NEC NOS |
| P6y4.00 | Coronary artery anomaly |
| P6y4000 | Congenital absence of coronary artery |
| P6y4100 | Single coronary artery |
| P6y4200 | Coronary artery from aorta |
| P6y4300 | Coronary artery from pulmonary trunk |
| P6y4400 | Anomalous coronary artery communication |
| P6y4411 | Congenital coronary arterio-venous fistula |
| P6y4500 | Congenital coronary aneurysm |
| P6y4600 | Congenital stricture of coronary artery |
| P6y4z00 | Coronary artery anomaly NOS |
| P6y5.00 | Congenital heart block |
| P6y5000 | Congenital heart block, unspecified |
| P6y5100 | Congenital complete atrio-ventricular heart block |
| P6y5200 | Congenital incomplete atrio-ventricular heart block |
| P6y5z00 | Congenital heart block NOS |
| P6y6.00 | Heart and cardiac apex malposition |
| P6y6.11 | Ectopic heart |
| P6y6000 | Dextrocardia |
| P6y6100 | Levocardia |
| P6y6111 | Laevocardia |
| P6y6200 | Mesocardia |
| P6y6300 | Ectopia cordis |
| P6y6400 | Abdominal heart |
| P6y6z00 | Heart or cardiac apex malposition NOS |
| P6y7.00 | Myocardial bridge of coronary artery |
| P6yy.00 | Other specified heart anomalies |
| P6yy.11 | Hypoplastic aortic orifice or valve |
| P6yy.12 | Hypoplasia of heart NOS |
| P6yy.13 | Congenital insufficiency of heart valve NEC |
| P6yy000 | Atresia of cardiac vein |
| P6yy100 | Hypoplasia of cardiac vein |
| P6yy200 | Congenital cardiomegaly |
| P6yy300 | Congenital left ventricular diverticulum |
| P6yy400 | Congenital pericardial defect |
| P6yy411 | Congenital absence of pericardium |
| P6yy500 | Congenital anomaly of myocardium |
| P6yy600 | Congenital aneurysm of heart |
| P6yy700 | Atresia of heart valve NEC |
| P6yy800 | Cor triloculare |
| P6yy900 | Congenital epicardial cyst |
| P6yyA00 | Hemicardia |
| P6yyB00 | Supernumerary heart valve cusps NEC |
| P6yyC00 | Fusion of mitral valve cusps |
| P6yyD00 | Fusion of heart valve cusps NEC |
| P6yyD11 | Fusion of tricuspid valve cusps NEC |
| P6yyz00 | Other specified heart anomalies NOS |
| P6z..00 | Congenital heart anomaly NOS |
| P6z..11 | Chiari's malformation |
| P6z0.00 | Unspecified anomaly of heart valve |
| P6z1.00 | Anomalous bands of heart |
| P6z1000 | Anomalous atrial bands |
| P6z1100 | Anomalous ventricular bands |
| P6z1z00 | Anomalous bands of heart NOS |
| P6z2.00 | Acyanotic congenital heart disease NOS |
| P6z3.00 | Cyanotic congenital heart disease NOS |
| P6z3.11 | Blue baby |
| P6zz.00 | Congenital heart anomaly NOS |
| P8...00 | Respiratory system congenital anomalies |
| P80..00 | Choanal atresia |
| P800.00 | Choanal atresia, unspecified |
| P801.00 | Atresia of the anterior nares |
| P802.00 | Atresia of the posterior nares |
| P803.00 | Congenital stenosis of the anterior nares |
| P804.00 | Congenital stenosis of the posterior nares |
| P80z.00 | Choanal atresia NOS |
| P81..00 | Other anomalies of nose |
| P810.00 | Congenital nose deformity, unspecified |
| P811.00 | Absent nose |
| P811.11 | Agenesis of nose |
| P811000 | Agenesis of nose |
| P811100 | Underdevelopment of nose |
| P811z00 | Absent nose NOS |
| P812.00 | Accessory nose |
| P813.00 | Congenital cleft nose |
| P814.00 | Deformity of nasal sinus wall |
| P815.00 | Congenital notching of tip of nose |
| P816.00 | Congenital perforation of the nasal sinus wall |
| P817.00 | Perforated nasal septum |
| P818.00 | Congenital fissure of nose |
| P819.00 | Congenital hypoplastic nose |
| P81z.00 | Other anomalies of nose NOS |
| P81z.11 | Single nostril |
| P82..00 | Congenital web of larynx |
| P820.00 | Congenital web of larynx, unspecified |
| P821.00 | Congenital glottic web of larynx |
| P822.00 | Congenital subglottic web of larynx |
| P82z.00 | Congenital web of larynx NOS |
| P83..00 | Other anomalies of larynx, trachea and bronchus |
| P830.00 | Agenesis of larynx, trachea and bronchus |
| P830000 | Agenesis of bronchus |
| P830100 | Agenesis of larynx |
| P830111 | Congenital absence of larynx |
| P830200 | Agenesis of trachea |
| P830211 | Congenital absence of trachea |
| P830z00 | Agenesis of larynx, trachea or bronchus NOS |
| P831.00 | Anomaly of laryngeal and tracheal cartilage |
| P831000 | Anomaly of cricoid cartilage |
| P831100 | Anomaly of epiglottis |
| P831200 | Anomaly of thyroid cartilage |
| P831300 | Anomaly of tracheal cartilage |
| P831400 | Tracheomalacia |
| P831500 | Laryngeal hypoplasia |
| P831600 | Laryngomalacia |
| P831z00 | Anomaly of laryngeal or tracheal cartilage NOS |
| P832.00 | Atresia of larynx and trachea |
| P832000 | Atresia of epiglottis |
| P832100 | Atresia of glottis |
| P832200 | Atresia of larynx |
| P832300 | Atresia of trachea |
| P832z00 | Atresia of larynx or trachea NOS |
| P833.00 | Congenital stenosis of larynx, trachea and bronchus |
| P833000 | Congenital stenosis of larynx |
| P833100 | Congenital stenosis of trachea |
| P833200 | Congenital stenosis of bronchus |
| P833300 | Congenital subglottic stenosis |
| P833400 | Congenital supraglottic stenosis |
| P833z00 | Congenital stenosis of larynx or trachea NOS |
| P83y.00 | Other anomaly of larynx, trachea and bronchus |
| P83y000 | Congenital cleft thyroid cartilage |
| P83y100 | Congenital dilatation of trachea |
| P83y200 | Congenital tracheocele |
| P83y300 | Congenital laryngocele |
| P83y400 | Congenital diverticulum of bronchus |
| P83y500 | Congenital diverticulum of trachea |
| P83y600 | Congenital fissure of epiglottis |
| P83y700 | Congenital cleft of posterior cricoid cartilage |
| P83y800 | Rudimentary tracheal bronchus |
| P83y900 | Congenital laryngeal stridor |
| P83yA00 | Congenital fissure of larynx |
| P83yB00 | Congenital bronchomalacia |
| P83yw00 | Other anomaly of larynx |
| P83yX00 | Congenital malformation of larynx, unspecified |
| P83yx00 | Other anomaly of trachea |
| P83yy00 | Other anomaly of bronchus |
| P83yz00 | Other anomaly of larynx, trachea or bronchus NOS |
| P83z.00 | Other anomalies of larynx, trachea or bronchus NOS |
| P84..00 | Congenital cystic lung |
| P840.00 | Congenital cystic lung disease, unspecified |
| P841.00 | Congenital polycystic lung |
| P841.11 | Multiple lung cysts |
| P841.12 | Multiple congenital bronchogenic cysts |
| P842.00 | Congenital honeycomb lung |
| P843.00 | Single lung cyst |
| P843.11 | Lung cyst |
| P843.12 | Congenital bronchogenic cyst |
| P84y.00 | Other specified congenital cystic lung |
| P84z.00 | Congenital cystic lung NOS |
| P85..00 | Lung agenesis, hypoplasia and dysplasia |
| P850.00 | Aplasia of lung |
| P851.00 | Hypoplasia of lung |
| P852.00 | Sequestration of lung |
| P853.00 | Agenesis of lung |
| P853.11 | Congenital absence of lung |
| P853000 | Congenital absence of lung fissures |
| P853100 | Congenital absence of lobe of lung |
| P853z00 | Agenesis of lung NOS |
| P85y.00 | Other specified lung agenesis, hypoplasia or dysplasia |
| P85y000 | Fusion of lobes of lung |
| P85yz00 | Other lung agenesis, hypoplasia or dysplasia NOS |
| P85z.00 | Lung agenesis, hypoplasia or dysplasia NOS |
| P86..00 | Other lung anomalies |
| P860.00 | Anomaly of lung, unspecified |
| P861.00 | Congenital bronchiectasis |
| P86y.00 | Other lung anomaly |
| P86y000 | Accessory lung |
| P86y100 | Azygos lobe of lung |
| P86y200 | Accessory lobe of lung |
| P86y300 | Ectopic tissues in lung |
| P86y311 | Ectopic bone and cartilage in lung |
| P86yz00 | Other lung anomaly NOS |
| P86z.00 | Lung anomaly NOS |
| P8y..00 | Other specified respiratory system anomalies |
| P8y0.00 | Abnormal pericardio-pleural communication |
| P8y1.00 | Anomaly, pleural folds |
| P8y2.00 | Atresia of nasopharynx |
| P8y3.00 | Congenital cyst of mediastinum |
| P8y4.00 | Congenital pulmonary lymphangiectasis |
| P8yz.00 | Other specified respiratory system anomaly NOS |
| P8z..00 | Respiratory system anomaly NOS |

Heart failure

| **Read Code** | **Description** |
| --- | --- |
| **Those we believe should be included** | |
| 1O1..00 | Heart failure confirmed |
| Q48y100 | Congenital cardiac failure |
| Q490.00 | Neonatal cardiac failure |

Hypertension

| **Read Code** | **Description** |
| --- | --- |
| **Those we believe should be included** | |
| 1JD..00 | Suspected hypertension |
| G8y3.00 | Chronic peripheral venous hypertension |
| Gyu2000 | [X]Other secondary hypertension |
| Gyu2100 | [X]Hypertension secondary to other renal disorders |
| J623.00 | Portal hypertension |
| Q492.00 | Neonatal hypertension |

## Central Nervous System Disease

Epilepsy

| **Read Code** | **Description** |
| --- | --- |
| **Those we believe should be included** | |
| F1...00 | Hereditary and degenerative diseases of the CNS |
| F250.00 | Generalised nonconvulsive epilepsy |
| F250000 | Petit mal (minor) epilepsy |
| F250011 | Epileptic absences |
| F250100 | Pykno-epilepsy |
| F250200 | Epileptic seizures - atonic |
| F250300 | Epileptic seizures - akinetic |
| F250400 | Juvenile absence epilepsy |
| F250500 | Lennox-Gastaut syndrome |
| F250y00 | Other specified generalised nonconvulsive epilepsy |
| F250z00 | Generalised nonconvulsive epilepsy NOS |
| F251.00 | Generalised convulsive epilepsy |
| F251000 | Grand mal (major) epilepsy |
| F251011 | Tonic-clonic epilepsy |
| F251100 | Neonatal myoclonic epilepsy |
| F251111 | Otohara syndrome |
| F251200 | Epileptic seizures - clonic |
| F251300 | Epileptic seizures - myoclonic |
| F251400 | Epileptic seizures - tonic |
| F251500 | Tonic-clonic epilepsy |
| F251600 | Grand mal seizure |
| F251y00 | Other specified generalised convulsive epilepsy |
| F251z00 | Generalised convulsive epilepsy NOS |
| F252.00 | Petit mal status |
| F253.00 | Grand mal status |
| F253.11 | Status epilepticus |
| F254.00 | Partial epilepsy with impairment of consciousness |
| F254000 | Temporal lobe epilepsy |
| F254100 | Psychomotor epilepsy |
| F254200 | Psychosensory epilepsy |
| F254300 | Limbic system epilepsy |
| F254400 | Epileptic automatism |
| F254500 | Complex partial epileptic seizure |
| F254z00 | Partial epilepsy with impairment of consciousness NOS |
| F255.00 | Partial epilepsy without impairment of consciousness |
| F255000 | Jacksonian, focal or motor epilepsy |
| F255011 | Focal epilepsy |
| F255012 | Motor epilepsy |
| F255100 | Sensory induced epilepsy |
| F255200 | Somatosensory epilepsy |
| F255300 | Visceral reflex epilepsy |
| F255311 | Partial epilepsy with autonomic symptoms |
| F255400 | Visual reflex epilepsy |
| F255500 | Unilateral epilepsy |
| F255600 | Simple partial epileptic seizure |
| F255y00 | Partial epilepsy without impairment of consciousness OS |
| F255z00 | Partial epilepsy without impairment of consciousness NOS |
| F256.00 | Infantile spasms |
| F256.11 | Lightning spasms |
| F256.12 | West syndrome |
| F256000 | Hypsarrhythmia |
| F256100 | Salaam attacks |
| F256z00 | Infantile spasms NOS |
| F257.00 | Kojevnikov's epilepsy |
| F258.00 | Post-ictal state |
| F259.00 | Early infant epileptic encephalopathy wth suppression bursts |
| F259.11 | Ohtahara syndrome |
| F25A.00 | Juvenile myoclonic epilepsy |
| F25B.00 | Alcohol-induced epilepsy |
| F25C.00 | Drug-induced epilepsy |
| F25D.00 | Menstrual epilepsy |
| F25E.00 | Stress-induced epilepsy |
| F25F.00 | Photosensitive epilepsy |
| F25X.00 | Status epilepticus, unspecified |
| F25y.00 | Other forms of epilepsy |
| F25y000 | Cursive (running) epilepsy |
| F25y100 | Gelastic epilepsy |
| F25y200 | Locl-rlt(foc)(part)idiop epilep&epilptic syn seiz locl onset |
| F25y300 | Complex partial status epilepticus |
| F25y400 | Benign Rolandic epilepsy |
| F25yz00 | Other forms of epilepsy NOS |
| F25z.00 | Epilepsy NOS |
| F25z.11 | Fit (in known epileptic) NOS |

MS/CNS (Vaccine High Risk)

| **Read Code** | **Description** |
| --- | --- |
| **Those we believe should be included** | |
| F1...00 | Hereditary and degenerative diseases of the CNS |
| F10..00 | Cerebral degenerations usually manifest in childhood |
| F100.00 | Leucodystrophy |
| F100000 | Krabbe's disease |
| F100100 | Schulz's disease |
| F100200 | Pelizaeus-Merzbacher disease |
| F100300 | Metachromatic leucodystrophy |
| F100z00 | Leucodystrophy NOS |
| F101.00 | Cerebral lipidoses |
| F101.11 | Amaurotic familial idiocy |
| F101000 | Jansky-Bielschowsky disease |
| F101100 | Kuf's disease |
| F101200 | Spielmeyer-Vogt (Batten) disease |
| F101211 | Batten's disease of retina |
| F101300 | Tay-Sach's disease |
| F101400 | Gangliosidosis |
| F101500 | Retinal dystrophy in cerebroretinal lipidosis |
| F101z00 | Cerebral lipidoses NOS |
| F102.00 | Cerebral degeneration in lipidoses EC |
| F102000 | Cerebral degeneration in Gaucher's disease |
| F102100 | Cerebral degeneration in Niemann-Pick disease |
| F102z00 | Cerebral degeneration in lipidosis NOS |
| F103.00 | Cerebral degeneration in diseases EC |
| F103000 | Cerebral degeneration in Hunter's disease |
| F103100 | Cerebral degeneration in mucopolysaccharidoses |
| F103z00 | Cerebral degeneration in disease NOS |
| F10y.00 | Other cerebral degenerations in childhood |
| F10y000 | Alper's disease |
| F10y100 | Leigh's disease |
| F10yz00 | Other cerebral degenerations in childhood NOS |
| F10z.00 | Childhood cerebral degenerations NOS |
| F11..00 | Other cerebral degenerations |
| F110.00 | Alzheimer's disease |
| F110000 | Alzheimer's disease with early onset |
| F110100 | Alzheimer's disease with late onset |
| F111.00 | Pick's disease |
| F112.00 | Senile degeneration of brain |
| F113.00 | Acquired communicating hydrocephalus |
| F113000 | Normal pressure hydrocephalus |
| F113011 | Low pressure hydrocephalus |
| F113z00 | Communicating hydrocephalus - acquired NOS |
| F114.00 | Acquired obstructive hydrocephalus |
| F115.00 | Hydrocephalus |
| F116.00 | Lewy body disease |
| F117.00 | Infantile posthaemorrhagic hydrocephalus |
| F11x.00 | Cerebral degeneration in other disease EC |
| F11X.00 | Post-traumatic hydrocephalus, unspecified |
| F11x000 | Cerebral degeneration due to alcoholism |
| F11x011 | Alcoholic encephalopathy |
| F11x100 | Cerebral degeneration due to beriberi |
| F11x200 | Cerebral degeneration due to cerebrovascular disease |
| F11x300 | Cerebral degeneration due to congenital hydrocephalus |
| F11x400 | Cerebral degeneration due to neoplastic disease |
| F11x500 | Cerebral degeneration due to myxoedema |
| F11x600 | Cerebral degeneration due to vitamin B12 deficiency |
| F11x700 | Cerebral degeneration due to Jakob - Creutzfeldt disease |
| F11x800 | Cerebral degeneration due to multifocal leucoencephalopathy |
| F11xz00 | Cerebral degeneration other disease NOS |
| F11y.00 | Other cerebral degeneration |
| F11y000 | Reye's syndrome |
| F11y100 | Cerebral ataxia |
| F11yz00 | Other cerebral degeneration NOS |
| F11z.00 | Cerebral degeneration NOS |
| F11z.11 | Cerebral atrophy |
| F12..00 | Parkinson's disease |
| F120.00 | Paralysis agitans |
| F121.00 | Parkinsonism secondary to drugs |
| F121.11 | Drug induced parkinsonism |
| F122.00 | Malignant neuroleptic syndrome |
| F123.00 | Postencephalitic parkinsonism |
| F12W.00 | Secondary parkinsonism due to other external agents |
| F12X.00 | Secondary parkinsonism, unspecified |
| F12z.00 | Parkinson's disease NOS |
| F13..00 | Other extrapyramidal disease and abnormal movement disorders |
| F13..11 | Extrapyramidal disease excluding Parkinson's disease |
| F130.00 | Other basal ganglia degenerative diseases |
| F130000 | Dejerine-Thomas syndrome |
| F130100 | Hallervorden-Spatz disease |
| F130200 | Striatonigral degeneration |
| F130300 | Parkinsonism with orthostatic hypotension |
| F130400 | Progressive supranuclear ophthalmoplegia |
| F130500 | Shy-Drager syndrome |
| F130600 | Aicardi Goutieres syndrome |
| F130z00 | Other basal ganglia degenerative disease NOS |
| F131.00 | Essential and other specified forms of tremor |
| F131000 | Benign essential tremor |
| F131100 | Familial tremor |
| F131200 | Drug-induced tremor |
| F131z00 | Essential and other specified forms of tremor NOS |
| F132.00 | Myoclonus |
| F132000 | Familial essential myoclonus |
| F132100 | Progressive myoclonic epilepsy |
| F132111 | Unverricht - Lundborg disease |
| F132200 | Myoclonic encephalopathy |
| F132300 | Myoclonic jerks |
| F132y00 | Other specified myoclonus |
| F132y11 | Paramyoclonus multiplex |
| F132z00 | Myoclonus NOS |
| F132z11 | Unverricht - Lundborg disease |
| F132z12 | Myoclonic seizure |
| F133.00 | Tics of organic origin |
| F134.00 | Huntington's chorea |
| F135.00 | Other choreas |
| F135000 | Hemiballismus |
| F135100 | Paroxysmal choreo-athetosis |
| F135200 | Drug-induced chorea |
| F135z00 | Other choreas NOS |
| F136.00 | Idiopathic torsion dystonia |
| F136000 | Idiopathic familial dystonia |
| F137.00 | Symptomatic torsion dystonia |
| F137.11 | Athetoid cerebral palsy |
| F137.12 | Athetosis - congenital |
| F137.13 | Vogt's disease |
| F137000 | Athetoid cerebral palsy |
| F137011 | Vogt's disease |
| F137100 | Double athetosis |
| F137111 | Congenital athetosis |
| F137200 | Drug-induced dystonia |
| F137y00 | Other specified symptomatic torsion dystonia |
| F137z00 | Symptomatic torsion dystonia NOS |
| F138.00 | Fragments of torsion dystonia |
| F138000 | Blepharospasm |
| F138100 | Orofacial dyskinesia |
| F138111 | Tardive dyskinesia |
| F138200 | Spasmodic torticollis |
| F138300 | Organic writers' cramp |
| F138z00 | Fragments of torsion dystonia NOS |
| F139.00 | Paroxysmal dyskinesia |
| F139000 | Paroxysmal non-kinesigenic dyskinesia |
| F139100 | Paroxysmal kinesigenic dyskinesia |
| F13A.00 | Paroxysmal dystonia |
| F13X.00 | Dystonia, unspecified |
| F13z.00 | Other/unspecified extrapyramidal/abnormal movement disorders |
| F13z000 | Unspecified extrapyramidal disease |
| F13z100 | Stiff-man syndrome |
| F13z200 | Restless legs syndrome |
| F13z300 | Akinetic rigid syndrome |
| F13z400 | Hyperekplexia |
| F13z500 | Benign neonatal sleep myoclonus |
| F13zz00 | Extrapyramidal disease and abnormal movement disorder NOS |
| F14..00 | Spinocerebellar disease |
| F14..11 | Cerebellar disease |
| F140.00 | Friedreich's ataxia |
| F141.00 | Hereditary spastic paraplegia |
| F142.00 | Primary cerebellar degeneration |
| F142000 | Marie's cerebellar ataxia |
| F142100 | Sanger-Brown cerebellar ataxia |
| F142200 | Dyssynergia cerebellaris myoclonica |
| F142z00 | Primary cerebellar degeneration NOS |
| F143.00 | Cerebellar ataxia NOS |
| F143.11 | Roussy-Levy syndrome |
| F144.00 | Cerebellar ataxia in diseases EC |
| F144000 | Cerebellar ataxia due to alcoholism |
| F144100 | Cerebellar ataxia due to myxoedema |
| F144200 | Cerebellar ataxia due to neoplasia |
| F144z00 | Cerebellar ataxia in disease NOS |
| F145.00 | Congenital nonprogressive ataxia |
| F14y.00 | Other spinocerebellar diseases |
| F14y000 | Ataxia-telangiectasia |
| F14y011 | Louis - Bar syndrome |
| F14y100 | Corticostriatal-spinal degeneration |
| F14yz00 | Other spinocerebellar disease NOS |
| F14z.00 | Spinocerebellar disease NOS |
| F15..00 | Anterior horn cell disease |
| F150.00 | Werdnig - Hoffmann disease |
| F150.11 | Infantile spinal muscular atrophy |
| F151.00 | Spinal muscular atrophy |
| F151000 | Unspecified spinal muscular atrophy |
| F151100 | Kugelberg - Welander disease |
| F151111 | Juvenile spinal muscular atrophy |
| F151200 | Adult spinal muscular atrophy |
| F151300 | X-linked bulbo-spinal atrophy |
| F151z00 | Spinal muscular atrophy NOS |
| F152.00 | Motor neurone disease |
| F152000 | Amyotrophic lateral sclerosis |
| F152100 | Progressive muscular atrophy |
| F152111 | Duchenne Aran muscular atrophy |
| F152200 | Progressive bulbar palsy |
| F152300 | Pseudobulbar palsy |
| F152400 | Primary lateral sclerosis |
| F152z00 | Motor neurone disease NOS |
| F15y.00 | Other anterior horn cell disease |
| F15z.00 | Anterior horn cell disease NOS |
| F16..00 | Other diseases of spinal cord |
| F160.00 | Syringomyelia and syringobulbia |
| F160000 | Syringomyelia |
| F160100 | Syringobulbia |
| F160z00 | Syringomyelia or syringobulbia NOS |
| F161.00 | Vascular myelopathies |
| F161000 | Myelopathy due to acute infarction of spinal cord |
| F161100 | Myelopathy due to arterial thrombosis of spinal cord |
| F161200 | Myelopathy due to oedema of spinal cord |
| F161300 | Myelopathy due to haematomyelia |
| F161400 | Subacute necrotic myelopathy |
| F161500 | Anterior spinal artery thrombosis |
| F161z00 | Vascular myelopathy NOS |
| F162.00 | Subacute combined degeneration of spinal cord |
| F163.00 | Myelopathy due to disease EC |
| F163000 | Myelopathy due to intervertebral disc disease |
| F163100 | Myelopathy due to neoplastic disease |
| F163200 | Myelopathy due to spondylosis |
| F163z00 | Myelopathy due to disease NOS |
| F16y.00 | Other myelopathy |
| F16y000 | Drug induced myelopathy |
| F16y100 | Radiation induced myelopathy |
| F16yz00 | Other myelopathy NOS |
| F16z.00 | Myelopathy NOS |
| F16z.11 | Cord compression NOS |
| F16z.12 | Spinal cord compression NOS |
| F17..00 | Autonomic nervous system disorders |
| F170.00 | Idiopathic peripheral autonomic neuropathy |
| F170000 | Carotid sinus syndrome |
| F170100 | Cervical sympathetic paralysis |
| F170z00 | Idiopathic peripheral autonomic neuropathy NOS |
| F171.00 | Peripheral autonomic neuropathy disease EC |
| F171000 | Autonomic neuropathy due to amyloid |
| F171100 | Autonomic neuropathy due to diabetes |
| F171z00 | Peripheral autonomic neuropathy due to disease NOS |
| F172.00 | [X] Horners syndrome |
| F173.00 | Shoulder-hand syndrome |
| F174.00 | Multiple system atrophy |
| F17z.00 | Autonomic nervous system disorder NOS |
| F17z.11 | Horner's syndrome |
| F17z.12 | Autonomic failure |
| F1y..00 | Hereditary and degenerative diseases of the CNS OS |
| F1z..00 | Hereditary and degenerative diseases of the CNS NOS |
| F20..00 | Multiple sclerosis |
| F20..11 | Disseminated sclerosis |
| F200.00 | Multiple sclerosis of the brain stem |
| F201.00 | Multiple sclerosis of the spinal cord |
| F202.00 | Generalised multiple sclerosis |
| F203.00 | Exacerbation of multiple sclerosis |
| F20z.00 | Multiple sclerosis NOS |
| F21..00 | Other central nervous system demyelinating diseases |
| F210.00 | Neuromyelitis optica |
| F210.11 | Devic's disease |
| F211.00 | Schilder's disease |
| F211.11 | Balo's concentric sclerosis |
| F212.00 | Acute and subacute haemorrhagic leukoencephalitis [Hurst] |
| F21X.00 | Acute disseminated demyelination, unspecified |
| F21y.00 | Other specified central nervous system demyelinating disease |
| F21y000 | Marchiafava-Bignami disease |
| F21y100 | Central pontine myelinosis |
| F21y200 | Binswanger's disease |
| F21y211 | Binswanger's encephalopathy |
| F21y300 | Central demyelination of corpus callosum |
| F21y400 | Subacute necrotizing myelitis |
| F21y500 | Concentric sclerosis |
| F21yz00 | Other specified central nervous system demyelination NOS |
| F21z.00 | Central nervous system demyelination NOS |
| F22..00 | Hemiplegia |
| F22..11 | Hemiparesis |
| F220.00 | Flaccid hemiplegia |
| F221.00 | Spastic hemiplegia |
| F221.11 | Spastic foot |
| F222.00 | Left hemiplegia |
| F222.11 | Left sided weakness |
| F223.00 | Right hemiplegia |
| F223.11 | Right sided weakness |
| F22z.00 | Hemiplegia NOS |
| F23..00 | Congenital cerebral palsy |
| F23..11 | Congenital spastic cerebral palsy |
| F23..12 | Infantile cerebral palsy |
| F23..13 | Littles disease |
| F23..14 | Cerebral atonia |
| F230.00 | Congenital diplegia |
| F230.11 | Paraplegia - congenital |
| F230000 | Congenital paraplegia |
| F230100 | Cerebral palsy with spastic diplegia |
| F230z00 | Congenital diplegia NOS |
| F231.00 | Congenital hemiplegia |
| F232.00 | Congenital quadriplegia |
| F232.11 | Tetraplegia - congenital |
| F233.00 | Congenital monoplegia |
| F233.11 | Congenital spastic foot |
| F234.00 | Infantile hemiplegia NOS |
| F23y.00 | Other congenital cerebral palsy |
| F23y000 | Ataxic infantile cerebral palsy |
| F23y100 | Flaccid infantile cerebral palsy |
| F23y200 | Spastic cerebral palsy |
| F23y300 | Dyskinetic cerebral palsy |
| F23y400 | Ataxic diplegic cerebral palsy |
| F23yz00 | Other infantile cerebral palsy NOS |
| F23z.00 | Congenital cerebral palsy NOS |
| F24..00 | Other paralytic syndromes |
| F240.00 | Quadriplegia |
| F240.11 | Tetraplegia |
| F240000 | Flaccid tetraplegia |
| F240100 | Spastic tetraplegia |
| F241.00 | Paraplegia |
| F241000 | Flaccid paraplegia |
| F241100 | Spastic paraplegia |
| F242.00 | Diplegia of upper limbs |
| F24y.00 | Other specified paralytic syndromes |
| F24y000 | Progressive supranuclear palsy |
| F24y011 | Steele Richardson Olszewsk syn |
| F24y012 | Steele - Richardson Oszewski syndrome |
| F24y100 | Todd's paralysis |
| F24y200 | Steele-Richardson-Olszewski syndrome |
| F24yz00 | Other paralytic syndromes NOS |
| F24yz11 | Specified palsy NEC |
| F24z.00 | Paralysis NOS |
| F2A..00 | Hemiparesis |
| F2Az.00 | Hemiparesis NOS |
| Fyu9.00 | [X]Cerebral palsy and other paralytic syndromes |
| Fyu9000 | [X]Other infantile cerebral palsy |
| Fyu9100 | [X]Other specified paralytic syndromes |

Psychosis

| **Read Code** | **Description** |
| --- | --- |
| **Those we believe should be included** | |
| E00y.00 | Other senile and presenile organic psychoses |
| E00y.11 | Presbyophrenic psychosis |
| E00z.00 | Senile or presenile psychoses NOS |
| E03..00 | Transient organic psychoses |
| E03y.00 | Other transient organic psychoses |
| E03yz00 | Other transient organic psychoses NOS |
| E03z.00 | Transient organic psychoses NOS |
| E04..00 | Other chronic organic psychoses |
| E04y.00 | Other specified chronic organic psychoses |
| E04z.00 | Chronic organic psychosis NOS |
| E0y..00 | Other specified organic psychoses |
| E0z..00 | Organic psychoses NOS |
| Eu02z12 | [X] Presenile psychosis NOS |
| Eu02z15 | [X] Senile psychosis NOS |
| Eu04.13 | [X]Acute / subacute infective psychosis |
| Eu0z.11 | [X]Organic psychosis NOS |
| Eu0z.12 | [X]Symptomatic psychosis NOS |
| E11..00 | Affective psychoses |
| E11..11 | Bipolar psychoses |
| E11..12 | Depressive psychoses |
| E11..13 | Manic psychoses |
| E110.11 | Hypomanic psychoses |
| E110300 | Single manic episode, severe without mention of psychosis |
| E110400 | Single manic episode, severe, with psychosis |
| E111300 | Recurrent manic episodes, severe without mention psychosis |
| E111400 | Recurrent manic episodes, severe, with psychosis |
| E112300 | Single major depressive episode, severe, without psychosis |
| E112400 | Single major depressive episode, severe, with psychosis |
| E113300 | Recurrent major depressive episodes, severe, no psychosis |
| E113400 | Recurrent major depressive episodes, severe, with psychosis |
| E114300 | Bipolar affect disord, currently manic, severe, no psychosis |
| E114400 | Bipolar affect disord, currently manic,severe with psychosis |
| E115300 | Bipolar affect disord, now depressed, severe, no psychosis |
| E115400 | Bipolar affect disord, now depressed, severe with psychosis |
| E116300 | Mixed bipolar affective disorder, severe, without psychosis |
| E116400 | Mixed bipolar affective disorder, severe, with psychosis |
| E117300 | Unspecified bipolar affective disorder, severe, no psychosis |
| E117400 | Unspecified bipolar affective disorder,severe with psychosis |
| E11y.00 | Other and unspecified manic-depressive psychoses |
| E11y000 | Unspecified manic-depressive psychoses |
| E11y300 | Other mixed manic-depressive psychoses |
| E11yz00 | Other and unspecified manic-depressive psychoses NOS |
| E11z.00 | Other and unspecified affective psychoses |
| E11z000 | Unspecified affective psychoses NOS |
| E11zz00 | Other affective psychosis NOS |
| E121.00 | Chronic paranoid psychosis |
| E12z.00 | Paranoid psychosis NOS |
| E13..00 | Other nonorganic psychoses |
| E13..11 | Reactive psychoses |
| E130.00 | Reactive depressive psychosis |
| E131.00 | Acute hysterical psychosis |
| E134.00 | Psychogenic paranoid psychosis |
| E13y.00 | Other reactive psychoses |
| E13y100 | Brief reactive psychosis |
| E13yz00 | Other reactive psychoses NOS |
| E13z.00 | Nonorganic psychosis NOS |
| E14..00 | Psychoses with origin in childhood |
| E141.00 | Disintegrative psychosis |
| E141000 | Active disintegrative psychoses |
| E141100 | Residual disintegrative psychoses |
| E141z00 | Disintegrative psychosis NOS |
| E14y.00 | Other childhood psychoses |
| E14y000 | Atypical childhood psychoses |
| E14y100 | Borderline psychosis of childhood |
| E14yz00 | Other childhood psychoses NOS |
| E14z.00 | Child psychosis NOS |
| E1y..00 | Other specified non-organic psychoses |
| E1z..00 | Non-organic psychosis NOS |
| Eu22011 | [X]Paranoid psychosis |
| Eu23012 | [X]Cycloid psychosis |
| Eu23112 | [X]Cycloid psychosis with symptoms of schizophrenia |
| Eu23312 | [X]Psychogenic paranoid psychosis |
| Eu23z11 | [X]Brief reactive psychosis NOS |
| Eu23z12 | [X]Reactive psychosis |
| Eu25011 | [X]Schizoaffective psychosis, manic type |
| Eu25012 | [X]Schizophreniform psychosis, manic type |
| Eu25111 | [X]Schizoaffective psychosis, depressive type |
| Eu25112 | [X]Schizophreniform psychosis, depressive type |
| Eu25212 | [X]Mixed schizophrenic and affective psychosis |
| Eu25z11 | [X]Schizoaffective psychosis NOS |
| Eu2y.11 | [X]Chronic hallucinatory psychosis |
| Eu2z.00 | [X]Unspecified nonorganic psychosis |
| Eu2z.11 | [X]Psychosis NOS |
| Eu31.12 | [X]Manic-depressive psychosis |
| Eu32312 | [X]Single episode of psychogenic depressive psychosis |
| Eu32314 | [X]Single episode of reactive depressive psychosis |
| Eu33213 | [X]Manic-depress psychosis,depressd,no psychotic symptoms |
| Eu33312 | [X]Manic-depress psychosis,depressed type+psychotic symptoms |
| Eu33314 | [X]Recurr severe episodes/psychogenic depressive psychosis |
| Eu33316 | [X]Recurrent severe episodes/reactive depressive psychosis |
| Eu3z.11 | [X]Affective psychosis NOS |
| Eu44.14 | [X]Hysterical psychosis |
| Eu53111 | [X]Puerperal psychosis NOS |
| Eu84013 | [X]Infantile psychosis |
| Eu84111 | [X]Atypical childhood psychosis |
| Eu84312 | [X]Disintegrative psychosis |
| Eu84314 | [X]Symbiotic psychosis |
| 146H.00 | H/O: psychosis |

## Cerebrovascular Disease

Cerebrovascular disease

| **Read Code** | **Description** |
| --- | --- |
| **Those we believe should be included** | |
| 1JA1.00 | Suspected cerebrovascular disease |
| G6...00 | Cerebrovascular disease |
| G67..00 | Other cerebrovascular disease |
| G671.00 | Generalised ischaemic cerebrovascular disease NOS |
| G671000 | Acute cerebrovascular insufficiency NOS |
| G671z00 | Generalised ischaemic cerebrovascular disease NOS |
| G67y.00 | Other cerebrovascular disease OS |
| G67z.00 | Other cerebrovascular disease NOS |
| G68..00 | Late effects of cerebrovascular disease |
| G68W.00 | Sequelae/other + unspecified cerebrovascular diseases |
| G68X.00 | Sequelae of stroke,not specfd as h'morrhage or infarction |
| G6y..00 | Other specified cerebrovascular disease |
| G6z..00 | Cerebrovascular disease NOS |
| 8HBJ.00 | Stroke / transient ischaemic attack referral |
| 8HHM.00 | Ref to multidisciplinary stroke function improvement service |
| 8HTQ.00 | Referral to stroke clinic |
| L440.00 | Cerebrovascular disorders in the puerperium |
| L440.11 | CVA - cerebrovascular accident in the puerperium |
| L440.12 | Stroke in the puerperium |
| L440000 | Puerperal cerebrovascular disorder unspecified |
| L440100 | Puerperal cerebrovascular disorder - delivered |
| L440z00 | Puerperal cerebrovascular disorder NOS |
| P7y0.00 | Cerebrovascular system anomalies |
| P7y0y00 | Other specified cerebrovascular anomaly |
| P7y0z00 | Cerebrovascular system anomaly NOS |

Stroke/TIA (Vaccine High Risk)

| **Read Code** | **Description** |
| --- | --- |
| **Those we believe should be included** | |
| G61..00 | Intracerebral haemorrhage |
| G61..11 | CVA - cerebrovascular accid due to intracerebral haemorrhage |
| G61..12 | Stroke due to intracerebral haemorrhage |
| G610.00 | Cortical haemorrhage |
| G611.00 | Internal capsule haemorrhage |
| G612.00 | Basal nucleus haemorrhage |
| G613.00 | Cerebellar haemorrhage |
| G614.00 | Pontine haemorrhage |
| G615.00 | Bulbar haemorrhage |
| G616.00 | External capsule haemorrhage |
| G617.00 | Intracerebral haemorrhage, intraventricular |
| G618.00 | Intracerebral haemorrhage, multiple localized |
| G61X.00 | Intracerebral haemorrhage in hemisphere, unspecified |
| G61X000 | Left sided intracerebral haemorrhage, unspecified |
| G61X100 | Right sided intracerebral haemorrhage, unspecified |
| G61z.00 | Intracerebral haemorrhage NOS |
| G63y000 | Cerebral infarct due to thrombosis of precerebral arteries |
| G63y100 | Cerebral infarction due to embolism of precerebral arteries |
| G64..00 | Cerebral arterial occlusion |
| G64..11 | CVA - cerebral artery occlusion |
| G64..12 | Infarction - cerebral |
| G64..13 | Stroke due to cerebral arterial occlusion |
| G640.00 | Cerebral thrombosis |
| G640000 | Cerebral infarction due to thrombosis of cerebral arteries |
| G641.00 | Cerebral embolism |
| G641.11 | Cerebral embolus |
| G641000 | Cerebral infarction due to embolism of cerebral arteries |
| G64z.00 | Cerebral infarction NOS |
| G64z.11 | Brainstem infarction NOS |
| G64z.12 | Cerebellar infarction |
| G64z000 | Brainstem infarction |
| G64z100 | Wallenberg syndrome |
| G64z111 | Lateral medullary syndrome |
| G64z200 | Left sided cerebral infarction |
| G64z300 | Right sided cerebral infarction |
| G64z400 | Infarction of basal ganglia |
| G65..00 | Transient cerebral ischaemia |
| G65..11 | Drop attack |
| G65..12 | Transient ischaemic attack |
| G65..13 | Vertebro-basilar insufficiency |
| G650.00 | Basilar artery syndrome |
| G650.11 | Insufficiency - basilar artery |
| G651.00 | Vertebral artery syndrome |
| G651000 | Vertebro-basilar artery syndrome |
| G652.00 | Subclavian steal syndrome |
| G653.00 | Carotid artery syndrome hemispheric |
| G654.00 | Multiple and bilateral precerebral artery syndromes |
| G655.00 | Transient global amnesia |
| G656.00 | Vertebrobasilar insufficiency |
| G65y.00 | Other transient cerebral ischaemia |
| G65z.00 | Transient cerebral ischaemia NOS |
| G65z000 | Impending cerebral ischaemia |
| G65z100 | Intermittent cerebral ischaemia |
| G65zz00 | Transient cerebral ischaemia NOS |
| G66..00 | Stroke and cerebrovascular accident unspecified |
| G66..11 | CVA unspecified |
| G66..12 | Stroke unspecified |
| G66..13 | CVA - Cerebrovascular accident unspecified |
| G660.00 | Middle cerebral artery syndrome |
| G661.00 | Anterior cerebral artery syndrome |
| G662.00 | Posterior cerebral artery syndrome |
| G663.00 | Brain stem stroke syndrome |
| G664.00 | Cerebellar stroke syndrome |
| G665.00 | Pure motor lacunar syndrome |
| G666.00 | Pure sensory lacunar syndrome |
| G667.00 | Left sided CVA |
| G668.00 | Right sided CVA |
| G669.00 | Cerebral palsy, not congenital or infantile, acute |
| G676000 | Cereb infarct due cerebral venous thrombosis, nonpyogenic |
| G6W..00 | Cereb infarct due unsp occlus/stenos precerebr arteries |
| G6X..00 | Cerebrl infarctn due/unspcf occlusn or sten/cerebrl artrs |
| Gyu6.00 | [X]Cerebrovascular diseases |
| Gyu6000 | [X]Subarachnoid haemorrhage from other intracranial arteries |
| Gyu6100 | [X]Other subarachnoid haemorrhage |
| Gyu6200 | [X]Other intracerebral haemorrhage |
| Gyu6300 | [X]Cerebrl infarctn due/unspcf occlusn or sten/cerebrl artrs |
| Gyu6400 | [X]Other cerebral infarction |
| Gyu6500 | [X]Occlusion and stenosis of other precerebral arteries |
| Gyu6600 | [X]Occlusion and stenosis of other cerebral arteries |
| Gyu6700 | [X]Other specified cerebrovascular diseases |
| Gyu6800 | [X]Cerebral arteritis in infectious and parasitic diseases |
| Gyu6900 | [X]Cerebral arteritis in other diseases CE |
| Gyu6A00 | [X]Other cerebrovascular disorders in diseases CE |
| Gyu6B00 | [X]Sequelae of other nontraumatic intracranial haemorrhage |
| Gyu6C00 | [X]Sequelae of stroke,not specfd as h'morrhage or infarction |
| Gyu6D00 | [X]Sequelae/other + unspecified cerebrovascular diseases |
| Gyu6E00 | [X]Subarachnoid haemorrh from intracranial artery, unspecif |
| Gyu6F00 | [X]Intracerebral haemorrhage in hemisphere, unspecified |
| Gyu6G00 | [X]Cereb infarct due unsp occlus/stenos precerebr arteries |

## Chronic Otitis Media

Chronic otitis media

| **Read Code** | **Description** |
| --- | --- |
| **Those we believe should be included** | |
| **Those that could be considered relevant that we don’t believe should be used** | |
| F511.00 | Chronic otitis media with effusion, serous |
| F511.11 | Chronic secretory otitis media, serous |
| F511100 | Serosanguinous chronic otitis media |
| F511z00 | Chronic serous otitis media NOS |
| F512.00 | Chronic otitis media with effusion, mucoid |
| F512.12 | Chronic secretory otitis media, mucoid |
| F512100 | Mucosanguinous chronic otitis media |
| F512z00 | Chronic mucoid otitis media NOS |
| F513.00 | Chronic otitis media with effusion, other |
| F513000 | Chronic allergic otitis media |
| F513100 | Chronic otitis media with effusion, purulent |
| F513111 | Chronic secretory otitis media, purulent |
| F513z00 | Other chronic nonsuppurative otitis media NOS |
| F518.00 | Chronic otitis media with effusion, unspecified |
| F521.00 | Chronic suppurative otitis media, tubotympanic |
| F522.00 | Chronic suppurative otitis media, atticoantral |
| F523.00 | Chronic suppurative otitis media NOS |
| F541.00 | Chronic myringitis without mention of otitis media |
| FyuP100 | [X]Other chronic nonsuppurative otitis media |
| FyuP200 | [X]Other chronic suppurative otitis media |

## Diabetes

Diabetes

| **Read Code** | **Description** |
| --- | --- |
| **Those we believe should be included** | |
| 66AJ.11 | Unstable diabetes |
| 66AJ100 | Brittle diabetes |
| 8H2J.00 | Admit diabetic emergency |
| 8H3O.00 | Non-urgent diabetic admission |
| C135.00 | Diabetes insipidus |
| C135.12 | Diabetes insipidus - pituitary |
| C314.11 | Renal diabetes |
| C350011 | Bronzed diabetes |
| Cyu2.00 | [X]Diabetes mellitus |
| Cyu2000 | [X]Other specified diabetes mellitus |
| Cyu2300 | [X]Unspecified diabetes mellitus with renal complications |
| F345000 | Diabetic mononeuritis multiplex |
| F35z000 | Diabetic mononeuritis NOS |
| F372.00 | Polyneuropathy in diabetes |
| F372.11 | Diabetic polyneuropathy |
| F372.12 | Diabetic neuropathy |
| F372100 | Chronic painful diabetic neuropathy |
| F372200 | Asymptomatic diabetic neuropathy |
| F381300 | Myasthenic syndrome due to diabetic amyotrophy |
| F381311 | Diabetic amyotrophy |
| F3y0.00 | Diabetic mononeuropathy |
| F420.00 | Diabetic retinopathy |
| F420000 | Background diabetic retinopathy |
| F420100 | Proliferative diabetic retinopathy |
| F420200 | Preproliferative diabetic retinopathy |
| F420300 | Advanced diabetic maculopathy |
| F420400 | Diabetic maculopathy |
| F420500 | Advanced diabetic retinal disease |
| F420600 | Non proliferative diabetic retinopathy |
| F420700 | High risk proliferative diabetic retinopathy |
| F420800 | High risk non proliferative diabetic retinopathy |
| F420z00 | Diabetic retinopathy NOS |
| F440700 | Diabetic iritis |
| F464000 | Diabetic cataract |
| G73y000 | Diabetic peripheral angiopathy |
| K01x100 | Nephrotic syndrome in diabetes mellitus |
| K081.00 | Nephrogenic diabetes insipidus |
| Kyu0300 | [X]Glomerular disorders in diabetes mellitus |
| L180500 | Pre-existing diabetes mellitus, insulin-dependent |
| L180600 | Pre-existing diabetes mellitus, non-insulin-dependent |
| L180X00 | Pre-existing diabetes mellitus, unspecified |
| Lyu2900 | [X]Pre-existing diabetes mellitus, unspecified |

Diabetes (Vaccine High Risk)

| **Read Code** | **Description** |
| --- | --- |
| **Those we believe should be included** | |
| C10..00 | Diabetes mellitus |
| C100.00 | Diabetes mellitus with no mention of complication |
| C100000 | Diabetes mellitus, juvenile type, no mention of complication |
| C100011 | Insulin dependent diabetes mellitus |
| C100100 | Diabetes mellitus, adult onset, no mention of complication |
| C100111 | Maturity onset diabetes |
| C100112 | Non-insulin dependent diabetes mellitus |
| C100z00 | Diabetes mellitus NOS with no mention of complication |
| C101.00 | Diabetes mellitus with ketoacidosis |
| C101000 | Diabetes mellitus, juvenile type, with ketoacidosis |
| C101100 | Diabetes mellitus, adult onset, with ketoacidosis |
| C101y00 | Other specified diabetes mellitus with ketoacidosis |
| C101z00 | Diabetes mellitus NOS with ketoacidosis |
| C102.00 | Diabetes mellitus with hyperosmolar coma |
| C102000 | Diabetes mellitus, juvenile type, with hyperosmolar coma |
| C102100 | Diabetes mellitus, adult onset, with hyperosmolar coma |
| C102z00 | Diabetes mellitus NOS with hyperosmolar coma |
| C103.00 | Diabetes mellitus with ketoacidotic coma |
| C103000 | Diabetes mellitus, juvenile type, with ketoacidotic coma |
| C103100 | Diabetes mellitus, adult onset, with ketoacidotic coma |
| C103y00 | Other specified diabetes mellitus with coma |
| C103z00 | Diabetes mellitus NOS with ketoacidotic coma |
| C104.00 | Diabetes mellitus with renal manifestation |
| C104.11 | Diabetic nephropathy |
| C104000 | Diabetes mellitus, juvenile type, with renal manifestation |
| C104100 | Diabetes mellitus, adult onset, with renal manifestation |
| C104y00 | Other specified diabetes mellitus with renal complications |
| C104z00 | Diabetes mellitis with nephropathy NOS |
| C105.00 | Diabetes mellitus with ophthalmic manifestation |
| C105000 | Diabetes mellitus, juvenile type, + ophthalmic manifestation |
| C105100 | Diabetes mellitus, adult onset, + ophthalmic manifestation |
| C105y00 | Other specified diabetes mellitus with ophthalmic complicatn |
| C105z00 | Diabetes mellitus NOS with ophthalmic manifestation |
| C106.00 | Diabetes mellitus with neurological manifestation |
| C106.11 | Diabetic amyotrophy |
| C106.12 | Diabetes mellitus with neuropathy |
| C106.13 | Diabetes mellitus with polyneuropathy |
| C106000 | Diabetes mellitus, juvenile, + neurological manifestation |
| C106100 | Diabetes mellitus, adult onset, + neurological manifestation |
| C106y00 | Other specified diabetes mellitus with neurological comps |
| C106z00 | Diabetes mellitus NOS with neurological manifestation |
| C107.00 | Diabetes mellitus with peripheral circulatory disorder |
| C107.11 | Diabetes mellitus with gangrene |
| C107.12 | Diabetes with gangrene |
| C107000 | Diabetes mellitus, juvenile +peripheral circulatory disorder |
| C107100 | Diabetes mellitus, adult, + peripheral circulatory disorder |
| C107200 | Diabetes mellitus, adult with gangrene |
| C107300 | IDDM with peripheral circulatory disorder |
| C107400 | NIDDM with peripheral circulatory disorder |
| C107y00 | Other specified diabetes mellitus with periph circ comps |
| C107z00 | Diabetes mellitus NOS with peripheral circulatory disorder |
| C108.00 | Insulin dependent diabetes mellitus |
| C108.11 | IDDM-Insulin dependent diabetes mellitus |
| C108.12 | Type 1 diabetes mellitus |
| C108.13 | Type I diabetes mellitus |
| C108000 | Insulin-dependent diabetes mellitus with renal complications |
| C108011 | Type I diabetes mellitus with renal complications |
| C108012 | Type 1 diabetes mellitus with renal complications |
| C108100 | Insulin-dependent diabetes mellitus with ophthalmic comps |
| C108111 | Type I diabetes mellitus with ophthalmic complications |
| C108112 | Type 1 diabetes mellitus with ophthalmic complications |
| C108200 | Insulin-dependent diabetes mellitus with neurological comps |
| C108211 | Type I diabetes mellitus with neurological complications |
| C108212 | Type 1 diabetes mellitus with neurological complications |
| C108300 | Insulin dependent diabetes mellitus with multiple complicatn |
| C108311 | Type I diabetes mellitus with multiple complications |
| C108312 | Type 1 diabetes mellitus with multiple complications |
| C108400 | Unstable insulin dependent diabetes mellitus |
| C108411 | Unstable type I diabetes mellitus |
| C108412 | Unstable type 1 diabetes mellitus |
| C108500 | Insulin dependent diabetes mellitus with ulcer |
| C108511 | Type I diabetes mellitus with ulcer |
| C108512 | Type 1 diabetes mellitus with ulcer |
| C108600 | Insulin dependent diabetes mellitus with gangrene |
| C108611 | Type I diabetes mellitus with gangrene |
| C108612 | Type 1 diabetes mellitus with gangrene |
| C108700 | Insulin dependent diabetes mellitus with retinopathy |
| C108711 | Type I diabetes mellitus with retinopathy |
| C108712 | Type 1 diabetes mellitus with retinopathy |
| C108800 | Insulin dependent diabetes mellitus - poor control |
| C108811 | Type I diabetes mellitus - poor control |
| C108812 | Type 1 diabetes mellitus - poor control |
| C108900 | Insulin dependent diabetes maturity onset |
| C108911 | Type I diabetes mellitus maturity onset |
| C108912 | Type 1 diabetes mellitus maturity onset |
| C108A00 | Insulin-dependent diabetes without complication |
| C108A11 | Type I diabetes mellitus without complication |
| C108A12 | Type 1 diabetes mellitus without complication |
| C108B00 | Insulin dependent diabetes mellitus with mononeuropathy |
| C108B11 | Type I diabetes mellitus with mononeuropathy |
| C108B12 | Type 1 diabetes mellitus with mononeuropathy |
| C108C00 | Insulin dependent diabetes mellitus with polyneuropathy |
| C108C11 | Type I diabetes mellitus with polyneuropathy |
| C108C12 | Type 1 diabetes mellitus with polyneuropathy |
| C108D00 | Insulin dependent diabetes mellitus with nephropathy |
| C108D11 | Type I diabetes mellitus with nephropathy |
| C108D12 | Type 1 diabetes mellitus with nephropathy |
| C108E00 | Insulin dependent diabetes mellitus with hypoglycaemic coma |
| C108E11 | Type I diabetes mellitus with hypoglycaemic coma |
| C108E12 | Type 1 diabetes mellitus with hypoglycaemic coma |
| C108F00 | Insulin dependent diabetes mellitus with diabetic cataract |
| C108F11 | Type I diabetes mellitus with diabetic cataract |
| C108F12 | Type 1 diabetes mellitus with diabetic cataract |
| C108G00 | Insulin dependent diab mell with peripheral angiopathy |
| C108G11 | Type I diabetes mellitus with peripheral angiopathy |
| C108G12 | Type 1 diabetes mellitus with peripheral angiopathy |
| C108H00 | Insulin dependent diabetes mellitus with arthropathy |
| C108H11 | Type I diabetes mellitus with arthropathy |
| C108H12 | Type 1 diabetes mellitus with arthropathy |
| C108J00 | Insulin dependent diab mell with neuropathic arthropathy |
| C108J11 | Type I diabetes mellitus with neuropathic arthropathy |
| C108J12 | Type 1 diabetes mellitus with neuropathic arthropathy |
| C108y00 | Other specified diabetes mellitus with multiple comps |
| C108z00 | Unspecified diabetes mellitus with multiple complications |
| C109.00 | Non-insulin dependent diabetes mellitus |
| C109.11 | NIDDM - Non-insulin dependent diabetes mellitus |
| C109.12 | Type 2 diabetes mellitus |
| C109.13 | Type II diabetes mellitus |
| C109000 | Non-insulin-dependent diabetes mellitus with renal comps |
| C109011 | Type II diabetes mellitus with renal complications |
| C109012 | Type 2 diabetes mellitus with renal complications |
| C109100 | Non-insulin-dependent diabetes mellitus with ophthalm comps |
| C109111 | Type II diabetes mellitus with ophthalmic complications |
| C109112 | Type 2 diabetes mellitus with ophthalmic complications |
| C109200 | Non-insulin-dependent diabetes mellitus with neuro comps |
| C109211 | Type II diabetes mellitus with neurological complications |
| C109212 | Type 2 diabetes mellitus with neurological complications |
| C109300 | Non-insulin-dependent diabetes mellitus with multiple comps |
| C109311 | Type II diabetes mellitus with multiple complications |
| C109312 | Type 2 diabetes mellitus with multiple complications |
| C109400 | Non-insulin dependent diabetes mellitus with ulcer |
| C109411 | Type II diabetes mellitus with ulcer |
| C109412 | Type 2 diabetes mellitus with ulcer |
| C109500 | Non-insulin dependent diabetes mellitus with gangrene |
| C109511 | Type II diabetes mellitus with gangrene |
| C109512 | Type 2 diabetes mellitus with gangrene |
| C109600 | Non-insulin-dependent diabetes mellitus with retinopathy |
| C109611 | Type II diabetes mellitus with retinopathy |
| C109612 | Type 2 diabetes mellitus with retinopathy |
| C109700 | Non-insulin dependent diabetes mellitus - poor control |
| C109711 | Type II diabetes mellitus - poor control |
| C109712 | Type 2 diabetes mellitus - poor control |
| C109800 | Reaven's syndrome |
| C109900 | Non-insulin-dependent diabetes mellitus without complication |
| C109911 | Type II diabetes mellitus without complication |
| C109912 | Type 2 diabetes mellitus without complication |
| C109A00 | Non-insulin dependent diabetes mellitus with mononeuropathy |
| C109A11 | Type II diabetes mellitus with mononeuropathy |
| C109A12 | Type 2 diabetes mellitus with mononeuropathy |
| C109B00 | Non-insulin dependent diabetes mellitus with polyneuropathy |
| C109B11 | Type II diabetes mellitus with polyneuropathy |
| C109B12 | Type 2 diabetes mellitus with polyneuropathy |
| C109C00 | Non-insulin dependent diabetes mellitus with nephropathy |
| C109C11 | Type II diabetes mellitus with nephropathy |
| C109C12 | Type 2 diabetes mellitus with nephropathy |
| C109D00 | Non-insulin dependent diabetes mellitus with hypoglyca coma |
| C109D11 | Type II diabetes mellitus with hypoglycaemic coma |
| C109D12 | Type 2 diabetes mellitus with hypoglycaemic coma |
| C109E00 | Non-insulin depend diabetes mellitus with diabetic cataract |
| C109E11 | Type II diabetes mellitus with diabetic cataract |
| C109E12 | Type 2 diabetes mellitus with diabetic cataract |
| C109F00 | Non-insulin-dependent d m with peripheral angiopath |
| C109F11 | Type II diabetes mellitus with peripheral angiopathy |
| C109F12 | Type 2 diabetes mellitus with peripheral angiopathy |
| C109G00 | Non-insulin dependent diabetes mellitus with arthropathy |
| C109G11 | Type II diabetes mellitus with arthropathy |
| C109G12 | Type 2 diabetes mellitus with arthropathy |
| C109H00 | Non-insulin dependent d m with neuropathic arthropathy |
| C109H11 | Type II diabetes mellitus with neuropathic arthropathy |
| C109H12 | Type 2 diabetes mellitus with neuropathic arthropathy |
| C109J00 | Insulin treated Type 2 diabetes mellitus |
| C109J11 | Insulin treated non-insulin dependent diabetes mellitus |
| C109J12 | Insulin treated Type II diabetes mellitus |
| C109K00 | Hyperosmolar non-ketotic state in type 2 diabetes mellitus |
| C10A.00 | Malnutrition-related diabetes mellitus |
| C10A000 | Malnutrition-related diabetes mellitus with coma |
| C10A100 | Malnutrition-related diabetes mellitus with ketoacidosis |
| C10A200 | Malnutrition-related diabetes mellitus with renal complicatn |
| C10A300 | Malnutrit-related diabetes mellitus wth ophthalmic complicat |
| C10A400 | Malnutrition-related diabetes mellitus wth neuro complicatns |
| C10A500 | Malnutritn-relat diabetes melitus wth periph circul complctn |
| C10A600 | Malnutrition-related diabetes mellitus with multiple comps |
| C10A700 | Malnutrition-related diabetes mellitus without complications |
| C10AW00 | Malnutrit-related diabetes mellitus with unspec complics |
| C10AX00 | Malnutrit-relat diabetes mellitus with other spec comps |
| C10B.00 | Diabetes mellitus induced by steroids |
| C10B000 | Steroid induced diabetes mellitus without complication |
| C10C.00 | Diabetes mellitus autosomal dominant |
| C10C.11 | Maturity onset diabetes in youth |
| C10C.12 | Maturity onset diabetes in youth type 1 |
| C10D.00 | Diabetes mellitus autosomal dominant type 2 |
| C10D.11 | Maturity onset diabetes in youth type 2 |
| C10E.00 | Type 1 diabetes mellitus |
| C10E.11 | Type I diabetes mellitus |
| C10E.12 | Insulin dependent diabetes mellitus |
| C10E000 | Type 1 diabetes mellitus with renal complications |
| C10E011 | Type I diabetes mellitus with renal complications |
| C10E012 | Insulin-dependent diabetes mellitus with renal complications |
| C10E100 | Type 1 diabetes mellitus with ophthalmic complications |
| C10E111 | Type I diabetes mellitus with ophthalmic complications |
| C10E112 | Insulin-dependent diabetes mellitus with ophthalmic comps |
| C10E200 | Type 1 diabetes mellitus with neurological complications |
| C10E211 | Type I diabetes mellitus with neurological complications |
| C10E212 | Insulin-dependent diabetes mellitus with neurological comps |
| C10E300 | Type 1 diabetes mellitus with multiple complications |
| C10E311 | Type I diabetes mellitus with multiple complications |
| C10E312 | Insulin dependent diabetes mellitus with multiple complicat |
| C10E400 | Unstable type 1 diabetes mellitus |
| C10E411 | Unstable type I diabetes mellitus |
| C10E412 | Unstable insulin dependent diabetes mellitus |
| C10E500 | Type 1 diabetes mellitus with ulcer |
| C10E511 | Type I diabetes mellitus with ulcer |
| C10E512 | Insulin dependent diabetes mellitus with ulcer |
| C10E600 | Type 1 diabetes mellitus with gangrene |
| C10E611 | Type I diabetes mellitus with gangrene |
| C10E612 | Insulin dependent diabetes mellitus with gangrene |
| C10E700 | Type 1 diabetes mellitus with retinopathy |
| C10E711 | Type I diabetes mellitus with retinopathy |
| C10E712 | Insulin dependent diabetes mellitus with retinopathy |
| C10E800 | Type 1 diabetes mellitus - poor control |
| C10E811 | Type I diabetes mellitus - poor control |
| C10E812 | Insulin dependent diabetes mellitus - poor control |
| C10E900 | Type 1 diabetes mellitus maturity onset |
| C10E911 | Type I diabetes mellitus maturity onset |
| C10E912 | Insulin dependent diabetes maturity onset |
| C10EA00 | Type 1 diabetes mellitus without complication |
| C10EA11 | Type I diabetes mellitus without complication |
| C10EA12 | Insulin-dependent diabetes without complication |
| C10EB00 | Type 1 diabetes mellitus with mononeuropathy |
| C10EB11 | Type I diabetes mellitus with mononeuropathy |
| C10EB12 | Insulin dependent diabetes mellitus with mononeuropathy |
| C10EC00 | Type 1 diabetes mellitus with polyneuropathy |
| C10EC11 | Type I diabetes mellitus with polyneuropathy |
| C10EC12 | Insulin dependent diabetes mellitus with polyneuropathy |
| C10ED00 | Type 1 diabetes mellitus with nephropathy |
| C10ED11 | Type I diabetes mellitus with nephropathy |
| C10ED12 | Insulin dependent diabetes mellitus with nephropathy |
| C10EE00 | Type 1 diabetes mellitus with hypoglycaemic coma |
| C10EE11 | Type I diabetes mellitus with hypoglycaemic coma |
| C10EE12 | Insulin dependent diabetes mellitus with hypoglycaemic coma |
| C10EF00 | Type 1 diabetes mellitus with diabetic cataract |
| C10EF11 | Type I diabetes mellitus with diabetic cataract |
| C10EF12 | Insulin dependent diabetes mellitus with diabetic cataract |
| C10EG00 | Type 1 diabetes mellitus with peripheral angiopathy |
| C10EG11 | Type I diabetes mellitus with peripheral angiopathy |
| C10EG12 | Insulin dependent diab mell with peripheral angiopathy |
| C10EH00 | Type 1 diabetes mellitus with arthropathy |
| C10EH11 | Type I diabetes mellitus with arthropathy |
| C10EH12 | Insulin dependent diabetes mellitus with arthropathy |
| C10EJ00 | Type 1 diabetes mellitus with neuropathic arthropathy |
| C10EJ11 | Type I diabetes mellitus with neuropathic arthropathy |
| C10EJ12 | Insulin dependent diab mell with neuropathic arthropathy |
| C10EK00 | Type 1 diabetes mellitus with persistent proteinuria |
| C10EK11 | Type I diabetes mellitus with persistent proteinuria |
| C10EL00 | Type 1 diabetes mellitus with persistent microalbuminuria |
| C10EL11 | Type I diabetes mellitus with persistent microalbuminuria |
| C10EM00 | Type 1 diabetes mellitus with ketoacidosis |
| C10EM11 | Type I diabetes mellitus with ketoacidosis |
| C10EN00 | Type 1 diabetes mellitus with ketoacidotic coma |
| C10EN11 | Type I diabetes mellitus with ketoacidotic coma |
| C10EP00 | Type 1 diabetes mellitus with exudative maculopathy |
| C10EP11 | Type I diabetes mellitus with exudative maculopathy |
| C10EQ00 | Type 1 diabetes mellitus with gastroparesis |
| C10F.00 | Type 2 diabetes mellitus |
| C10F.11 | Type II diabetes mellitus |
| C10F000 | Type 2 diabetes mellitus with renal complications |
| C10F011 | Type II diabetes mellitus with renal complications |
| C10F100 | Type 2 diabetes mellitus with ophthalmic complications |
| C10F111 | Type II diabetes mellitus with ophthalmic complications |
| C10F200 | Type 2 diabetes mellitus with neurological complications |
| C10F211 | Type II diabetes mellitus with neurological complications |
| C10F300 | Type 2 diabetes mellitus with multiple complications |
| C10F311 | Type II diabetes mellitus with multiple complications |
| C10F400 | Type 2 diabetes mellitus with ulcer |
| C10F411 | Type II diabetes mellitus with ulcer |
| C10F500 | Type 2 diabetes mellitus with gangrene |
| C10F511 | Type II diabetes mellitus with gangrene |
| C10F600 | Type 2 diabetes mellitus with retinopathy |
| C10F611 | Type II diabetes mellitus with retinopathy |
| C10F700 | Type 2 diabetes mellitus - poor control |
| C10F711 | Type II diabetes mellitus - poor control |
| C10F800 | Reaven's syndrome |
| C10F811 | Metabolic syndrome X |
| C10F900 | Type 2 diabetes mellitus without complication |
| C10F911 | Type II diabetes mellitus without complication |
| C10FA00 | Type 2 diabetes mellitus with mononeuropathy |
| C10FA11 | Type II diabetes mellitus with mononeuropathy |
| C10FB00 | Type 2 diabetes mellitus with polyneuropathy |
| C10FB11 | Type II diabetes mellitus with polyneuropathy |
| C10FC00 | Type 2 diabetes mellitus with nephropathy |
| C10FC11 | Type II diabetes mellitus with nephropathy |
| C10FD00 | Type 2 diabetes mellitus with hypoglycaemic coma |
| C10FD11 | Type II diabetes mellitus with hypoglycaemic coma |
| C10FE00 | Type 2 diabetes mellitus with diabetic cataract |
| C10FE11 | Type II diabetes mellitus with diabetic cataract |
| C10FF00 | Type 2 diabetes mellitus with peripheral angiopathy |
| C10FF11 | Type II diabetes mellitus with peripheral angiopathy |
| C10FG00 | Type 2 diabetes mellitus with arthropathy |
| C10FG11 | Type II diabetes mellitus with arthropathy |
| C10FH00 | Type 2 diabetes mellitus with neuropathic arthropathy |
| C10FH11 | Type II diabetes mellitus with neuropathic arthropathy |
| C10FJ00 | Insulin treated Type 2 diabetes mellitus |
| C10FJ11 | Insulin treated Type II diabetes mellitus |
| C10FK00 | Hyperosmolar non-ketotic state in type 2 diabetes mellitus |
| C10FL00 | Type 2 diabetes mellitus with persistent proteinuria |
| C10FL11 | Type II diabetes mellitus with persistent proteinuria |
| C10FM00 | Type 2 diabetes mellitus with persistent microalbuminuria |
| C10FM11 | Type II diabetes mellitus with persistent microalbuminuria |
| C10FN00 | Type 2 diabetes mellitus with ketoacidosis |
| C10FN11 | Type II diabetes mellitus with ketoacidosis |
| C10FP00 | Type 2 diabetes mellitus with ketoacidotic coma |
| C10FP11 | Type II diabetes mellitus with ketoacidotic coma |
| C10FQ00 | Type 2 diabetes mellitus with exudative maculopathy |
| C10FQ11 | Type II diabetes mellitus with exudative maculopathy |
| C10FR00 | Type 2 diabetes mellitus with gastroparesis |
| C10G.00 | Secondary pancreatic diabetes mellitus |
| C10G000 | Secondary pancreatic diabetes mellitus without complication |
| C10H.00 | Diabetes mellitus induced by non-steroid drugs |
| C10H000 | DM induced by non-steroid drugs without complication |
| C10J.00 | Insulin autoimmune syndrome |
| C10J000 | Insulin autoimmune syndrome without complication |
| C10K.00 | Type A insulin resistance |
| C10K000 | Type A insulin resistance without complication |
| C10L.00 | Fibrocalculous pancreatopathy |
| C10L000 | Fibrocalculous pancreatopathy without complication |
| C10M.00 | Lipoatrophic diabetes mellitus |
| C10M000 | Lipoatrophic diabetes mellitus without complication |
| C10N.00 | Secondary diabetes mellitus |
| C10N000 | Secondary diabetes mellitus without complication |
| C10y.00 | Diabetes mellitus with other specified manifestation |
| C10y000 | Diabetes mellitus, juvenile, + other specified manifestation |
| C10y100 | Diabetes mellitus, adult, + other specified manifestation |
| C10yy00 | Other specified diabetes mellitus with other spec comps |
| C10yz00 | Diabetes mellitus NOS with other specified manifestation |
| C10z.00 | Diabetes mellitus with unspecified complication |
| C10z000 | Diabetes mellitus, juvenile type, + unspecified complication |
| C10z100 | Diabetes mellitus, adult onset, + unspecified complication |
| C10zy00 | Other specified diabetes mellitus with unspecified comps |
| C10zz00 | Diabetes mellitus NOS with unspecified complication |

## High Risk Groups

High risk groups

| **Read Code** | **Description** |
| --- | --- |
| **Those we believe should be included** | |
| 65E..00 | Influenza vaccination |
| 68NE.00 | No consent - influenza imm. |
| 68NN.00 | Influenza imm.advised in surg. |
| 68NN.11 | Influenza immunization advised |
| 68NO.00 | Influenza imm.advised at home |
| 68NV.00 | Influenza vacc consent given |
| 9k7..00 | Influenza immunisation - enhanced services administration |
| 9N4q.00 | Did not attend flu vaccination appointment |
| 9OX..00 | Influenza vacc. administration. |
| 9OX..11 | Flu vaccination administration |
| 9OX1.00 | Has 'flu vaccination at home |
| 9OX2.00 | Has'flu vaccination at surgery |
| 9OX3.00 | Has 'flu vaccination at hosp. |
| 9OX4.00 | Needs influenza immunisation |
| 9OX5.00 | Influenza vaccination declined |
| 9OX6.00 | Influenza vaccination invitation letter sent |
| 9OX7.00 | Influenza vaccination telephone invite |
| 9OX8.00 | Has influenza vaccination at work |
| 9OX9.00 | Influenza vaccination invitation first letter sent |
| 9OXA.00 | Influenza vaccination invitation second letter sent |
| 9OXB.00 | Influenza vaccination invitation third letter sent |
| 9OXZ.00 | Influenza vacc.administrat.NOS |
| ZV04800 | [V]Influenza vaccination |
| ZV04811 | [V]Flu - influenza vaccination |

## Liver Disease

Liver disease (Vaccine High Risk)

| **Read Code** | **Description** |
| --- | --- |
| **Those we believe should be included** | |
| J60..00 | Acute and subacute liver necrosis |
| J600.00 | Acute necrosis of liver |
| J600000 | Acute hepatic failure |
| J600011 | Acute liver failure |
| J600100 | Acute hepatitis - noninfective |
| J600200 | Acute yellow atrophy |
| J600z00 | Acute necrosis of liver NOS |
| J601.00 | Subacute necrosis of liver |
| J601000 | Subacute hepatic failure |
| J601100 | Subacute hepatitis - noninfective |
| J601200 | Subacute yellow atrophy |
| J601z00 | Subacute necrosis of liver NOS |
| J60z.00 | Acute and subacute liver necrosis NOS |
| J61..00 | Cirrhosis and chronic liver disease |
| J610.00 | Alcoholic fatty liver |
| J611.00 | Acute alcoholic hepatitis |
| J612.00 | Alcoholic cirrhosis of liver |
| J612.11 | Florid cirrhosis |
| J612.12 | Laennec's cirrhosis |
| J612000 | Alcoholic fibrosis and sclerosis of liver |
| J613.00 | Alcoholic liver damage unspecified |
| J613000 | Alcoholic hepatic failure |
| J614.00 | Chronic hepatitis |
| J614000 | Chronic persistent hepatitis |
| J614100 | Chronic active hepatitis |
| J614111 | Autoimmune chronic active hepatitis |
| J614200 | Chronic aggressive hepatitis |
| J614300 | Recurrent hepatitis |
| J614400 | Chronic lobular hepatitis |
| J614y00 | Chronic hepatitis unspecified |
| J614z00 | Chronic hepatitis NOS |
| J615.00 | Cirrhosis - non alcoholic |
| J615.11 | Portal cirrhosis |
| J615000 | Unilobular portal cirrhosis |
| J615100 | Multilobular portal cirrhosis |
| J615111 | Postnecrotic cirrhosis of liver |
| J615200 | Mixed portal cirrhosis |
| J615300 | Diffuse nodular cirrhosis |
| J615400 | Fatty portal cirrhosis |
| J615500 | Hypertrophic portal cirrhosis |
| J615600 | Capsular portal cirrhosis |
| J615700 | Cardiac portal cirrhosis |
| J615711 | Congestive cirrhosis |
| J615800 | Juvenile portal cirrhosis |
| J615811 | Childhood function cirrhosis |
| J615812 | Indian childhood cirrhosis |
| J615900 | Pigmentary portal cirrhosis |
| J615A00 | Pipe-stem portal cirrhosis |
| J615B00 | Toxic portal cirrhosis |
| J615C00 | Xanthomatous portal cirrhosis |
| J615D00 | Bacterial portal cirrhosis |
| J615E00 | Cardituberculous cirrhosis |
| J615F00 | Syphilitic portal cirrhosis |
| J615G00 | Zooparasitic portal cirrhosis |
| J615H00 | Infectious cirrhosis NOS |
| J615y00 | Portal cirrhosis unspecified |
| J615z00 | Non-alcoholic cirrhosis NOS |
| J615z11 | Macronodular cirrhosis of liver |
| J615z12 | Cryptogenic cirrhosis of liver |
| J615z13 | Cirrhosis of liver NOS |
| J615z14 | Laennec's cirrhosis, non-alcoholic |
| J615z15 | Hepatic fibrosis |
| J616.00 | Biliary cirrhosis |
| J616000 | Primary biliary cirrhosis |
| J616100 | Secondary biliary cirrhosis |
| J616200 | Biliary cirrhosis of children |
| J616z00 | Biliary cirrhosis NOS |
| J617.00 | Alcoholic hepatitis |
| J617000 | Chronic alcoholic hepatitis |
| J61y.00 | Other non-alcoholic chronic liver disease |
| J61y000 | Chronic yellow liver atrophy |
| J61y100 | Non-alcoholic fatty liver |
| J61y200 | Hepatosplenomegaly |
| J61y300 | Portal fibrosis without cirrhosis |
| J61y400 | Hepatic fibrosis |
| J61y500 | Hepatic sclerosis |
| J61y600 | Hepatic fibrosis with hepatic sclerosis |
| J61y700 | Steatosis of liver |
| J61yz00 | Other non-alcoholic chronic liver disease NOS |
| J61z.00 | Chronic liver disease NOS |
| J62..00 | Liver abscess and sequelae of chronic liver disease |
| J620.00 | Liver abscess - excluding amoebic liver abscess |
| J620000 | Liver abscess due to portal pyaemia |
| J620100 | Liver abscess due to cholangitis |
| J620200 | Liver abscess via hepatic artery |
| J620300 | Liver abscess via umbilicus |
| J620400 | Liver abscess due to direct extension |
| J620z00 | Liver abscess NOS |
| J621.00 | Portal pyaemia |
| J621.11 | Phlebitis of portal vein |
| J622.00 | Hepatic coma |
| J622.11 | Encephalopathy - hepatic |
| J623.00 | Portal hypertension |
| J624.00 | Hepatorenal syndrome |
| J625.00 | [X] Hepatic failure |
| J625.11 | [X] Liver failure |
| J62y.00 | Other sequelae of chronic liver disease |
| J62y.11 | Hepatic failure NOS |
| J62y.12 | Liver failure NOS |
| J62y.13 | Hepatic failure |
| J62z.00 | Liver abscess and chronic liver disease causing sequelae NOS |
| PB61.00 | Biliary atresia |
| PB61.11 | Bile duct atresia |
| PB61000 | Congenital absence of bile duct |
| PB61011 | Agenesis of bile duct |
| PB61100 | Congenital hypoplasia of bile duct |
| PB61200 | Congenital obstruction of bile duct |
| PB61300 | Congenital stricture of bile duct |
| PB61311 | Congenital stricture of common bile duct |
| PB61400 | Atresia of bile duct |
| PB61411 | Intrahepatic atresia of bile duct |
| PB61412 | Extrahepatic atresia of bile duct |
| PB61500 | Congenital absence of hepatic ducts |
| PB61511 | Agenesis of hepatic ducts |
| PB61600 | Atresia of hepatic ducts |
| PB61z00 | Biliary atresia NOS |
| PB63.00 | Congenital absence of liver and gallbladder |
| PB63000 | Congenital absence of gallbladder |
| PB63011 | Agenesis of gallbladder |
| PB63100 | Congenital absence of liver lobe |
| PB63111 | Congenital agenesis of liver lobe |
| PB63200 | Congenital small left lobe of liver |
| PB63300 | Riedel's lobe liver |
| PB63400 | Congenital absence of liver,total |
| PB63411 | Congenital agenesis liver,total |
| PB63500 | Alagille syndrome |
| PB63z00 | Absence of liver or gallbladder NOS |
| PB6y100 | Congenital hepatomegaly |

## Pregnancy

Pregnancy

| **Read Code** | **Description** |
| --- | --- |
| **Those we believe should be included** | |
| 13H7.00 | Unwanted pregnancy |
| 13H8.00 | Illegitimate pregnancy |
| 13Hd.00 | Teenage pregnancy |
| 4453.00 | Serum pregnancy test positive |
| 4654.00 | Urine pregnancy test positive |
| 584D.00 | Antenatal ultrasound confirms intra-uterine pregnancy |
| 584E.00 | Antenatal ultrasound confirms ectopic pregnancy |
| 615C.00 | IUD failure - pregnant |
| 615C.11 | Pregnant, IUD failure |
| 6166.00 | Pregnant, diaphragm failure |
| 6174.00 | Pregnant, sheath failure |
| 62...00 | Patient pregnant |
| 62...13 | Pregnancy care |
| 621..00 | Patient currently pregnant |
| 621..11 | Pregnancy confirmed |
| 6211.00 | Pregnant - urine test confirms |
| 6212.00 | Pregnant - blood test confirms |
| 6213.00 | Pregnant - V.E. confirms |
| 6214.00 | Pregnant - on history |
| 6215.00 | Pregnant - on abdom. palpation |
| 6216.00 | Pregnant - planned |
| 6217.00 | Pregnant - unplanned - wanted |
| 6218.00 | Pregnant -unplanned-not wanted |
| 6219.00 | Patient ? pregnant |
| 621A.00 | Pregnancy unplanned ? wanted |
| 621B.00 | Pregnant - ? planned |
| 621C.00 | Unplanned pregnancy |
| 621Z.00 | Patient pregnant NOS |
| 6222.00 | Antenatal care: 2nd pregnancy |
| 6223.00 | Antenatal care: 3rd pregnancy |
| 624..00 | A/N care: precious pregnancy |
| 62a..00 | Pregnancy review |
| 62a..11 | Review of pregnancy |
| 62O..12 | Static weight gain pregnancy |
| 62O7.00 | Pregnancy prolonged - 41 weeks |
| 62O8.00 | Pregnancy prolonged - 42 weeks |
| 66AX.00 | Diabetes: shared care in pregnancy - diabetol and obstet |
| 7E06600 | Hysterotomy and termination of pregnancy |
| 7E07011 | Dilation cervix uteri & curettage for termination pregnancy |
| 7E07111 | Curettage of term pregnancy NEC |
| 7E07113 | Curettage of uterus for termination of pregnancy NEC |
| 7E07114 | Curettage of uterus for termination of pregnancy NEC |
| 7E08400 | Suction termination of pregnancy |
| 7E08411 | Vacuum termination of pregnancy |
| 7E08500 | Dilation of cervix and extraction termination of pregnancy |
| 7E08600 | Termination of pregnancy NEC |
| 7E13100 | Excision of ectopic ovarian pregnancy |
| 7E13300 | Excision of ruptured ectopic tubal pregnancy |
| 7E19011 | Removal of ectopic pregnancy from fallopian tube |
| 7E19012 | Fimbrial extraction of tubal pregnancy |
| 7F...12 | Pregnancy operations |
| 7F06012 | Shirodkar suture in pregnancy |
| 7F2B100 | Ultrasound monitoring of early pregnancy |
| 8B68.00 | Pregnancy prophylactic therapy |
| 8B7..11 | Pregnancy vitamin/iron prophyl |
| 8B74.00 | Iron supplement in pregnancy |
| 8B75.00 | Vitamin supplement - pregnancy |
| 8HHV.00 | Referral for termination of pregnancy |
| 8M6..00 | Requests pregnancy termination |
| 9Ea..00 | Reason for termination of pregnancy |
| 9Ea0.00 | Risk life pregnant woman greater than if pregnancy terminatd |
| L....00 | Complications of pregnancy, childbirth and the puerperium |
| L0...00 | Pregnancy with abortive outcome |
| L010.11 | Anembryonic pregnancy |
| L03..00 | Ectopic pregnancy |
| L030.00 | Abdominal pregnancy |
| L030000 | Delivery of viable fetus in abdominal pregnancy |
| L031.00 | Tubal pregnancy |
| L031000 | Fallopian tube pregnancy |
| L031z00 | Tubal pregnancy NOS |
| L032.00 | Ovarian pregnancy |
| L03y.00 | Other ectopic pregnancy |
| L03y000 | Cervical pregnancy |
| L03y100 | Cornual pregnancy |
| L03y200 | Membranous pregnancy |
| L03y300 | Combined or heterotopic pregnancy |
| L03y400 | Mural pregnancy |
| L03y500 | Intraligamentous pregnancy |
| L03y600 | Mesenteric pregnancy |
| L03y700 | Angular pregnancy |
| L03y800 | Mesometric pregnancy |
| L03yz00 | Other ectopic pregnancy NOS |
| L03z.00 | Ectopic pregnancy NOS |
| L05..12 | Termination of pregnancy |
| L0y..00 | Other specified pregnancy with abortive outcome |
| L0z..00 | Pregnancy with abortive outcome NOS |
| L15..00 | Prolonged or post-term pregnancy |
| L15..11 | Post-term pregnancy |
| L150.00 | Post-term pregnancy |
| L150000 | Post-term pregnancy unspecified |
| L150100 | Post-term pregnancy - delivered |
| L150200 | Post-term pregnancy - not delivered |
| L150z00 | Post-term pregnancy NOS |
| L15z.00 | Prolonged pregnancy NOS |
| L191.00 | Continuing pregnancy after abortion of one fetus or more |
| L2...00 | Risk factors in pregnancy |
| L21..00 | Multiple pregnancy |
| L210.00 | Twin pregnancy |
| L210000 | Twin pregnancy unspecified |
| L210100 | Twin pregnancy - delivered |
| L210200 | Twin pregnancy with antenatal problem |
| L210z00 | Twin pregnancy NOS |
| L211.00 | Triplet pregnancy |
| L211000 | Triplet pregnancy unspecified |
| L211100 | Triplet pregnancy - delivered |
| L211200 | Triplet pregnancy with antenatal problem |
| L211z00 | Triplet pregnancy NOS |
| L212.00 | Quadruplet pregnancy |
| L212000 | Quadruplet pregnancy unspecified |
| L212100 | Quadruplet pregnancy - delivered |
| L212200 | Quadruplet pregnancy with antenatal problem |
| L212z00 | Quadruplet pregnancy NOS |
| L21y.00 | Other multiple pregnancy |
| L21y000 | Other multiple pregnancy unspecified |
| L21y100 | Other multiple pregnancy - delivered |
| L21y200 | Other multiple pregnancy with antenatal problem |
| L21yz00 | Other multiple pregnancy NOS |
| L21z.00 | Multiple pregnancy NOS |
| L21z000 | Multiple pregnancy NOS, unspecified |
| L21z100 | Multiple pregnancy NOS - delivered |
| L21z200 | Multiple pregnancy NOS with antenatal problem |
| L21zz00 | Multiple pregnancy NOS |
| L228.00 | Multiple pregnancy with malpresentation |
| L228000 | Multiple pregnancy with malpresentation unspecified |
| L228100 | Multiple pregnancy with malpresentation - delivered |
| L228200 | Multiple pregnancy with malpresentation with antenatal prob |
| L228z00 | Multiple pregnancy with malpresentation NOS |
| L2z..00 | Risk factors in pregnancy NOS |
| L398200 | Caesarean section - pregnancy at term |
| Lyu0.00 | [X]Pregnancy with abortive outcome |
| Lyu0000 | [X]Other ectopic pregnancy |
| Lyu0A00 | [X]Other complications follow abortn+ectopic+molar pregnancy |
| Lyu1.00 | [X]Oedema,proteinuria+hypertens in pregnancy,childbrth,puerp |
| Lyu2.00 | [X]Other maternal disorders predominant related to pregnancy |
| Lyu2500 | [X]Other specified pregnancy-related conditions |
| Z2...00 | Pregnancy, childbirth and puerperium observations |
| Z21..00 | Care relating to reproduction and pregnancy |
| Z212.11 | Pregnancy care |
| Z22..00 | Pregnancy observations |
| Z225.00 | Normal pregnancy |
| Z226.00 | Pregnancy problem |
| Z227.00 | Confirmation of pregnancy |
| Z229.00 | Observation of position of pregnancy |
| Z229100 | Intrauterine pregnancy |
| Z22A.00 | Observation of pattern of pregnancy |
| Z22A100 | Low risk pregnancy |
| Z22A200 | High risk pregnancy |
| Z22A211 | HRP - High risk pregnancy |
| Z22A300 | Concealed pregnancy |
| Z22A400 | Early stage of pregnancy |
| Z22A500 | Biochemical pregnancy |
| Z22A600 | Teenage pregnancy |
| Z22A700 | Surrogate pregnancy |
| Z22A800 | Undiagnosed pregnancy |
| Z22A900 | Unwanted pregnancy |
| Z22AA00 | Wanted pregnancy |
| Z22AB00 | Unplanned pregnancy |
| Z22AB11 | Accidental pregnancy |
| Z22AC00 | Pregnancy with uncertain dates |
| Z22AD00 | Presentation of pregnancy |
| Z22AD11 | Reported conception - pregnancy |
| Z22B.00 | Observation of quantity of pregnancy |
| Z22B100 | Single pregnancy |
| Z22B500 | Quintuplet pregnancy |
| Z22B600 | Sextuplet pregnancy |
| Z22B700 | Septulet pregnancy |
| Z22B800 | Undiagnosed multiple pregnancy |
| Z22B900 | Continuing pregnancy after abortion of sibling fetus |
| Z22BA00 | Contin pregnancy after intrauterine death of sibling fetus |
| Z235.00 | Observation of shape of pregnant abdomen |
| Z235100 | Ovoid pregnant abdomen |
| Z235200 | Rounded pregnant abdomen |
| Z235211 | Globular pregnant abdomen |
| Z235300 | Transversely enlarged pregnant abdomen |
| Z235400 | Pendulous pregnant abdomen |
| Z236300 | Pregnant uterus displaced laterally |
| Z23D100 | Girth of pregnant abdomen |
| Z23D200 | Pregnant abdomen observation |
| Z254500 | Delivered by caesarean section - pregnancy at term |
| ZV22.00 | [V]Normal pregnancy |
| ZV22.11 | [V]Supervision of normal pregnancy |
| ZV22000 | [V]First normal pregnancy supervision |
| ZV22100 | [V]Other normal pregnancy supervision |
| ZV22200 | [V]Pregnancy confirmed |
| ZV22300 | [V]Pregnant state, incidental |
| ZV22400 | [V]Supervision of other normal pregnancy |
| ZV22y00 | [V]Other specified pregnant state |
| ZV22z00 | [V]Unspecified pregnant state |
| ZV23.00 | [V]High-risk pregnancy supervision |
| ZV23000 | [V]Pregnancy with history of infertility |
| ZV23100 | [V]Pregnancy with history of trophoblastic disease |
| ZV23111 | [V]Pregnancy with history of hydatidiform mole |
| ZV23112 | [V]Pregnancy with history of vesicular mole |
| ZV23200 | [V]Pregnancy with history of abortion |
| ZV23400 | [V]Pregnancy with other poor obstetric history |
| ZV23500 | [V]Pregnancy with other poor reproductive history |
| ZV23600 | [V]Supervisn/pregnancy wth history insufficnt antenatal care |
| ZV23800 | [V]Supervision of high-risk pregnancy due to social problems |
| ZV23y00 | [V]Other specified high-risk pregnancy |
| ZV23z00 | [V]Unspecified high-risk pregnancy |
| ZV25311 | [V]Admission for termination of pregnancy (TOP) |
| ZV25313 | [V]Admission for termination of pregnancy |
| ZV4J000 | [V]Problems related to unwanted pregnancy |
| ZV61800 | [V]Illegitimate pregnancy |
| ZV61900 | [V]Other unwanted pregnancy |
| ZVu2300 | [X]Supervision of other normal pregnancy |

## Renal Disease

Myoglobinuria

| **Read Code** | **Description** |
| --- | --- |
| **Those we believe should be included** | |
| R113.00 | [D]Myoglobinuria |

Renal disease

| **Read Code** | **Description** |
| --- | --- |
| **Those we believe should be included** | |
| G22..00 | Hypertensive renal disease |
| G220.00 | Malignant hypertensive renal disease |
| G221.00 | Benign hypertensive renal disease |
| G222.00 | Hypertensive renal disease with renal failure |
| G22z.00 | Hypertensive renal disease NOS |
| G23..00 | Hypertensive heart and renal disease |
| G230.00 | Malignant hypertensive heart and renal disease |
| G231.00 | Benign hypertensive heart and renal disease |
| G233.00 | Hypertensive heart and renal disease with renal failure |
| G23z.00 | Hypertensive heart and renal disease NOS |

Renal disease (Vaccine High Risk)

| **Read Code** | **Description** |
| --- | --- |
| **Those we believe should be included** | |
| 1Z1..00 | Chronic renal impairment |
| 1Z10.00 | Chronic kidney disease stage 1 |
| 1Z11.00 | Chronic kidney disease stage 2 |
| 1Z12.00 | Chronic kidney disease stage 3 |
| 1Z13.00 | Chronic kidney disease stage 4 |
| 1Z14.00 | Chronic kidney disease stage 5 |
| K01..00 | Nephrotic syndrome |
| K010.00 | Nephrotic syndrome with proliferative glomerulonephritis |
| K011.00 | Nephrotic syndrome with membranous glomerulonephritis |
| K012.00 | Nephrotic syndrome+membranoproliferative glomerulonephritis |
| K013.00 | Nephrotic syndrome with minimal change glomerulonephritis |
| K013.11 | Lipoid nephrosis |
| K013.12 | Steroid sensitive nephrotic syndrome |
| K014.00 | Nephrotic syndrome, minor glomerular abnormality |
| K015.00 | Nephrotic syndrome, focal and segmental glomerular lesions |
| K016.00 | Nephrotic syndrome, diffuse membranous glomerulonephritis |
| K017.00 | Nephrotic syn difus mesangial prolifertiv glomerulonephritis |
| K018.00 | Nephrotic syn,difus endocapilary proliftv glomerulonephritis |
| K019.00 | Nephrotic syn,diffuse mesangiocapillary glomerulonephritis |
| K01A.00 | Nephrotic syndrome, dense deposit disease |
| K01B.00 | Nephrotic syndrome, diffuse crescentic glomerulonephritis |
| K01w.00 | Congenital nephrotic syndrome |
| K01w000 | Finnish nephrosis syndrome |
| K01w011 | Microcystic type congenital nephrotic syndrome |
| K01wz00 | Congenital nephrotic syndrome NOS |
| K01x.00 | Nephrotic syndrome in diseases EC |
| K01x000 | Nephrotic syndrome in amyloidosis |
| K01x100 | Nephrotic syndrome in diabetes mellitus |
| K01x111 | Kimmelstiel - Wilson disease |
| K01x200 | Nephrotic syndrome in malaria |
| K01x300 | Nephrotic syndrome in polyarteritis nodosa |
| K01x400 | Nephrotic syndrome in systemic lupus erythematosus |
| K01x411 | Lupus nephritis |
| K01xz00 | Nephrotic syndrome in diseases EC NOS |
| K01y.00 | Nephrotic syndrome with other pathological kidney lesions |
| K01z.00 | Nephrotic syndrome NOS |
| K02..00 | Chronic glomerulonephritis |
| K02..11 | Nephritis - chronic |
| K02..12 | Nephropathy - chronic |
| K020.00 | Chronic proliferative glomerulonephritis |
| K021.00 | Chronic membranous glomerulonephritis |
| K022.00 | Chronic membranoproliferative glomerulonephritis |
| K023.00 | Chronic rapidly progressive glomerulonephritis |
| K02y.00 | Other chronic glomerulonephritis |
| K02y000 | Chronic glomerulonephritis + diseases EC |
| K02y100 | Chronic exudative glomerulonephritis |
| K02y200 | Chronic focal glomerulonephritis |
| K02y300 | Chronic diffuse glomerulonephritis |
| K02yz00 | Other chronic glomerulonephritis NOS |
| K02z.00 | Chronic glomerulonephritis NOS |
| K05..00 | Chronic renal failure |
| K05..11 | Chronic uraemia |
| K05..12 | End stage renal failure |
| K050.00 | End stage renal failure |
| K0A3.00 | Chronic nephritic syndrome |
| K0A3000 | Chronic nephritic syndrome, minor glomerular abnormality |
| K0A3100 | Chronic nephritic syndrm focal+segmental glomerular lesions |
| K0A3200 | Chron nephritic syndrom difuse membranous glomerulonephritis |
| K0A3300 | Chron neph syn difus mesangial prolifrtiv glomerulonephritis |
| K0A3400 | Chron neph syn difuse endocap prolifrativ glomerulonephritis |
| K0A3500 | Chronic neph syn difus mesangiocapillary glomerulonephritis |
| K0A3600 | Chronic nephritic syndrome, dense deposit disease |
| K0A3700 | Chronic nephritic syn diffuse crescentic glomerulonephritis |
| K0D..00 | End-stage renal disease |
| 7B00.00 | Transplantation of kidney |
| 7B00000 | Autotransplant of kidney |
| 7B00100 | Transplantation of kidney from live donor |
| 7B00111 | Allotransplantation of kidney from live donor |
| 7B00200 | Transplantation of kidney from cadaver |
| 7B00211 | Allotransplantation of kidney from cadaver |
| 7B00300 | Allotransplantation of kidney from cadaver, heart-beating |
| 7B00400 | Allotransplantation kidney from cadaver, heart non-beating |
| 7B00500 | Allotransplantation of kidney from cadaver NEC |
| 7B00y00 | Other specified transplantation of kidney |
| 7B00z00 | Transplantation of kidney NOS |
| 7B01.00 | Total nephrectomy |
| 7B01.11 | Total excision of kidney |
| 7B01000 | Radical nephrectomy |
| 7B01011 | Nephrectomy and excision of perirenal tissue |
| 7B01100 | Nephroureterectomy-unspecified |
| 7B01200 | Bilateral nephrectomy |
| 7B01300 | Heminephrectomy for horseshoe kidney |
| 7B01311 | Excision of half of horseshoe kidney |
| 7B01400 | Simple nephrectomy - other |
| 7B01500 | Transplant nephrectomy |
| 7B01511 | Excision of rejected transplanted kidney |
| 7B01600 | Simple nephrectomy -live donor |
| 7B01700 | Nephroureterectomy with open lower ureterectomy |
| 7B01800 | Nephroureterectomy with pluck lower ureterectomy |
| 7B01y00 | Other specified total nephrectomy |
| 7B01z00 | Total nephrectomy NOS |
| 7B06300 | Exploration of renal transplant |
| 8L50.00 | Renal transplant planned |
| SP08300 | Kidney transplant failure and rejection |
| TB00100 | Kidney transplant with complication, without blame |
| TB00111 | Renal transplant with complication, without blame |
| ZV42000 | [V]Kidney transplanted |

## Respiratory Disease

Asthma

| **Read Code** | **Description** |
| --- | --- |
| **Those we believe should be included** | |
| G581.11 | Asthma - cardiac |
| 1O2..00 | Asthma confirmed |

Chronic respiratory disease (Vaccine High Risk)

| **Read Code** | **Description** |
| --- | --- |
| **Those we believe should be included** | |
| H3...00 | Chronic obstructive pulmonary disease |
| H3...11 | Chronic obstructive airways disease |
| H31..00 | Chronic bronchitis |
| H310.00 | Simple chronic bronchitis |
| H310000 | Chronic catarrhal bronchitis |
| H310100 | Smokers' cough |
| H310z00 | Simple chronic bronchitis NOS |
| H311.00 | Mucopurulent chronic bronchitis |
| H311000 | Purulent chronic bronchitis |
| H311100 | Fetid chronic bronchitis |
| H311z00 | Mucopurulent chronic bronchitis NOS |
| H312.00 | Obstructive chronic bronchitis |
| H312000 | Chronic asthmatic bronchitis |
| H312011 | Chronic wheezy bronchitis |
| H312100 | Emphysematous bronchitis |
| H312200 | Acute exacerbation of chronic obstructive airways disease |
| H312300 | Bronchiolitis obliterans |
| H312z00 | Obstructive chronic bronchitis NOS |
| H313.00 | Mixed simple and mucopurulent chronic bronchitis |
| H31y.00 | Other chronic bronchitis |
| H31y000 | Chronic tracheitis |
| H31y100 | Chronic tracheobronchitis |
| H31yz00 | Other chronic bronchitis NOS |
| H31z.00 | Chronic bronchitis NOS |
| H32..00 | Emphysema |
| H320.00 | Chronic bullous emphysema |
| H320000 | Segmental bullous emphysema |
| H320100 | Zonal bullous emphysema |
| H320200 | Giant bullous emphysema |
| H320300 | Bullous emphysema with collapse |
| H320311 | Tension pneumatocoele |
| H320z00 | Chronic bullous emphysema NOS |
| H321.00 | Panlobular emphysema |
| H322.00 | Centrilobular emphysema |
| H32y.00 | Other emphysema |
| H32y000 | Acute vesicular emphysema |
| H32y100 | Atrophic (senile) emphysema |
| H32y111 | Acute interstitial emphysema |
| H32y200 | MacLeod's unilateral emphysema |
| H32yz00 | Other emphysema NOS |
| H32yz11 | Sawyer - Jones syndrome |
| H32z.00 | Emphysema NOS |
| H33..00 | Asthma |
| H33..11 | Bronchial asthma |
| H330.00 | Extrinsic (atopic) asthma |
| H330.11 | Allergic asthma |
| H330.12 | Childhood asthma |
| H330.13 | Hay fever with asthma |
| H330.14 | Pollen asthma |
| H330000 | Extrinsic asthma without status asthmaticus |
| H330011 | Hay fever with asthma |
| H330100 | Extrinsic asthma with status asthmaticus |
| H330111 | Extrinsic asthma with asthma attack |
| H330z00 | Extrinsic asthma NOS |
| H331.00 | Intrinsic asthma |
| H331.11 | Late onset asthma |
| H331000 | Intrinsic asthma without status asthmaticus |
| H331100 | Intrinsic asthma with status asthmaticus |
| H331111 | Intrinsic asthma with asthma attack |
| H331z00 | Intrinsic asthma NOS |
| H332.00 | Mixed asthma |
| H333.00 | Acute exacerbation of asthma |
| H334.00 | Brittle asthma |
| H33z.00 | Asthma unspecified |
| H33z.11 | Hyperreactive airways disease |
| H33z000 | Status asthmaticus NOS |
| H33z011 | Severe asthma attack |
| H33z100 | Asthma attack |
| H33z111 | Asthma attack NOS |
| H33z200 | Late-onset asthma |
| H33zz00 | Asthma NOS |
| H33zz11 | Exercise induced asthma |
| H33zz12 | Allergic asthma NEC |
| H33zz13 | Allergic bronchitis NEC |
| H34..00 | Bronchiectasis |
| H340.00 | Recurrent bronchiectasis |
| H341.00 | Post-infective bronchiectasis |
| H34z.00 | Bronchiectasis NOS |
| H35..00 | Extrinsic allergic alveolitis |
| H350.00 | Farmers' lung |
| H351.00 | Bagassosis |
| H352.00 | Bird-fancier's lung |
| H352000 | Budgerigar-fanciers' lung |
| H352100 | Pigeon-fanciers' lung |
| H352z00 | Bird-fancier's lung NOS |
| H353.00 | Suberosis ( cork-handlers' lung ) |
| H354.00 | Malt workers' lung |
| H355.00 | Mushroom workers' lung |
| H356.00 | Maple bark strippers' lung |
| H357.00 | "Ventilation" pneumonitis |
| H35y.00 | Other allergic alveolitis |
| H35y000 | Cheese-washers' lung |
| H35y100 | Coffee-workers' lung |
| H35y200 | Fish-meal workers' lung |
| H35y300 | Furriers' lung |
| H35y400 | Grain-handlers' disease |
| H35y500 | Pituitary snuff-takers' disease |
| H35y600 | Sequoiosis (red-cedar asthma) |
| H35y700 | Wood asthma |
| H35y800 | Air-conditioner and humidifier lung |
| H35yz00 | Other allergic alveolitis NOS |
| H35z.00 | Allergic alveolitis and pneumonitis NOS |
| H35z000 | Allergic extrinsic alveolitis NOS |
| H35z100 | Hypersensitivity pneumonitis NOS |
| H35zz00 | Allergic alveolitis and pneumonitis NOS |
| H36..00 | Mild chronic obstructive pulmonary disease |
| H37..00 | Moderate chronic obstructive pulmonary disease |
| H38..00 | Severe chronic obstructive pulmonary disease |
| H3y..00 | Other specified chronic obstructive airways disease |
| H3y..11 | Other specified chronic obstructive pulmonary disease |
| H3y0.00 | Chronic obstruct pulmonary dis with acute lower resp infectn |
| H3y1.00 | Chron obstruct pulmonary dis wth acute exacerbation, unspec |
| H3z..00 | Chronic obstructive airways disease NOS |
| H3z..11 | Chronic obstructive pulmonary disease NOS |
| H4...00 | Lung disease due to external agents |
| H4...11 | Pneumoconioses |
| H4...12 | Occupational lung disease |
| H40..00 | Coal workers' pneumoconiosis |
| H41..00 | Asbestosis |
| H410.00 | Pleural plaque disease due to asbestosis |
| H41z.00 | Asbestosis NOS |
| H42..00 | Silica and silicate pneumoconiosis |
| H420.00 | Talc pneumoconiosis |
| H421.00 | Simple silicosis |
| H422.00 | Complicated silicosis |
| H423.00 | Massive silicotic fibrosis |
| H42z.00 | Silica pneumoconiosis NOS |
| H43..00 | Pneumoconiosis due to other inorganic dust |
| H430.00 | Aluminosis of lung |
| H431.00 | Bauxite fibrosis of lung |
| H432.00 | Berylliosis |
| H433.00 | Graphite fibrosis of lung |
| H434.00 | Siderosis |
| H435.00 | Stannosis |
| H43z.00 | Pneumoconiosis due to inorganic dust NOS |
| H44..00 | Pneumopathy due to inhalation of other dust |
| H440.00 | Byssinosis |
| H441.00 | Cannabinosis |
| H442.00 | Flax-dressers' disease |
| H44z.00 | Pneumopathy due to inhalation of other dust NOS |
| H45..00 | Pneumoconiosis NOS |
| H450.00 | Pneumoconiosis associated with tuberculosis |
| H46..00 | Respiratory disease due to chemical fumes and vapours |
| H460.00 | Bronchitis and pneumonitis due to chemical fumes |
| H460000 | Acute bronchitis due to chemical fumes |
| H460100 | Acute pneumonitis due to chemical fumes |
| H460z00 | Bronchitis and pneumonitis due to chemical fumes NOS |
| H461.00 | Acute pulmonary oedema due to chemical fumes |
| H462.00 | Upper respiratory inflammation due to chemical fumes |
| H463.00 | Other acute respiratory diseases due to chemical fumes |
| H464.00 | Chronic respiratory conditions due to chemical fumes |
| H464000 | Chronic emphysema due to chemical fumes |
| H464100 | Obliterative bronchiolitis due to chemical fumes |
| H464200 | Chronic pulmonary fibrosis due to chemical fumes |
| H464z00 | Chronic respiratory conditions due to chemical fumes NOS |
| H46z.00 | Respiratory conditions due to chemical fumes NOS |
| H46z000 | Silo-fillers' disease |
| H46zz00 | Respiratory conditions due to chemical fumes NOS |
| H47..00 | Pneumonitis due to inhalation of solids or liquids |
| H47..11 | Aspiration pneumonitis |
| H47y.00 | Pneumonitis due to inhalation of other solid or liquid |
| H47y000 | Detergent asthma |
| H47yz00 | Pneumonitis due to inhalation of solid or liquid NOS |
| H47z.00 | Pneumonitis due to inhalation of solid or liquid NOS |
| H48..00 | Progressive massive fibrosis |
| H4y..00 | Other specified lung diseases due to external agent |
| H4y0.00 | Acute pulmonary radiation disease |
| H4y0000 | Acute radiation pneumonitis |
| H4y0z00 | Acute pulmonary radiation disease NOS |
| H4y1.00 | Chronic pulmonary radiation disease |
| H4y1000 | Chronic pulmonary fibrosis following radiation |
| H4y1z00 | Chronic pulmonary radiation disease NOS |
| H4y2.00 | Drug-induced interstitial lung disorders |
| H4y2000 | Acute drug-induced interstitial lung disorders |
| H4y2100 | Chronic drug-induced interstitial lung disorders |
| H4yy.00 | Other external agent causing respiratory condition |
| H4yz.00 | External agent causing respiratory conditions NOS |
| H4z..00 | Lung disease due to external agents NOS |
| H541000 | Chronic pulmonary oedema |
| H55..00 | Postinflammatory pulmonary fibrosis |
| H55..11 | Cirrhosis of lung |
| H562.00 | Pulmonary alveolar microlithiasis |
| H563.00 | Idiopathic fibrosing alveolitis |
| H563.11 | Hamman - Rich syndrome |
| H563.12 | Cryptogenic fibrosing alveolitis |
| H563000 | Alveolar capillary block |
| H563100 | Diffuse pulmonary fibrosis |
| H563z00 | Idiopathic fibrosing alveolitis NOS |
| H57..00 | Lung involvement in diseases EC |
| H570.00 | Rheumatoid lung |
| H571.00 | Rheumatic pneumonia |
| H572.00 | Lung disease with systemic sclerosis |
| H57y.00 | Lung disease with diseases EC |
| H57y000 | Pulmonary amyloidosis |
| H57y100 | Lung disease with polymyositis |
| H57y200 | Pulmonary sarcoidosis |
| H57y300 | Lung disease with Sjogren's disease |
| H57y400 | Lung disease with systemic lupus erythematosus |
| H57y500 | Lung disease with syphilis |
| H57yz00 | Lung disease with diseases EC NOS |
| H581.00 | Interstitial emphysema |
| H581.11 | Pneumomediastinum |
| H582.00 | Compensatory emphysema |
| H583.00 | Pulmonary eosinophilia |
| H583000 | Loeffler's syndrome |
| H583100 | Tropical eosinophilia |
| H583z00 | Pulmonary eosinophilia NOS |
| H59..00 | Respiratory failure |
| H591.00 | Chronic respiratory failure |
| Hyu3.00 | [X]Chronic lower respiratory diseases |
| Hyu3000 | [X]Other emphysema |
| Hyu3100 | [X]Other specified chronic obstructive pulmonary disease |
| Hyu4000 | [X]Pneumoconiosis due to other dust containing silica |
| Hyu4100 | [X]Pneumoconiosis due to other specified inorganic dusts |
| Hyu4800 | [X]Chronic+other pulmonary manifestations due to radiation |
| Hyu5.00 | [X]Other resp diseases principally affecting interstitium |
| Hyu5000 | [X]Other interstitial pulmonary diseases with fibrosis |
| Hyu5100 | [X]Other specified interstitial pulmonary diseases |
| Hyu5200 | [X]Hepatopulmonary syndrome |
| Q317.00 | Perinatal chronic respiratory disease |
| Q317000 | Perinatal bronchopulmonary dysplasia |
| Q317100 | Prematurity with interstitial pulmonary fibrosis |
| Q317200 | Wilson-Mikity syndrome |
| Q317y00 | Other specified perinatal chronic respiratory disease |
| Q317y11 | Perinatal pulmonary fibroplasia |
| Q317z00 | Perinatal chronic respiratory disease NOS |

Emphysema

| **Read Code** | **Description** |
| --- | --- |
| **Those we believe should be included** | |
| Q312.00 | Perinatal interstitial emphysema and related conditions |
| Q312111 | Perinatal mediastinal emphysema |
| Q312300 | Perinatal interstitial emphysema |
| Q312y00 | Perinatal interstitial emphysema or related condition OS |
| Q312z00 | Perinatal interstitial emphysema or related condition NOS |
| Qyu3400 | [X]Oth conds relat/interstial emphysema orig perinatl period |
| SK07.00 | Subcutaneous emphysema |

Laryngitis

| **Read Code** | **Description** |
| --- | --- |
| **Those we believe should be included** | |
| H16..00 | Chronic laryngitis and laryngotracheitis |
| H160.00 | Chronic laryngitis |
| H160000 | Chronic simple laryngitis |
| H160z00 | Chronic laryngitis NOS |
| H16z.00 | Chronic laryngitis NOS |
| H271000 | Influenza with laryngitis |
| H055.00 | Pharyngolaryngitis |
| H160100 | Chronic catarrhal laryngitis |
| H160200 | Chronic hypertrophic laryngitis |
| H160300 | Chronic atrophic laryngitis |
| H160400 | Laryngitis sicca |

Nasopharyngitis

| **Read Code** | **Description** |
| --- | --- |
| **Those we believe should be included** | |
| H12..00 | Chronic pharyngitis and nasopharyngitis |
| H122.00 | Chronic nasopharyngitis |
| H12z.00 | Chronic pharyngitis and nasopharyngitis NOS |

Pharyngitis

| **Read Code** | **Description** |
| --- | --- |
| **Those we believe should be included** | |
| H053.00 | Tracheopharyngitis |
| H12..00 | Chronic pharyngitis and nasopharyngitis |
| H121.00 | Chronic pharyngitis |
| H121000 | Simple chronic pharyngitis |
| H121z00 | Chronic pharyngitis NOS |
| H122.00 | Chronic nasopharyngitis |
| H12z.00 | Chronic pharyngitis and nasopharyngitis NOS |
| H271100 | Influenza with pharyngitis |
| 2DC2.00 | O/E - granular pharyngitis |
| A340200 | Streptococcal pharyngitis |
| AA12.00 | Vincent's pharyngitis |
| AA25.11 | Rhinopharyngitis mutilans |
| H121100 | Atrophic pharyngitis |
| H121200 | Granular pharyngitis |
| H121300 | Hypertrophic pharyngitis |
| H121400 | Pharyngitis keratosa |
| H121500 | Pharyngitis sicca |
| H121600 | Chronic follicular pharyngitis |

Pneumonia and secondary bacterial pneumonia

| **Read Code** | **Description** |
| --- | --- |
| H56y000 | Endogenous lipoid pneumonia |

Sinusitis

| **Read Code** | **Description** |
| --- | --- |
| **Those we believe should be included** | |
| H01..11 | Sinusitis |
| H01y.00 | Other acute sinusitis |
| H01yz00 | Other acute sinusitis NOS |
| H13..00 | Chronic sinusitis |
| H13..11 | Chronic rhinosinusitis |
| H130.00 | Chronic maxillary sinusitis |
| H130.12 | Maxillary sinusitis |
| H131.00 | Chronic frontal sinusitis |
| H131.11 | Frontal sinusitis |
| H132.00 | Chronic ethmoidal sinusitis |
| H133.00 | Chronic sphenoidal sinusitis |
| H135.00 | Recurrent sinusitis |
| H13y.00 | Other chronic sinusitis |
| H13y000 | Chronic pansinusitis |
| H13y100 | Pansinusitis |
| H13yz00 | Other chronic sinusitis NOS |
| H13z.00 | Chronic sinusitis NOS |
| Hyu0000 | [X]Other acute sinusitis |
| Hyu2200 | [X]Other chronic sinusitis |
| SN31.11 | Aerosinusitis |

## Other Disease

Immunosuppressed

| **Read Code** | **Description** |
| --- | --- |
| **Those we believe should be included** | |
| 14N7.00 | H/O: splenectomy |
| 2J3..00 | General immune status |
| 2J30.00 | Patient immunocompromised |
| 2J31.00 | Patient immunosuppressed |
| 43C3.00 | HTLV-3 antibody positive |
| 43c3.00 | Lupus anticoagulant screening test |
| 43C3.11 | HIV positive |
| 7840.00 | Total excision of spleen |
| 7840.11 | Total splenectomy |
| 7840000 | Total excision of spleen and replantation of fragments |
| 7840100 | Total splenectomy |
| 7840200 | Excision of accessory spleen |
| 7840300 | Splenectomy NEC |
| 7840400 | Laparoscopic total splenectomy |
| 7840y00 | Other specified total excision of spleen |
| 7840z00 | Total excision of spleen NOS |
| A788.00 | Acquired immune deficiency syndrome |
| A788.11 | Human immunodeficiency virus infection |
| A788000 | Acute human immunodeficiency virus infection |
| A788100 | Asymptomatic human immunodeficiency virus infection |
| A788200 | HIV infection with persistent generalised lymphadenopathy |
| A788300 | Human immunodeficiency virus with constitutional disease |
| A788400 | Human immunodeficiency virus with neurological disease |
| A788500 | Human immunodeficiency virus with secondary infection |
| A788600 | Human immunodeficiency virus with secondary cancers |
| A788U00 | HIV disease result/haematological+immunologic abnorms,NEC |
| A788V00 | HIV disease resulting in multiple diseases CE |
| A788W00 | HIV disease resulting in unspecified malignant neoplasm |
| A788X00 | HIV disease resulting/unspcf infectious+parasitic disease |
| A788y00 | Human immunodeficiency virus with other clinical findings |
| A788z00 | Acquired human immunodeficiency virus infection syndrome NOS |
| A789.00 | Human immunodef virus resulting in other disease |
| A789000 | HIV disease resulting in mycobacterial infection |
| A789100 | HIV disease resulting in cytomegaloviral disease |
| A789200 | HIV disease resulting in candidiasis |
| A789300 | HIV disease resulting in Pneumocystis carinii pneumonia |
| A789400 | HIV disease resulting in multiple infections |
| A789500 | HIV disease resulting in Kaposi's sarcoma |
| A789600 | HIV disease resulting in Burkitt's lymphoma |
| A789700 | HIV dis resulting oth types of non-Hodgkin's lymphoma |
| A789800 | HIV disease resulting in multiple malignant neoplasms |
| A789900 | HIV disease resulting in lymphoid interstitial pneumonitis |
| A789A00 | HIV disease resulting in wasting syndrome |
| A789X00 | HIV dis reslt/oth mal neopl/lymph,h'matopoetc+reltd tissu |
| B6...00 | Malignant neoplasm of lymphatic and haemopoietic tissue |
| B6...11 | Malignant neoplasm of histiocytic tissue |
| B60..00 | Lymphosarcoma and reticulosarcoma |
| B600.00 | Reticulosarcoma |
| B600000 | Reticulosarcoma of unspecified site |
| B600100 | Reticulosarcoma of lymph nodes of head, face and neck |
| B600200 | Reticulosarcoma of intrathoracic lymph nodes |
| B600300 | Reticulosarcoma of intra-abdominal lymph nodes |
| B600400 | Reticulosarcoma of lymph nodes of axilla and upper limb |
| B600500 | Reticulosarcoma of lymph nodes of inguinal region and leg |
| B600600 | Reticulosarcoma of intrapelvic lymph nodes |
| B600700 | Reticulosarcoma of spleen |
| B600800 | Reticulosarcoma of lymph nodes of multiple sites |
| B600z00 | Reticulosarcoma NOS |
| B601.00 | Lymphosarcoma |
| B601000 | Lymphosarcoma of unspecified site |
| B601100 | Lymphosarcoma of lymph nodes of head, face and neck |
| B601200 | Lymphosarcoma of intrathoracic lymph nodes |
| B601300 | Lymphosarcoma of intra-abdominal lymph nodes |
| B601400 | Lymphosarcoma of lymph nodes of axilla and upper limb |
| B601500 | Lymphosarcoma of lymph nodes of inguinal region and leg |
| B601600 | Lymphosarcoma of intrapelvic lymph nodes |
| B601700 | Lymphosarcoma of spleen |
| B601800 | Lymphosarcoma of lymph nodes of multiple sites |
| B601z00 | Lymphosarcoma NOS |
| B602.00 | Burkitt's lymphoma |
| B602000 | Burkitt's lymphoma of unspecified site |
| B602100 | Burkitt's lymphoma of lymph nodes of head, face and neck |
| B602200 | Burkitt's lymphoma of intrathoracic lymph nodes |
| B602300 | Burkitt's lymphoma of intra-abdominal lymph nodes |
| B602400 | Burkitt's lymphoma of lymph nodes of axilla and upper limb |
| B602500 | Burkitt's lymphoma of lymph nodes of inguinal region and leg |
| B602600 | Burkitt's lymphoma of intrapelvic lymph nodes |
| B602700 | Burkitt's lymphoma of spleen |
| B602800 | Burkitt's lymphoma of lymph nodes of multiple sites |
| B602z00 | Burkitt's lymphoma NOS |
| B60y.00 | Other specified reticulosarcoma or lymphosarcoma |
| B60z.00 | Reticulosarcoma or lymphosarcoma NOS |
| B61..00 | Hodgkin's disease |
| B610.00 | Hodgkin's paragranuloma |
| B610000 | Hodgkin's paragranuloma of unspecified site |
| B610100 | Hodgkin's paragranuloma of lymph nodes of head, face, neck |
| B610200 | Hodgkin's paragranuloma of intrathoracic lymph nodes |
| B610300 | Hodgkin's paragranuloma of intra-abdominal lymph nodes |
| B610400 | Hodgkin's paragranuloma of lymph nodes of axilla and arm |
| B610500 | Hodgkin's paragranuloma lymph nodes inguinal region and leg |
| B610600 | Hodgkin's paragranuloma of intrapelvic lymph nodes |
| B610700 | Hodgkin's paragranuloma of spleen |
| B610800 | Hodgkin's paragranuloma of lymph nodes of multiple sites |
| B610z00 | Hodgkin's paragranuloma NOS |
| B611.00 | Hodgkin's granuloma |
| B611000 | Hodgkin's granuloma of unspecified site |
| B611100 | Hodgkin's granuloma of lymph nodes of head, face and neck |
| B611200 | Hodgkin's granuloma of intrathoracic lymph nodes |
| B611300 | Hodgkin's granuloma of intra-abdominal lymph nodes |
| B611400 | Hodgkin's granuloma of lymph nodes of axilla and upper limb |
| B611500 | Hodgkin's granuloma lymph nodes of inguinal region and leg |
| B611600 | Hodgkin's granuloma of intrapelvic lymph nodes |
| B611700 | Hodgkin's granuloma of spleen |
| B611800 | Hodgkin's granuloma of lymph nodes of multiple sites |
| B611z00 | Hodgkin's granuloma NOS |
| B612.00 | Hodgkin's sarcoma |
| B612000 | Hodgkin's sarcoma of unspecified site |
| B612100 | Hodgkin's sarcoma of lymph nodes of head, face and neck |
| B612200 | Hodgkin's sarcoma of intrathoracic lymph nodes |
| B612300 | Hodgkin's sarcoma of intra-abdominal lymph nodes |
| B612400 | Hodgkin's sarcoma of lymph nodes of axilla and upper limb |
| B612500 | Hodgkin's sarcoma of lymph nodes of inguinal region and leg |
| B612600 | Hodgkin's sarcoma of intrapelvic lymph nodes |
| B612700 | Hodgkin's sarcoma of spleen |
| B612800 | Hodgkin's sarcoma of lymph nodes of multiple sites |
| B612z00 | Hodgkin's sarcoma NOS |
| B613.00 | Hodgkin's disease, lymphocytic-histiocytic predominance |
| B613000 | Hodgkin's, lymphocytic-histiocytic predominance unspec site |
| B613100 | Hodgkin's, lymphocytic-histiocytic pred of head, face, neck |
| B613200 | Hodgkin's, lymphocytic-histiocytic pred intrathoracic nodes |
| B613300 | Hodgkin's, lymphocytic-histiocytic pred intra-abdominal node |
| B613400 | Hodgkin's, lymphocytic-histiocytic pred axilla and arm |
| B613500 | Hodgkin's, lymphocytic-histiocytic pred inguinal and leg |
| B613600 | Hodgkin's, lymphocytic-histiocytic pred intrapelvic nodes |
| B613700 | Hodgkin's, lymphocytic-histiocytic predominance of spleen |
| B613800 | Hodgkin's, lymphocytic-histiocytic pred of multiple sites |
| B613z00 | Hodgkin's, lymphocytic-histiocytic predominance NOS |
| B614.00 | Hodgkin's disease, nodular sclerosis |
| B614000 | Hodgkin's disease, nodular sclerosis of unspecified site |
| B614100 | Hodgkin's nodular sclerosis of head, face and neck |
| B614200 | Hodgkin's nodular sclerosis of intrathoracic lymph nodes |
| B614300 | Hodgkin's nodular sclerosis of intra-abdominal lymph nodes |
| B614400 | Hodgkin's nodular sclerosis of lymph nodes of axilla and arm |
| B614500 | Hodgkin's nodular sclerosis of inguinal region and leg |
| B614600 | Hodgkin's nodular sclerosis of intrapelvic lymph nodes |
| B614700 | Hodgkin's disease, nodular sclerosis of spleen |
| B614800 | Hodgkin's nodular sclerosis of lymph nodes of multiple sites |
| B614z00 | Hodgkin's disease, nodular sclerosis NOS |
| B615.00 | Hodgkin's disease, mixed cellularity |
| B615000 | Hodgkin's disease, mixed cellularity of unspecified site |
| B615100 | Hodgkin's mixed cellularity of lymph nodes head, face, neck |
| B615200 | Hodgkin's mixed cellularity of intrathoracic lymph nodes |
| B615300 | Hodgkin's mixed cellularity of intra-abdominal lymph nodes |
| B615400 | Hodgkin's mixed cellularity of lymph nodes of axilla and arm |
| B615500 | Hodgkin's mixed cellularity of lymph nodes inguinal and leg |
| B615600 | Hodgkin's mixed cellularity of intrapelvic lymph nodes |
| B615700 | Hodgkin's disease, mixed cellularity of spleen |
| B615800 | Hodgkin's mixed cellularity of lymph nodes of multiple sites |
| B615z00 | Hodgkin's disease, mixed cellularity NOS |
| B616.00 | Hodgkin's disease, lymphocytic depletion |
| B616000 | Hodgkin's lymphocytic depletion of unspecified site |
| B616100 | Hodgkin's lymphocytic depletion of head, face and neck |
| B616200 | Hodgkin's lymphocytic depletion of intrathoracic lymph nodes |
| B616300 | Hodgkin's lymphocytic depletion intra-abdominal lymph nodes |
| B616400 | Hodgkin's lymphocytic depletion lymph nodes axilla and arm |
| B616500 | Hodgkin's lymphocytic depletion lymph nodes inguinal and leg |
| B616600 | Hodgkin's lymphocytic depletion of intrapelvic lymph nodes |
| B616700 | Hodgkin's disease, lymphocytic depletion of spleen |
| B616800 | Hodgkin's lymphocytic depletion lymph nodes multiple sites |
| B616z00 | Hodgkin's disease, lymphocytic depletion NOS |
| B61z.00 | Hodgkin's disease NOS |
| B61z000 | Hodgkin's disease NOS, unspecified site |
| B61z100 | Hodgkin's disease NOS of lymph nodes of head, face and neck |
| B61z200 | Hodgkin's disease NOS of intrathoracic lymph nodes |
| B61z300 | Hodgkin's disease NOS of intra-abdominal lymph nodes |
| B61z400 | Hodgkin's disease NOS of lymph nodes of axilla and arm |
| B61z500 | Hodgkin's disease NOS of lymph nodes inguinal region and leg |
| B61z600 | Hodgkin's disease NOS of intrapelvic lymph nodes |
| B61z700 | Hodgkin's disease NOS of spleen |
| B61z800 | Hodgkin's disease NOS of lymph nodes of multiple sites |
| B61zz00 | Hodgkin's disease NOS |
| B62..00 | Other malignant neoplasm of lymphoid and histiocytic tissue |
| B620.00 | Nodular lymphoma (Brill - Symmers disease) |
| B620.11 | Reticulosarcoma - follicular or nodular |
| B620000 | Nodular lymphoma of unspecified site |
| B620100 | Nodular lymphoma of lymph nodes of head, face and neck |
| B620200 | Nodular lymphoma of intrathoracic lymph nodes |
| B620300 | Nodular lymphoma of intra-abdominal lymph nodes |
| B620400 | Nodular lymphoma of lymph nodes of axilla and upper limb |
| B620500 | Nodular lymphoma of lymph nodes of inguinal region and leg |
| B620600 | Nodular lymphoma of intrapelvic lymph nodes |
| B620700 | Nodular lymphoma of spleen |
| B620800 | Nodular lymphoma of lymph nodes of multiple sites |
| B620z00 | Nodular lymphoma NOS |
| B621.00 | Mycosis fungoides |
| B621000 | Mycosis fungoides of unspecified site |
| B621100 | Mycosis fungoides of the lymph nodes of head, face and neck |
| B621200 | Mycosis fungoides of intrathoracic lymph nodes |
| B621300 | Mycosis fungoides of intra-abdominal lymph nodes |
| B621400 | Mycosis fungoides of lymph nodes of axilla and upper limb |
| B621500 | Mycosis fungoides of lymph nodes of inguinal region and leg |
| B621600 | Mycosis fungoides of intrapelvic lymph nodes |
| B621700 | Mycosis fungoides of spleen |
| B621800 | Mycosis fungoides of lymph nodes of multiple sites |
| B621z00 | Mycosis fungoides NOS |
| B622.00 | Sezary's disease |
| B622000 | Sezary's disease of unspecified site |
| B622100 | Sezary's disease of lymph nodes of head, face and neck |
| B622200 | Sezary's disease of intrathoracic lymph nodes |
| B622300 | Sezary's disease of intra-abdominal lymph nodes |
| B622400 | Sezary's disease of lymph nodes of axilla and upper limb |
| B622500 | Sezary's disease of lymph nodes of inguinal region and leg |
| B622600 | Sezary's disease of intrapelvic lymph nodes |
| B622700 | Sezary's disease of spleen |
| B622800 | Sezary's disease of lymph nodes of multiple sites |
| B622z00 | Sezary's disease NOS |
| B623.00 | Malignant histiocytosis |
| B623000 | Malignant histiocytosis of unspecified site |
| B623100 | Malignant histiocytosis of lymph nodes head, face and neck |
| B623200 | Malignant histiocytosis of intrathoracic lymph nodes |
| B623300 | Malignant histiocytosis of intra-abdominal lymph nodes |
| B623400 | Malignant histiocytosis of lymph nodes of axilla and arm |
| B623500 | Malignant histiocytosis of lymph nodes inguinal and leg |
| B623600 | Malignant histiocytosis of intrapelvic lymph nodes |
| B623700 | Malignant histiocytosis of spleen |
| B623800 | Malignant histiocytosis of lymph nodes of multiple sites |
| B623z00 | Malignant histiocytosis NOS |
| B624.00 | Leukaemic reticuloendotheliosis |
| B624.11 | Leukaemic reticuloendotheliosis |
| B624000 | Leukaemic reticuloendotheliosis of unspecified sites |
| B624100 | Leukaemic reticuloend of lymph nodes of head, face and neck |
| B624200 | Leukaemic reticuloendotheliosis of intrathoracic lymph nodes |
| B624300 | Leukaemic reticuloend of intra-abdominal lymph nodes |
| B624400 | Leukaemic reticuloend of lymph nodes of axilla and arm |
| B624500 | Leukaemic reticuloend of lymph nodes inguinal region and leg |
| B624600 | Leukaemic reticuloendotheliosis of intrapelvic lymph nodes |
| B624700 | Leukaemic reticuloendotheliosis of spleen |
| B624800 | Leukaemic reticuloend of lymph nodes of multiple sites |
| B624z00 | Leukaemic reticuloendotheliosis NOS |
| B625.00 | Letterer-Siwe disease |
| B625.11 | Histiocytosis X (acute, progressive) |
| B625000 | Letterer-Siwe disease of unspecified sites |
| B625100 | Letterer-Siwe disease of lymph nodes of head, face and neck |
| B625200 | Letterer-Siwe disease of intrathoracic lymph nodes |
| B625300 | Letterer-Siwe disease of intra-abdominal lymph nodes |
| B625400 | Letterer-Siwe disease of lymph nodes of axilla and arm |
| B625500 | Letterer-Siwe disease of lymph nodes inguinal region and leg |
| B625600 | Letterer-Siwe disease of intrapelvic lymph nodes |
| B625700 | Letterer-Siwe disease of spleen |
| B625800 | Letterer-Siwe disease of lymph nodes of multiple sites |
| B625z00 | Letterer-Siwe disease NOS |
| B626.00 | Malignant mast cell tumours |
| B626000 | Mast cell malignancy of unspecified site |
| B626100 | Mast cell malignancy of lymph nodes of head, face and neck |
| B626200 | Mast cell malignancy of intrathoracic lymph nodes |
| B626300 | Mast cell malignancy of intra-abdominal lymph nodes |
| B626400 | Mast cell malignancy of lymph nodes of axilla and upper limb |
| B626500 | Mast cell malignancy of lymph nodes inguinal region and leg |
| B626600 | Mast cell malignancy of intrapelvic lymph nodes |
| B626700 | Mast cell malignancy of spleen |
| B626800 | Mast cell malignancy of lymph nodes of multiple sites |
| B626z00 | Malignant mast cell tumour NOS |
| B627.00 | Non - Hodgkin's lymphoma |
| B627000 | Follicular non-Hodgkin's small cleaved cell lymphoma |
| B627100 | Follicular non-Hodg mixed sml cleavd & lge cell lymphoma |
| B627200 | Follicular non-Hodgkin's large cell lymphoma |
| B627300 | Diffuse non-Hodgkin's small cell (diffuse) lymphoma |
| B627400 | Diffuse non-Hodgkin's small cleaved cell (diffuse) lymphoma |
| B627500 | Diffuse non-Hodgkin mixed sml & lge cell (diffuse) lymphoma |
| B627600 | Diffuse non-Hodgkin's immunoblastic (diffuse) lymphoma |
| B627700 | Diffuse non-Hodgkin's lymphoblastic (diffuse) lymphoma |
| B627800 | Diffuse non-Hodgkin's lymphoma undifferentiated (diffuse) |
| B627B00 | Other types of follicular non-Hodgkin's lymphoma |
| B627C00 | Follicular non-Hodgkin's lymphoma |
| B627C11 | Follicular lymphoma NOS |
| B627D00 | Diffuse non-Hodgkin's centroblastic lymphoma |
| B627W00 | Unspecified B-cell non-Hodgkin's lymphoma |
| B627X00 | Diffuse non-Hodgkin's lymphoma, unspecified |
| B62x.00 | Malignant lymphoma otherwise specified |
| B62x000 | T-zone lymphoma |
| B62x100 | Lymphoepithelioid lymphoma |
| B62x200 | Peripheral T-cell lymphoma |
| B62x300 | Malignant reticuloendotheliosis |
| B62x400 | Malignant reticulosis |
| B62x500 | Malignant immunoproliferative small intestinal disease |
| B62x600 | True histiocytic lymphoma |
| B62xX00 | Oth and unspecif peripheral & cutaneous T-cell lymphomas |
| B62y.00 | Malignant lymphoma NOS |
| B62y000 | Malignant lymphoma NOS of unspecified site |
| B62y100 | Malignant lymphoma NOS of lymph nodes of head, face and neck |
| B62y200 | Malignant lymphoma NOS of intrathoracic lymph nodes |
| B62y300 | Malignant lymphoma NOS of intra-abdominal lymph nodes |
| B62y400 | Malignant lymphoma NOS of lymph nodes of axilla and arm |
| B62y500 | Malignant lymphoma NOS of lymph node inguinal region and leg |
| B62y600 | Malignant lymphoma NOS of intrapelvic lymph nodes |
| B62y700 | Malignant lymphoma NOS of spleen |
| B62y800 | Malignant lymphoma NOS of lymph nodes of multiple sites |
| B62yz00 | Malignant lymphoma NOS |
| B62z.00 | Malignant neoplasms of lymphoid and histiocytic tissue NOS |
| B62z000 | Unspec malig neop lymphoid/histiocytic of unspecified site |
| B62z100 | Unspec malig neop lymphoid/histiocytic lymph node head/neck |
| B62z200 | Unspec malig neop lymphoid/histiocytic of intrathoracic node |
| B62z300 | Unspec malig neop lymphoid/histiocytic intra-abdominal nodes |
| B62z400 | Unspec malig neop lymphoid/histiocytic lymph node axilla/arm |
| B62z500 | Unspec malig neop lymphoid/histiocytic nodes inguinal/leg |
| B62z600 | Unspec malig neop lymphoid/histiocytic of intrapelvic nodes |
| B62z700 | Unspec malig neop lymphoid/histiocytic of spleen |
| B62z800 | Unspec malig neop lymphoid/histiocytic of multiple sites |
| B62zz00 | Lymphoid and histiocytic malignancy NOS |
| B62zz11 | Immunoproliferative neoplasm |
| B63..00 | Multiple myeloma and immunoproliferative neoplasms |
| B630.00 | Multiple myeloma |
| B630.11 | Kahler's disease |
| B630.12 | Myelomatosis |
| B630000 | Malignant plasma cell neoplasm, extramedullary plasmacytoma |
| B630100 | Solitary myeloma |
| B630200 | Plasmacytoma NOS |
| B630300 | Lambda light chain myeloma |
| B631.00 | Plasma cell leukaemia |
| B63y.00 | Other immunoproliferative neoplasms |
| B63z.00 | Immunoproliferative neoplasm or myeloma NOS |
| B64..00 | Lymphoid leukaemia |
| B64..11 | Lymphatic leukaemia |
| B640.00 | Acute lymphoid leukaemia |
| B641.00 | Chronic lymphoid leukaemia |
| B641.11 | Chronic lymphatic leukaemia |
| B642.00 | Subacute lymphoid leukaemia |
| B64y.00 | Other lymphoid leukaemia |
| B64y000 | Aleukaemic lymphoid leukaemia |
| B64y100 | Prolymphocytic leukaemia |
| B64y200 | Adult T-cell leukaemia |
| B64yz00 | Other lymphoid leukaemia NOS |
| B64z.00 | Lymphoid leukaemia NOS |
| B65..00 | Myeloid leukaemia |
| B650.00 | Acute myeloid leukaemia |
| B651.00 | Chronic myeloid leukaemia |
| B651.11 | Chronic granulocytic leukaemia |
| B651000 | Chronic eosinophilic leukaemia |
| B651200 | Chronic neutrophilic leukaemia |
| B651z00 | Chronic myeloid leukaemia NOS |
| B652.00 | Subacute myeloid leukaemia |
| B653.00 | Myeloid sarcoma |
| B653000 | Chloroma |
| B653100 | Granulocytic sarcoma |
| B653z00 | Myeloid sarcoma NOS |
| B65y.00 | Other myeloid leukaemia |
| B65y000 | Aleukaemic myeloid leukaemia |
| B65y100 | Acute promyelocytic leukaemia |
| B65yz00 | Other myeloid leukaemia NOS |
| B65z.00 | Myeloid leukaemia NOS |
| B66..00 | Monocytic leukaemia |
| B66..11 | Histiocytic leukaemia |
| B66..12 | Monoblastic leukaemia |
| B660.00 | Acute monocytic leukaemia |
| B661.00 | Chronic monocytic leukaemia |
| B662.00 | Subacute monocytic leukaemia |
| B66y.00 | Other monocytic leukaemia |
| B66y000 | Aleukaemic monocytic leukaemia |
| B66yz00 | Other monocytic leukaemia NOS |
| B66z.00 | Monocytic leukaemia NOS |
| B67..00 | Other specified leukaemia |
| B670.00 | Acute erythraemia and erythroleukaemia |
| B670.11 | Di Guglielmo's disease |
| B671.00 | Chronic erythraemia |
| B671.11 | Heilmeyer - Schoner disease |
| B672.00 | Megakaryocytic leukaemia |
| B672.11 | Thrombocytic leukaemia |
| B673.00 | Mast cell leukaemia |
| B674.00 | Acute panmyelosis |
| B675.00 | Acute myelofibrosis |
| B67y.00 | Other and unspecified leukaemia |
| B67y000 | Lymphosarcoma cell leukaemia |
| B67yz00 | Other and unspecified leukaemia NOS |
| B67z.00 | Other specified leukaemia NOS |
| B68..00 | Leukaemia of unspecified cell type |
| B680.00 | Acute leukaemia NOS |
| B681.00 | Chronic leukaemia NOS |
| B682.00 | Subacute leukaemia NOS |
| B68y.00 | Other leukaemia of unspecified cell type |
| B68z.00 | Leukaemia NOS |
| B69..00 | Myelomonocytic leukaemia |
| B690.00 | Acute myelomonocytic leukaemia |
| B691.00 | Chronic myelomonocytic leukaemia |
| B692.00 | Subacute myelomonocytic leukaemia |
| B6y..00 | Malignant neoplasm lymphatic or haematopoietic tissue OS |
| B6y0.00 | Myeloproliferative disorder |
| B6y0.11 | Myeloproliferative disease |
| B6y1.00 | Myelosclerosis with myeloid metaplasia |
| B6y1.11 | Megakaryocytic myelosclerosis |
| B6z..00 | Malignant neoplasm lymphatic or haematopoietic tissue NOS |
| B6z0.00 | Kaposi's sarcoma of lymph nodes |
| D415100 | Chronic congestive splenamegaly |
| D415400 | Splenic atrophy |
| D415600 | Splenic fibrosis |
| G74y600 | Embolism and thrombosis of the splenic artery |
| PK01.00 | Absent spleen |
| PK01.11 | Asplenia |
