# Supplementary material for: Resource use and direct medical costs of acute respiratory illness in the UK based on linked primary and secondary care records from 2001 to 2009
Source: PLoS One. 2020 Aug 6;15(8):e0236472. doi: 10.1371/journal.pone.0236472 (PMC7410242; doi:10.1371/journal.pone.0236472)
Supplement: S2 Data — (DOCX) [file pone.0236472.s002.docx]

**Ambulatory healthcare resource use and direct medical costs of acute respiratory illness during influenza seasons from 2001 to 2009 in the CPRD/HES population and extrapolated to the UK population**

|  | **CPRD/HES** | **Extrapolated** |
| --- | --- | --- |
| **GP consultations** |  |  |
| Number with ≥ 1 GP consultation | 156,193 | 5,664,963 |
| Absolute number of visits | 178,304 | 6,466,907 |
| Absolute cost of visits | £6,418,944 | £232,808,644 |
| Mean number of GP surgery visits (SD) | 1.1 (0.44) | — |
| Mean (SD) total costs | £6,185,243 (£2,474,097) | £224,332,544 (£89,733,010) |
| **GP prescriptions** |  |  |
| Had ≥ 1 prescription, n (%) | 82,204 (52.6) | 2,981,456 |
| Number of prescriptions | 209,160 | 7,586,023 |
| Total cost of prescriptions | £87,193 | £3,162,402 |
| Mean number of prescriptions per patient (SD) | 2.5 (3.0) | — |
| Average cost per prescription (SD) | £0.84 (£3.24) | — |
| **Total GP costs** |  |  |
| Cost of GP visit including prescription | £6,506,137 | £235,971,046 |
| Number with ≥ 1 outpatient clinic visit | 574 | 20,818 |
| **Out-patient clinic visits** |  |  |
| Number of outpatient clinic visits | 618 | 22,414 |
| Cost of outpatient clinic visits | £90,846 | £3,294,893 |
| Mean no. of outpatient clinic visits/patient (SD) | 1.1 (0.36) | – |
| Mean (SD) total costs | £92,816 (£30,376) | £3,366,343 (£1,101,707) |

SD, standard deviation; CPRD/HES, Clinical Practice Research Datalink/ Hospital Event Statistics database; GP, general practitioner
